# Occipital condyle width (OCW) is a highly accurate predictor of body mass in therian mammals

**DOI:** 10.1186/s12915-021-01224-9

**Published:** 2022-02-07

**Authors:** Russell K. Engelman

**Affiliations:** Department of Biology, Case Western Reserve University, 10900 Euclid Avenue, Cleveland, OH 44106 USA

**Keywords:** Body size, Mass estimation, Allometry, Non-linear regression, Non-linear allometry

## Abstract

**Background:**

Body mass estimation is of paramount importance for paleobiological studies, as body size influences numerous other biological parameters. In mammals, body mass has been traditionally estimated using regression equations based on measurements of the dentition or limb bones, but for many species teeth are unreliable estimators of body mass and postcranial elements are unknown. This issue is exemplified in several groups of extinct mammals that have disproportionately large heads relative to their body size and for which postcranial remains are rare. In these taxa, previous authors have noted that the occiput is unusually small relative to the skull, suggesting that occiput dimensions may be a more accurate predictor of body mass.

**Results:**

The relationship between occipital condyle width (OCW) and body mass was tested using a large dataset (2127 specimens and 404 species) of mammals with associated in vivo body mass. OCW was found to be a strong predictor of body mass across therian mammals, with regression models of Mammalia as a whole producing error values (~ 31.1% error) comparable to within-order regression equations of other skeletal variables in previous studies. Some clades (e.g., monotremes, lagomorphs) exhibited specialized occiput morphology but followed the same allometric relationship as the majority of mammals. Compared to two traditional metrics of body mass estimation, skull length, and head-body length, OCW outperformed both in terms of model accuracy.

**Conclusions:**

OCW-based regression models provide an alternative method of estimating body mass to traditional craniodental and postcranial metrics and are highly accurate despite the broad taxonomic scope of the dataset. Because OCW accurately predicts body mass in most therian mammals, it can be used to estimate body mass in taxa with no close living analogues without concerns of insufficient phylogenetic bracketing or extrapolating beyond the bounds of the data. This, in turn, provides a robust method for estimating body mass in groups for which body mass estimation has previously been problematic (e.g., “creodonts” and other extinct Paleogene mammals).

**Supplementary Information:**

The online version contains supplementary material available at 10.1186/s12915-021-01224-9.

## Background

Body size (body mass) is a particularly important feature of an organism’s biology, as it is correlated with dietary habits [1–4], basal metabolic rate [5], population density [6], longevity [7], reproductive rate [8], home range size [9], degree of sexual size dimorphism [10], relative brain size [11, 12], morphology and degree of morphological specialization [13], defensive behavior [14], guild structure [15, 16], isotope enrichment ratios [17], and extinction risk [18], among various other factors (see [19–21] and references therein). Indeed, many authors have gone so far as to say that body mass is the single most important aspect of the biology of any organism [22–29]. As a result, estimations of body mass are of extreme importance when studying the paleobiology and paleoecology of a given species, both in terms of how it affects the taxon’s biology and how the taxon interacts with other species in its community.

**Fig. 1:**
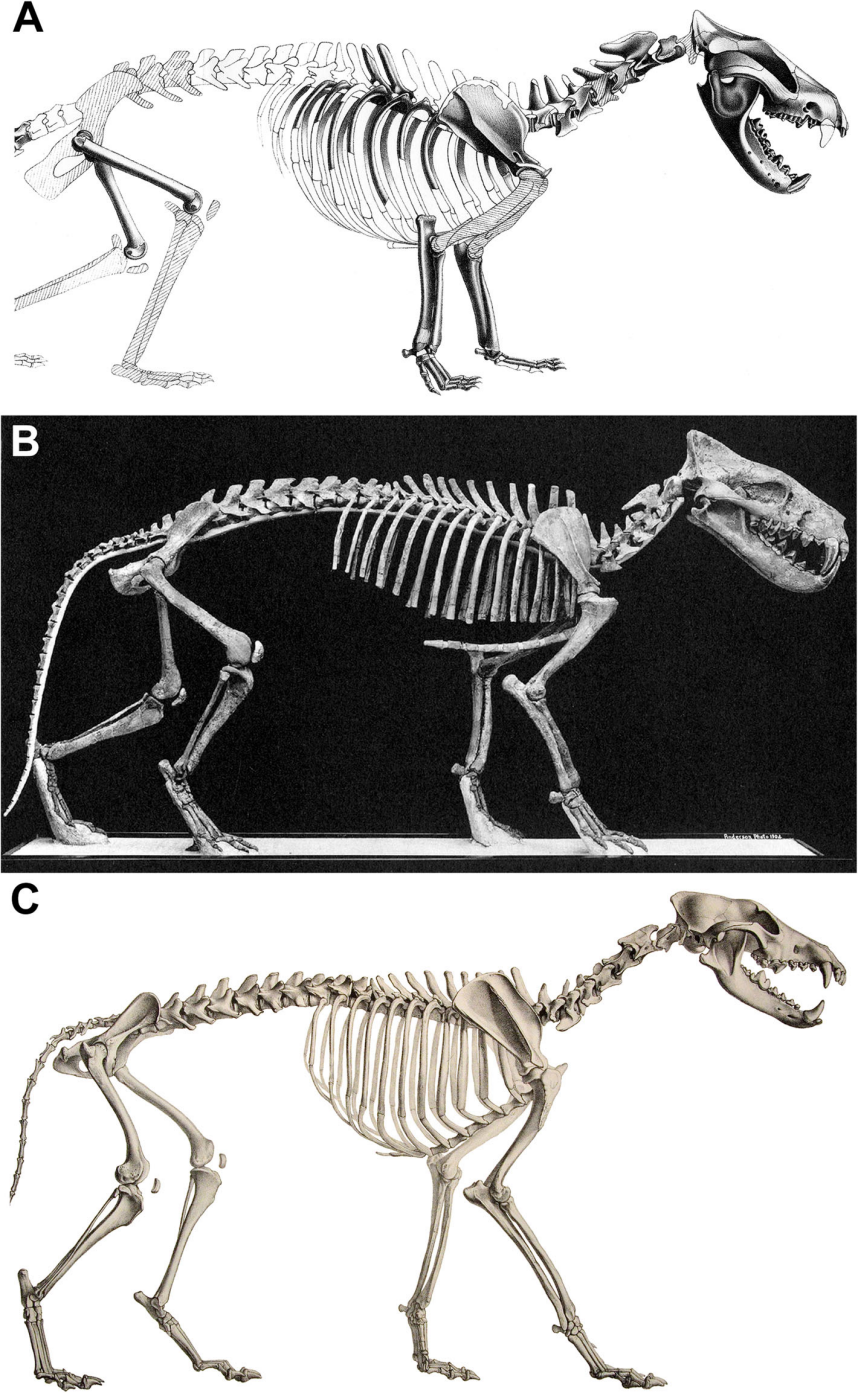
Skeletal reconstructions of a borhyaenid sparassodont (**A**, *Borhyaena tuberata*; modified from Sinclair [33]), hyaenodont “creodont” (**B**, *Hyaenodon horridus*; modified from Scott and Jepsen [34]), and canid carnivoran (**C,** *Canis lupus*, public domain from Wikimedia Commons), scaled to the same thorax length (not head-body length, due to differences in relative neck length in the three taxa), illustrating the proportionally larger heads of *Borhyaena* and *Hyaenodon*

The most common method of estimating body mass in extinct animals is to use a regression equation based on skeletal measurements and body mass from a comparative sample of closely related extant species (which are often assumed to have geometric similarity). For fossil mammals, these estimates are often based on teeth, which are commonly preserved [30] and are often the only fossil remains known for many species. However, regression equations based on teeth can be problematic when trying to apply them to mammals that have no close living relatives [31, 32]. Furthermore, many of these extinct animals may exhibit dental morphologies, body proportions, and patterns of allometric scaling unlike any living species. For example, many groups of extinct mammals have disproportionately large heads relative to extant species (Fig. 1). This phenomenon has been most extensively discussed in extinct carnivorous mammals, such as sparassodonts [35, 36], mesonychians [37], and oxyaenid [38] and hyaenodont [32, 39] “creodonts,” as well as some extinct carnivorans such as amphicyonids [40, 41] and nimravids [39]. However, this condition also occurs in pantodonts [42, 43], “condylarths” [44], taeniodonts [45], entelodonts [46], diprotodontoid marsupials [47], South American endemic ungulates [48–52], large-bodied rodents [53, 54], and Malagasy subfossil lemurs [55], among others. Given the disproportionately large heads of these taxa, body mass estimates based on craniodental regression equations derived from modern taxa are thought to overestimate body mass (see [32, 39, 56]).

Because of these difficulties with craniodental measurements many authors have considered head-body length (HBL) or postcranial measurements such as the length, diameter, or cross-sectional area of long bone diaphysis or articular surfaces of limb bones to be better estimators of body mass [32, 55, 57–59]. However, body mass estimates based on postcranial measurements present their own difficulties, which have often been under-appreciated and rarely discussed in the literature. Perhaps most importantly, the postcranium in most species of fossil mammals is either poorly known or represented by very fragmentary material, and the postcranial anatomy of even some higher-level clades remains more or less unknown (e.g., the notoungulate family Archaeohyracidae [60, 61]). This is because taxonomic diagnoses of most extinct mammal are almost exclusively based on craniodental features, with postcranial remains usually only identified to genus or species if they directly associated with craniodental material [62–65; E. Davis, pers. comm., 2018]. Even if postcranial remains are better predictors of body mass in mammals, it is a moot point in terms of estimating body mass if no postcrania are known for the taxon.

This scarcity of postcranial remains particularly hinders attempts to use HBL to estimate body mass, a measurement which has otherwise been suggested to be one of the best estimators of body mass in fossil mammals [52, 66]. HBL can only be accurately measured on a nearly complete, undistorted skeleton with a complete spinal column and as a result can only be applied to extremely well-known taxa [67, 68] (see also Sarko et al. [69] for discussion of a comparable issue in sirenians). Even well-preserved taxa are often missing one or more vertebrae and must be reconstructed by filling in missing parts with ones based on those of close relatives, which can affect body mass estimates. For example, Sinclair [33] originally restored the sparassodont *Borhyaena tuberata* with parts of *Prothylacynus patagonicus* and *Thylacinus cynocephalus*, whereas Argot [70] restored *B. tuberata* with a much shorter torso and longer limbs based on extrapolation from the known limb and vertebral dimensions of this taxon. Using the all-taxon HBL regression equation for carnivorous mammals in Van Valkenburgh [39], the reconstruction of *B. tuberata* in Sinclair [33] produces a body mass estimate of 22.88 kg whereas that in Argot [70] using the same equation produces a body mass estimate of 18.36 kg, nearly 5 kg (or 20%) lighter. Another issue is that HBL includes the length of the cranium as well as the body as a part of formulating this measurement. Thus, HBL is influenced by skull size in the same manner as craniodental measurements and can produce unreliable body mass estimates in large-headed mammals [32, 56].

Furthermore, although postcranial body mass estimates are often regarded as being more independent of phylogeny or biology than craniodental measurements, limb bone dimensions are still influenced by these factors. Good example of this are xenarthrans and caviomorph rodents, which have disproportionately robust hindlimbs relative to their body size [31, 71], likely because these animals often feed or mate in a bipedal stance and therefore must occasionally support all of their weight on their hindlimbs [71–73], which violates the assumption that weight is being distributed in a comparable manner across the fore- and hindlimbs in Mammalia. In particular, Millien and Bovy [31] found that extinct giant caviomorphs like *Phoberomys pattersoni* have hindlimb bones that are disproportionately robust to their body size even relative to extant caviomorphs, which according to these authors may have produced inaccurate body mass estimates for this taxon. This is demonstrated in the fact that, due to their unusually robust hindlimbs, body mass estimates for glyptodonts and extinct giant caviomorphs like *Phoberomys* based on the femur range from 70 to 380% higher than estimates based on the humerus [74, 75]. Compounding problems with the influence of ecology or phylogenetic signal is the fact that most extant large mammals, such as artiodactyls, equids, many carnivorans, and even rhinocerotids to some degree [76, 77], are cursorial and have relatively gracile limbs. By contrast, most of the extinct mammal groups that researchers are frequently interested in estimating body mass for, such as “creodonts” [78, 79], sparassodonts, mesonychians [37], pantodonts [80], extinct Paleogene or South American ungulates [50, 52, 81], caviomorph rodents [31], xenarthrans [74, 82], tend to be more ambulatory and have more robust limbs than extant large mammals. As a result, the limb dimensions of large extant mammals may not reflect the proportions of extinct taxa, and this is likely to cause errors in body mass estimation if the two are assumed to be directly comparable [31, 32, 37, 74, 82].

A related issue is one of phylogenetic bracketing. Phylogenetic bracketing is a key concept in modern paleontology, for if a biological inference can be applied to two distinct branches of a phylogeny, it also likely applies to the extinct taxa between them as well [83]. However, many prior studies estimating body mass in wholly extinct groups of mammals often estimate mass based on regression equations derived (often by necessity) from unrelated species that do not bracket the taxon of study. For example, body masses in “creodonts” and sparassodonts have often been estimated based on regression equations derived from distantly related carnivorans, didelphimorphians, and dasyuromorphians (e.g., [32, 39, 84]), and body masses in extinct hyracoids and South American ungulates have typically been estimated based on regression equations derived from perissodactyls and artiodactyls (e.g., [81, 85, 86]). Very rarely do studies examine if the relationships in their regression equations can be more broadly applied across Mammalia or are only applicable within their respective clade (with some exceptions including [58, 87, 88]).

Even when phylogenetic bracketing is present it may not be sufficient if the variables are not broadly applicable. For example, McGrath et al. [81] noted that both postcranial and craniodental variables failed to produce reliable body mass estimates in macraucheniid litopterns, due to unique features of macraucheniids (robust limbs and complete, closed dentitions) that are not present in most extant ungulates. Similarly, Croft et al. [52] found that craniodental equations likely overestimated body mass in notoungulates due to characteristics of notoungulates not present in modern ungulates (namely large heads relative to body size). This is despite the fact that the equations used to calculate these estimates were based on perissodactyls and artiodactyls, which phylogenetically bracket litopterns and notoungulates [89, 90]. As a result, when estimating the body mass of species belonging to wholly extinct groups, it is critical to use variables that can be confidently applied across Mammalia more generally and are not specific to a particular group.

Because of these issues, interest in potential alternative methods of estimating body mass in mammals has been steadily increasing. Recent studies have suggested that dimensions of the scapula [91], astragalus [58, 92], and calcaneus [93] may all be strong predictors of body mass. Another potential alternative to traditional craniodental and postcranial-based methods of estimating body mass, especially for the aforementioned extinct “large-headed” taxa, are dimensions of the occiput. Argot and Babot [94] noted that although the heads of the hyaenodont “creodont” *Hyaenodon* and the sparassodont *Callistoe* are relatively large for their body size, the occiput appeared unusually small, resembling the overall disparity in size between the cranium and postcranium in these taxa. This suggests that dimensions of the occiput may scale with the size of the postcranium, rather than the cranium, and therefore may be a more accurate proxy of body size than other craniodental measurements, particularly in these large-headed extinct mammals.

There are several reasons to believe that occiput dimensions may be good estimators of body size. Because the atlanto-occipital joint is the link between the postcranium and cranium, dimensions of the occiput might be expected to more closely correlate with postcranial proportions than other craniodental measurements, as the occiput is constrained by the size of the spinal column. The occiput also shares a common developmental origin with the vertebral column separate from the rest of the skull, as the post-otic region of the skull (including the occiput) is formed by the incorporation of the anteriormost trunk somites into the cranium [95, 96]. Hence, the dimensions of the occiput can be thought of as postcranial landmarks measurable on the cranium. All of the nerves that innervate the postcranium (with the exception of the vagus nerve) pass through the foramen magnum, in addition to the vertebral arteries, anterior and posterior spinal arteries, tectorial membranes, and alar ligaments, among other structures. Given that the number of neurons per unit mass of postcranial body tissue is relatively consistent within mammals [97], this means that the size of the foramen magnum and its surrounding structures (i.e., the occiput) would be expected to closely correlate with body size (but see [98]).

More broadly, the postcranium of most terrestrial mammals is also relatively conservative, with most species exhibiting a relatively short neck with seven cervical vertebrae, 19–20 thoracolumbar vertebrae [99], a reduced or absent tail that contributes little to body mass compared to other chordates, and four limbs of roughly comparable size. Specifically, with regard to the tail, mammals exhibit a reduction in overall tail robustness compared to other tetrapods [100], driven by factors such as a more gracile caudal skeleton, a reduction of caudal musculature such as the reptilian caudofemoralis, and the fact that, unlike limbed squamates and crocodilians, most mammals do not use the tail as a major fat-storing organ (with some exceptions, see [101]). The reptilian caudofemoralis alone (which is not homologous to the caudofemoralis muscle in mammals and is actually absent in the latter) comprises about 1/3 of total caudal muscle mass in most non-avian sauropsids and in *Iguana iguana* represents ~ 3.6% of total body weight ([100, 102]). In non-avian sauropsids, the tail is typically 20% or more of total body mass (Table 1), whereas even in mammals with relatively long, muscular tails like *Ateles* the tail is no more than 8% of the total body mass (and it is typically less than 5% in mammals without prehensile tails). As a result, the body proportions of mammals are less variable than those of most other tetrapods and thus there are fewer potentially confounding variables when estimating body size based on axial dimensions (e.g., variation in tail size, presacral vertebral counts, or bipedalism versus quadrupedalism in reptiles [22, 112, 113];).

**Table 1:** Comparison of tail masses as a percent of total body mass in mammals and non-mammalian tetrapods. Note that the available data for mammals is disproportionately focused on taxa with large tails (Macropodiformes and prehensile-tailed taxa), the average mammal (e.g., *Canis*, *Felis*, *Peromyscus*) typically has a much smaller tail

Taxon	Group	Family	% Tail mass	Reference
*Alligator mississippiensis*	Sauropsida	Alligatoridae	24.5%	[103]
*Alligator mississippiensis*	Sauropsida	Alligatoridae	27.8%	[104]
*Iguana iguana*	Sauropsida	Iguanidae	18.8%	[102]
*Christinus marmoratus*	Sauropsida	Gekkonidae	20–24%	[105]
*Eublepharis macularius*	Sauropsida	Gekkonidae	22%	[106]
*Plethodon cinereus*	Caudata	Plethodontidae	15–20%	[107]
*Macaca fascicularis*	Mammalia	Cercopithecidae	4%	[108]
*Macaca fuscata*	Mammalia	Cercopithecidae	0.1%	[108]
*Macaca nemestrina*	Mammalia	Cercopithecidae	0.2%	[108]
*Ateles* sp.	Mammalia	Atelidae	7.8%	[108]
*Alouatta caraya*	Mammalia	Atelidae	5.5%	[108]
*Cebus* sp.	Mammalia	Cebidae	5.4%	[108]
*Saguinus Oedipus*	Mammalia	Callitrichidae	3.0%	[108]
*Aotus trivirgatus*	Mammalia	Aotidae	4.2%	[108]
*Perodicticus potto*	Mammalia	Lorisidae	0.4%	[108]
*Otolemur crassicaudatus*	Mammalia	Galagidae	4.3%	[108]
*Galago senegalensis*	Mammalia	Galagidae	2.5%	[108]
*Tupaia glis*	Mammalia	Tupaiidae	2.6%	[108]
*Dasyprocta aguti*	Mammalia	Caviidae	< 0.1%	[102]
*Dolichotis salinicola*	Mammalia	Caviidae	0.0%	[102]
*Dinomys branickii*	Mammalia	Dinomyidae	1.1%	[109]
*Erethizon dorsatum*	Mammalia	Erethizontidae	3.3%	[109]
*Coendou prehensilis*	Mammalia	Erethizontidae	8.7%	[109]
*Peromyscus maniculatus*	Mammalia	Cricetidae	1.0–4.0%	[110]
*Canis familiaris*	Mammalia	Canidae	0.4%	[108]
*Felis catus*	Mammalia	Felidae	1.3%	[108]
*Bradypus variegatus*	Mammalia	Bradypodidae	< 0.1%	[102]
*Choloepus hoffmanni*	Mammalia	Choloepodidae	< 0.1%	[102]
*Didelphis marsupialis*	Mammalia	Didelphidae	3.0%	[108]
*Macropus rufus*	Mammalia	Macropodidae	7.0%	[111]
*Dendrolagus matschiei*	Mammalia	Macropodidae	5.0%	[111]
*Potorous apicalus*	Mammalia	Potoroidae	3.0%	[111]
*Pseudocheirus peregrinus*	Mammalia	Pseudocheiridae	7.0%	[111]

Additionally, there is likely to be very strong stabilizing selection on the occiput. Maintaining function of the atlanto-occipital joint is critical for an individual’s fitness, as luxation of this joint is almost invariably fatal [114]. Any mutation that compromised occiput function would be rapidly removed from the gene pool and as a result morphological change in this structure due to genetic drift would be low. This suggests that occiput evolution would be highly conservative and thus the occiput may be a good proxy for estimating body mass across a broad range of mammals. While it would be theoretically possible for selection to favor an occiput that is disproportionately large relative to body size (as might be expected if there were very strong stresses at the atlanto-occipital joint, such as perhaps in some horned artiodactyls; [115]), it is unlikely that many animals would have occiputs that are disproportionately small relative to body size. This is because if an animal had a disproportionately small occiput relative to its head and body it would result in a greater amount of force being applied to a smaller joint surface and thus increase the risk of atlanto-occipital luxation. Additionally, a smaller occiput would result in greater transverse torque at the atlanto-occipital joint when mediolateral forces are applied at the anterior end of the skull (as in during prey capture, inter/intraspecific combat, or otherwise interacting with a resistant object) due to the narrower distance between the condyles relative to the anteroposterior length of the skull resulting in a less stable joint. This would be even more pronounced in large-headed species because the moment arm (the anteroposterior length of the skull) is inherently longer. Therefore, if an animal has an occiput that is small relative to skull size, it is more likely that the animal merely has a disproportionately large head, with the occiput dimensions being constrained by the size of the spinal column, rather than the animal having a disproportionately small occiput relative to its body. This agrees with what is observed in taxa like *Callistoe* and *Hyaenodon*.

One measurement of the occiput that may prove particularly useful for estimating body mass in fossil mammals is occipital condylar width (hereafter abbreviated as OCW). Martin [116] used OCW to estimate body mass in extinct mammals; however, these regressions were based on a relatively small (*N* = 26), taxonomically restricted sample. After Martin [116], only a few studies have used dimensions of the occipital condyles to estimate body mass in extinct terrestrial mammals [65, 117–121]. OCW has been used more frequently to predict body mass in marine mammals (cetaceans, [122–124]; sirenians, [69, 125]; pinnipeds, [126, 127]). This is in stark contrast to the large number of studies that have estimated body mass of terrestrial mammals via dimensions of postcrania, teeth, and measurements such as HBL or skull length. Many multivariate studies of body mass based on craniodental or whole-body metrics do not even consider dimensions of the occiput outside of occiput height [86, 128]. The applicability of OCW across mammals more generally has never been tested, though it has been suggested [129]. In this study, I examine the allometric relationship between OCW and body mass in a wide range of extant mammals, calculate regression equations based on these data, and compare the accuracy of these regression equations with previous studies.

## Results

### Data distribution and model fitting

**Fig. 2:**
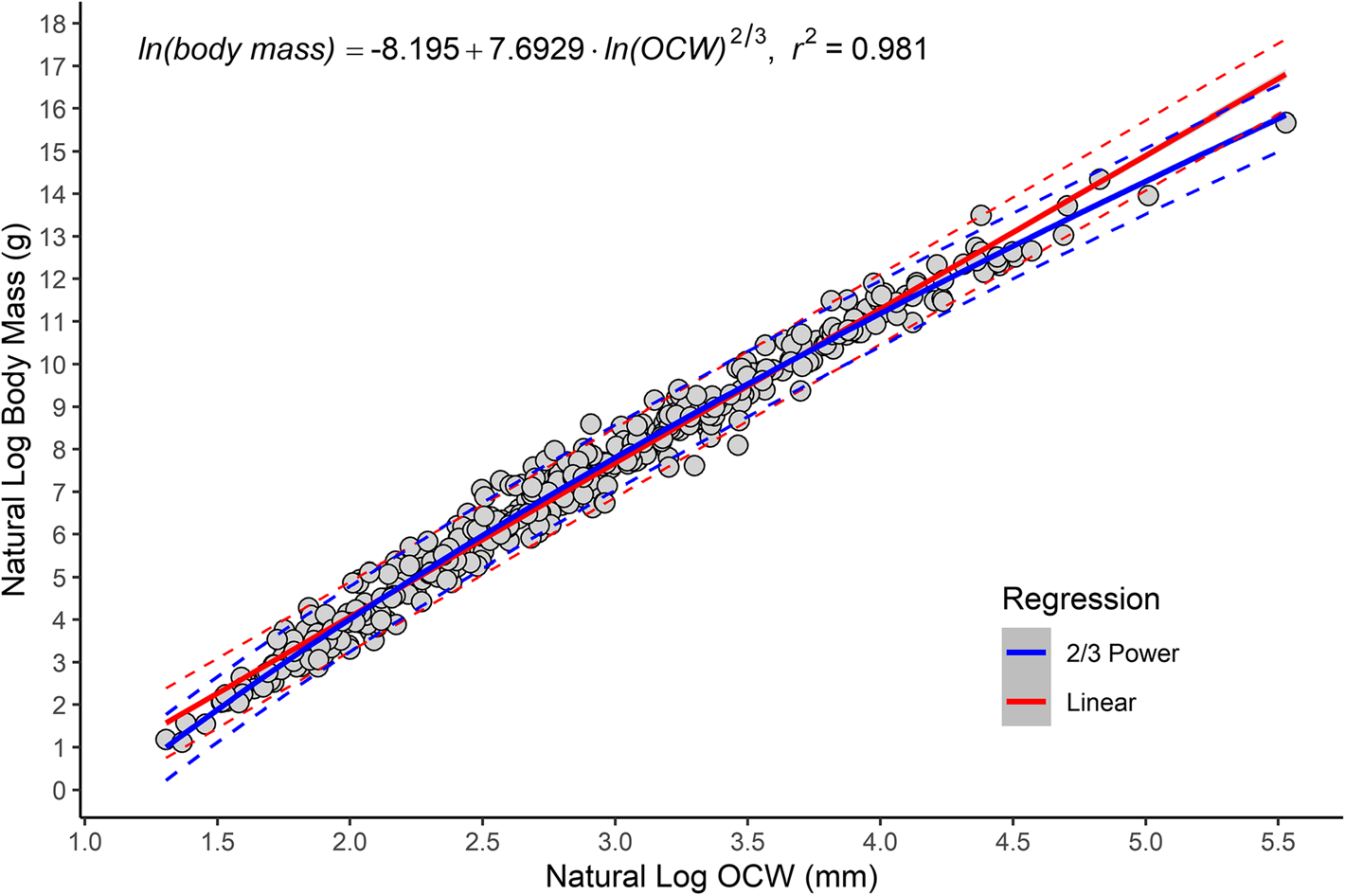
Scatterplot of natural log of OCW versus natural log of body mass, showing the best fit (natural log OCW raised to the 2/3 power) regression line for all species and the non-linear distribution of the data. Linear regression is in red and 2/3 power regression is in blue. Dashed lines represent 95% prediction intervals. Most of the species located above the upper bounds of the prediction interval are lagomorphs (see Fig. 7)

A strong correlation exists between OCW and body mass (Fig. 2). However, the relationship between the two variables is not log-linear. Instead, plotting ln OCW against ln body mass shows the points form a curvilinear distribution that is slightly concave down, with larger mammals having proportionally larger OCW relative to body size (Fig. 2). This is supported by a general observation made during data collection that larger taxa had proportionally larger occipital condyles. For example, in the present study, the occipital condyles represent a proportionally smaller part of OCW in smaller mammals like *Reithrodontomys megalotis* (27.9%) and *Tarsipes rostratus* (35.4%), whereas in larger mammals like *Cervus canadensis* (55.6%), *Ursus americanus* (48.4%), and *Diceros bicornis* (55.1%) the occipital condyles comprise a greater proportion of OCW.

Assuming a log-linear model, the best-fit line systematically overestimates body mass at the extremes of the dataset and underestimates values for taxa closer to the midpoint (Additional file 1). The effects of non-linearity in the data after log-transformation can be best seen in the largest taxon in the dataset, *Loxodonta africana*, which also exhibits one of the largest absolute residuals under a log-linear regression model. The OCW of *L. africana* is nearly 75 mm wider than would be predicted based on a log-linear model (250 mm versus 175 mm), and body mass under a log-linear model is overestimated by 64% (Additional file 2). For *Ursus maritimus*, the largest taxon in this dataset for which *N* > 2, the difference in predicted versus actual OCW is less extreme (6 mm, or 7% of actual OCW), but still produces an underestimate of body mass (especially compared to the final non-linear model used here). The same issue is present for the smallest mammals in this study, though is less obvious in magnitude due to the differences in scales involved. Overall, however, the data seems to curve significantly more at its upper extreme than its lower one.

**Fig. 3:**
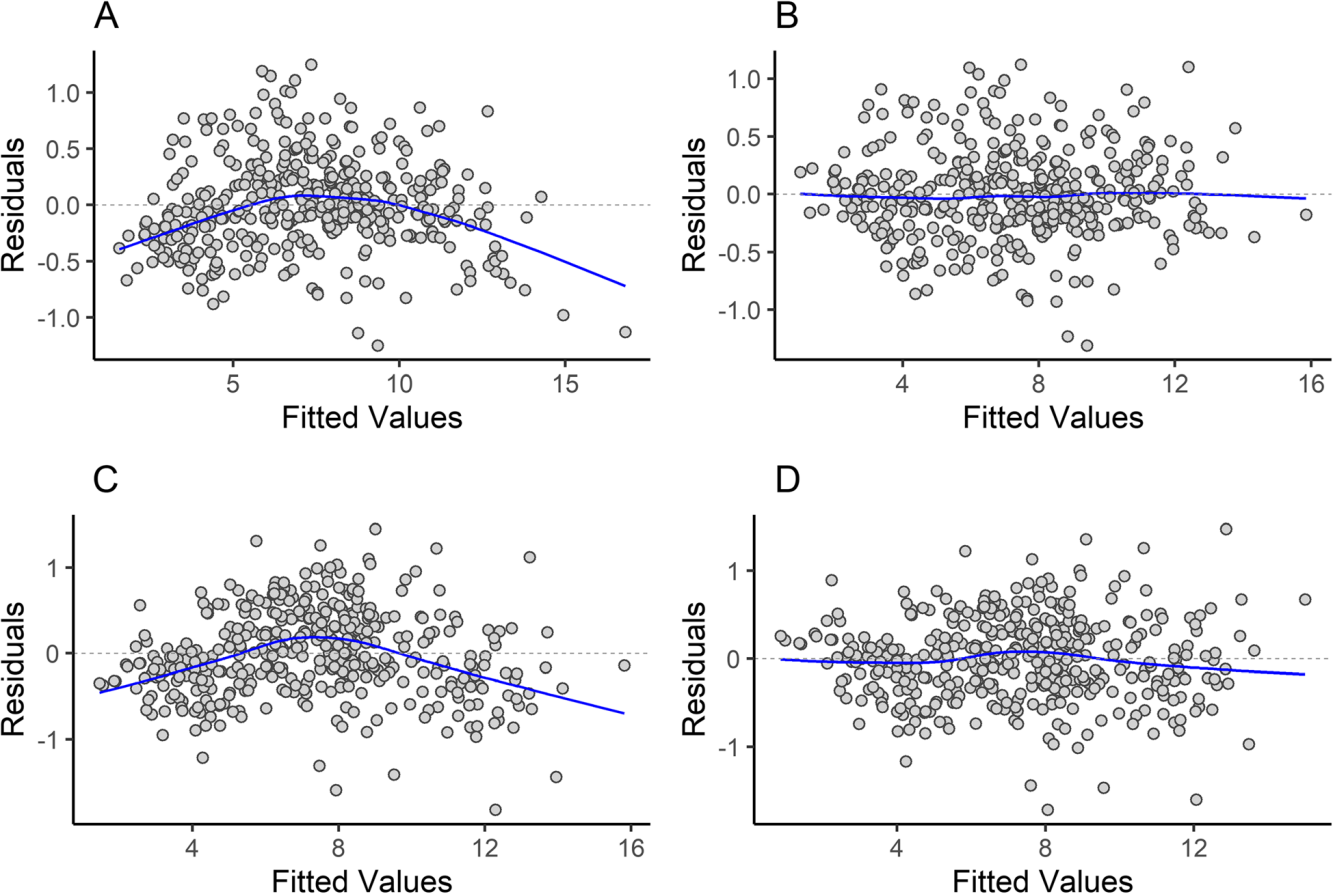
Residuals versus fitted plot for the regression of OCW (**A,B**) or skull length (**C,D**) against body mass. **A** and **C** represent residuals versus fitted graphs for regression lines where isometry is assumed, and **B** and **D** represent graphs with the natural log of the independent variable raised to the 2/3 power (in **B**) or the 1/2 power (in **D**)

When comparing several different regression models, a log-power model in which natural log OCW was raised to the 2/3 power significantly outperformed a log-linear one in terms of %PE, %SEE, log likelihood, AIC, and BIC (Table 2). The next best-fitting model was a log-quadratic model (Additional file 3), which had comparable %PE and %SEE but higher log likelihood, AIC, and BIC. The residuals versus fits plot for a log-linear model between OCW and body mass shows a distinctly non-linear, heteroskedastic relationship (Fig. 3a), whereas under a 2/3 power model (see Fig. 3b) or a log-quadratic model this distribution is linearized. Empirical curve fitting of a power rule using the non-linear least squares (*nls*) function in R produced a model with an exponent of 0.688 (Table 3), very close to the exponent expected if the data scaled to the 2/3 power (0.667). The 95% confidence interval for the exponent (Table 3) rules out a strictly linear regression line, though it cannot fully rule out a 3/4 power scaling relationship. Comparing the models under ANOVA found the log-quadratic and 2/3 power model to be non-significantly different (F = 0.3243, *p* = 0.5694), but the 2/3 power model is preferred here for reasons that will be detailed below. Unless otherwise mentioned, the results of this study refer to the model where log OCW is transformed by being raised to the 2/3 power before regression.

**Table 2:** Accuracy statistics for the regression model between natural log OCW and natural log body mass using the all taxon, species average dataset under several different ordinary least squares (OLS) and phylogenetic least squares (PGLS) regression models

Method	Regression	AIC	BIC	logLik	df	*r*^2^_adj_	%PE	CF	%PE_cf_	%SEE
OLS	Linear	441	453	− 217	402	0.9784	34.88	0.905	38.99	51.50
OLS	1/2 power	409	421	− 201	402	0.9810	32.70	1.145	31.28	49.09
OLS	1/3 power	455	467	− 224	402	0.9777	35.22	1.260	33.80	52.63
OLS	2/3 power	389	401	− 192	402	0.9811	32.03	1.047	31.09	47.69
OLS	3/4 power	391	403	− 193	402	0.9809	32.21	1.004	32.10	47.82
OLS	Quadratic	391	407	− 192	401	0.9810	32.05	1.068	30.98	47.73
OLS	Cubic	393	413	− 191	400	0.9809	32.04	1.055	31.05	47.79
PGLS	Linear	420	432	− 207	402	–	70.12	1.725	34.30	309.11
PGLS	1/3 power	367	379	− 180	402	–	75.61	1.953	32.95	276.45
PGLS	2/3 power	368	380	− 181	402	–	68.88	1.754	31.56	276.00
PGLS (OU)	2/3 power	394	406	− 194	402	–	32.00	1.046	31.08	47.45

Abbreviations: *PGLS (OU)*, phylogenetic least squares under an Ornstein-Uhlenbeck model (rather than Brownian); *AIC*, Akaike Information Criterion; *BIC*, Bayesian Information Criterion; *logLik*, log likelihood; *df*, degrees of freedom; *r*^*2*^_*adj*_, adjusted *r*^2^ value; *%PE*, percent prediction error; *CF*, averaged correction factor (see “Methods”); *%PE*_*cf*_, percent prediction error after applying correction factor; *%SEE*, standard error of estimate

**Table 3:** Results of non-linear curve fitting of OCW, CBL, and HBL. The first seven regression equations are regressing natural log OCW, CBL, or HBL (*x* variables) against natural log body mass (*y* variable, in g). The last two equations are regressing natural log OCW against natural log CBL and natural log HBL, respectively. All equations are written in the form ln(*y*) = *a* × ln(*x*)^*b* + *c*

	a		b		C	
Metric	Value	95% CI	Value	95% CI	Value	95% CI
OCW	7.289	(5.780, 8.800)	0.688	(0.578, 0.771)	− 7.724	(− 9.501, − 5.947)
OCW (therians only)	7.470	(5.958, 8.981)	0.679	(0.599,0.760)	− 7.939	(− 9.709, − 6.168)
OCW (excluding taxa)	7.253	(5.931, 8.575)	0.694	(0.621, 0.767)	− 7.767	(− 9.324, − 6.209)
OCW (all specimens treated independently)	6.852	(6.160, 7.544)	0.714	(0.671,0.757)	− 7.235	(− 8.039, -6.431)
OCW (average of wild-caught specimens only)	7.694	(5.544, 9.843)	0.663	(0.551, 0.775)	− 8.154	(− 10.619, − 5.690)
CBL	18.017	(8.059, 27.975)	0.435	(0.288, 0.581)	− 26.885	(− 38.366, − 15.403)
HBL	3.747	(2.036, 5.457)	0.918	(0.759, 1.078)	− 11.883	(− 15.170, − 8.596)
OCW versus CBL	1.001	(0.768, 1.234)	1.020	(0.907, 1.134)	1.446	(1.123, 1.770)
OCW versus HBL	2.631	(1.844, 3.418)	0.652	(0.536, 0.768)	0.713	(− 0.199, 1.626)

The second-order term of the log-quadratic model significantly correlated with log body mass (*t* = − 7.384, *p* < 0.001), whereas under a log-cubic model the quadratic term remained significantly correlated (*t* = − 7.376, *p* < 0.001) but the cubic term did not (*t* = 0.424, *p* < 0.672). This suggests that the addition of a quadratic term substantially improved model accuracy, but the addition of a cubic term is not statistically justifiable.

A major difference between the 2/3 power model and the log-quadratic model is the distribution of leverage. In the log-linear and 2/3 power model, leverage is relatively evenly distributed across the data points, although data at the extreme ends of the *x*-axis have more leverage (Additional file 2). By contrast, in the log-quadratic model, most of the points have almost no leverage, with only the points at the extreme ends of the axis influencing the shape of the quadratic curve. It is this reason, along with the fact that the shape of the log-quadratic model is very sensitive to taxon inclusion and data distribution (see below), that a simpler 2/3 power model is preferred here.

**Fig. 4:**
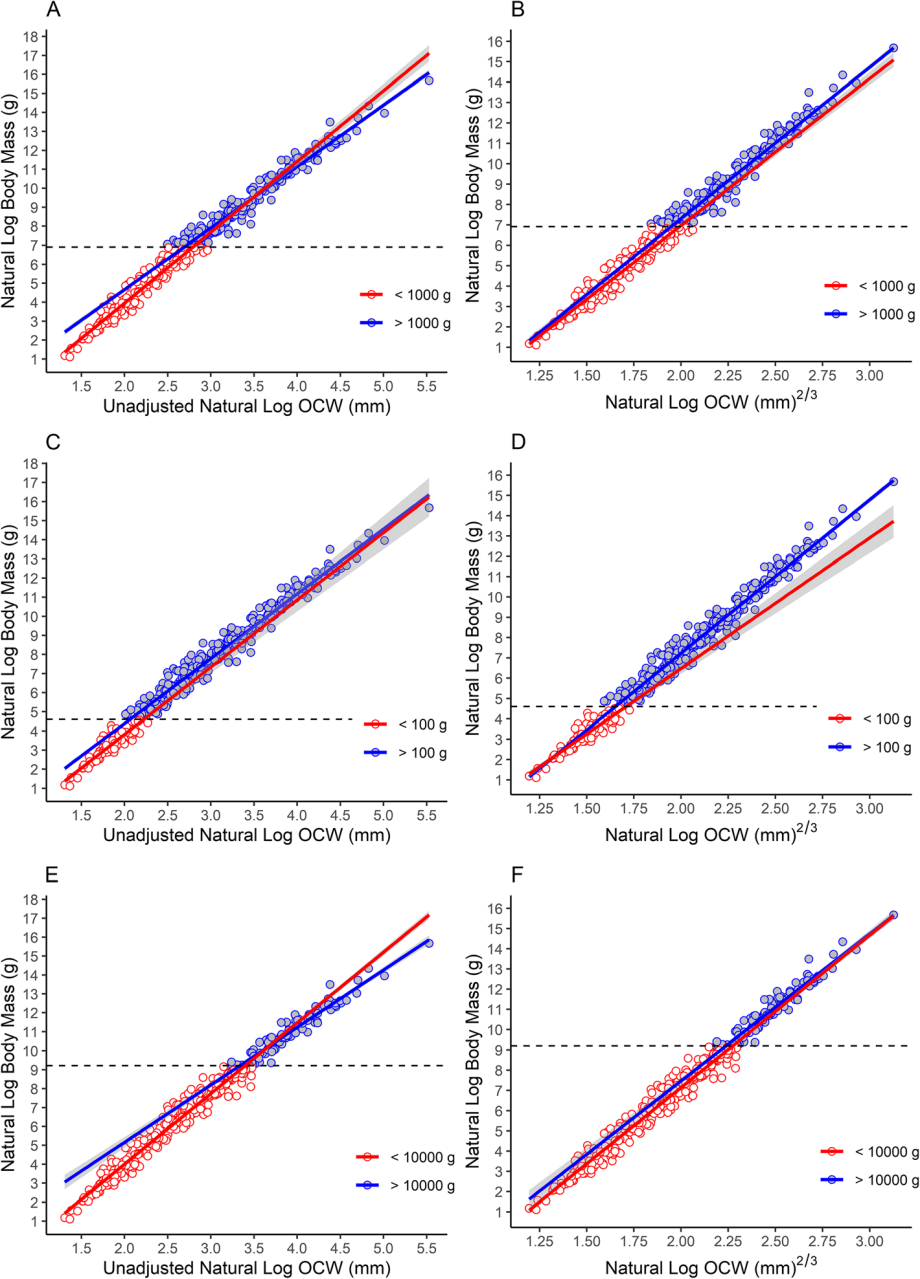
Comparison of scaling patterns for different size classes. **A, C, E** Log-linear scaling relationships; **B**, **D, F** Scaling relationships of the data where log OCW is transformed by raising it to the 2/3 power. **A, B** Scaling patterns for taxa above and below 1 kg. **C, D** Scaling patterns for taxa above and below 100 g. **E, F** Scaling patters for taxa above and below 10 kg

When comparing log-linear regression lines for different size classes (Fig. 4), the slope of the regression line becomes noticeably shallower at larger body sizes, indicating that log OCW increases at a greater rate relative to body size at larger body sizes. These differences in slope are significant when the dataset is divided into all taxa greater than or less than 1 kg (*t* = − 5.568, *p* < 0.001) and 10 kg (*t* = − 6.460, *p* < 0.001), but not at 100 g (*t* = − 0.619, *p* = 0.5360). However, there is no obvious inflection point that would suggest a threshold between different linear scaling models, as suggested by Economos [130], but rather a gradual change in slope. This, again, suggests the relationship between the data is non-linear and that a non-linear 2/3 power or log-quadratic model is more appropriate than a log-linear one.

Transforming log OCW by raising it to the 2/3 power resulted in this pattern of non-linear allometry being linearized. Under a 2/3 power model, when comparing the regression lines formed by all taxa above and below 1 kg finds both slope (*t* = 1.194, *p* = 0.233) and intercept (*t* = − 0.270, *p* = 0.787) to be non-significantly affected by size class (Fig. 4b). The same was true when comparing slope (*t* = − 1.081, *p* = 0.281) and intercept (*t* = − 1.449. *p* = 0.148) for all taxa above or below 10 kg (Fig. 4f). The thresholds for these two bins were slightly lower or higher, respectively, than the midpoint for body mass in the data set (4430 g). Both slope (*t* = 3.083, *p* = 0.002) and intercept (*t* = − 2.643, *p* = 0.008) significantly differed between taxa above and below 100 g (Fig. 4d), but it is possible that this is due to the relatively smaller number of species less than 100 g in the present sample (*N* = 84, 20.8% of the total sample) and the relatively narrow size range spanned by these species compared to the other two size class analyses. Even for taxa above 10 kg, which span a similar number of species (*N* = 96), the log range of body sizes spanned by these taxa was much larger.

### Results of regression between OCW and body mass

**Fig. 5:**
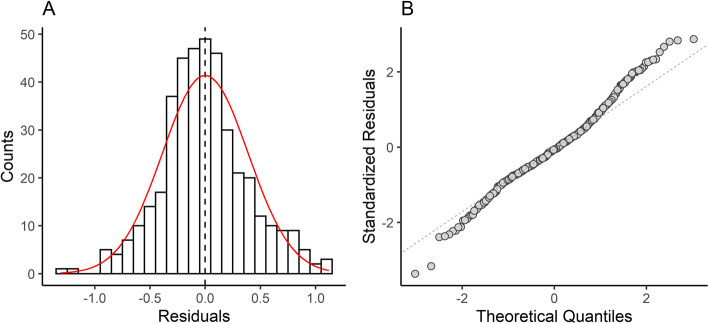
Histogram (**A**) and Q-Q plot (**B**) of the residuals of the total species regression analysis between natural log OCW and natural log body mass, showing the approximately normal distribution of the residuals

**Fig. 6:**
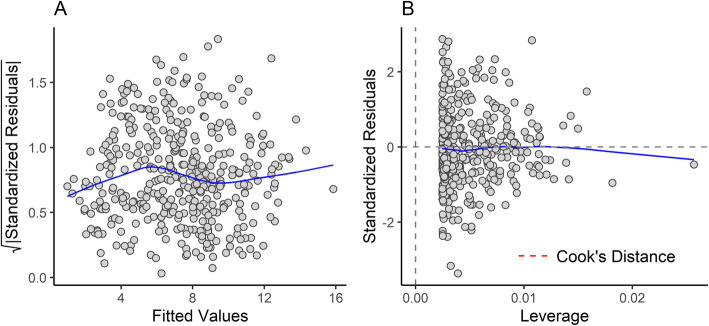
Diagnostic plots of the total species regression between natural log OCW and natural log body mass, showing the scale-location plot (**A**) and the residuals versus leverage (**B**)

The regression equation between OCW and body mass has a percent prediction error (%PE) of 31.09 (Table 4). 41.6% of taxa have an estimated body mass within ± 20% of the actual value, whereas 81.4% of taxa have estimates masses within ± 50% of the actual value. The median error (21.73%) is much lower than the mean error, suggesting that error rates in the regression equation are being inflated by outlier points with high error. This is supported by the distribution of the residuals (Fig. 5a). Residuals of the regression equation are homoscedastic (Breusch-Pagal test for heteroscedasticity; BP = 0.13618; df = 1, *p* = 0.7121), as also indicated by the scale-location plot (Fig. 6a), but have a slight positive skew primarily due to several taxa that exhibit occiput morphology that deviates from the typical mammalian condition (Fig. 5a). Skewness (0.193) is relatively low (≤ |0.5|, [131]), indicating the distribution of the residuals are roughly symmetrical. Excess kurtosis is 0.532, suggesting that the residuals are slightly leptokurtic (i.e., there are more observations closer to the mean than to the tails of the distribution).

**Table 4:** Results of the regressions of natural log OCW (in mm) against natural log body mass (in g)

Dataset	*N*	Equation	*r*^2^_adj_	%PE	CF	%PE_cf_	%SEE
All species	404	ln(body mass) = 7.69289 × ln(OCW)^2/3^ − 8.19502	0.9810	32.03	1.047	31.09	47.69
Therians only	401	ln(body mass) = 7.70727 × ln(OCW)^2/3^ − 8.21527	0.9823	31.55	1.037	30.83	45.92
Excluding taxa	374	ln(body mass) = 7.76568 × ln(OCW)^2/3^ − 8.36414	0.9862	27.89	1.021	27.47	40.62
All species (*N* ≥ 6)	170	ln(body mass) = 7.68862 × ln(OCW)^2/3^ − 8.18776	0.9785	29.77	1.065	28.69	43.99
All species (*N* ≥ 6), excluding taxa	160	ln(body mass) = 7.76999 × ln(OCW)^2/3^ − 8.2391	0.9815	27.72	1.068	26.56	41.30
All species (*N* ≥ 10)	75	ln(body mass) = 7.71479 × ln(OCW)^2/3^ − 8.19504	0.9901	22.57	1.041	22.53	32.76
All species (*N* ≥ 10), excluding taxa	73	ln(body mass) = 7.71406 × ln(OCW)^2/3^ − 8.21147	0.9916	20.71	1.042	20.66	30.14
Species > 1000 g	232	ln(body mass) = 7.4292 × ln(OCW)^2/3^ − 7.5507	0.9526	30.76	1.080	30.04	46.17
Australidelphia	32	ln(body mass) = 8.1213 × ln(OCW)^2/3^ − 8.9050	0.9720	31.30	1.025	30.54	47.44
Ungulates	61	ln(body mass) = 7.6451 × ln(OCW)^2/3^ − 8.0565	0.9619	27.62	1.057	27.47	39.95
Primates	44	ln(body mass) = 8.2761 × ln(OCW)^2/3^ − 9.5675	0.9636	18.75	1.029	18.06	28.41
Rodentia	96	ln(body mass) = 7.8157 × ln(OCW)^2/3^ − 8.2573	0.9710	29.35	1.078	29.19	40.80
Sciuromorpha	29	ln(body mass) = 8.2497 × ln(OCW) ^2/3^ − 9.1570	0.9726	16.63	1.039	16.45	24.37
Carnivora	81	ln(body mass) = 8.5852 × ln(OCW)^2/3^ − 10.2696	0.9750	22.60	1.031	21.96	34.62

Excluded datasets refer to analyses where groups with apomorphic occiput morphology (Monotremata, Cingulata, Dermoptera, Lagomorpha, Caviidae, Dinomyidae, and *Dipodomys*) were removed from the calculation. All abbreviations follow Table 3

The residuals of the data are not normally distributed according to a Shapiro-Wilk test (*W* = 0.98739, *p* = 0.0014). However, this is probably due to the sensitivity of the Shapiro-Wilk test to departures from normality at large sample sizes, where even small departures from normality will result in the sample failing the normality test [132]. Visual inspection of a histogram of the residuals shows the residuals follow a nearly normal distribution (Fig. 5a). Based on the large number of observations (*N* = 404) and the central limit theorem (which states at large sample sizes most bivariate independent distributions are close enough to normality for assumptions of normality to hold), the distribution of the data is close enough to normal to be used for regression [132]. The quantile-quantile plot of the residuals supports a normal distribution of the residuals (Fig. 5b), though there is a slight deviation in the upper quantile due to a longer negative tail. None of the species included in this dataset exhibit a particularly high Cook’s distance in the residuals versus leverage plot, suggesting that none of the species (including those with specialized condyle morphology) significantly influence the regression model on their own (Fig. 6b).

The absolute value of the residuals is significantly correlated with the sample size for each species (*t* = − 2.011, *p* = 0.045). However, the *r*^2^ value of this correlation is very low (0.010) and the slope is close to zero (*m* = − 0.00633). Plotting the absolute value of the residuals versus sample size does shows a general decrease in error as *N* increases (Additional file 4), though this effect is not strong. These results are likely a consequence of the way that sampling works. Drawing from smaller sample sizes of species increases the influence of individual variation on the mean value, but a single sampled individual could by sheer random chance be close to the theoretical mean value for the species. By contrast, larger sample sizes generally “average out” individual deviations from the species average [133, 134]. Hence, there is not a straightforward linear correlation between sample size and the absolute value of the residuals. Low sample sizes do not necessarily produce higher error rates, but higher sample sizes generally reduce error.

On an ordinal scale (treating the four suborders of rodents separately due to their large sample size in the present data and high overall diversity and morphological disparity), residuals are high (> |0.5|, negative values representing overestimates of body mass and positive values underestimates) in Castorimorpha (0.535, primarily driven by *Dipodomys* spp. and Geomyidae), Dermoptera (1.040), Lagomorpha (0.664), Macroscelidea (− 0.533), Monotremata (− 1.157), Paucituberculata (− 0.721), and Scandentia (− 0.560) (see Table 5). Cingulata also exhibits high average residuals (− 0.495), though not greater than − 0.5. Most of these groups exhibited occiput morphology that significantly deviates from the mammalian average with the exception of Macroscelidea, Paucituberculata, and Scandentia. The high residuals in these taxa cannot be attributed to small sample size, as all three are represented by at least three species and most species are represented by six or more specimens each.

**Table 5:** Average residuals and %PE_cf_ by order, along with the number of species sampled for each order

Order	*N*	Mean residual	Mean %PE_cf_	Order	*N*	Mean residual	Mean %PE_cf_
Afrosoricida	3	− 0.009	58.10	Macroscelidea	4	− 0.533	43.74
Anomaluromorpha	4	0.098	26.94	Microbiotheria	1	− 0.317	30.47
Artiodactyla	51	− 0.015	28.85	Monotremata	3	− 1.157	69.54
Carnivora	81	− 0.125	27.63	Myomorpha	35	0.059	25.76
Castorimorpha	10	0.535	68.46	Paucituberculata	3	− 0.721	53.28
Cingulata	6	− 0.495	39.62	Peramelemorphia	3	0.044	19.70
Dasyuromorphia	9	− 0.123	25.18	Perissodactyla	6	0.039	23.01
Dermoptera	1	1.04	170.10	Pholidota	1	0.099	10.40
Didelphimorphia	27	− 0.062	27.39	Pilosa	4	0.141	25.69
Diprotodontia	19	0.264		Primates	44	− 0.108	19.18
Eulipotyphla	23	0.044	18.70	Proboscidea	1	− 0.178	20.09
Hyracoidea	4	0.056	15.59	Scandentia	3	− 0.56	45.32
Hystricomorpha	18	0.277	49.37	Sciuromorpha	29	0.067	18.82
Lagomorpha	10	0.664	91.74	Tubulidentata	1	0.102	5.78

Rodents categorized by suborder (i.e., Myomorpha, Sciuromorpha) due to large sample size (*N* = 95) and high intraordinal %PE seen within this group

A major concern of using species averages as the unit of observation in studies of body mass estimation is that it impedes the ability of the resulting regression models to make predictions about individual organisms. It is often assumed that using species average values will improve the overall accuracy of regression models by removing noise created by individual variation and body condition (e.g., underweight and overweight animals “offsetting” each other), but this may in turn inhibit the ability of such equations to identify intraspecific patterns of body size variation such as sexual dimorphism, growth patterns, clinal variation, or differences in body size across geologic time [133, 135]. This is especially true for fossil mammals, where body mass estimations are often made on single specimens due to small sample sizes rather than species averages. A regression equation calculated using individual specimens as the observational unit, rather than species averages, produces comparable regression accuracies (%PE = 34.43, %PE_cf_ = 32.93, %SEE = 50.60; Table 6) to the all-species regression equation (Table 4). Additionally, the non-linear least squares fit treating all specimens independently producing a similar exponent to the species average equation (Table 3). This suggests that variation in OCW may not just correlate to species average body size, but the body size of the individual organism being measured.

**Table 6:** Regression equations for OCW under PGLS, using all individual specimens, using only wild-caught specimens, and including condyle shape or brain size as additional variables

Analysis	*N*	Equation	df	*r*^2^_adj_	%PE	CF	%PE_cf_	%SEE
PGLS								
Brownian	404	ln(body mass) = 7.933560 × ln(OCW)^2/3^ − 9.095213	402	–	68.88	1.754	31.56	276.00
OU model	404	ln(body mass) = 7.691496 × ln(OCW)^2/3^ − 8.191059	402	–	32.00	1.046	31.08	47.45
Using all specimens individually								
OCW	2127	ln(body mass) = 7.68179 × ln(OCW)^2/3^ − 8.18617	2125	0.9775	34.43	1.071	32.93	50.60
Using species average of wild-caught specimens only								
OCW	346	ln(body mass) = 7.6249 × ln(OCW)^2/3^ − 8.0759	344	0.9764	32.09	1.071	30.91	48.51
Including “monotreme-like” and “rabbit-like” states as additional variables								
OCW	404	ln(body mass) = 0.71517 × rabbit − 1.14562 × monotreme + 7.75844 × ln(OCW)^2/3^ − 8.35284	400	0.9850	28.18	1.025	27.68	41.37
OCW + brain mass								
OCW + brain mass	323	ln(body mass) = 6.96789 × ln(OCW)^2/3^ + 0.12473 × ln(brain mass) − 7.03109	320	0.9807	31.51	1.063	30.48	46.36
OCW	323	ln(body mass) = 7.65044 × ln(OCW)^2/3^ − 8.08812	321	0.9803	31.54	1.050	30.56	46.88

Abbreviations as for Table 3 and “Methods”

Another concern is that differences in sexual dimorphism might influence the accuracy of regression models, which is why some studies have calculated regression equations treating the means for males and females as separate data points [39, 57]. Treating the means of males and females as separate data points in the present study, filtering out individuals in which sex was unknown, resulted in a regression equation very similar to the all-species regression equation (Additional file 2) and differences between males and females were found to be non-significant (*t* = 0.552, *p* = 0.581).

### Regression models considering condyle morphology

**Fig. 7:**
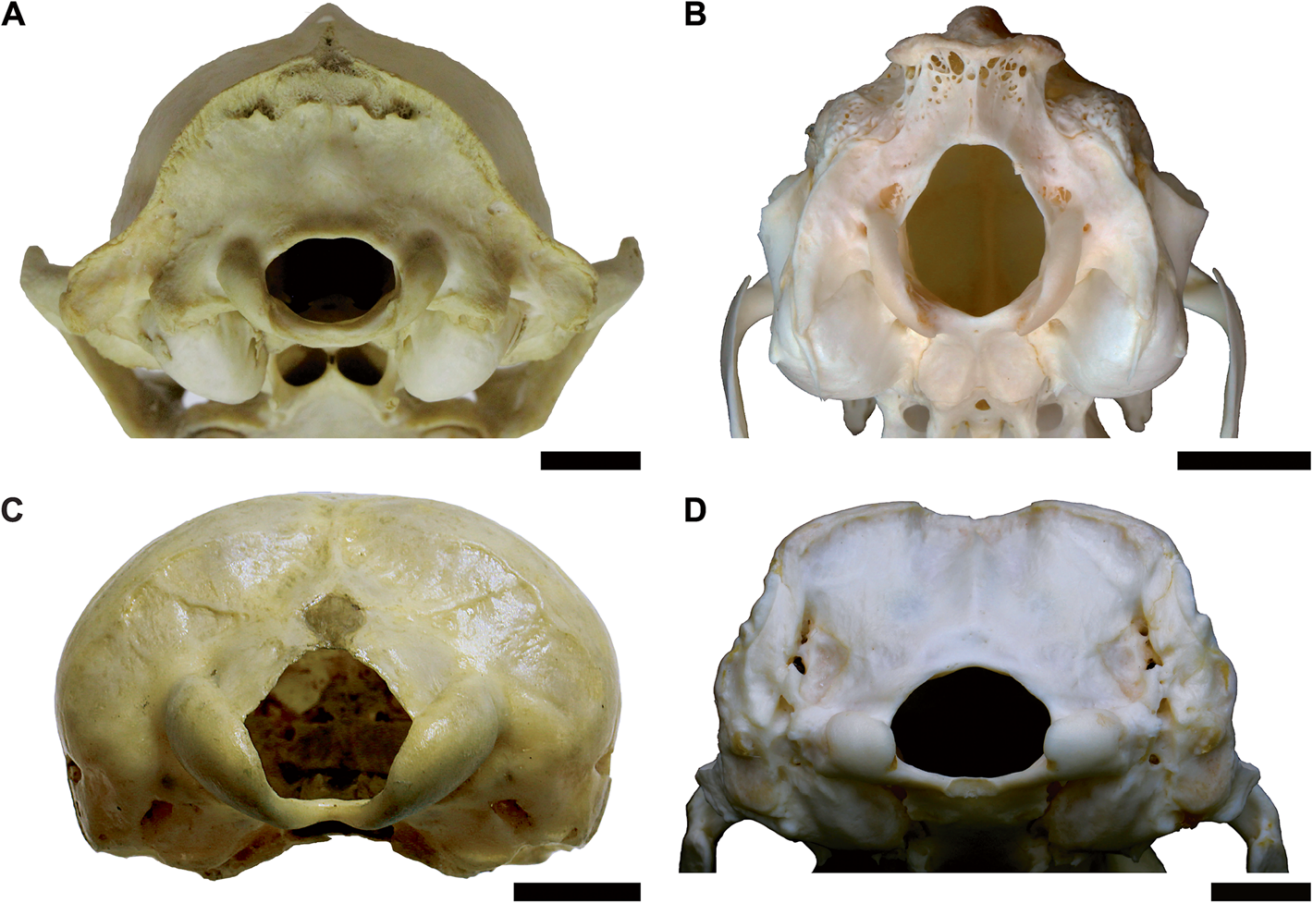
Occipital region of a typical mammal (**A**; *Procyon lotor*, CMNH 22076), contrasting with the distinctive occiput morphology of lagomorphs (**B**; *Lepus* sp., R. Engelman pers. col.), monotremes (**C**; *Tachyglossus aculeata*, CMNH 18877), and cingulates (**D**; *Euphractus sexcinctus*, R. Engelman pers. col.). Scale = 1 cm

Three specialized configurations of the occiput were observed in this study. Most mammals had rounded, reniform condyles that were located directly lateral to the foramen magnum and closely followed the margins of this structure (Fig. 7a). However, several alternate states of occiput morphology could be observed, notably the mediolaterally narrow, pulley-like condyles of lagomorphs and caviids (Fig. 7b), the very wide occipital condyles of monotremes which do not follow the margins of the foramen magnum (Fig. 7c), and the rectangular, laterally projecting condyles of cingulates (Fig. 7d)

**Fig. 8:**
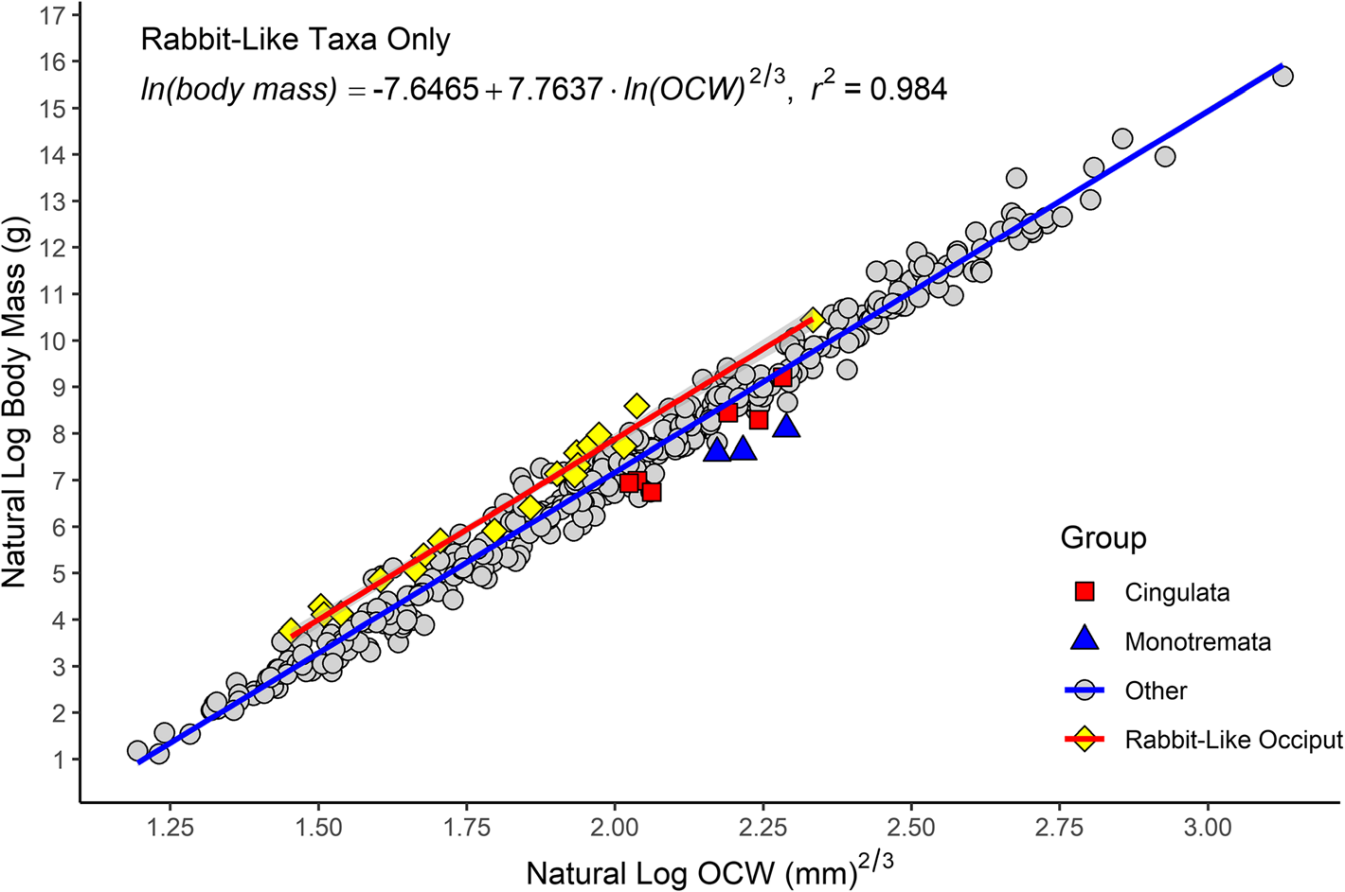
Scatter plot of natural log of OCW raised to the 2/3 power against the natural log of body mass, showing groups that deviate from the main regression line (cingulates, monotremes, and taxa with rabbit-like occiputs) as well as the regression line formed by taxa with rabbit-like occiputs (in red)

With regard to lagomorphs and taxa with lagomorph-like occipital condyles, which were the most heavily sampled group of mammals with a specialized condyle morphology (*N* = 19), a summary of slopes test found that the interaction between slope and the presence of lagomorph-like occipital condyles was non-significant (*t* = 0.050, *p* = 0.960). What this means rabbit-like and non-rabbit-like taxa have near-identical allometries, and the primary difference between these two groups driving the high residuals in taxa with rabbit-like condyles is a shift in the *y*-intercept. This, in turn can be related to the fact that the mediolaterally narrow condyles of lagomorphs and taxa with similar occiput morphology results in a lower OCW relative to other mammals. This observation is further supported by the fact that the slopes of the all-taxon regression line and a regression line calculated based solely on with lagomorph-like occipital condyles are nearly identical (see Tables 4 and 6 and Fig. 8). A regression line could not be calculated for Monotremata as only three monotreme taxa were included in this analysis and all extant monotremes span a very narrow range of body sizes (2–3 kg).

Adding two additional binary categorical variables to the model describing whether a taxon has a “lagomorph-like” or “monotreme-like” occipital morphology results in higher *r*^2^ values and much lower %PE and %SEE (Table 6). The AIC (295), BIC (315), and log likelihood (− 143) for the model considering additional variables for condyle shape are much lower than for any of the models only considering OCW and body mass (compare these values to the ones reported in Table 2). Both the state of having of a monotreme-like occiput morphology (*t* = -5.702, *p* < 0.001) or a lagomorph-like occiput morphology (*t* = 8.720, *p* < 0.001) significantly correlated with body mass when considered as additional independent factor variables in the regression equation.

### Regression models by taxon

Datasets excluding taxa with apomorphic occiput morphology (e.g., Monotremata, Lagomorpha) had lower values of %PE_cf_ and standard error of the estimate (%SEE), with %PE_cf_ < 30% for all analyses (Table 4). Even when excluding these data, the regression line of the log-power model still showed a 2/3 power exponent (Table 3). Calculating the regression line based only on species with large sample sizes also resulted in lower error. However, the low error values for the equations only including species with more than 10 observations may also be due to decreased taxonomic and morphological breadth, as most species in these analyses pertain to a few taxonomic groups (Eulipotyphla, Rodentia, Carnivora) and only eight species in this analysis were larger than 10 kg. The regression equation including only taxa for which body mass was greater than 1000 g produced results that were almost identical to the regression for the entire dataset (Table 4).

Examining the best-fit lines by order found that most species with sample sizes > 5 produced lines with similar allometries to the all species best-fit line, though some groups had different intercept (Additional file 5). Testing for differences in intercept between mammalian orders (or suborders in the case of rodents) found non-significant differences for the majority of clades (*N* = 19, Additional file 2). However, eight clades did show significant differences in intercept: Castorimorpha, Cingulata, Dermoptera, Lagomorpha, Macroscelidea, Monotremata, Paucituberculata, and Scandentia. These clades are all groups which are either characterized by specialized occiput morphology relative to other mammals (Castorimorpha, Cingulata, Dermoptera, Lagomorpha, Monotremata), or otherwise exhibit high residuals as a clade (Macroscelidea, Scandentia, Paucituberculata). Additionally, Macroscelidea, Scandentia, and Paucituberculata exhibit higher *p* values (0.05 > *p* > 0.01) than taxa with extreme occiput specializations (*p* < 0.01).

Examining differences in slope between clades by creating an interaction term between taxonomic group and OCW found that most of the differences between groups were non-significant. When setting Artiodactyla as the reference level (because of the low number of species in the alphabetically first taxon, Afrosoricida), the only groups to have significantly different slopes were Afrosoricida, Carnivora, Dasyuromorphia, Didelphimorphia, and Hystricomorpha. However, the 95% confidence intervals for slopes all strongly overlap with one another and the slope for the all-species regression line except for Afrosoricida, which is composed of a small number of species spanning a narrow range of body sizes (*N* = 3, 140–500 g), and thus this result might be due to sampling error. Notably, the slope of Lagomorpha (which are exclusively composed of species with a specialized occiput morphology) did not differ significantly from the remaining sample, further supporting the idea that residuals in the present equation are driven by differences in occiput shape rather than clade-specific patterns of allometric scaling.

Accuracy of the taxonomically restricted regression equations were higher than those of the total species regression, as would be expected based on previous studies. The taxonomically narrowest dataset, the one including only sciuromorph rodents, produced the lowest error values, suggesting that taxonomic breadth is correlated with overall error rates. However, for the all-rodent regression equation, residuals and %PE_cf_ for rodent taxa that were outliers in the total species regression (i.e., caviids, *Dinomys*, and *Dipodomys*) remain high even when rodents are considered by themselves. The QQ plot and histogram of the residuals of the rodent-only regression also show a strong departure from normality (compare Fig. 5 and Additional file 6), suggesting that all rodents may not conform to a single regression equation (though it is possible this departure from normality could disappear with a larger sample of rodents). Rodentia in general seems to show much higher variation in occiput proportions than most other groups, even after accounting for the high diversity of this clade.

Under a log-quadratic model, the best-fit regression curve was somewhat more variable than the best-fit lines under a log-power model. In particular, the curvature of the best-fit curve was not very well-resolved when trying to predict data beyond the upper and lower bounds of the data (see Additional file 2). This can be seen in the very wide confidence intervals for the best-fit curve beyond the distribution of measured species and the fact that the extrapolated curve for Australidelphia and Primates did not follow the general shape of the data for all mammals. Perhaps the most extreme example of this was the all-Sciuromorph equation, which produced a concave-up curve with an extremely wide confidence interval. This result seems to be the result of several species of *Marmota* spp., which are known to go through extreme annual variation in body mass [136], but in this case the presence of a few species is able to massively influence the shape of the loq-quadratic regression curve. Indeed, for Sciuromorpha, the second-order term did not have a statistical effect (*t* = 1.054, *p* = 0.301).

Binning the data by superorder to increase sample size results in regression curves for the five therian superorders that are roughly comparable to the all-species model. Xenarthra shows slightly more variation than other therians, but this appears to be due to the low diversity within this clade and the presence of Cingulata (which exhibit specialized occiput morphology). When comparing intercepts between superorders, Euarchontoglires (*t* = 2.429, *p* = 0.0156) has a significantly different intercept from other therians, but this result appears to be driven by the inclusion of species with a specialized lagomorph-like occiput (Lagomorpha, Caviidae) as including the presence of a lagomorph-like occiput as an additional explanatory variable reduces the statistical effect of this result (*t* = 1.779, *p* = 0.073).

Overall, the results of the log-quadratic curves in this study agree with the results of Campione [137] and Müller et al. [138], who found that log-quadratic curves were very unpredictable when extrapolated beyond the range of values used to calculate them and the detection of non-linear allometry was heavily dependent on the range of body sizes included in the dataset, respectively.

### Phylogenetic signal and phylogenetic generalized least squares

The residuals of the all-species regression equation show strong phylogenetic signal (mean *λ* = 0.901, *p* < 0.001). However, %PE and %SEE are much higher for under a Brownian model (%PE = 68.88%, %SEE = 276%) than OLS (%PE = 32.03%, %SEE = 47.59) (Table 6). Applying correction factors decreases this disparity (PGLS %PE_cf_, 31.56; OLS %PE_cf_, 31.09), but at the same time, PGLS requires extremely large correction factors (1.754) that require increasing the fitted value by over 75% to produce a more accurate result, which suggests deeper methodological problems that are being obscured by the use of correction factors. The high %SEE is likely due to the fact that PGLS does not remove the effects of phylogeny from the analysis nor adjust the predicted values based on phylogenetic position, rather it merely fits the best-fit line that minimizes the covariance between the residuals of the regression and the underlying phylogenetic correlation matrix [139]. Indeed, PGLS generally results in higher standard errors, weaker correlations between variables, and broader confidence intervals compared to OLS [139].

AIC, BIC, and log likelihood values for PGLS were extremely variable and depended entirely on which of the trees from the random sample were chosen for analysis. Despite all 100 trees producing similar regression lines with a relatively little variation in the coefficients (slope = 7.967 ± 0.088; y intercept = − 9.160 ± 0.179, see Additional file 2), AIC and BIC values formed normal distributions with a range of over 150 and standard deviations of 50 (see Additional file 2), when differences of AIC more than 2 are considered statistically significant [140]. However, the mean and median values for both AIC (mean = 406, median = 396) and BIC (mean = 418, median = 408) were higher than for OLS. This extreme variability in AIC values is noteworthy given that all of the PGLS analyses used the same dataset, the trees were similar enough in topology for each to be considered a reasonable approximation of mammalian phylogeny, and the resulting regression equations were near-identical. The high variability in AIC, BIC, and log likelihood values in potential most parsimonious trees makes it almost impossible to use these statistics to make model selection. Indeed, it is rather concerning that whether or not an OLS model is favored over a PGLS one is entirely driven by relatively minor differences in tree choice. Notably, this variation in AIC and BIC did not correlate with model prediction accuracy nor variation in model coefficients. That is, although PGLS under some trees produced an AIC lower than the OLS model, these models did not produce more accurate results. Excluding one model that produced unusually poor support values, %PE ranged from 62.8 to 78.1% and %SEE ranged from 219.9 to 452.6 across the 100 trees examined, at minimum producing error statistics twice as high as OLS. Because the goal of this study is predictive accuracy, rather than model fit, methods for PGLS as currently utilized are inappropriate here. Notably, the issues highlighted in this study are not driven by the data used, but are broader issues concerning PGLS. As the focus of this paper is on using OCW as a body mass estimator, addressing these issues is beyond the scope of the paper.

**Fig. 9:**
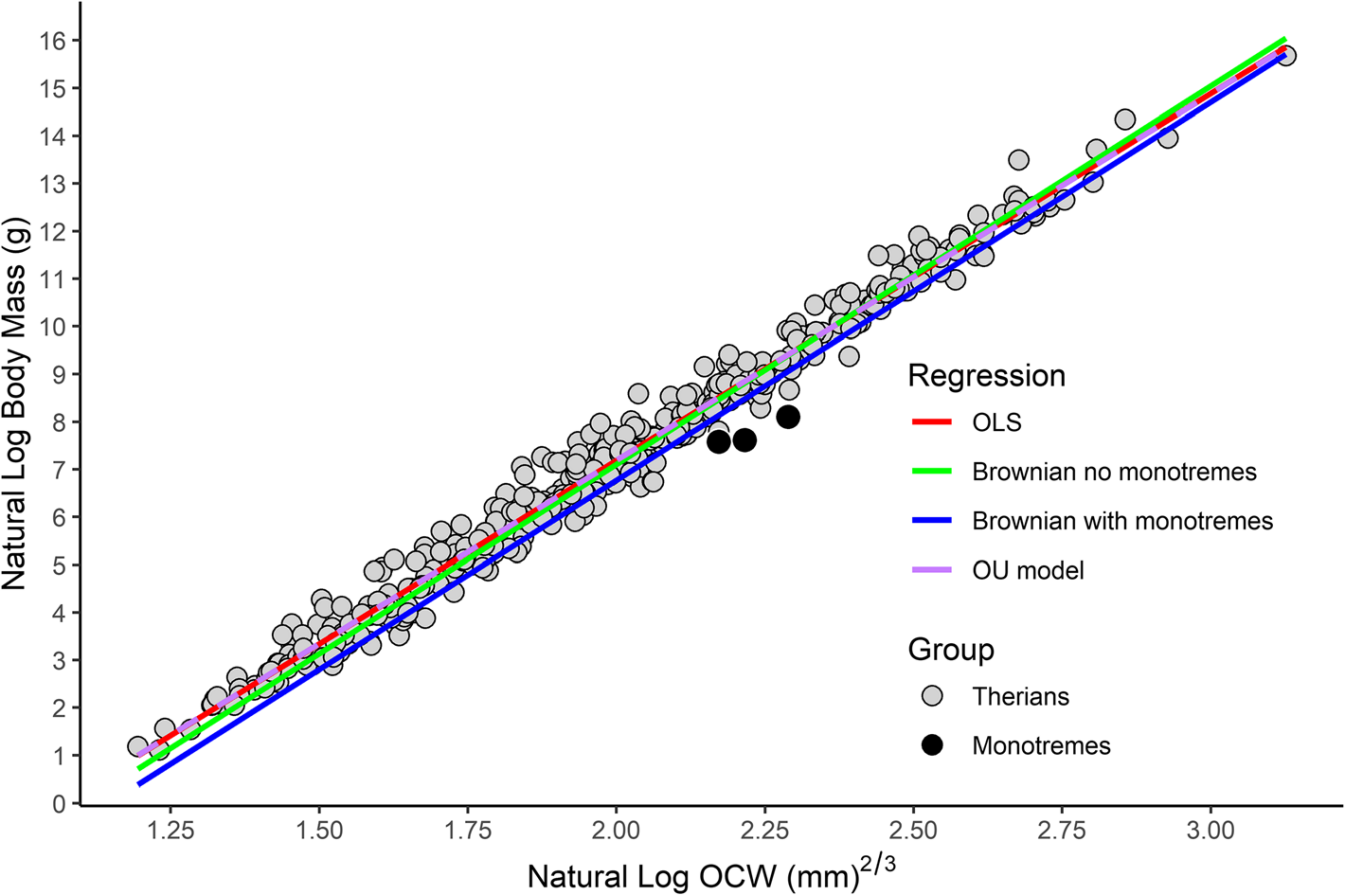
Linear regression between log OCW and log body mass under OLS (in red), PGLS under a Brownian model (in blue), PGLS under a Brownian model excluding monotremes (in green), and PGLS under an OU model (in purple, dashed to not obscure the other lines), showing how the inclusion of monotreme taxa greatly biases the PGLS regression line under a Brownian model due to the deep divergence between Theria and Monotremata

The PGLS model under Brownian motion produced a best-fit line that almost completely bypassed the distribution of the data (Fig. 9). This pattern is almost entirely driven by the apomorphic occiput of Monotremata (see “Discussion”), demonstrated by the fact that omitting monotremes results in a regression line very close to that produced by an OLS or OU model. Even excluding Monotremata phylogenetic signal in the dataset was still very high (mean *λ* = 0.884, *p* < 0.001), the resulting goodness-of-fit and the accuracy of PGLS (%PE = 36.12, %PE_CF_ = 34.30, %SEE = 277) was lower than for the therian-only model in OLS (%PE = 31.55, %PE_CF_ = 30.83, %SEE = 45.9). This is a result of the fact that in the absence of phylogenetic information when predicting new values PGLS defaults to assuming the new taxa are located at the root of the entire tree [141]. This, again, is supported by the results of the current dataset: the best fit line under Brownian motion is roughly halfway between the lines formed by monotremes and therians, whereas the line under an Ornstein-Uhlenbeck (OU) model (which can better account for non-uniform rates of evolution) seemingly identified Monotremata as exhibiting a rate shift compared to other mammals. Phylogenetic information from the evolutionary model must be incorporated back into the model in order to produce accurate predictions, or else it will produce inaccurate results. This is an issue that is known in the specialist literature on phylogenetic comparative methods and has mathematical solutions [141], but is currently not implemented in available PGLS software (i.e., the fitted values reported by available PGLS R packages produce identical values to those manually calculated by treating the PGLS best-fit line as an OLS line). As this is a larger issue with the software presently available to perform PGLS, rather than unique to the present dataset, discussing and addressing this problem is beyond the scope of the present study. It seems likely that a PGLS model that includes signal in its predictions will outperform OLS given that signal apparently is present in the relationship between OCW and body mass, but presently available R packages do not allow for the consideration of signal when making predictions. As a result, the PGLS models produced here should not be used to estimate body mass over OLS.

Fitting a PGLS using an OU model instead of a Brownian model produces much lower error statistics with almost identical values to the OLS model (Table 2), though the AIC, BIC, and log likelihood are still much higher than under OLS. The PGLS under an OU model does not offer a significant improvement in error rates over OLS. The best-fit line produced by the OU model is nearly identical to that produced under OLS (Fig. 9).

The results of a PGLS using a log-quadratic model are very similar to those of a log-power model. The second-order term is found to be significant under both a Brownian (*t* = − 8.528, *p* < 0.001) and an OU model (*t* = − 7.416, *p* < 0.001). The resulting best-fit line under a Brownian model shows significantly greater curvature than the OLS model, regardless of whether or not monotremes are excluded (see Additional file 2). By contrast, the PGLS fit under an OU model and log-quadratic regression equation is nearly identical to that under OLS. As with the log-power model, the Brownian PGLS without monotremes has a positive shift in the *y*-intercept compared to the model including monotremes. Overall, these results suggest that the curvilinear pattern between ln OCW and ln body mass is a real pattern and cannot be attributed to phylogenetic signal or different scaling patterns between clades.

Ancestral state reconstruction suggests that the relationship between OCW and body mass has remained relatively constant within Theria with pronounced shifts in covariance between the two variables occurring at several nodes, rather than covariance changing through random (=Brownian) drift across the entire tree (Additional file 7). The nodes that characterize abrupt shifts in OCW residuals almost always pertain to the most recent common ancestor of clades characterized by unusual occiput morphology. Negative shifts (i.e., OCW is smaller than expected for body size) are observed at the base of the clades Pseudocheiridae, Macropodini, Microtinae, Geomyidae, Dipodomyinae, Caviidae, Lagomorpha, and Camelidae. Positive shifts are observed at the bases of the clades Paucituberculata, Euphractinae, Macroscelidea, Scandentia, and Mustelidae (especially *Mustela* spp.). Monotremata also had strong positive residuals (and indeed, had the highest positive residuals of any clade), though due to its basal position it is not clear if this represents the ancestral state for the group or is another morphological shift. Several of these shifts (namely with Dipodomyinae, Caviidae, Lagomorpha, and Euphractinae) are associated with apomorphic occiput morphology compared to the rest of Theria.

### Effects of captivity status

When fitting a multivariate ordinary least squares (OLS) regression of all individual specimens treating captivity status as an additional independent factor variable, captivity status is found to be significant (*t* = − 3.259, *p* = 0.00114), with specimens from zoological parks exhibiting slightly higher body mass. However, this difference is slight when examining the distribution of residuals in the two groups via a box plot (Additional file 8). Captivity status is non-randomly distributed with respect to order or body size in the dataset (Additional file 8), due to most data from zoological parks pertaining to megafauna (and vice versa, due to the logistic difficulty in obtaining body masses for wild megafauna). For example, 29.3% of all artiodactyl specimens (51/174), 12.7% of all carnivoran specimens (55/432), and 71.4% of all perissodactyl specimens (5/7) in this analysis came from zoological collections. By contrast, only 1.7% of rodents (11/636), 0.0% of didelphimorphian (0/168), and 0.6% of all eulipotyphlan specimens (1/180) came from captivity. Due to this, captivity status is strongly correlated with body size (*t* = − 13.54, *p* < 0.001). Hence, what appears to be a straightforward relationship between captivity status and the residuals could be confounded by the non-randomness of the data with respect to phylogeny.

The results of the phylogenetic generalized linear mixed model including both phylogenetic signal and captivity status found captivity status to be non-significant (0.325). Omitting all captive specimens and recalculating the regression equation using the species averages for all wild-caught specimens produced a slope (7.289) and exponent (0.663) comparable to that of the all-taxon species average regression equation. The values of this model were within the 95% confidence interval of the OLS model and the resulting regression line was not significantly different from the regression line using the average values for all specimens (ANOVA, F = 0.791, *p* = 0.863).

### Covariance with brain size

Plotting the residuals of the regression equation against relative brain size (measured as the residuals of a regression between brain mass and body mass) found the relationship between the two to be significant (*p* < 0.001) with a negative slope, but with a low correlation coefficient (*r*^2^_adj_ = 0.17; Additional file 9). Brain size significantly correlated with body mass (*p* = 0.009) when treated as an additional independent variable but produced almost no change in model accuracy (Table 6). Comparing the residuals of the regression with brain mass as an additional independent variable against the equation where brain mass is not considered (Additional file 10) finds the residuals of the two regression equations to be highly correlated (*r*^2^ = 0.979, *t* = 121.3, *p* < 0.001) and the slope of this regression line is equal to 1, suggesting that adding brain mass as an additional variable does not significantly reduce the residual variance in the regression between OCW and body mass. By contrast, if brain size significantly improved the regression model, it would be expected that the slope of the plot between the residuals of the two equations would be lower (due to residuals in the regression equation with brain size being lower residuals than the one without brain size), as well as exhibit a poorer fit on the extreme ends of the equation (due to the regression equation with brain size producing more accurate results and lower residuals). If natural log brain mass was included as an additional quantitative predictor variable, mass estimates from this model only differed from the model where brain mass was not considered by approximately 4.3%. Ultimately, brain size had less of an effect on predicted body mass differences in occiput shape (i.e., excluding species with apomorphic occiput morphology or adding additional categorical variables to describe occiput shape, see Table 6).

### Comparisons with skull length and head-body length

As with OCW, the relationship between skull length (as condylobasal length) and body size was not log-linear, but instead showed non-linear allometry. Specifically, larger mammals had disproportionately larger skulls relative to their body size compared to smaller mammals (Additional file 1). Comparing several models, the best-fitting model was a power model where ln condylobasal length was transformed by raising it to the ½ power before regressing it against ln body mass (Additional file 2) and had better accuracy (%PE_cf_ = 35.93, AIC = 544) than if skull length was assumed to scale with geometric similarity (%PE_cf_ = 42.49, AIC = 597). A log-quadratic model had the second-lowest AIC and BIC values (Additional file 2) and the second-order term in this model was found to be statistically significant (*t* = − 7.604, *p* < 0.001).

A residuals versus fitted plot where skull length is not raised to the 1/2 power reveals a distinctly non-linear pattern (Fig. 3c), which is supported by a Breusch-Pagel test of the function (BP = 9.0425, df = 1, *p* = 0.003). This indicates it is not appropriate to treat the relationship between skull length and body mass as isometric. After raising skull length to the 1/2 power, the residuals are more linear (Fig. 3d) but not completely homoscedastic (Breush-Pagan test; BP = 8.0292, df = 1 *p* = 0.004). However, the scale-location plot shows little obvious signs of heteroskedasticity (see Additional file 2) and the data is much closer to homoscedasticity than the model where log skull length is not raised to the 1/2 power. Examination of the data (see Additional file 2) suggests that the heteroskedasticity of the transformed dataset is driven by a few taxa such as the giant anteater (*Myrmecophaga tridactyla*) which have a very long skull relative to body size, or by taxa with fewer observations (which tend to be larger species), rather than by a significant heteroskedastic pattern across the entire dataset as in the untransformed variable. The regression equation between skull length (measured as condylobasal length) and body mass produces an equation with a %PE_cf_ of 35.93% and an SEE of 60.32% (Table 7), which is much higher than the same values for the same taxa under OCW. Even if excluding primates, which are characterized by a short rostrum and thus may not be comparable to other mammals, OCW still outperformed skull length (Table 7).

**Table 7:** Body mass regression equations using condylobasal length (CBL) and head-body length (HBL)

Analysis	*N*	Equation	df	*r*^2^_adj_	%PE	CF	%PE_cf_	%SEE
Condylobasal length (CBL)								
CBL	404	ln(body mass) = 14.2241 × ln(CBL)^1/2^ − 22.4349	402	0.9722	39.96	1.182	35.93	60.32
CBL no primates	360	ln(body mass) = 14.1665 × ln(CBL)^1/2^ − 22.3646	358	0.9753	38.80	1.211	35.06	58.65
HBL								
HBL	404	ln(body mass) = 2.9749 × ln(HBL) − 10.3386	402	0.9775	35.79	1.195	34.38	52.91
OCW versus CBL and HBL								
OCW versus CBL	404	ln(body mass) = 1.04366 × ln(OCW) − 1.38825	402	0.9654	12.04	1.012	12.01	16.55
OCW versus HBL	404	ln(body mass) = 2.53378 × ln(OCW)^2/3^ − 0.82550	402	0.9634	13.92	1.015	13.77	19.70
OCW + CBL + HBL								
OCW + CBL + HBL	404	ln(body mass) = 3.8162 × ln(OCW)^2/3^ + 1.1223 × ln(HBL) + 1.9521 × ln(CBL)^1/2^ − 11.1212	402	0.9889	24.41	1.098	23.34	34.95

Abbreviations as for Table 3 and “Methods”. Condylobasal length approximately equivalent to skull length in this analysis

In contrast to OCW and skull length, HBL scaled isometrically with body mass (Table 7). The regression equation of head-body length (HBL) versus body mass produces an equation with a %PE of 35.79% (PE_cf_ = 34.38) and SEE of 52.91%. Again, this is much higher than the same accuracy statistics under OCW.

Error values when regressing OCW against skull length or HBL were much lower than that produced when any of these variables were regressed against body mass (Table 4). This suggests that these three linear metrics are all closely approximating a similar measure of overall size, but that much of the residual variation in body mass regression equations is being driven by factors that are not being captured by linear skeletal measurements. Residuals for the regression between HBL and OCW were high (>|0.2|) in Dermoptera (0.329), Lagomorpha (0.261), and Monotremata (− 0.448), as well as Cingulata (− 0.192) to a lesser degree. This highlights how even under a different proxy for body size these animals had occipital condyles that were much narrower (Dermoptera, Lagomorpha) or wider (Cingulata, Monotremata) than would be predicted for a mammal of their size.

The covariance of the residuals of the regression for OCW and skull length (0.054), OCW and HBL (0.022), and HBL and skull length (0.091) are all very low. The residuals of OCW are significantly correlated with the residuals of both HBL (*t* = 2.638, *p* < 0.001) and skull length (*t* = 6.146, *p* < 0.001), but the *r*^2^ value for these regressions is extremely low (OCW versus HBL, *r*^2^ = 0.02; OCW versus skull length, *r*^2^ = 0.09). Plotting the residuals of the body mass regression equations for skull length and HBL (Additional file 11) against the residuals for OCW does not produce a strong pattern of correlation. Indeed, the covariance between HBL and skull length and the correlation coefficient between these two variables (*r*^2^ = 0.21) is higher than that for OCW and either HBL or skull length, which may be due to the non-independence of HBL and skull length due to skull length contributing to HBL. The low covariance between the residuals of OCW and skull length suggests that OCW is not strongly influenced by relative head size, in contrast to skull length.

Finally, a multivariate regression equation was performed considering all three variables together as independent estimators. OCW, skull length, and HBL all significantly correlated with body mass as singular variables (*p* < 0.001 in all cases), so the question remained whether error would be reduced if all three were considered together. In the multivariate regression equation all three variables were significantly correlated with body mass (*p* < 0.001), but skull length was less correlated with body mass (F = 3.774, *p* = 1.85 × 10^−4^) than OCW (F = 15.632, *p* < 2 × 10^−16^) or HBL (F = 10.812, *p* < 2 × 10^−16^). Both %PE (24.41, PE_cf_ = 23.34) and %SEE (34.95) were much lower for the multivariate equation than for any of the univariate equations (Table 7). Notably, even when three extremely strong predictors of body mass were used together, it was still not possible to reduce percent estimation error to below 20%.

## Discussion

### Allometry of OCW

The data here show that the scaling relationship between OCW and body mass is not log-linear, but instead exhibits non-linear allometry (and the same is true of skull length). There are four lines of statistical evidence which suggest that this non-linear relationship is not a statistical artifact. First, model support statistics such as *r*^2^, %PE_cf_, log likelihood, AIC, and BIC are substantially better for non-linear models (primarily a 2/3 power or log-quadratic model for OCW) than a linear one (Table 2). Second, this pattern remains even under PGLS (Table 2) and is present in multiple clades of similar size, indicating that it cannot be attributed to phylogenetic signal within a particular clade biasing the regression model (e.g., as discussed in [142]). Third, the residuals versus fits plot of these data under a log-linear model indicates significant non-linearity, which is normalized under a non-linear model (Fig. 3). Fourth, the slope under models with log-linear data is slightly different between size classes, with the slope of the larger taxa being slightly lower (Fig. 4), which is what would be expected if the data scaled sublinearly after log-transformation.

In general, regressions of biological variables have been performed with the assumption that log-transformation sufficiently linearizes the data for further analysis. However, a number of studies have found that several biological variables of interest, including brain size [143], mammalian basal metabolic rate [138], limb bone dimensions [137, 144, 145], and (in the present study) skull length and OCW, retain significant curvilinearity even after log-transformation (i.e., non-linear allometry, sensu Knell [146]). Previous studies have tried to account for non-linear allometry by proposing differential allometry within distinct size classes [130, 145] or different clades [145]. However, this approach appears to be unjustifiable. When examining the distributions of the data (see OCW and skull size in this study and limb bone measurements in Bertram and Biewener [145]), there are no sharp changes in slope that might represent logical thresholds at which different scaling models might be applicable between size classes (e.g., the 20 kg threshold proposed by Economos [130]). Instead, there appears to be a gradual change in slope across the sample as a whole. Similarly, this curvilinear relationship does not appear to be attributable to clade-specific allometry patterns. For example, the limb bones of similar-sized bovids and carnivorans exhibit the same pattern of allometric scaling [137: Fig. 1]. This can also be seen in the present study where even under PGLS the relationship between OCW and body mass is curvilinear, indicating that the non-linear allometric pattern is not driven by a single clade. Instead, at least in OCW, variation in phylogeny or natural history seems to be primarily reflected in the intercept.

Other studies have attempted to model non-linear allometry by adding a second-order (quadratic) term to the model, but there are some difficulties with this approach. For one, log-quadratic and other models of non-linear allometry have been less studied than log-linear ones, and no real biological justifications have been proposed as to how to model this relationship. In a log-linear equation, the slope of the line is converted to a power rule when the antilog is taken [29], which can then be compared to various theoretical models which have known coefficients (e.g., the square-cube law, elastic similarity). This cannot easily be done with a non-log-linear model, particularly with the second-order term [147]. Similarly, while some studies have proposed possible reasons as to why non-linear allometry might occur (e.g., greater stresses on the skeleton at larger sizes [148];), they do not provide any biomechanical reasoning as to why this relationship is best modelled by a log-quadratic model versus a log-exponential or log-power one beyond the log-transformed data having a non-linear distribution.

Another issue is that log-quadratic models can be very sensitive to data distribution and taxon selection. Müller et al. [138] found that reliably identifying whether a log-linear and log-quadratic model more appropriately fit a set of biological data was heavily dependent on the range of body sizes included in the dataset, with those data that comprised a narrow range of body sizes often failing to detect non-linearity [138]. Additionally, quadratic models are very sensitive to the distribution of values at the extremes of the dataset, and this can produce unreliable predictions at their extreme ends or if extrapolating, as noted by Campione [137]. This is seen in the present study where the curvature of various groups can be strongly influenced by a few points and subsets of the data with smaller ranges of body sizes are often unable to identify non-linearity. Yet ignoring non-linear allometry and assuming log-linear models approximate the true relationship may not be possible due to producing systematic error in the estimate, especially if these methods are intended to be applied to extinct megafauna (which, at least among paleobiologists, is often the end goal of such analyses [58, 74, 121, 137, 149]). A good example of this can be seen in the present dataset with *Loxodonta africana*, in which a log-linear model utterly fails to accurately predict body mass.

One possible solution might be modelled as the following. Consider for a moment some linear skeletal measurement represented by *L*, and body size (represented by body mass, or *BM*). The traditional way allometric equations work is to linearize the distribution of the data by taking the log of both sides, resulting in:

$$ \ln L=m\ast \ln BM+b $$

in which *m* is the slope and *b* is the intercept. As a result, when the antilog is taken, the slope is converted to an exponent, resulting in:

$$ L={BM}^m+\exp (b) $$

Usually, in these equations body mass is treated as the independent variable, because the primary focus of these studies is determining how the biological variable of interest scales with respect to body size. However, because in this case, the dependent variable of interest is body mass the equation can be modelled as:

$$ \ln BM=m\times \ln L+b $$

such that when the equation is converted back to an arithmetic scale the result is:

$$ BM={L}^m+\exp (b) $$

The general assumption made in most allometric studies is that the exponent *m* is constant across all body sizes. However, this is not the case. Weight-bearing elements of very small mammals (rodents and small carnivorans such as mustelids) generally scale close to geometric similarity (L ∝ BM^0.33^), larger mammals such as large carnivorans (large felids, ursids) and most bovids scale according to elastic similarity (L ∝ BM^0.25^), and the very largest mammals (ceratomorphs, large bovids) scale according to stress similarity (L ∝ BM^0.125^) [145, 148, 150, 151]. This is likely a multiplicative effect due to the increasing stress placed on skeletal structures (e.g., articular surfaces, limb bone circumferences) at larger body masses. This means that the exponent in the model is not a constant integer across taxa but instead is proportional to some constant *Z* such that:

$$ L={BM}^{m\times Z} $$

or, rewriting the equation such that body mass is the dependent variable results in…

$$ BM={L}^{m\times Z} $$

*Z*, in turn, is proportional to log body size (*Z* ∝ ln *BM*). However, body mass (the dependent variable) cannot be used as a variable to calculate itself. However, assuming that the variable *L* scales with isometry outside of this upscaling factor (i.e., the majority of the magnitude in values of *L* correlate with size), then *Z* ∝ ln *BM* ∝ ln *L*.

Similarly, the distribution of the data and differences in exponent across scaling models suggests that simply adding a second order (quadratic term) is not appropriate, as the δ*m* is not constant but increases at greater values of *x*. This is more consistent with a power rule, with the value of *Z* being close to zero at small body sizes (resulting in geometric similarity) but becomes increasingly influential at larger body sizes. Therefore, *Z* ∝ ln *L*^*r*^, where *r* is some constant. This results in the allometric model being written as:

$$ \ln BM=m\times \ln {L}^r\ast \ln L+b $$

which, due to the rules of multiplying exponents with the same base, can be rewritten as

$$ \ln BM=m\times \ln {L}^{1+r}+b $$

The constant *r* must then be solved for experimentally. In the present study the best-fit relationship is one of ln(BM) ∝ ln(OCW)^2/3^, which suggests that *r* has a value of − 1/3. Thus, because OCW scales positively with respect to body size, it is necessary to downweight OCW at larger values in order to produce reliable estimates of body mass.

The slope of the regression line not considering the scaling factor is roughly OCW ∝ BM^0.130^. This is close to the exponent predicted by stress similarity, which might be expected given that the primary role of the occipital condyles is the stabilization of the occipito-atlantal joint, and scaling under elastic similarity for stresses produced by bending and torsion is predicted to L ∝ BM^0.125^ [148]. However, this should be treated with some caution as it is not clear how adding in a scaling factor affects predictions of model shape. The log-linear regression model suggests that in a broad sense OCW ∝ BM^0.277^, which might imply scaling according to elastic similarity (L ∝ BM^0.25^, [148]), but again making comparisons between a linear and non-linear allometric model is difficult.

There are several possible biological explanations for this non-linear allometry in OCW. One is that the non-linear relationship between ln OCW and ln body size is correlated to the non-linear pattern for relationship between skull length and body size described here. If maintenance of a functional occipito-atlantal joint is a major selective factor on the dimensions of the occiput and if larger animals have proportionally larger heads, then it might be expected that larger animals might require proportionally larger condyles to support the weight of their heads. This would agree with previous observations that articular dimensions tend to scale with positively allometry [152, 153], and that in this study larger animals tend to have larger condyles relative to their body size. However, this would not explain why several animals with very large heads relative to their body size (e.g., the “creodonts” and sparassodonts mentioned in the “Background”) have small occiputs relative to skull size, and the fact that the residuals for the regressions between OCW and body size and skull length and body size are not strongly correlated.

Another possibility is the non-linear allometry in occipital condyle dimensions is part of a broader phenomenon that occurs across articular dimensions more generally, given that non-linear allometry has been most frequently documented in reference to tetrapod weight-bearing structures [137, 144, 145, 148, 151]. Non-linear allometry in limb elements follows the same pattern as documented here for OCW: the rate of increase in skeletal measurements is greater at larger body sizes than smaller ones [148]. Bertram and Biewener [145] and Biewener [154] suggested that non-linear allometry in mammalian limb bones was related to limb posture: at smaller sizes mammals compensate for increased stress on weight-bearing structures by adopting an increasingly erect limb posture, whereas at larger sizes the limbs are virtually columnar and the only functional solution is to dramatically increase limb bone thickness. However, the fact that this pattern also occurs in OCW, which is an axial articular surface and thus not affected by changes in limb posture, as well as skull length, suggests this phenomenon may be more broadly applicable across the skeleton.

Despite exhibiting non-linear allometry, the correlation between ln OCW and ln body mass is extremely strong once non-linear scaling is accounted for. A 2/3 power model is also robust to differences between size classes and phylogeny, given it occurs even under PGLS and most clades exhibit similar slopes under the 2/3 power model. That is, the non-linear allometry of the best-fit model is not driven by one very large or very small clade exhibiting differential allometry relative to other taxa but occurs across all clades. Because the goal of this study is to use OCW to predict body mass, this empirically determined model seems reasonably practical for further use even though the biological mechanisms that produce non-linear allometry across skeletal dimensions more generally are poorly understood.

On a similar note, one of the more noteworthy findings of this study is that skull length does not scale isometrically to body mass but also exhibits non-linear allometry, with ln body mass scaling to ln condylobasal length raised to the 1/2 power. The 95% confidence interval for the empirically fitted curve rules out the possibility of skull length scaling isometrically to body mass (Table 3), the plot of the residuals versus fitted values (Fig. 3) suggests the relationship cannot be modelled linearly, and a log-quadratic model finds the second-order term to be significant. This result is rather concerning given the large number of studies have used skull length to estimate body mass in fossil mammals [39, 64, 149, 155], all of which have assumed either explicitly or implicitly that this measurement scales log-linearly with body size. This pattern may occur for the same reasons as non-linear allometry in limb bone measurements and OCW, but another possibility is that it is due to craniofacial evolutionary allometry (CREA [156, 157];). This result shows that non-linear allometry is widely distributed in mammalian skeletal measurements and that the influence of this phenomenon may have been underestimated on previous studies of mammalian biology.

### Utility of OCW in body mass estimation

OCW is a good predictor of body mass in mammals, with a %PE of about 31% (~ 27.5% if outlier taxa with apomorphic occiput morphology are excluded). This agrees with some previous studies [117, 119, 120] which found OCW to strongly correlate with body mass. A percent error of 31% by itself may seem high, but it must be kept in mind that much of this error arises from small differences between predicted and actual values on a logarithmic scale being magnified when back-transformed to an arithmetic scale (%PE for log-transformed values is only ± 5%, compared to ± 31% for detransformed ones). In general, regression models with errors of less than 33% are considered “good” in body mass estimation, particularly if not restricting comparisons to taxonomically narrow datasets [39, 57, 58, 64].

Despite the great phylogenetic breadth of the present sample, OCW actually produces lower %PE and %SEE many regression equations of previous studies based on more restrictive taxonomic groups (Table 8). These include all of the total species regression equations for carnivorans in Van Valkenburgh [39], most of the total species craniodental regression equations for ungulates in Janis [64], all but two of the equations for Australian marsupials in Myers [158], all but one of the cranial or postcranial equations produced by Aiello and Wood [160], and all but one of the regression equations based on linear measurements of the astragalus calculated by Tsubamoto [58]. Compared to the limb bone equations of Campione and Evans [87] (subsetted to only include mammals in order to allow for direct comparisons), in which the authors found limb bone dimensions to be highly correlated with body mass across tetrapods, OCW outperformed both humeral length and femoral length and produced values comparable to femoral circumference. If occiput shape is controlled for, humeral circumference and OCW produce similar accuracy rates. Similarly, within the present study OCW performs much better than skull length in estimating body mass and produces results comparable to that achieved through the regression of HBL on the same sample. The high accuracy of OCW despite the wide phylogenetic breadth of the present sample is especially notable given that accuracy and taxonomic breath are often inversely correlated in regression equations of body mass [134].

**Table 8:** Accuracy of OCW-based regressions compared to those of other studies

Taxonomic scope	OCW				Other regression equations					
	*N*	*r*^2^	%PE	%SEE	*N*	*r*^2^	%PE	%SEE	Variable	Ref.
All Therians	401	0.982	31.55	45.92	80*	0.985	28.83	41.98	Astragalus (Li1)	[58]
					80*	0.980	34.01	49.68	Astragalus (Li7)	[58]
					80*	0.980	37.17	55.53	Astragalus (Li4)	[58]
All Therians (controlling for shape)	404	0.985	28.18	41.37	69*	0.979	32.36	47.21	Calcaneus (CA2)	[93]
					69*	0.973	38.67	55.46	Calcaneus (CA5)	[93]
					69*	0.973	38.92	55.42	Calcaneus (CA10)	[93]
					187	0.955	50.60	84.78	Humerus length	[87]
					200	0.982	27.73	47.86	Humerus circum.	[87]
					188	0.944	66.77	98.01	Femur length	[87]
					200	0.983	31.16	45.93	Femur circum.	[87]
Australidelphian Marsupials	32	0.972	30.54	47.44	38	0.980	27	39	Total jaw length	[158]
					38	0.979	28	40	CBL	[158]
					38	0.967	35	51	M1-4 length	[158]
Carnivora + Large Dasyuromorphia	86	0.973	23.59	34.74	72	0.96	36	53	HBL	[39]
					72	0.95	47	66	CBL	[39]
					72	0.95	42	61	Occiput-orbit length	[39]
Ungulates (except proboscideans)	61	0.962	27.47	39.95	91	0.975	31.42	45.54	HBL	[66]
					94	0.967	34.51	53.02	p4-m3 length	[66]
					94	0.963	36.34	55.82	m1-3 length	[66]
Rodents	96	0.971	29.35	40.80	36	0.940	30.65	15.70	CBL	[31]
					36	0.890	43.36	21.50	P4-M3 length	[31]
					34	0.890	44.62	21.80	m1 length	[31]
Rodents†	58	0.953	10.21	13.50	36	0.880	39.42	21.70	Humerus diameter	[31]
					203*	0.964	29.55	43.33	CBL	[155]
					93	0.948	34.55	50.14	CBL	[155]
					75	0.920	40.10	64.45	p4-m3 length	[159]
Anthropoid primates	43	0.965	18.49	28.41	46	0.98	24	–	Biorbital breadth	[160]
					46	0.97	25	–	Foramen magnum area	[160]
					38	0.98	18	–	Humeral length	[160]
					38	0.99	20	–	Anteroposterior diameter of femoral head	[160]

Only linear variables are considered here as models with multiple independent anatomical variables tend to produce lower errors regardless of what measurements are considered [58, 86, 159]. When large numbers of regression equations were calculated in a given study, only the equations with the lowest %PE were used for comparison. %PE was reported for this study instead of %PE_cf_ as many of the studies considered here did not use correction factors. * = regression equation calculated using individual specimens, instead of species averages. † = results of Moncunill-Solé et al. [119]

OCW has several advantages over other regression equations (Table 9). OCW is relatively easy to measure and can be unambiguously recognized by different observers. Previous studies have found that OCW is an extremely replicable measurement, at least among features of the occiput, and shows low inter-observer bias [163]. Furthermore, because OCW is a cranial measurement, it does not require associated postcrania to estimate mass (as is the case for limb bones) or a relatively complete specimen with undistorted skull and spinal column (as is the case for HBL). This allows it to be applied to a wider range of specimens, including taxa known only from the skull (e.g., *Andrewsarchus* [164], *Josephoartigasia* [149]). Additionally, because OCW scales with the size of the postcranium and is relatively consistent across Theria, it avoids many of the common pitfalls of estimating body mass based on craniodental measurements such as taxa having a disproportionately large skull [39] or teeth and limbs that are not comparable across taxa [64, 66]. Indeed, in the present dataset, OCW even accurately estimates body mass in many taxa which have skulls and teeth that cannot easily be compared to other mammals such as anteaters, sloths, and aardvarks (%PE_cf_ for *Orycteropus afer* = 5.78%, see below for discussion of %PE_cf_ in Pilosa).

**Table 9:** Pros and cons of various methods of body mass estimation discussed in this paper

Variable	Pros	Cons
Pros and cons of various methods of body mass estimation discussed in this paper		
Dental variables (e.g., molar row length)	1. Abundant in the fossil record and known for almost every fossil mammalian taxon 2. Easy to obtain large sample sizes 3. Dimensions of permanent teeth in mammals always reflect adult size rather than growth stage due to diphyodonty	1. Extremely morphologically variable and often cannot be applied across taxa (e.g., edentulous taxa) 2. Little reason to believe they necessarily correlate better with mass than other variables 3. Cannot be used to reconstruct growth patterns in fossil mammals 4. May be biased by head size
Cranial variables (e.g., skull length)	1. Does not require associated postcrania 2. Slightly less biased by dietary habits than dental variables	1. Can be highly influenced by ecology and non-isometric allometry (e.g., rostrum length) 2. Often fails to produce accurate estimates if head is disproportionately large/small relative to body size
OCW	1. Highly conserved across Mammalia 2. Strong theoretical reasons to believe OCW correlates with spine size, and therefore body size 3. Unlike other craniodental variables is not biased by skull size 4. Only requires skull rather than partial skeleton	1. Not as well studied, so potential confounding factors and interspecific variation less understood 2. Sample sizes can be small because skulls may have damaged occiputs 3. Potential concerns with covariation with brain size
Head-body length	1. Probably the strongest theoretical reasoning as to why this variable should most closely approximate mass [66, 161] 2. Extremely large sample of published extant comparative data with associated mass	1. Requires rare, nearly-complete specimens, much less tolerant of missing data than any other method 2. Head-body length highly subject to subjective decisions and almost always requires some degree of estimation, even near-complete specimens are often missing vertebrae that must be filled in from close relatives
Limb elements	1. Strong biomechanical reason to believe they correlate with body mass [87, 162]	1. Limb bones rarely associated with diagnostic specimens 2. Can be biased when phylogenetic/paleoecological signal overrides size-based signal (e.g., fossoriality or differences in weight distribution between clades [31, 87];)

OCW accurately predicts body mass in mammals regardless of variables such as body proportions (i.e., most mammals had their weight accurately estimated despite differences in head size, neck length, limb proportions) or phylogeny (very distantly related taxa fell along the same regression line, unless occiput specializations were present). The lowest error values in the present dataset were primarily for mammals with generalized postcrania (e.g., didelphids, hyracoids, procyonids, many sciuromorphs, and cricetids) regardless of their phylogenetic position. This gives further evidence that OCW may work well for many wholly extinct groups of Paleogene or South American mammals, which tend to have generalized postcrania [44]. OCW accurately estimated body mass in the extant large-headed dasyuromorphians *Dasyurus viverrinus* (%PE_cf_ = 4.7%), *Dasyurus maculatus* (%PE_cf_ = 28.05%), *Sarcophilus* (%PE_cf_ = 27.19%), and *Thylacinus cynocephalus* (%PE_cf_ = 14.48%), suggesting it should be a strong predictor of body mass in extinct large-headed carnivorous mammals like sparassodonts and “creodonts.”

Because OCW correlates with body mass in nearly all therians regardless of phylogenetic position or body size, it can be used to estimate the body mass of taxa that belong to groups that are now totally extinct (e.g., South American ungulates, sparassodonts, “creodonts”), are only represented by a few morphologically similar living species (e.g., perissodactyls, proboscideans, and hyracoids), or are outside the range of body masses spanned by extant members of the group (e.g., the lagomorph *Nuralagus*, the giant caviomorph rodents *Josephoartigasia* or *Phoberomys*, or dwarf island proboscideans) without concerns about a lack of phylogenetic bracketing by extant species [83] or extrapolation of the data (except possibly in some of the most extreme cases, like *Paraceratherium* and some of the largest fossil proboscideans [165, 166]). However, representation for the upper end of the body size spectrum (> 500 kg) in the current dataset is very sparse, with only five specimens each represented by a single captive specimen, and this is an area where the present dataset could use improvement.

Applications of OCW to particular extinct mammal groups (e.g., the large-headed extinct mammal groups mentioned in the “Background”) is beyond the scope of the present study and is planned for future analyses currently in preparation by the author. However, as a case study, OCW was used to estimate the body mass of two large-headed extinct mammals for which previous body mass estimates based on craniodental variables have been considered dubious: the early Oligocene North American hyaenodont “creodonts” *Hyaenodon horridus* and *Hyaenodon crucians*. *Hyaenodon* is used as a case study because this genus is known from several nearly complete skeletons (see Fig. 1) and thus its body mass has been estimated using a number of both craniodental and postcranial proxies [32, 39, 58]. OCW produces an estimated body mass of 32.2 kg for *Hyaenodon horridus* (OCW = 42 mm) and 13.0 kg for *H. crucians* (OCW = 32 mm), comparable to estimates in previous analyses (Table 10). 95% confidence intervals for *H. horridus* and *H. crucians* are very large (Table 10), but this is related to log-transformation issues (i.e., if the data are log-distributed, the confidence intervals will be on a logarithmic scale as well and thus be very large when back-transformed into arithmetic units [39, 57, 58]). This is a problem present in nearly all body mass regression models, rather than unique to OCW. Indeed, confidence intervals for HBL, limb bone dimensions, and astragalar dimensions are very large and comparable to OCW (Table 10). Body mass estimates of *Hyaenodon* spp. using OCW agree with mass estimates produced with HBL or postcranial variables, but do not agree with the extremely high body mass estimates produced by skull length, demonstrating that OCW scales with postcranial variables and is not biased by the disproportionately large heads seen in hyaenodonts.

**Table 10:** Body mass estimates (in kg) and 95% confidence interval for *Hyaenodon* spp. in this study, compared to estimates in previous studies

*Hyaenodon horridus*			*Hyaenodon crucians*				
Mean	Lower	Upper	Mean	Lower	Upper	Measurement	Reference
32.2	14.9	69.3	13.0	6.0	27.9	OCW (all taxa)	Present Study
31.5	15.9	62.4	12.6	6.4	24.9	OCW (all taxa with condyle shape)	Present study
34.1	19.0	61.2	12.4	6.9	22.1	OCW (Carnivora-only)	Present Study
30.2	12.9	71.0	8.8	3.8	20.7	HBL	[39, 167]
130.6	47.5	359.3	30.5	11.2	83.2	Skull length	[39, 167]
38.4	28.1	52.6	–	–	–	Humeral trochlea area	[168]
41.4	24.9	43.3	15.0	9.1	24.8	“Limb bones”	[32]
25.8	12.8	51.8	10.6	5.3	21.3	Li1 (Astragalus)	[58]
29.2	13.9	61.1	11.0	5.3	23.1	Ar1 (Astragalus)	[58]

The equations of Morlo [78] were not used because this study estimated body mass in hyaenodonts by regressing molar row/average molar length against body mass estimates created using other proxies (done at least in part to avoid the issue of hyaenodonts having large heads), rather than directly estimating body mass from skeletal proxies in a sample of living taxa. The measurement from Egi [32] is listed as “limb bones” because Egi [32] does not detail which of the equations they produced were used to estimate the reported body mass. Note that body mass estimates reported here are not all drawn from the same individuals

The present study also highlights the importance of including OCW and other occiput measurements in multivariate analyses of craniodental morphology or geometric morphometric analyses. Even in cases where the primary objective of the study are interspecific shape differences rather than body mass, it is often of interest to distinguish shape differences that are driven by isometric size or allometric scaling (often measured by correlation with PC1 or centroid size [169]) from non-size-related differences in shape. Despite the strong correlation between OCW and body mass recovered here, a survey of the paleontological and zoological literature finds OCW (and occiput measurements more generally) to be one of the most rarely recorded morphological measurements in morphometric studies. Similarly, most morphometric analyses using traditional or geometric morphometrics do not record dimensions or landmarks of the occiput. For example, Mendoza et al. [86] and Figueirido et al. [41] considered a large number of craniodental variables (*N* = 25 and 39, respectively) in their multivariate regression equations of body mass in ungulates and carnivorans, respectively, but included almost no measurements of the occiput or foramen magnum. Mendoza et al*.* [86] did include occiput height, but there is reason to believe that this variable is less correlated with body size than other occiput dimensions and is influenced by dietary habits or paleobiology [64].

This issue is especially pertinent if there is reason to suspect that the taxon of interest has a disproportionately large head compared to extant taxa. This is because estimating body mass in extinct mammals using a dataset of craniodental data and body mass for extant species implicitly assumes that the extinct taxon of interest has a head that is the same size relative to its body as the taxa in the sample dataset. As a result, the dependent variable in a multivariate analysis of craniodental data (or centroid size in a geometric morphometric analysis) will implicitly correlate to the size of the head, not the size of the actual animal. Thus, increasing the number of craniodental variables in a body mass regression model may increase precision in the estimate, but will not increase accuracy. Because occiput dimensions (especially OCW) are shown here to correlate with the size of the entire body and not the cranium, the inclusion of occiput dimensions in multivariate or geometric morphometric analyses may help properly weigh variables to produce a more accurate relationship between craniodental features and body size.

### Limitations of OCW

The majority of mammals exhibit a similar occiput morphology, in which there are a pair of reniform condyles positioned laterally on either side of the foramen magnum (Fig. 7a). However, a few groups of mammals exhibit specialized occiput morphology that differs from the general mammalian condition, most of which can be groups into three distinct morphotypes (Fig. 7b–d). These morphotypes appear to differ on an almost categorial level, rather than representing an arbitrary extreme in a morphological shape gradient. However, it seems likely that intermediate states could exist in the fossil record. This may be because of the predicted high stabilizing selection on the occipito-atlantal joint in mammals: under most circumstances, there is strong selective pressure for the occiput to retain a consistent shape, but if a lineage enters an adaptive zone that puts different selective pressures on the occiput, there is extremely strong selection to a new local optimum because maintaining a stable occipito-atlantal joint is critical for Darwinian fitness. Because these taxa violate the assumption of that their occiput morphology is comparable to the general mammalian condition, OCW unsurprisingly failed to estimate body mass in these species. However, because of these morphotypes are highly distinctive fossil taxa that exhibit them can be identified as unsuitable for body mass estimation via OCW a priori, and thus are detailed further here.

The most common of these alternative occiput morphotypes are seen in lagomorphs (both leporids and ochotonids), caviids, and *Dipodomys* spp., hereafter called a “rabbit or lagomorph-like” occiput given it is most prevalent in this clade. These taxa have opisthobasally long, mediolaterally narrow condyles that are almost pulley-like in appearance (Fig. 7b). Because these condyles are narrower than would be expected in a mammal of their size, OCW consistently underestimates mass in these taxa. It is tempting to suggest this condition might be correlated with saltatorial/richochetal habits, but this condition is not exclusively correlated with this lifestyle. Some non-hopping mammals such as pikas or caviids have a lagomorph-like occiput morphology, whereas some ricochetal taxa (i.e., *Pedetes*) have a typical therian occiput morphology. The deviation from the Q-Q plot for normality is driven by these rabbit-like taxa.

However, taxa with a rabbit-like occiput form a regression line that is parallel to that of other therians (Fig. 8). Taxa with rabbit-like occiputs primarily differ from other mammals in terms of their *y*-intercept, with differences in slope between these taxa and the rest of the sample being non-significantly different. This indicates that the scaling relationship between OCW and body mass remains constant in rabbit-like taxa, and these taxa differ from other mammals in terms of occiput shape (i.e., narrow condyles) but not overall occiput size. This, in turn, means that it is possible to use OCW to estimate body mass in taxa with rabbit-like occiputs (and possibly other alternate morphotypes) provided corrections for morphological differences are made (i.e., including condyle morphotype as an additional categorical predictor variable, see “Results”). This agrees with previous studies that found OCW to be a good predictor of body mass within Lagomorpha [120].

The second major alternate occiput morphotype was found in monotremes, in which the condyles are low, mediolaterally long, and strongly diverge laterally from the borders of the foramen magnum (Fig. 7c), in contrast to most therians where the occipital condyles generally follow the border of the foramen magnum (Fig. 7a). This resulted in OCW being wider than expected, and thus OCW significantly overestimated body mass in the monotremes *Ornithorhynchus* (%PE_cf_ = 72.1%), *Tachyglossus* (62.2%), and *Zaglossus* (74.2%). This alternative occiput morphology also occurs in the extinct ornithorhynchid *Obdurodon* [170]. It is not clear whether the unusual morphology of the condyles seen in monotremes is plesiomorphic for mammals or represents an autapomorphy of Monotremata. The occipital condyles of the mammaliamorph *Adelobasileus cromptoni* [171], the multituberculates *Tombataar sabuli* [172] and *Kryptobaatar dashzevegi* [173], the non-therian zatherian *Vincelestes neuquenianus* [174: fig. 37b], and the gondwanatherian *Vintana sertichi* [175: Fig. 6] are all more similar to those of therians than monotremes. This suggests that the morphology seen in extant monotremes is an autapomorphy of Montremata, whereas therians represent the plesiomorphic condition. Nevertheless, because there are no living non-monotreme, non-therian mammals with known body masses to act as independent data points that can be used to verify if multituberculates and other extinct non-therian mammals follow the same regression line as extant therians, the regression equations in this study should be applied to non-therians with caution.

The third alternative morphotype was seen in cingulates, which have occipital condyles that are typically very robust, almost cylindrical in lateral view and rectangular in occipital view (Fig. 7d). This feature is also present in extant cingulates such as pampatheres and glyptodonts and is considered a synapomorphy of Cingulata [176]. As with monotremes, this resulted in greater than expected OCW, resulting in body mass being overestimated in taxa like *Euphractus sexcinctus* (%PE_cf_ = 55%) and *Zaedyus pichiy* (%PE_cf_ = 62%). The current sample size of Cingulata is too small (*N* = 6) to determine if these taxa form a distinct regression line parallel to that of other therians, similar to taxa with rabbit-like occiputs. By contrast, error in body mass estimates for most pilosans (specifically *Myrmecophaga tridactyla* [%PE_cf_ = 9.65%], *Tamandua tetradactyla* [5.86%], and *Choloepus hoffmanni* [5.42%]; *Bradypus variegatus* showed high underestimates of body mass [%PE_cf_ = 81.8%]), the sister taxon to Cingulata, were closer to other therians. Unusually, most specimens of *Dasypus novemcinctus*, one specimen of *D. sabanicola*, and one specimen of *Tolypeutes matacus* showed comparatively lower error values (%PE_cf_ < 50%). It is possible that the robustness of the occipital condyles could vary within Cingulata, with more fossorial species (such as *Euphractus*) having more robust condyles and less fossorial ones (such as *Tolypeutes*) having less robust condyles, but the present sample size was too small to test this idea.

The sole species of Dermoptera included in this analysis, *Galeopterus variegatus*, consistently showed an extreme underestimation of body mass (%PE_cf_ = 170%, estimated body mass = 529 g, actual mass 1429 g) representing the highest error for any taxon in which *N* > 1. A priori observations of specimens of *Galeopterus* in this study almost immediately noticed this taxon had an unusually small occiput relative to skull size. The high error in *G. variegatus* cannot be attributed to the gliding habits of this species, as other gliding mammals (*Idiurus macrotis*, *Petaurus breviceps*, *Glaucomys volans*) showed much lower degrees of error (%PE_cf_ for *I. macrotis* = 34.8%; *Petaurus breviceps,* 2.9%; *G. volans*, 14.8%). Exactly why *Galeopterus* has such a small occiput is unclear. The small occiput in *Galeopterus* does not appear to be the result of this taxon having a large head relative to body size; indeed, it actually has one of the smallest skull lengths relative to both head-body length and body mass in the present dataset. That is, *Galeopterus* has a smaller occiput than would be expected based on its already small skull length. The broader significance of this is unclear given only a single dermopteran species could be included in this analysis. Regardless of the reasons why this occurs, *Galeopterus* represents a clear outlier relative to other mammalian taxa.

The sole living representative of Dinomyidae, *Dinomys branickii*, has a unique occiput morphology among living mammals with distinct laterally projecting accessory condyles or paracondyles [177]. *D. branickii* shows a very high %PE (120.3%), but allometric comparisons beyond this are difficult given this is the only living taxon with such an arrangement.

A few other taxa exhibited relatively high %PE in the regression equation, including camelids, pseudocheirids, *Geocapromyx ingrahmi* (Capromyidae), geomyids, most arvicolines, and the sigmodontines *Sigmodon hispidus* and *Nectomys squamipes*. These taxa do not exhibit distinctive occiput morphology, but some of them have been described as having small brains relative to their body size [12, 178, 179]. Similarly, several taxa with high positive residuals have been described as having large brains relative to body size, including macroscelideans [180] and paucituberculatans [178]. This suggests that encephalization quotient may have some effect on OCW-based body mass estimates, which is a concern given that many extinct Paleogene and South American mammal groups are often described as having low encephalization quotients [11]. However, including brain mass as an additional independent variable did not greatly affect model accuracy, differences between mass estimates including and excluding brain mass were low, and the correlation between encephalization quotient and the residuals of the regression equation has a low *r*^2^ value, suggesting if bias does exist it is minor. Additionally, encephalization quotients did not always correlate with high residuals. Some species known to have very low encephalization quotients, including *Marmota* spp., Eulipotyphla, and Peramelemorphia [12, 181], had their body mass accurately estimated using OCW (Mean %PE_cf_ = 0.38 for *Marmota* spp., see Table 5 for %PE for Eulipotyphla and Peremelemorphia). Future multivariate methods of estimating body mass including multiple basicranial measurements (compare with [182, 183]) may be able to produce more accurate results.

Overall, OCW shows a very strong correlation with body mass in most mammal groups assuming a generalized occiput morphology. Most groups that show high residuals also violate the assumption of geometric similarity; they show alternate states of occiput morphology that deviates radically in shape from the generalized mammalian condition and thus cannot be directly compared. These taxa with specialized occiputs can be readily identified in osteological specimens, which suggests they should also be readily recognizable in the fossil record (e.g., comparable states can be seen in *Nuralagus* [184] and *Obdurodon* [170]). However, intra-group comparisons between taxa with similar condylar morphology (i.e., lagomorphs and similar taxa) show a similar scaling relationship, suggesting that differences between taxa are primarily ones of shape rather than different allometric relationships and that there is a common allometry between all species. Other taxa show high residuals for unclear reasons, identifying particular biological or metric reasons for this pattern or further sampling to determine if these patterns are real or potentially due to individual variation in body condition, especially given that the functional morphology of the occiput is poorly studied.

The fact that OCW, HBL, and skull length all more accurately predict one another than any of these linear measurements predicted body mass suggests that much of the residual variation in this study and error in the regression equation is driven by individual variation in body condition. Namely, it implies that all three of these measurements broadly agree with one another when it comes to describing the geometric size of an organism, but lack key information that prevents them from accurately predicting individual body mass. This is reminiscent of the results of Sarko et al. [69] and Churchill et al. [126], who also found OCW to more accurately predict body length than body mass in sirenians and pinnipeds, respectively, and also attributed this to individual variability in body mass. There are many potential sources of individual variation in body mass that would not be expected to be reflected in skeletal morphology, including sexual dimorphism (males in many mammal species are more massive than females of the same size due to higher lean muscle mass [185–187]), age (most mammals typically achieve adult linear dimensions by the time of sexual maturity but continue to “fill out” and gain mass afterwards, and then mass may be lost as part of senescence [188–191]), seasonality (e.g., fat reserves in hibernating species), reproductive status (i.e., pregnancy), individual body condition, and the weight of the gut contents [39]. Additionally, there are also significant sources of interspecific error in mass due to soft tissue distribution, such as species-specific differences in fat reserves or muscle mass [102, 108, 111, 192]. Decomposition and fluid loss can be other significant sources of error in recorded body masses [126, 193], especially in large taxa which often have to be weighed piecemeal (fluid loss may account for 3–7% live body mass in large animals such as rhinoceroses and elephants [193];). By contrast, osteological or body measurements like OCW, HBL, or skull length are much less susceptible to individual variation due to environmental conditions, and cannot easily increase or decrease in adults the way body mass can.

The idea that much of the residual variation and error in the OCW regression equation is driven by variation in body condition is supported by the general observation that when it was possible to select from large sample sizes of specimens the absolute value of the residuals for these taxa was generally lower (Welch two sample *t* test; *t* = 3.3755, df = 168.51, *p* < 0.001; Additional file 8). This is likely because in these species it was possible to select “optimal” individuals that were close to the mean value for the entire population and avoid extremes in body condition, rather than simply measuring whatever individuals happened to have body mass recorded. However, it is also plausible that the lower error values for these taxa are a consequence of larger sample size, as most of these taxa had *N* > 10. Indeed, this is the exact reason why most studies of body mass use the average value of several individuals in the first place, in the hopes that differences between individuals will average out and hopefully produce a truer estimate of the relationship between skeletal proxy and body mass in the species as a whole [53, 129, 133].

### Applying occipital condyles beyond mammals

OCW also has potential applications beyond just mammals. Indeed, Anderson [194] used occipital condyle dimensions as a proxy for body size in *Triceratops*. Nevertheless, there are several potential issues with using OCW to estimate mass in non-mammals that are not present in therian mammals. First, extinct non-mammalian amniotes span a much greater range of body sizes than extant amniotes. The largest non-mammalian amniote is the saltwater crocodile (*Crocodylus porosus*), whereas many extinct sauropsids greatly exceed living crocodylians in size (e.g., many non-avian dinosaurs). This means that estimating body mass using occipital condyles in these groups requires significant extrapolation beyond extant taxa [137, 195]. By contrast, most extinct mammals are within the range of body sizes spanned by living taxa [165, 166, 196].

Secondly, a large number of extinct amniote groups are not well-bracketed by living representatives. These include non-mammalian synapsids, which are bracketed by sauropsids and extant mammals. Sauropsids and extant mammals share a non-analogous occiput morphology, with sauropsids having a singular median condyle ventral to the foramen magnum and extant mammals having paired condyles positioned lateral to this foramen [197]. Paired occipital condyles seem to have originated at the base of Cynodontia [198], but Rowe [199] noted that non-mammaliaform cynodonts exhibit a transitional morphology where the condyles are positioned more ventrally than in modern mammals. Rowe [199] considered non-mammalian mammaliaformes (i.e., Morganucodontidae) to also exhibit this condition, but the condyles of *Morganucodon* [200] and *Adelobasileus* [171] show a condition similar to extant therians. Occipital condyle morphology varies greatly in non-cynodontian synapsids, with stem (“pelycosaurs”) exhibiting a single, sauropsid-like occiput [201, 202]; biarmosuchians exhibiting a strange condition where the exoccipital forms paired structures lateral to the foramen magnum distinct from the condyle that may contribute to occiput function [203, 204]; gorgonopsians exhibiting an unusual “kidney-shaped” occipital condyle [201]; and dinocephalians, anomodonts, and therocephalians exhibiting a tripartite occiput [201, 205, 206] unlike either mammals or sauropsids (with the condyle of therocephalians sometimes being notched as in gorgonopsians [206];). Similarly, non-avian archosaurs are bracketed by extant crocodilians and neornithe birds, but given the extreme differences in body shape between these two groups, it is unlikely that a regression equation based on both groups would produce accurate results.

Third, extinct non-amniotes exhibit much more diversity in body shape than terrestrial mammals. Most terrestrial mammals have a relatively conservative quadrupedal body plan, and it is for this reason that differences in body shape between mammal species are assumed to contribute little to the relationship between OCW and body mass. By contrast, body shape in other amniotes can involve significant differences in head size (e.g., the very large heads of ceratopsians or the very small heads of stegosaurs and sauropods), neck length (e.g., sauropodomorphs and sauropterygians), presacral vertebral count [207], limb posture (sprawling versus erect limbs), tail length [22, 113], and limb proportions (e.g., bipedalism versus quadrupedalism [112]) that would be expected to increase error in the correlation between occiput dimensions and body mass. Overall, it is possible that the results found here for mammals could be applied to other amniotes like sauropsids, but there are other considerations that suggest the correlation between occiput dimensions and body mass might not be as straightforward in non-mammalian groups.

## Conclusions

Occipital condyle width (OCW) is found to be a strong predictor of body mass in mammals, especially given the constraints of the present dataset (high phylogenetic breadth, extreme range of body sizes, no phylogenetic correction or separate regression lines for species of different diets or locomotor habits). Although there are groups for which OCW does not accurately predict body mass, these groups are characterized by highly recognizable, specialized occiput morphology and can be identified as unsuitable for this method a priori, in contrast to other size proxies which require nearly complete remains to identify the discrepancy (e.g., craniodental features in hyaenodontsors skull length and femoral cross-section in large caviomorphs). The low inter-ordinal variability seen in most species makes OCW a particularly useful method for estimating mass in extinct species for which dental or postcranial remains are either unknown or are considered to be poor correlates of body mass. Additionally, it provides another method of estimating body mass partially independent of the biases seen in traditional metrics (cranio-dental dimensions, HBL, limb bone dimensions). Along with recent studies on the scapula, astragalus, and calcaneus, the present study illustrates the value of utilizing variables from other regions of the body in addition to traditionally preferred metrics of the dentition and long bones when estimating body mass in extinct mammals.

## Methods

### Data collection

Occipital condyle width (OCW) and body mass were collected from 2127 specimens of mammals with associated body mass representing 404 species and 91 families of non-volant terrestrial mammals, including all extant terrestrial mammal orders except Notoryctemorphia. These specimens come from the collections of the American Museum of Natural History (AMNH), Carnegie Museum of Natural History (CM), Cleveland Museum of Natural History (CMNH), Cornell University Museum of Vertebrates (CUMV), University of California Museum of Vertebrate Zoology (MVZ), Núcleo de Pesquisa em Ecologia e Desenvolvimento Sócio-Ambiental de Macaé (NPM), Sam Noble Museum of Natural History (OMNH), University of Florida/Florida Museum of Natural History (UF), University of Michigan Museum of Zoology (UMMZ), Smithsonian Museum of Natural History (USNM), Burke Museum of Natural History and Culture (UWBM), University of Wyoming Museum of Vertebrates (UWYMV), and the Yellowstone National Park Archives (YELL). This sample (including the additional specimens listed below) represents approximately 71.6% of all currently recognized recent terrestrial mammal families [208].

Data for seven additional specimens (see Additional file 12) were obtained from photographs of specimens housed at the University of Alaska Museum (UAM) on ARCTOS (arctos.database.museum) and the Harvard Museum of Comparative Zoology (MCZ) from the MCZ database (https://mcz.harvard.edu/database). With regard to primates, data for 205 specimens from the USNM were measured from X-ray images provided by Terry Ritzman to the USNM Collections Database (https://collections.nmnh.si.edu/search/mammals/). Data for the sole proboscidean specimen used in this analysis (*Loxodonta africana*, ROM R6000/96185) was taken from Jukar et al. [121], data for *Lagidium ahucaense* was taken from photographs in Ledesma et al. [209], and data for *Ichthyomys stolzmanni* and *I. tweedii* were taken from Brito et al. [210] and Ramírez-Fernández et al. [211], respectively. Data for the specimen of *Zaglossus bruijni* (AMNH 157072) and *Hydromys chrysogaster* (MVZ 175330) were measured from CT scans from MorphoSource (https://www.morphosource.org).

The primary purpose of this study was to examine scaling relationships of OCW across generalized terrestrial mammals. Part of this is based on the theoretical assumption that because most mammals have a roughly similar body plan their proportions are relatively consistent (primarily differing in limb length) and thus axial dimensions would be expected to be correlated to body mass. Marine mammals (Cetacea, Pinnipedia, and Sirenia), bats, and subterranean talpids were not considered because their body plan deviates radically from this generalized body plan and thus might violate assumptions that postcranial morphology is comparable among examined taxa. Although subterranean talpids (e.g., *Talpa*, *Condylura*) were not considered; the less-specialized [212] American shrew-mole *Neurotrichus gibbsii* was included given it has a body plan similar to other eulipotyphlans. Chrysochlorids and notoryctemorphians were also not considered due to lack of available specimens with associated body mass.

**Fig. 10:**
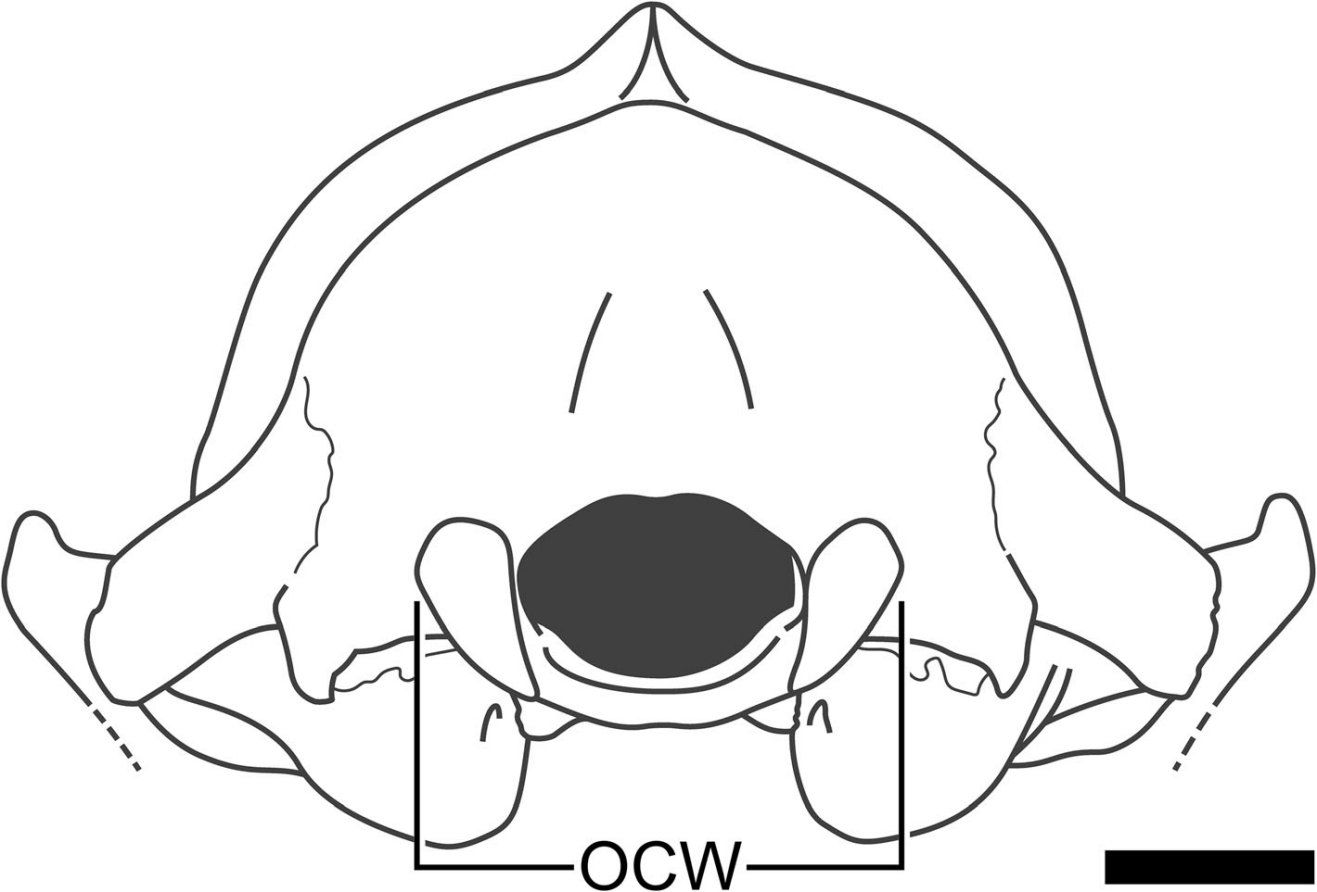
Line drawing of skull of *Procyon lotor* (CMNH 22076) in occipital view illustrating how occipital condylar width (OCW) was measured in this analysis. Scale = 1 cm

OCW was measured as the greatest transverse width across the occipital condyles (Fig. 10) to the nearest 0.01 mm using a Mitutoyo Digimatic caliper. All body mass data used in this study (in g) represent tag data directly associated with each specimen. Average OCW and body mass for each species can be found in Additional file 12, and individual values for each specimen are listed in Additional file 13. Few body mass estimations in extinct mammals are based on models where mass and measurement data are drawn from the same individuals due to availability of data [213]. Instead, body mass data in these studies are typically mean values for the species reported in the previously published literature. However, this can lead to errors in body mass estimations as bone and weight measurements do not correspond to the same sample of individuals (see [214]).

When not constrained by availability of specimens, at least ten individuals were measured for each species, ideally consisting of five male and five female individuals to account for sexual dimorphism in a given taxon. Specimens from adult, wild-caught individuals were used whenever possible. However, for many large-bodied species, no mass data were available for wild individuals. Most collectors only began regularly recording body mass data for mammalian specimens in the 1980s (S. McLaren pers. comm., 2015, J. Martin pers. comm., 2015), and even afterwards associated weights for large mammals are rare due to ogistical difficulties. As a result, specimens from zoological parks are often the only option to get associated weight and skeletal measurement data for many taxa. Domesticated or feral animals were not considered due to artificial selection or anthropogenic food provisioning potentially biasing the relationship between OCW and body mass.

Body mass and OCW for each specimen were cross-referenced with other measured specimens of the same species in the present study and/or reported body masses for the species in the previously published literature to ensure each specimen was representative of the species. Specimens were only excluded if the recorded body mass was outside of the range of reported adult body masses in the literature, suggesting the specimen was not a reasonable representative of the species (or that some form of lapsus arose during the original measurement, such as recording “eviscerated” weight as live weight, which is a common issue for tag weights of game animals). For some species, hundreds or even thousands of specimens with body mass were available (e.g., many rodents), and it was not feasible to measure every available individual. In these cases intraspecific regression equations were created by regressing HBL against body mass of available specimens as an index of body condition [215, 216], and individuals with the lowest residuals were used.

### Analysis

All analyses and statistical calculations were performed in R 4.1.1 [217]. R code used to perform this analysis can be found in Additional file 14, and a knitted .html document showing the direct results of all analyses performed in this study can be found in Additional file 2. Regression models were created using the average, natural log-transformed body mass and OCW for each species. Species averages were used as is standard in comparisons of interspecific data (see [129] for more information). Curve fitting was performed with the nls function and the package *nlstools* [218]. The relative strength of the best-fit model against several alternate models (linear, power, quadratic/cubic equations) was evaluated using an Akaike Information Criterion (AIC [219]) and a Bayesian Information Criterion (BIC [220]). For both criteria, more negative numbers generally mean better fits.

Most mammals exhibited a roughly comparable occiput morphology across all taxa (Fig. 7a), in which the condyles are reniform and follow the margins of the foramen magnum. However, several taxa, specifically monotremes, dermopterans, lagomorphs, certain hystricomorphs (Caviidae and Dinomyidae), and ricochetal heteromyids (i.e., *Dipodomys*) exhibited one of several highly distinctive alternate states of occipital condyle morphology (see Fig. 7b–b and “Discussion” for more details) that might violate assumptions of morphological similarity. These alternate states of occiput morphology are readily recognizable in osteological specimens (i.e., they can be recognized in extinct taxa), and thus regression equations were also calculated excluding these groups to determine the accuracy of the correlation including only specimens with a generalized mammalian morphology. Regression equations were also calculated using a sample of all species for which large numbers of individuals (≥ 6 and ≥ 10) could be measured.

Taxon-specific regression equations were calculated for Australidelphia, Carnivora, all rodents, Sciuromorpha, and “ungulates” (i.e., Artiodactyla, Perissodactyla, and Hyracoidea), given their large sample size (Table 4) and observed range of body sizes and the fact that taxonomically restricted datasets are generally said to have better predictive power [66, 86, 134]. Sciuromorpha were examined both separately and as part of the all rodent dataset in order to test if narrower taxonomic selectivity produced improved accuracy rates, as has been suggested by some authors [134]. Sciuromorphs were ideal for this purpose, as a large number of sciuromorph taxa (*N* = 29) spanning a wide range of body sizes (~ 50–5000 g) could be measured, reducing concerns that taxonomic regression lines might be biased by a limited range of body sizes.

One concern with the present dataset is that error rates in the regression equation might be biased by oversampling of small mammals. Even if OCW produces a low %PE in extant mammals as a whole, if the sample dataset is biased by large numbers of small mammals such as rodents (which represent roughly half of all living mammals [208]), it might not produce an accurate estimate of how well the equation performs in larger mammals. Small mammals tend to be generalized and postcranially conservative [221], whereas larger mammals tend to exhibit more postcranial diversity and more extreme anatomical modifications for the same lifestyles [13], possibly due to physical demands on the postcranium increase with increasing body size. This is a concern because most of the specimens OCW can be applied towards are larger mammals, cranial morphology being rarely preserved in fossil micromammals. As a result, I performed an additional analysis including all taxa for which body mass was > 1 kg.

To correct for log-transformation bias [222, 223], three correction factors were calculated: the quasi-maximum likelihood estimator [222, 223], smearing estimate [222–224], and ratio estimator [222, 223, 225].

$$ \mathrm{Quasi}-\mathrm{maximum}\ \mathrm{likelihood}\ \mathrm{estimator}=\exp \left(\frac{s^2}{2}\right) $$

where *s*^2^ = residual mean square of the regression equation,

$$ \mathrm{smearing}\ \mathrm{estimate}=\frac{\sum \exp \left(\log \left({r}_i\right)\right)}{N} $$

where *N* = number of observations and *r*_*i*_ = residual of data point *i*, and

$$ \mathrm{ratio}\ \mathrm{estimator}=\frac{y}{\hat{y}} $$

where *y* = the mean of the observed values for the dependent variable and $\hat{y}$ = the mean of the predicted value for this variable without correction. These corrections factors were averaged to calculate a mean correction factor following the methodology of Tsubamoto [58], and corrected estimates were calculated by multiplying the uncorrected body mass estimates by the correction factor.

Accuracy of the correlation between OCW and body mass was primarily examined using mean percentage prediction error (%PE) and percent standard error of the estimate (%SEE). As mentioned in previous studies [39, 226], the correlation coefficient (*r*^2^) is not a good measure of the accuracy of a log-log regression as *r*^2^ is very sensitive to the range of the data. Data with high log-scaled ranges produce high *r*^2^ values even when the actual predicting power of variables is low. %PE was calculated as

$$ \%\mathrm{PE}=\frac{\mathrm{observed}\ \mathrm{value}-\mathrm{estimated}\ \mathrm{value}}{\mathrm{estimated}\ \mathrm{value}}\times 100 $$

following previous studies. Mean %PE was calculated as

$$ \%\mathrm{PE}=\frac{\sum \left|\%\mathrm{PE}\right|}{\mathrm{N}} $$

%PE has been suggested to be an suboptimal metric to gauge accuracy in regression equations [134] as it may overpenalize underestimates of body mass due to using the predicted value as a denominator (similar to how another estimate of accuracy, mean absolute percentage error, penalizes overestimates [227]). However, %PE was used in this study in order to compare the results of this analysis with previous studies, which also used %PE. %SEE for natural log transformed variables was calculated following Ruff [57], in which

$$ \%\mathrm{SEE}=\exp \left(\mathrm{SEE}+4.6052\right)-100 $$

where SEE = standard error of the estimate.

To test for phylogenetic signal in the present dataset OCW was regressed against body mass using phylogenetic generalized least squares regression (PGLS) via the R packages *ape* [228], *phytools* [229], *sensiPhy* [230], *nlme* [231], and *geiger* [232], and a pruned mammal phylogeny from Upham et al. [233] downloaded from vertlife.org (Additional file 15). The strength of phylogenetic signal was measured using Pagel’s *λ* [234].

The accuracy of OCW was compared to two other commonly used estimators of body mass: skull length (measured as condylobasal or condyloincisive length, CBL), and head-body length (HBL). Skull length has been considered to be one of the best estimators of body mass in certain groups of mammals [155, 158], though in some groups such as carnivorans other metrics are considered more accurate predictors of body mass [39]. HBL, on the other hand, has often been considered to be one of the most accurate estimators of body mass when available [39, 52, 66]. Skull length was mostly compiled for the taxa using previously published values. HBL was calculated based on a mix of literature data and actual tag data associated with the specimens (see Additional file 12 for more details). Regression equations were calculated for both variables using the body mass from the present dataset and methodologies described above.

In order to test for potential bias in the dataset due to mixing data from both captive and wild individuals and the tendency of most species to become obese while in captivity [235, 236], a phylogenetic multivariate generalized linear mixed model using Markov chain Monte Carlo techniques was fit using the *MCMCglmm* package [237] in R. OCW and captivity status were treated as fixed effects, whereas phylogenetic signal was calculated using the dataset of Upham et al. [233].

Relative brain size was identified as a potentially confounding variable during the course of the study. To examine the effects of brain size on the regression equations, I compiled a list of published brain masses available for 280 of the examined species (roughly 78.2% of the dataset) primarily from Burger et al. [12] with a few additions from other sources (see Additional file 12 for more details), and ran analyses treating brain mass as an additional independent factor variable. In order to avoid bias by comparing results from different samples, the results of this analysis were compared to a single-variate regression using only those 280 taxa for which brain mass could be obtained.

As a case study in the use of OCW for estimating body mass in fossil taxa, body mass was estimated using OCW in the early Oligocene North American hyaenodont “creodonts” *Hyaenodon cruciens* and *H. horridus*. OCW was measured from the figures of the occiput of *H. cruciens* and *H. horridus* in Lange-Badré [238]. *Hyaenodon cruciens* and *H. horridus* were chosen specifically because these species are known from nearly complete remains and have had their body mass estimated by different authors using a variety of skeletal proxies [32, 39, 58].

## Supplementary Information

**Additional file 1: Figure S1**. Plots of variables examined versus body mass. **A**, OCW versus body mass assuming isometry. **B**, OCW versus body mass with OCW raised to the 2/3 power. **C**, condylobasal length versus body mass assuming isometry. **D**, skull length versus body mass raised to the 1/2 power. **E**, HBL versus body mass. No power transformation for HBL is included as the non-linear fit indicates that the relationship between natural log HBL and natural log body mass is linear. For **A** and **C**, blue lines are linear regression lines and red dashed lines are loess fit lines. Note how the linear regression lines in **A** and **C** do not precisely follow the trend of the data, overestimating body mass at the extremes and underestimating it in the middle ranges of the data set (.tiff).

**Additional file 2.** Knitted html report showing the raw results of the analyses performed in this study (.html).

**Additional file 3: Figure S2**. Scatterplot of natural log of OCW versus natural log of body mass, comparing the best fit curve between a linear (in red), 2/3 power (in blue) and quadratic model (in green). Dashed lines represent the 95% prediction intervals (.tiff).

**Additional file 4: Figure S3**. Scatter plot (**A**) and boxplot (**B**) of sample size versus absolute value of the residuals of the regression equation between log OCW and log body mass (.tiff).

**Additional file 5: Figure S4**. Ordinal-level regression equations of log OCW and log body mass for various orders of mammals (and suborders of rodents) for which ≥5 species are sampled. The dashed black line represents the best fit line of the total dataset. Data points which pertain to clades for which N < 5 are denoted in gray (.tiff).

**Additional file 6: Figure S5**. Histogram (**A**) and Q-Q plot (**B**) of the residuals for the rodent regression equation between log OCW and log body mass (.tiff).

**Additional file 7: Figure S6**. Residuals of the all-species regression equation of log OCW versus log body mass plotted onto a phylogeny of the examined taxa. Higher than expected body masses are shown in reds and yellows and lower than expected body masses are shown in cyans and blues. There is very little variation in the residuals across most of the model, suggesting a lack of Brownian motion in the evolution of this trait, but there are extreme shifts in residual values at the base of several clades such as Lagomorpha and Monotremata (.pdf).

**Additional file 8: Figure S7**. **A**, box plot of residuals versus captivity status for all specimens. **B**, box plot of residuals versus natural log of body mass (in g) for all specimens. **C**, box plot comparing species average residuals for species in which it was possible to be selective about what specimens were chosen versus species in which it was not possible to be selective (.tiff).

**Additional file 9: Figure S8**. Plot of the residuals of the regression of OCW and body mass against the residuals of the regression between brain mass (scaled to the 3/4 power) and body mass, showing that the residuals in relative brain size are not strongly correlated with residuals in OCW (.tiff).

**Additional file 10: Figure S9**. Plot of the residuals of the regression equation including brain mass as an independent variable and the residuals of the regression of the same data where brain mass is not included. Red line represents a line with intercept of 0 and slope of 1, blue line represents OLS fit. If including brain size significantly improved estimates, it would be expected that the slope would be much shallower than 1 due to residuals for extreme values being lower (.tiff).

**Additional file 11: Figure S10**. Plot of the residuals for the regression of OCW and body mass against the residuals for the regressions of head-body length (**A**) and skull length (**B**) against body mass (.tiff).

**Additional file 12: Table S1**. Database of average OCW and body mass in all specimens measured, as well as skull length (measured as condylobasal or condyloincisive length when available), HBL, and brain mass for each species compiled from the previously published literature (.csv) [12, 136, 180, 209–211, 239–564].

**Additional file 13: Table S2**. Database of the individual specimens measured in this analysis, including their raw measurements, gender, and captivity status (.csv).

**Additional file 14.** R code used to perform the analyses in this study, in Rmarkdown format (.Rmd).

**Additional file 15.** Phylogenetic tree used to perform the tests for phylogenetic signal and PGLS, in NEXUS format (.nex).

## Data Availability

All data generated or analyzed during this study are included in this published article (and its supplementary information files).

## Abbreviations

AMNH: American Museum of Natural History, New York, USA
CM: Carnegie Museum of Natural History, Pittsburgh, USA
CMNH: Cleveland Museum of Natural History, Cleveland, USA
CUMV: Cornell University Museum of Vertebrates, Ithaca, USA
MECN: Museo Ecuatoriano de Ciencias Naturales
MCZ: Harvard Museum of Comparative Zoology, Cambridge, USA
MNPN: Escuela Politécnica Nacional, Ecuador
MNCR: Museo Nacional de Costa Rica, San José, Costa Rica
MVZ: University of California Museum of Vertebrate Zoology, Berkeley, USA
NPM: Núcleo de Pesquisa em Ecologia e Desenvolvimento Sócio-Ambiental de Macaé, Macaé, Brazil
OMNH: Sam Noble Museum of Natural History, Norman, USA
ROM: Royal Ontario Museum, Toronto, Canada
UAM: University of Alaska Museum, Fairbanks, USA
UF: University of Florida/Florida Museum of Natural History, Gainesville, USA
UMMZ: University of Michigan Museum of Zoology, Ann Arbor, USA
USNM: Smithsonian Museum of Natural History, Washington D.C., USA
UWBM: Burke Museum of Natural History and Culture, Seattle, USA
UWYMV: University of Wyoming Museum of Vertebrates, Laramie, USA
YELL: Yellowstone National Park Archives, Gardiner, USA
AIC: Akaike Information Criterion
BIC: Bayesian Information Criterion
CBL: Condylobasal length of skull
CF: Averaged correction factor (see Methods)
HBL: Head-body length
OCW: Occipital condyle width
PE: Prediction error
PGLS: Phylogenetic generalized least squares regression
SEE: Standard error of estimate
