# Author Correction: Occipital condyle width (OCW) is a highly accurate predictor of body mass in therian mammals

**DOI:** 10.1186/s12915-022-01261-y

**Published:** 2022-02-23

**Authors:** Russell K. Engelman

**Affiliations:** Department of Biology, Case Western Reserve University, 10,900 Euclid Avenue, Cleveland, OH 44106 USA

**Correction to: BMC Biology 20, 1–44 (2022)**

**https://doi.org/10.1186/s12915-021-01224-9**

The original article contained an error whereby the Mean % PE_cf_ value for Diprotodontia of 46.25% was mistakenly omitted after being removed from Table 5 during proofing.

In addition, Figs. 2 and 3 were mistakenly swapped.

These errors have since been corrected.

