# Supplementary figures and images for: Occipital condyle width (OCW) is a highly accurate predictor of body mass in therian mammals
Source: BMC Biol. 2022 Feb 7;20:37. doi: 10.1186/s12915-021-01224-9 (PMC8883515; doi:10.1186/s12915-021-01224-9)

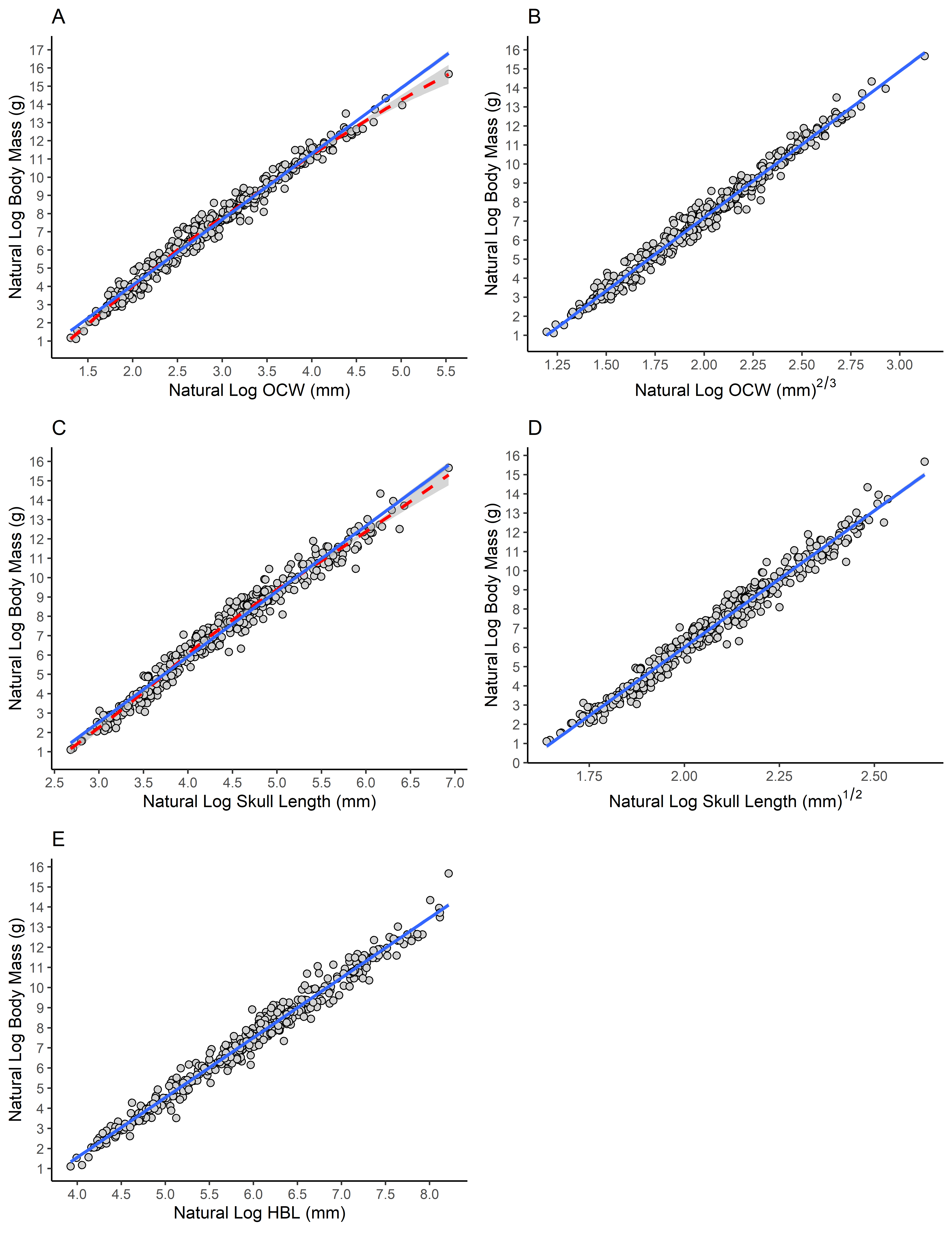

Supplement: Supplementary file 1 — Additional file 1: Figure S1. Plots of variables examined versus body mass. A, OCW versus body mass assuming isometry. B, OCW versus body mass with OCW raised to the 2/3 power. C, condylobasal length versus body mass assuming isometry. D, skull length versus body mass raised to the 1/2 power. E, HBL versus body mass. No power transformation for HBL is included as the non-linear fit indicates that the relationship between natural log HBL and natural log body mass is linear. For A and C, blue lines are linear regression lines and red dashed lines are loess fit lines. Note how the linear regression lines in A and C do not precisely follow the trend of the data, overestimating body mass at the extremes and underestimating it in the middle ranges of the data set (.tiff). [file 12915_2021_1224_MOESM1_ESM.tiff]

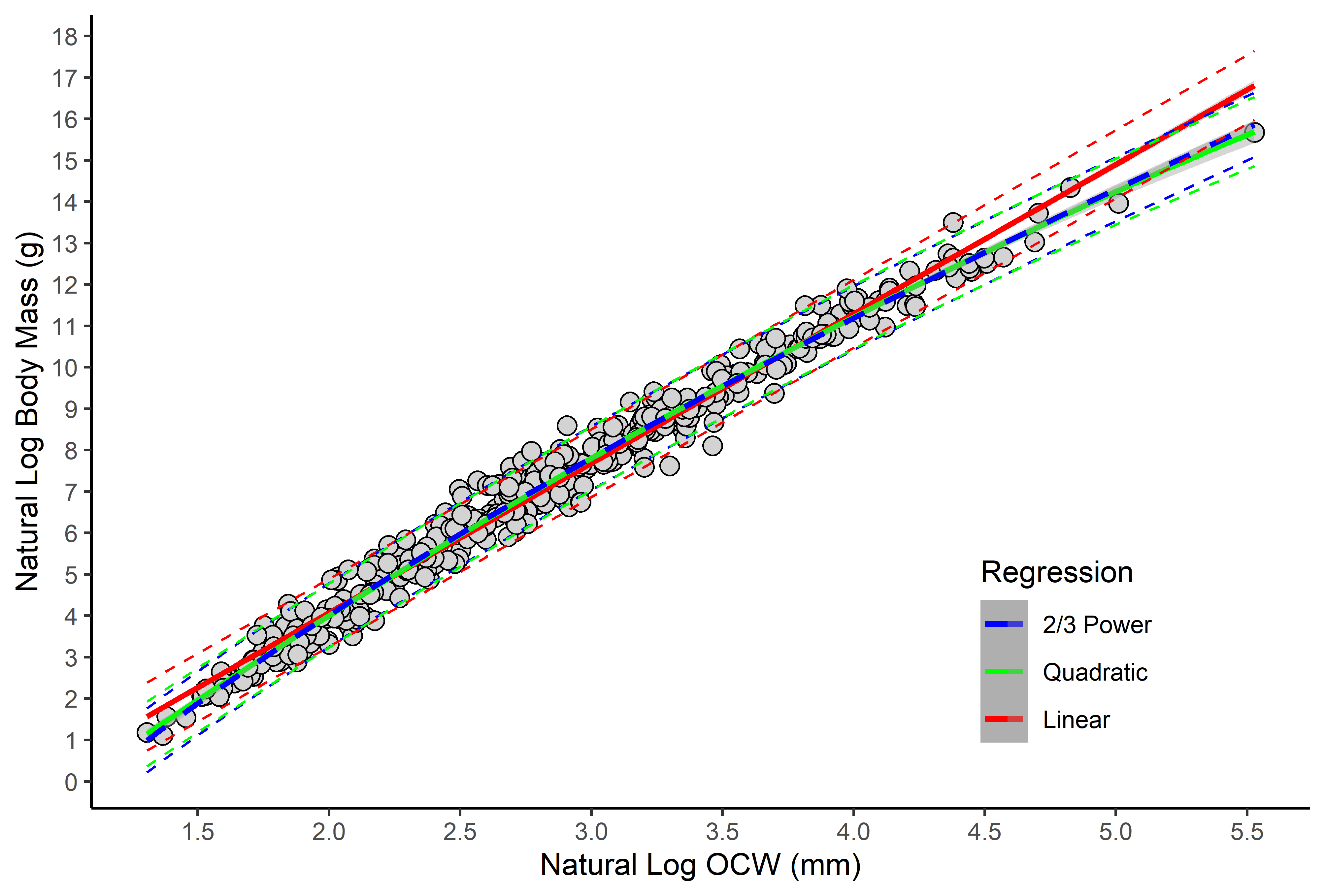

Supplement: Supplementary file 3 — Additional file 3: Figure S2. Scatterplot of natural log of OCW versus natural log of body mass, comparing the best fit curve between a linear (in red), 2/3 power (in blue) and quadratic model (in green). Dashed lines represent the 95% prediction intervals (.tiff). [file 12915_2021_1224_MOESM3_ESM.tiff]

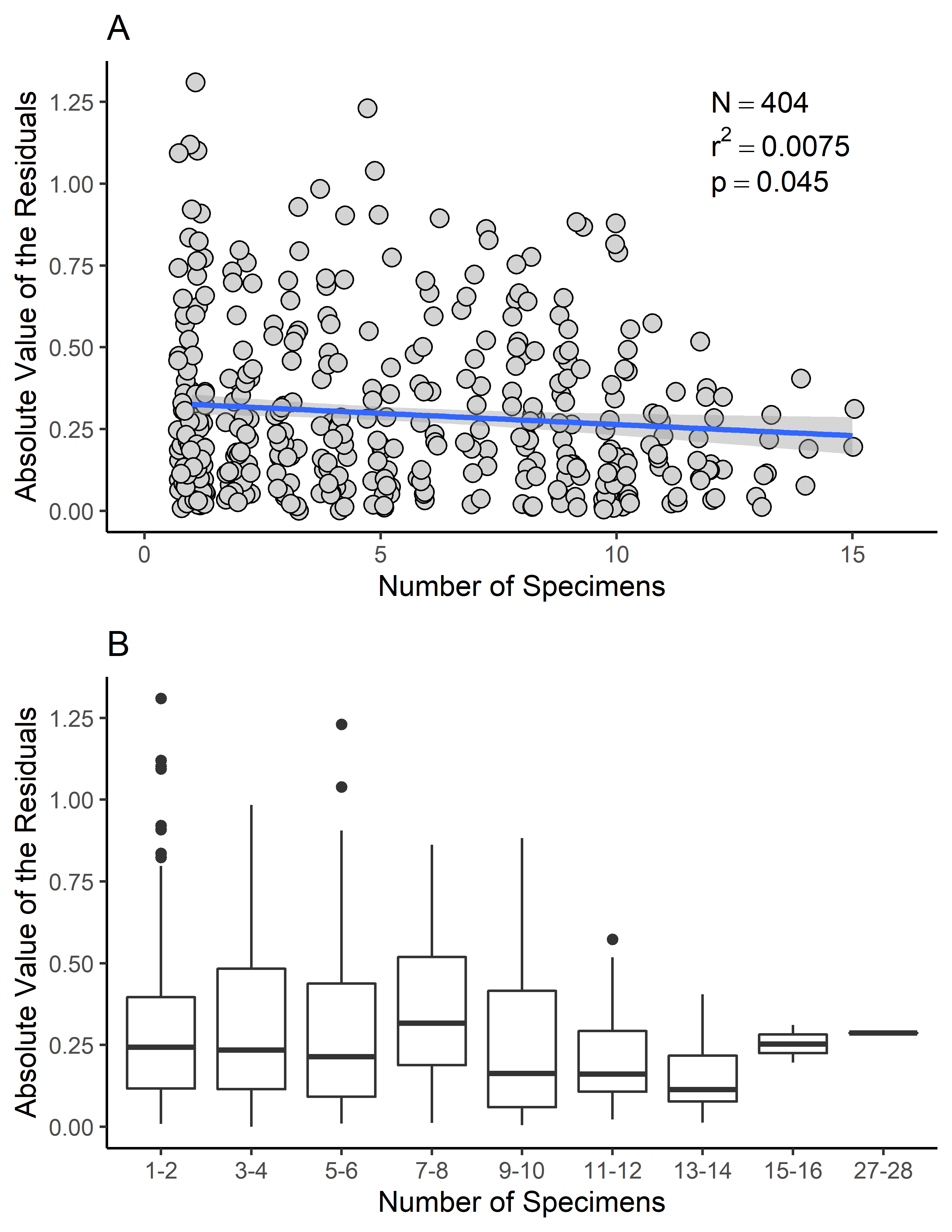

Supplement: Supplementary file 4 — Additional file 4: Figure S3. Scatter plot (A) and boxplot (B) of sample size versus absolute value of the residuals of the regression equation between log OCW and log body mass (.tiff). [file 12915_2021_1224_MOESM4_ESM.tiff]

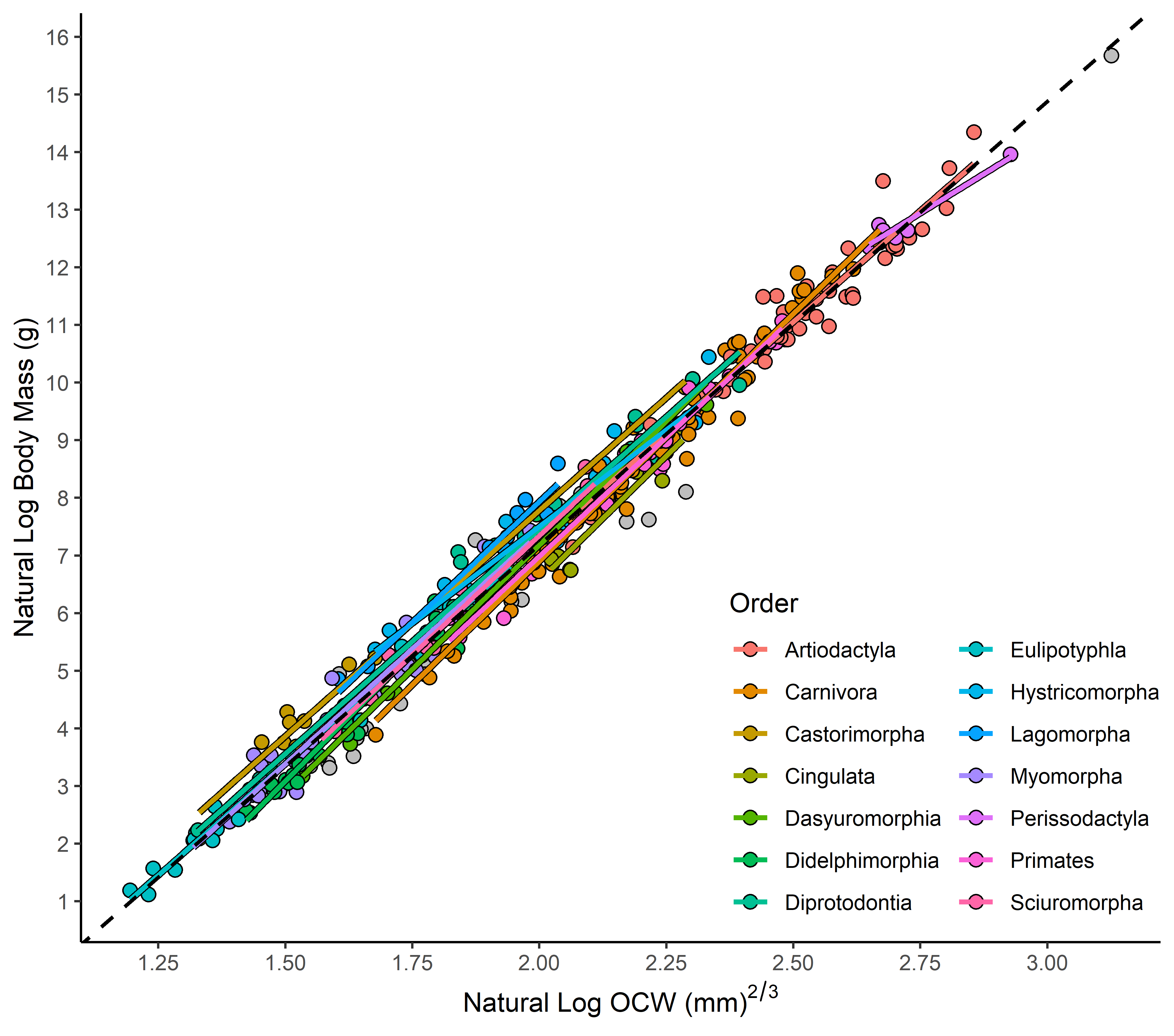

Supplement: Supplementary file 5 — Additional file 5: Figure S4. Ordinal-level regression equations of log OCW and log body mass for various orders of mammals (and suborders of rodents) for which ≥5 species are sampled. The dashed black line represents the best fit line of the total dataset. Data points which pertain to clades for which N < 5 are denoted in gray (.tiff). [file 12915_2021_1224_MOESM5_ESM.tiff]

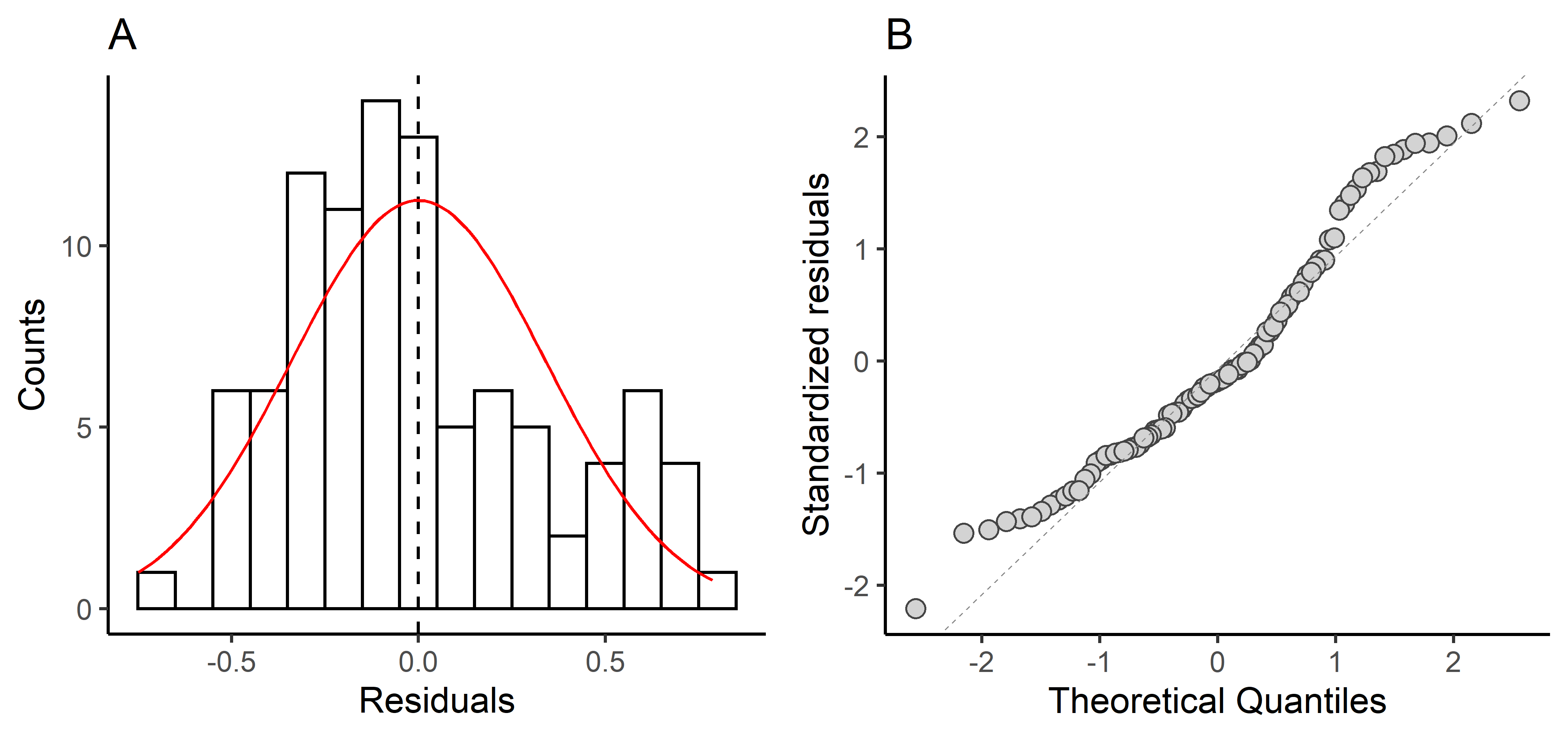

Supplement: Supplementary file 6 — Additional file 6: Figure S5. Histogram (A) and Q-Q plot (B) of the residuals for the rodent regression equation between log OCW and log body mass (.tiff). [file 12915_2021_1224_MOESM6_ESM.tiff]

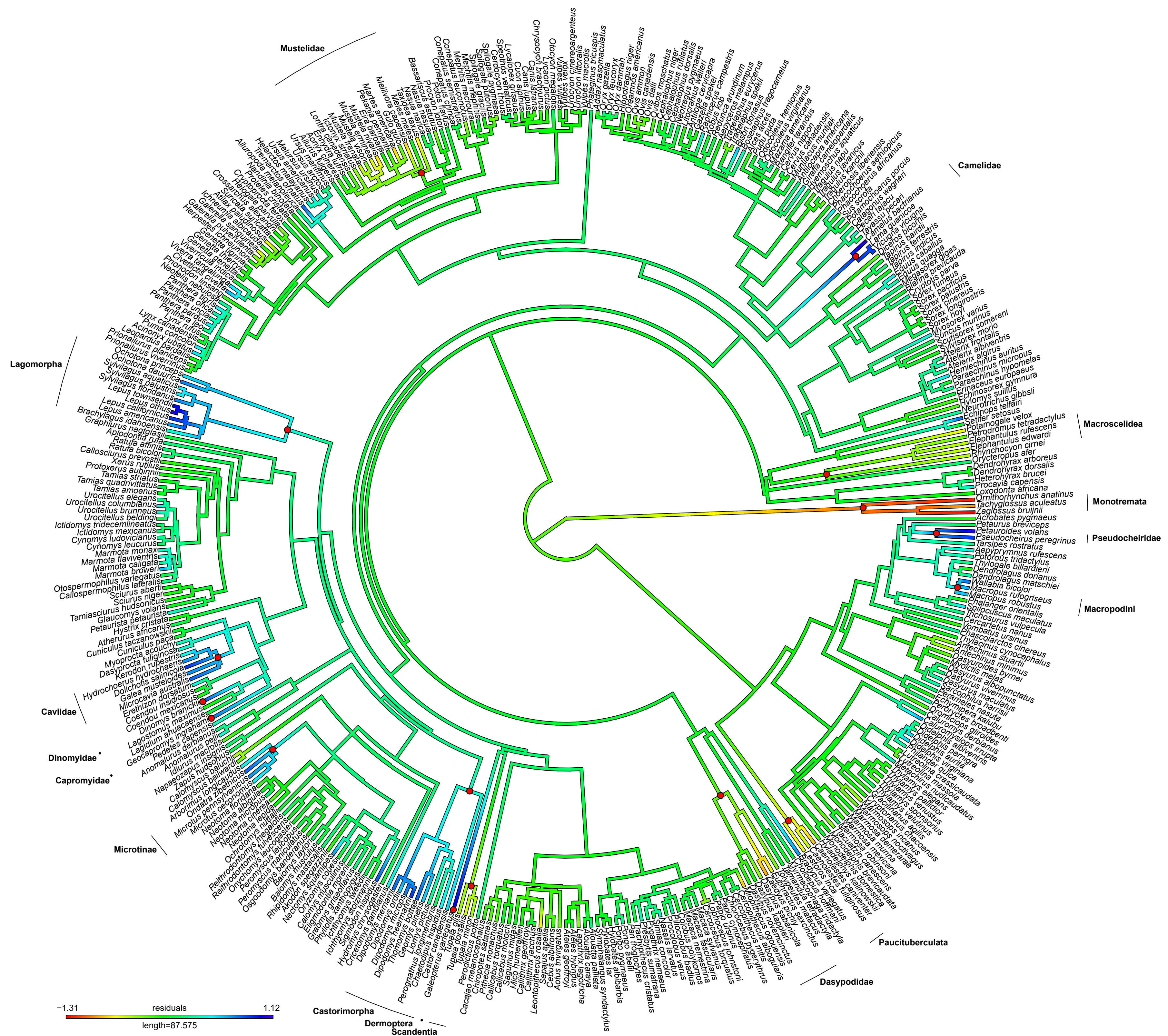

Supplement: Supplementary file 7 — Additional file 7: Figure S6. Residuals of the all-species regression equation of log OCW versus log body mass plotted onto a phylogeny of the examined taxa. Higher than expected body masses are shown in reds and yellows and lower than expected body masses are shown in cyans and blues. There is very little variation in the residuals across most of the model, suggesting a lack of Brownian motion in the evolution of this trait, but there are extreme shifts in residual values at the base of several clades such as Lagomorpha and Monotremata (.pdf). [file 12915_2021_1224_MOESM7_ESM.pdf]

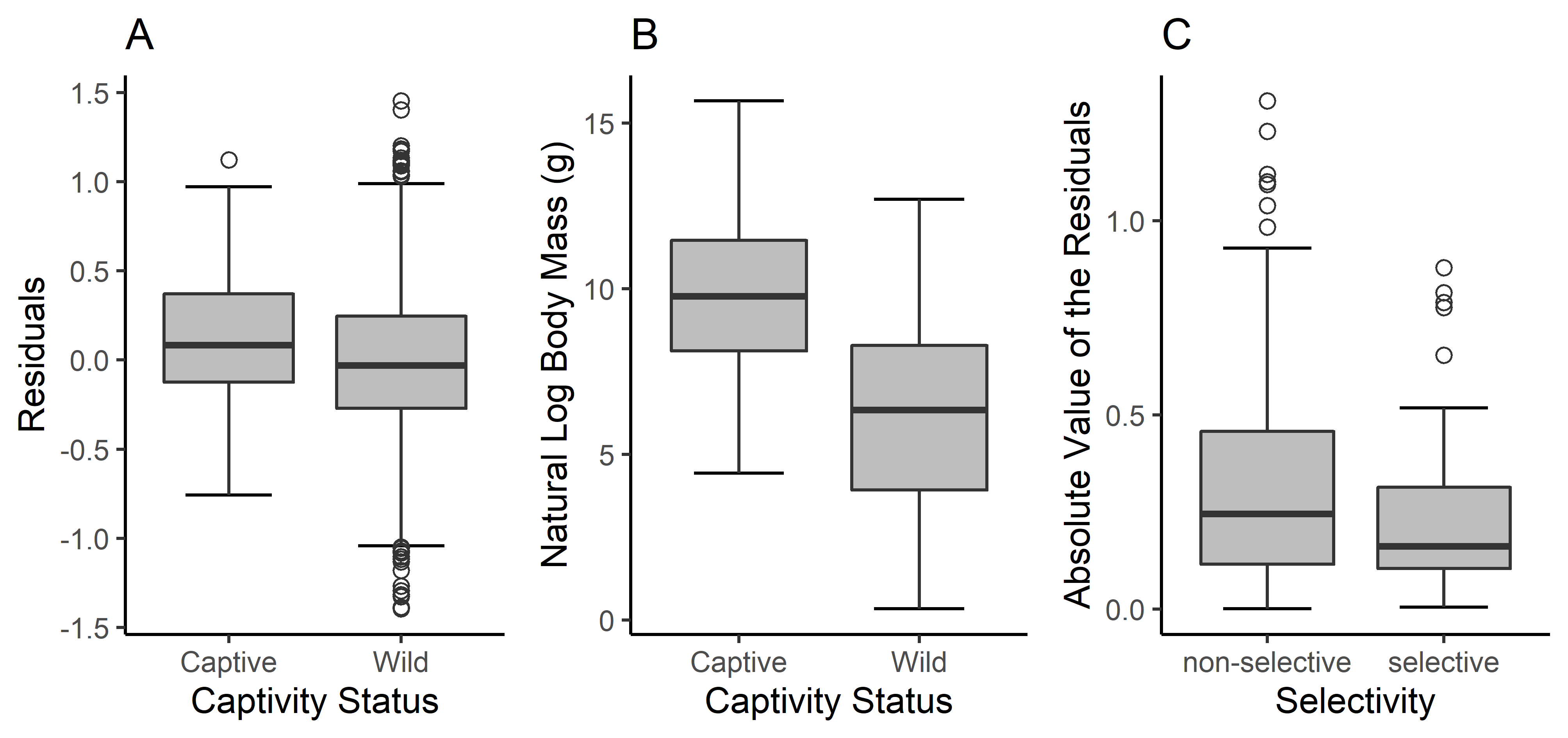

Supplement: Supplementary file 8 — Additional file 8: Figure S7. A, box plot of residuals versus captivity status for all specimens. B, box plot of residuals versus natural log of body mass (in g) for all specimens. C, box plot comparing species average residuals for species in which it was possible to be selective about what specimens were chosen versus species in which it was not possible to be selective (.tiff). [file 12915_2021_1224_MOESM8_ESM.tiff]

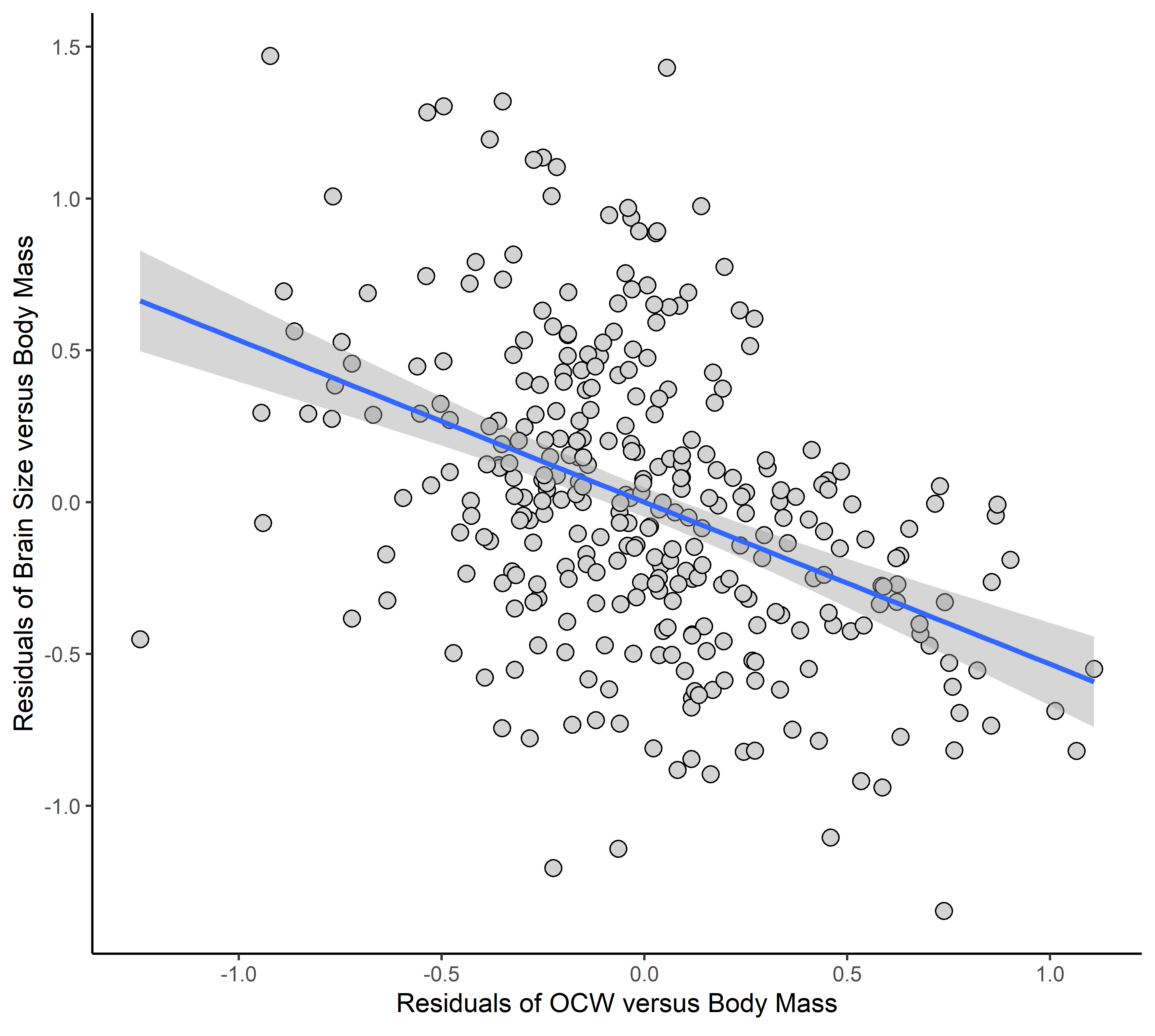

Supplement: Supplementary file 9 — Additional file 9: Figure S8. Plot of the residuals of the regression of OCW and body mass against the residuals of the regression between brain mass (scaled to the 3/4 power) and body mass, showing that the residuals in relative brain size are not strongly correlated with residuals in OCW (.tiff). [file 12915_2021_1224_MOESM9_ESM.tiff]

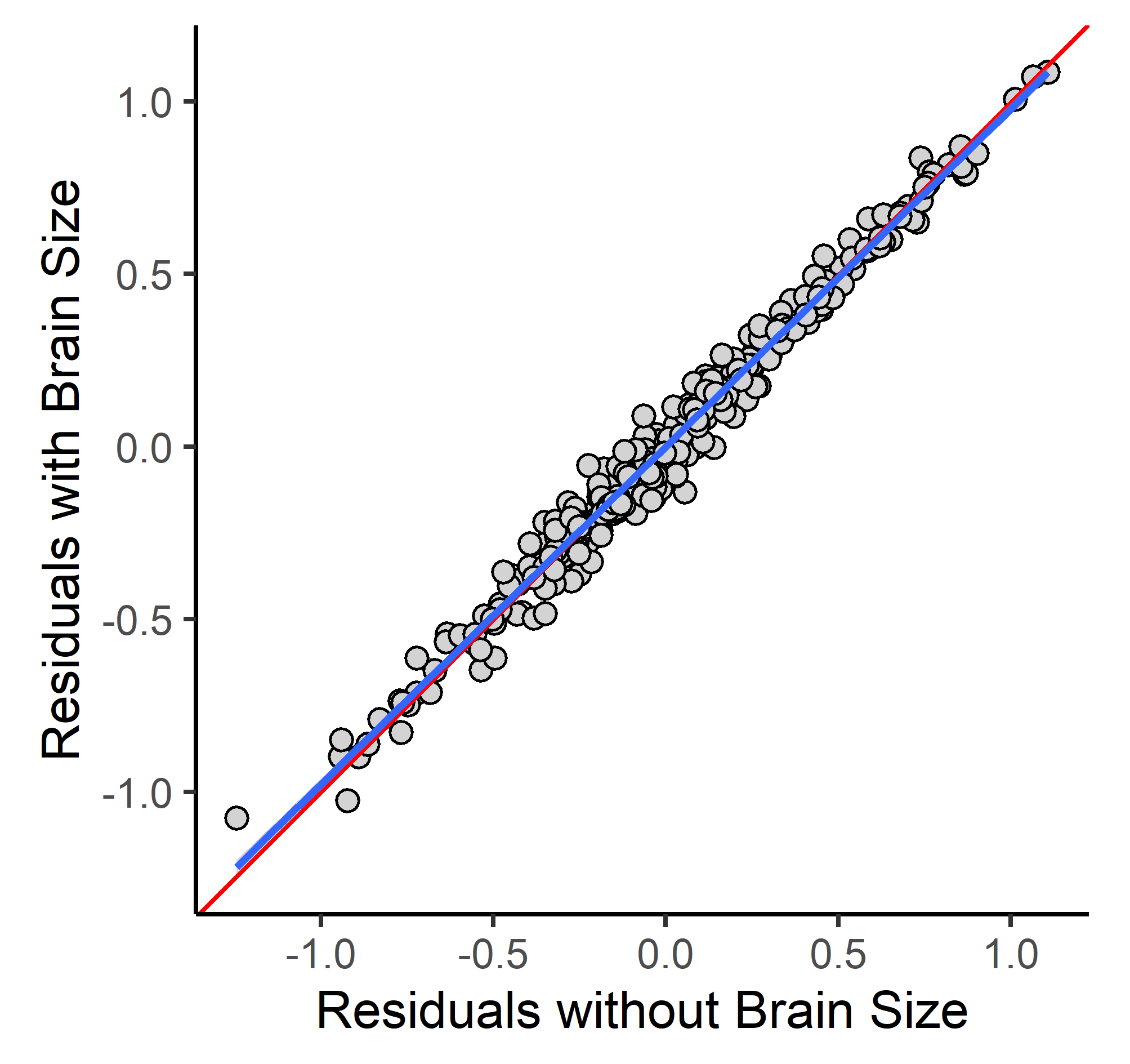

Supplement: Supplementary file 10 — Additional file 10: Figure S9. Plot of the residuals of the regression equation including brain mass as an independent variable and the residuals of the regression of the same data where brain mass is not included. Red line represents a line with intercept of 0 and slope of 1, blue line represents OLS fit. If including brain size significantly improved estimates, it would be expected that the slope would be much shallower than 1 due to residuals for extreme values being lower (.tiff). [file 12915_2021_1224_MOESM10_ESM.tiff]

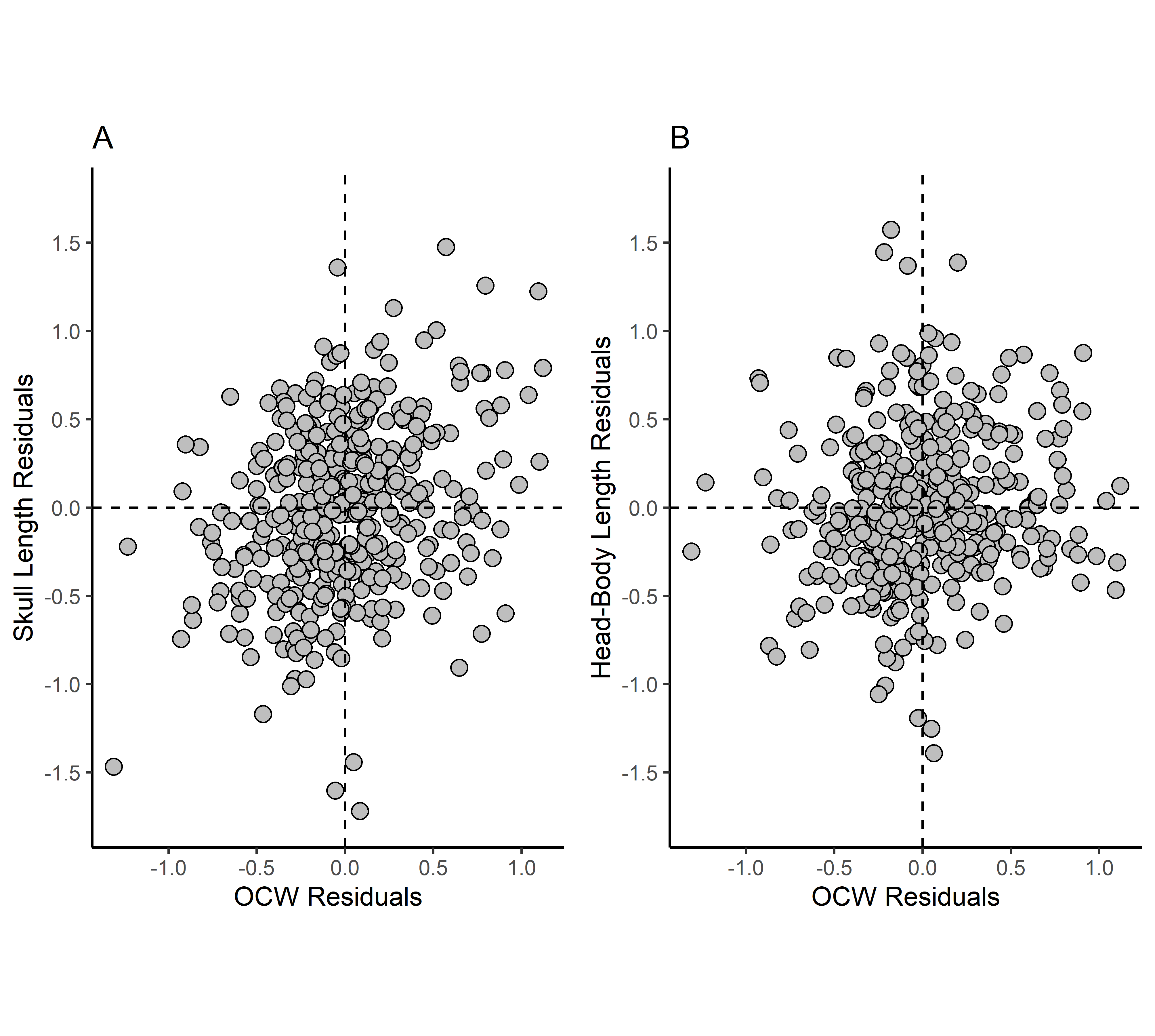

Supplement: Supplementary file 11 — Additional file 11: Figure S10. Plot of the residuals for the regression of OCW and body mass against the residuals for the regressions of head-body length (A) and skull length (B) against body mass (.tiff). [file 12915_2021_1224_MOESM11_ESM.tiff]
