# Supplementary material for: Occipital condyle width (OCW) is a highly accurate predictor of body mass in therian mammals
Source: BMC Biol. 2022 Feb 7;20:37. doi: 10.1186/s12915-021-01224-9 (PMC8883515; doi:10.1186/s12915-021-01224-9)
Supplement: Supplementary file 2 — Additional file 2. Knitted html report showing the raw results of the analyses performed in this study (.html). [file 12915_2021_1224_MOESM2_ESM.html]

Additional File 2 (R Code) for Occipital condyle width (OCW) is a highly accurate predictor of body mass in therian mammals


Code 

- Show All Code
- Hide All Code

# Additional File 2 (R Code) for Occipital condyle width (OCW) is a highly accurate predictor of body mass in therian mammals

#### *Russell Engelman*

#### *2/19/2021*

# 1 Installing R Packages

# 2 Importing and subsetting dataset

## 2.1 Importing dataset

```
b<-read.csv("tabS1_ocw_mean_data.csv",header=T) %>%
    mutate(bin=ifelse(bm >= 1000,"> 1000 g","< 1000 g"))%>%
    mutate(bin2=ifelse(bm >= 100,"> 100 g","< 100 g"))%>%
    mutate(bin3=ifelse(bm >= 10000,"> 10000 g","< 10000 g"))%>%
    mutate(superorder = case_when(
      group %in% c("Proboscidea","Afrosoricida","Hyracoidea","Tubulidentata",
                   "Macroscelidea") ~ "Afrotheria",
      group %in% c("Pilosa","Cingulata") ~ "Xenarthra",
      group %in% c("Dasyuromorphia","Didelphimorphia","Diprotodontia",
                   "Paucituberculata","Microbiotheria",
                   "Peramelemorphia") ~ "Marsupialia",
      group %in% c("Carnivora","Artiodactyla","Perissodactyla","Eulipotyphla",
                   "Pholidota") ~ "Laurasiatheria",
      group %in% c("Monotremata") ~ "Monotremata",
      group %in% c("Sciuromorpha","Primates","Castorimorpha","Anomaluromorpha",
                   "Scandentia","Hystricomorpha","Myomorpha","Lagomorpha",
                   "Dermoptera") ~ "Euarchontoglires"
    ))%>%
    mutate(species2=species) %>%
    tibble::column_to_rownames(var = "species2")
e<-read.csv("tabS2_ocw_individual_data.csv",header=T) %>%
    mutate(animal=species)
```

## 2.2 Subsetting data for necessary analyses

```
reduced<-b[b$outliertaxon!="Yes",] #Dataset excluding taxa with specialized occipital condyles
therian<-b[b$group!="Monotremata",] #Therian-only dataset, excluding monotremata
onekg<-subset(b,bm >= 1000)
```

## 2.3 Importing phylogenetic data

```
trees<-read.nexus(file="Occipital Condyles Additional File 3.nex")
```

## 2.4 Code Book

| Value | Type | Definition |
| --- | --- | --- |
| `species` | Identifier | Taxon name |
| `group` | Identifier | Higher-level taxonomic group to which the species belongs. Groups were considered at the ordinal level except for rodents, which were considered at subordinal level due to their large number of species |
| `superorder` | Identifier | Binned groups at the level of superorder (Afrotheria, Xenarthra, Laurasiatheria, Euarchontoglires, Marsupialia, and Monotremata) |
| `N` | Quantitative | Number of specimens considered |
| `ocw` | Quantitative | Bilateral width across occipital condyles measured in millimeters (see Figure 10 of manuscript) |
| `bm` | Quantitative | Average body mass of specimens measured in grams. |
| `skull_length` | Quantitative | Skull length of taxon (typically measured as condylobasal length) in millimeters. See tabS1\_ocw\_mean\_data.csv for how this value was obtained. |
| `hbl` | Quantitative | Head-body length of taxon in millimeters. See tabS1\_ocw\_mean\_data.csv for how this value was obtained. |
| `brainmass` | Quantitative | Brain mass of taxon in grams. See tabS1\_ocw\_mean\_data.csv for where this value was obtained. |
| `outliertaxon` | Categorical | Binary variable describing whether or not taxon has condyles that exhibit apomorphic specializations in some way. |
| `monotreme` | Categorical | Binary variable describing whether or not the taxon has a specialized condylar morphology similar to that seen in Monotremata (where the occipital condyles are mediolaterally divergent and do not follow the borders of the foramen magnum, see Figure 7c of manuscript) |
| `rabbit` | Categorical | Binary variable describing whether or not the taxon has a condylar morphology similar to that seen in Lagomorpha (where the occipital condyles are mediolaterally very narrow yet opisthobasally very long, creating a “pulley-shape”, see Figure 7b of manuscript) |
| `selective` | Identifier | Whether or not it was possible to be discriminatory in the selection of specimens for this species, or whether every available individual with reasonable body mass was considered. |

Table 2. Values produced by the function `regression.stats()` and their definitions.

| Value | Definition |
| --- | --- |
| `df` | degrees of freedom |
| `r2` | coefficient of determination |
| `adjr2` | adjusted coefficient of determination |
| `AIC` | Akaike information criterion |
| `BIC` | Bayesian information criterion |
| `logLik` | log-likelihood value |
| `PE` | mean percent prediction error |
| `QMLE` | quasi-maximum likelihood estimator |
| `smear` | smearing estimate |
| `RE` | ratio estimator |
| `CF` | combined correction factor as the average of `QMLE`,`smear`, and `RE` |
| `adjPE` | mean percent prediction error after applying correction factor |
| `SEE` | Percent standard error of the estimate |

# 3 Examining non-linearity of the data

## 3.1 Fitting Models

```
fit.all<-lm(log(bm)~I(log(ocw)^(2/3)),data=b)
fit.line<-lm(log(bm)~log(ocw),data=b) #linear fit
fit.power12<-lm(log(bm)~I(log(ocw)^(1/2)),data=b) #1/2 power
fit.power13<-lm(log(bm)~I(log(ocw)^(1/3)),data=b) #1/3 power
fit.power34<-lm(log(bm)~I(log(ocw)^(3/4)),data=b) #3/4 power
fit.quadratic<-lm(log(bm)~I(log(ocw)^2)+log(ocw),data=b) #quadratic regression
fit.cubic<-lm(log(bm)~I(log(ocw)^3)+I(log(ocw)^2)+log(ocw),data=b) #cubic regression
```

## 3.2 Distribution of data

```
options(scipen = 999)
grid.arrange(
ggplot(b,aes(ocw))+
  geom_histogram()+
  labs(x="Occipital Condyle Width (mm)",y="Count")+
  ggtitle("Untransformed OCW")+
  theme_classic(),
ggplot(b,aes(bm))+
  geom_histogram()+
  labs(x="Body Mass (g)",y="Count")+
  ggtitle("Untransformed BM")+
  theme_classic(),
ggplot(b,aes(log(ocw)))+
  geom_histogram()+
  labs(x="Natural Log Occipital Condyle Width (mm)",y="Count")+
  ggtitle("Natural Log OCW")+
  theme_classic(),
ggplot(b,aes(log(bm)))+
  geom_histogram()+
  labs(x="Natural Log Body Mass (g)",y="Count")+
  ggtitle("Natural Log BM")+
  theme_classic()
)
```

```
`stat_bin()` using `bins = 30`. Pick better value with `binwidth`.
`stat_bin()` using `bins = 30`. Pick better value with `binwidth`.
`stat_bin()` using `bins = 30`. Pick better value with `binwidth`.
`stat_bin()` using `bins = 30`. Pick better value with `binwidth`.
```

Distributions of raw and natural log-transformed OCW and body mass, showing the strongly log-distributed distributions of each.

## 3.3 Box-cox plots

```
par(mfrow=c(2,2))
boxcox(lm(bm~ocw,data=b))
title(main="Untransformed Data")
boxcox(fit.line)
title(main="Log-Linear Model")
boxcox(fit.all)
title(main="Log-2/3 Power Model")
boxcox(fit.quadratic)
title(main="Log-Quadratic Model")
```

Box-cox plots for several regression models, including raw data, log-linear model, log-quadratic model, and a log-2/3 power model (the latter being the preferred model here)

```
data.frame("lambda"=boxcox(fit.line,plotit=FALSE)$x,"log-likelihood"=boxcox(fit.line,plotit=FALSE)$y)
```

## 3.4 Scatterplot of log-transformed data

```
fit.line<-lm(log(bm)~log(ocw),data=b) #linear fit
plot_data<-data.frame(b,predict(fit.line,interval="prediction"),
                      predict(fit.all,interval="prediction"))
lm_eqn.all <- function(plot_data){
    m <- lm(I(log(bm)) ~ I(log(ocw)^(2/3)), plot_data);
    eq <- substitute(italic("ln(body mass)") == a + b %.% italic("ln(OCW)")^{2/3}*","~~italic(r)^2~"="~r2, 
                     list(a = format(unname(coef(m)[1]), digits = 5),
                          b = format(unname(coef(m)[2]), digits = 5),
                          r2 = format(summary(m)$r.squared, digits = 3)))
    as.character(as.expression(eq));
}
(scatterplot.total<-ggplot(plot_data,aes(log(ocw),log(bm)))+
    geom_point(size=3,shape=21,col="black",fill="light gray")+
    labs(x= expression(paste("Natural Log OCW (mm)")),
         y="Natural Log Body Mass (g)")+
    scale_x_continuous(breaks=seq(1,6,0.5))+
    scale_y_continuous(breaks=seq(0,18,1))+
    geom_smooth(method="lm",aes(color="red"),formula=y~x,level=0.9)+
    geom_smooth(aes(x=log(ocw),y=log(bm),color="blue"),
                method="lm",formula=y~I(x^(2/3)),level=0.9)+
    geom_line(aes(y=lwr), color = "red", linetype = "dashed")+
    geom_line(aes(y=upr), color = "red", linetype = "dashed")+
    geom_line(aes(y=lwr.1), color = "blue", linetype = "dashed",size=0.6)+
    geom_line(aes(y=upr.1), color = "blue", linetype = "dashed",size=0.6)+
    geom_text(aes(x = 1.2, y = 17.5, label = lm_eqn.all(plot_data)),hjust=0, parse = TRUE,data.frame())+
    scale_color_identity(name="Regression",
                         labels=c(red="Linear",blue="2/3 Power"),
                         guide="legend")+
    theme_classic()+
    theme(legend.position=c(.8,.2)))
```

Figure 2. Scatterplot of natural log of OCW versus natural log of body mass, showing the best fit (log OCW raised to the 2/3 power) regression line for all species and the non-linear distribution of the data. Linear regression is in red and 2/3 power regression is in blue. Dashed lines represent 95% prediction intervals. Most of the species located above the upper bounds of the prediction interval are lagomorphs.

## 3.5 Akaike Information Criterion and accuracy statistics for various alternative regression models

```
data.frame(rbind("Linear model"=regression.stats(fit.line),
                 "1/2 power model"=regression.stats(fit.power12),
                 "1/3 power model"=regression.stats(fit.power13),
                 "2/3 power model"=regression.stats(fit.all),
                 "3/4 power model"=regression.stats(fit.power34),
                 "Quadratic model"=regression.stats(fit.quadratic),
                 "Cubic model"=regression.stats(fit.cubic)))
```

## 3.6 Plot of Ln OCW versus Ln body mass showing non-linearity

```
plot_data.quadratic<-data.frame(b,predict(fit.line,interval="prediction"),
                                predict(fit.quadratic,interval="prediction"),
                                predict(fit.all,interval="prediction"))
(scatterplot.quadratic<-ggplot(plot_data.quadratic,aes(log(ocw),log(bm)))+
        geom_point(size=3,shape=21,col="black",fill="light gray")+
        labs(x="Natural Log OCW (mm)",
             y="Natural Log Body Mass (g)")+
        scale_x_continuous(breaks=seq(1,10,0.5))+
        scale_y_continuous(breaks=seq(0,18,1))+
        geom_smooth(method="lm",aes(color="red"),formula=y~x,level=0.9)+
        geom_smooth(aes(x=log(ocw),y=log(bm),color="green"),
                    method="lm",formula=y~x+I(x^2),level=0.9)+
        geom_smooth(aes(x=log(ocw),y=log(bm),color="blue"),
                    method="lm",formula=y~I(x^(2/3)),linetype="dashed",level=0.9)+
        geom_line(aes(y=lwr), color = "red", linetype = "dashed")+
        geom_line(aes(y=upr), color = "red", linetype = "dashed")+
        geom_line(aes(y=lwr.2), color = "blue", linetype = "dashed")+
        geom_line(aes(y=upr.2), color = "blue", linetype = "dashed")+
        geom_line(aes(y=lwr.1), color = "green", linetype = "dashed")+
        geom_line(aes(y=upr.1), color = "green", linetype = "dashed")+
        theme_classic()+
        scale_color_identity(name="Regression",labels=c(red="Linear",blue=                              "2/3 Power",green="Quadratic"), guide="legend")+
        theme(legend.position=c(.8,.2)))
```

Figure S2. Scatterplot of natural log of OCW versus natural log of body mass, comparing the best fit curve between a linear (in red), 2/3 power (in blue) and quadratic model (in green). Dashed lines represent the 95% prediction intervals.

## 3.7 Residuals vs. Fitted Graphs

```
fit.line<-lm(log(bm)~log(ocw),data=b)
fit.skull_length.line<-lm(log(bm)~log(skull_length),data=b)
fit.skull_length<-lm(log(bm)~I(log(skull_length)^(1/2)),data=b)
invisible(resfit1<-autoplot(fit.line,which=1,label=FALSE,
                  fill="light gray",shape=21,ncol=1)+
                  labs(x="Fitted Values")+
                  ggtitle("A")+
                  theme_classic())
invisible(resfit2<-autoplot(fit.all,which=1,label=FALSE,
                  fill="light gray",shape=21,ncol=1)+
                  labs(x="Fitted Values")+
                  ggtitle("B")+
                  theme_classic())
invisible(resfit3<-autoplot(fit.skull_length.line,which=1,label=FALSE,
                  fill="light gray",shape=21,ncol=1)+
                  labs(x="Fitted Values")+
                  ggtitle("C")+
                  theme_classic())
invisible(resfit4<-autoplot(fit.skull_length,which=1,label=FALSE,
                  fill="light gray",shape=21,ncol=1)+
                  labs(x="Fitted Values")+
                  ggtitle("D")+
                  theme_classic())
resfitfigure<-grid.arrange(resfit1[[1]],resfit2[[1]],resfit3[[1]],resfit4[[1]],nrow=2)
```

Figure 3. Residuals versus fitted plot for the regression of OCW (**A**-**B**) or skull length (**C**-**D**) against body mass. **A** and **C** represent residuals versus fitted graphs for regression lines where isometry is assumed, and **B** and **D** represent graphs with the natural log of the independent variable raised to the 2/3 power (in **B**) or the 1/2 power (in **D**).

## 3.8 Regression plot of other variables and untransformed variables

```
varplot1<-ggplot(b,aes(x=log(ocw),y=log(bm)))+
        geom_point(size=2,shape=21,col="black",fill="light gray")+
        labs(x= expression(paste("Natural Log OCW (mm)")),
             y="Natural Log Body Mass (g)")+
        scale_x_continuous(breaks=seq(1,17,0.5))+
        scale_y_continuous(breaks=seq(0,17,1))+
        geom_smooth(method="loess",formula=y~x,col="red",linetype="dashed"                     )+
        geom_smooth(method="lm",formula=y~x)+
        ggtitle("A")+
        theme_classic()
varplot2<-ggplot(b,aes(x=log(ocw)^(2/3),y=log(bm)))+
        geom_point(size=2,shape=21,col="black",fill="light gray")+
        labs(x= expression(paste("Natural Log OCW (mm)"^{2/3})),
             y="Natural Log Body Mass (g)")+
        scale_x_continuous(breaks=seq(1,17,0.25))+
        scale_y_continuous(breaks=seq(0,17,1))+
        geom_smooth(method="lm",formula=y~x)+
        ggtitle("B")+
        theme_classic()
varplot3<-ggplot(b,aes(x=log(skull_length),y=log(bm)))+
        geom_point(size=2,shape=21,col="black",fill="light gray")+
        labs(x= expression(paste("Natural Log Skull Length (mm)")),
             y="Natural Log Body Mass (g)")+
        scale_x_continuous(breaks=seq(1,17,0.5))+
        scale_y_continuous(breaks=seq(0,17,1))+
        geom_smooth(method="loess",formula=y~x,col="red",linetype="dashed")+
        geom_smooth(method="lm",formula=y~x)+
        ggtitle("C")+
        theme_classic()
varplot4<-ggplot(b,aes(x=log(skull_length)^(1/2),y=log(bm)))+
        geom_point(size=2,shape=21,col="black",fill="light gray")+
        labs(x= expression(paste("Natural Log Skull Length (mm)"^{1/2})),
             y="Natural Log Body Mass (g)")+
        scale_x_continuous(breaks=seq(1,3.25,0.25))+
        scale_y_continuous(breaks=seq(0,17,1))+
        geom_smooth(method="lm",formula=y~x)+
        ggtitle("D")+
        theme_classic()
varplot5<-ggplot(b,aes(x=log(hbl),y=log(bm)))+
        geom_point(size=2,shape=21,col="black",fill="light gray")+
        labs(x= expression(paste("Natural Log HBL (mm)")),
             y="Natural Log Body Mass (g)")+
        scale_x_continuous(breaks=seq(1,17,0.5))+
        scale_y_continuous(breaks=seq(0,17,1))+
        geom_smooth(method="lm",formula=y~x)+
        ggtitle("E")+
        theme_classic()
varplots<-grid.arrange(varplot1,varplot2,varplot3,varplot4,varplot5,ncol=2,nrow=3)
```

Figure S1. Plots of variables examined versus body mass. **A**, OCW versus body mass assuming isometry. **B**, OCW versus body mass with OCW raised to the 2/3 power. **C**, skull length versus body mass assuming isometry. **D**, skull length versus body mass raised to the 1/2 power. **E**, HBL versus body mass. No power transformation for HBL is included as the non-linear fit indicates that the relationship between natural log HBL and natural log body mass is linear. For **A** and **C**, blue lines are linear regression lines and red dashed lines are loess fit lines. Note how the linear regression lines in **A** and **C** do not precisely follow the trend of the data, overestimating body mass at the extremes and underestimating it in the middle ranges of the data set.

## 3.9 Examining effects of non-linearity on predicting OCW and body mass

```
fit.bm_to_ocw<-lm(log(ocw)~log(bm),data=b)
b%>%
  filter(species %in% c("Loxodonta_africana","Ursus_maritimus",
                        "Mephitis_mephitis","Coendou_insidiosus",
                        "Sorex_longirostris"))%>%
  mutate(pred_bm=exp(predict(fit.line,newdata=.)))%>%
  mutate(pred_ocw=exp(predict(fit.bm_to_ocw,newdata=.)))%>%
  mutate(PE_ocw=(pred_ocw*regression.stats.ocw(fit.bm_to_ocw)$CF-ocw)/(pred_ocw*regression.stats.ocw(fit.bm_to_ocw)$CF))%>%
  mutate(PE_bm=(pred_bm*regression.stats(fit.line)$CF-bm)/(pred_bm*regression.stats(fit.line)$CF))%>%
  mutate(across(c(pred_ocw,PE_ocw,pred_bm,PE_bm),round,2))%>%
  select(N,ocw,pred_ocw,PE_ocw,bm,pred_bm,PE_bm)%>%
  arrange(bm)%>%
  kable(col.names=c("N","Actual","Predicted","%PEcf","Actual","Predicted","%PEcf"),caption="Effects of non-linearity on attempting to estimate OCW and body mass using a log-linear model. <i>Loxodonta africana</i> is the largest taxon in the dataset, <i>Ursus maritimus</i> is the largest taxon for which N > 2, <i>Sorex longrostris</i> is the smallest taxon in the dataset, and <i>Coendou insidiosus</i> and <i>Mephitis mephitis</i> are the two median values for the dataset.",table.attr = "style='width:100%;'")%>%
  add_header_above(c(" "=2,"OCW (mm)"=3,"Body Mass (g)"=3))
```

Effects of non-linearity on attempting to estimate OCW and body mass using a log-linear model. *Loxodonta africana* is the largest taxon in the dataset, *Ursus maritimus* is the largest taxon for which N > 2, *Sorex longrostris* is the smallest taxon in the dataset, and *Coendou insidiosus* and *Mephitis mephitis* are the two median values for the dataset.

|  | | OCW (mm) | | | Body Mass (g) | | |
| --- | --- | --- | --- | --- | --- | --- | --- |
|  | N | Actual | Predicted | %PEcf | Actual | Predicted | %PEcf |
| Sorex\_longirostris | 11 | 3.92 | 3.38 | -0.15 | 3.05 | 5.96 | 0.43 |
| Mephitis\_mephitis | 6 | 17.66 | 18.57 | 0.06 | 1641.40 | 1365.23 | -0.33 |
| Coendou\_insidiosus | 1 | 18.50 | 18.65 | 0.02 | 1666.00 | 1614.56 | -0.14 |
| Ursus\_maritimus | 4 | 78.44 | 72.49 | -0.07 | 249476.00 | 296931.98 | 0.07 |
| Loxodonta\_africana | 1 | 251.60 | 174.95 | -0.42 | 6435000.00 | 19942974.54 | 0.64 |

## 3.10 Non-linear curve fitting for OCW, HBL, and skull length

### 3.10.1 Curve fit for OCW, all species

```
nls(log(bm)~a*log(ocw)^b+c,data=b,start=list(a=1,b=1,c=1))
```

```
Nonlinear regression model
  model: log(bm) ~ a * log(ocw)^b + c
   data: b
      a       b       c 
 7.2885  0.6882 -7.7239 
 residual sum-of-squares: 61.07

Number of iterations to convergence: 5 
Achieved convergence tolerance: 0.000001146
```

```
confint2(nls(log(bm)~a*log(ocw)^b+c,data=b,start=list(a=1,b=1,c=1)))
```

```
       2.5 %     97.5 %
a  5.7769069  8.8000144
b  0.6050588  0.7712713
c -9.5009661 -5.9468553
```

### 3.10.2 Curve fit for OCW, therians only

```
nls(log(bm)~a*log(ocw)^b+c,data=therian,start=list(a=1,b=1,c=1))
```

```
Nonlinear regression model
  model: log(bm) ~ a * log(ocw)^b + c
   data: therian
      a       b       c 
 7.4696  0.6791 -7.9389 
 residual sum-of-squares: 56.96

Number of iterations to convergence: 5 
Achieved convergence tolerance: 0.000001401
```

```
confint2(nls(log(bm)~a*log(ocw)^b+c,data=therian,start=list(a=1,b=1,c=1)))
```

```
       2.5 %     97.5 %
a  5.9578746  8.9813993
b  0.5985936  0.7595757
c -9.7094717 -6.1684156
```

### 3.10.3 Curve fit for OCW, reduced dataset

```
nls(log(bm)~a*log(ocw)^b+c,data=reduced,start=list(a=1,b=1,c=1))
```

```
Nonlinear regression model
  model: log(bm) ~ a * log(ocw)^b + c
   data: reduced
      a       b       c 
 7.2531  0.6939 -7.7665 
 residual sum-of-squares: 43.17

Number of iterations to convergence: 5 
Achieved convergence tolerance: 0.0000001759
```

```
confint2(nls(log(bm)~a*log(ocw)^b+c,data=reduced,start=list(a=1,b=1,c=1)))
```

```
       2.5 %     97.5 %
a  5.9312401  8.5750108
b  0.6205828  0.7672006
c -9.3243368 -6.2085802
```

### 3.10.4 Curve fit for condylobasal length of skull

```
nls(log(bm)~a*log(skull_length)^b+c,data=b,start=list(a=1,b=1,c=1))
```

```
Nonlinear regression model
  model: log(bm) ~ a * log(skull_length)^b + c
   data: b
       a        b        c 
 18.0171   0.4346 -26.8848 
 residual sum-of-squares: 89.36

Number of iterations to convergence: 13 
Achieved convergence tolerance: 0.000002423
```

```
confint2(nls(log(bm)~a*log(skull_length)^b+c,data=b,start=list(a=1,b=1,c=1)))
```

```
        2.5 %      97.5 %
a   8.0589419  27.9753081
b   0.2881211   0.5811162
c -38.3664132 -15.4032713
```

### 3.10.5 Curve fit for HBL

```
nls(log(bm)~a*log(hbl)^b+c,data=b,start=list(a=1,b=1,c=1))
```

```
Nonlinear regression model
  model: log(bm) ~ a * log(hbl)^b + c
   data: b
       a        b        c 
  3.7468   0.9181 -11.8832 
 residual sum-of-squares: 72.31

Number of iterations to convergence: 5 
Achieved convergence tolerance: 0.0000000821
```

```
confint2(nls(log(bm)~a*log(hbl)^b+c,data=b,start=list(a=1,b=1,c=1)))
```

```
        2.5 %    97.5 %
a   2.0363617  5.457162
b   0.7585989  1.077585
c -15.1701928 -8.596144
```

### 3.10.6 Curve fit for OCW versus skull length

```
nls(log(skull_length)~a*log(ocw)^b+c,data=b,start=list(a=1,b=1,c=1))
```

```
Nonlinear regression model
  model: log(skull_length) ~ a * log(ocw)^b + c
   data: b
    a     b     c 
1.001 1.020 1.446 
 residual sum-of-squares: 9.425

Number of iterations to convergence: 3 
Achieved convergence tolerance: 0.0000001503
```

```
confint2(nls(log(skull_length)~a*log(ocw)^b+c,data=b,start=list(a=1,b=1,c=1)))
```

```
      2.5 %   97.5 %
a 0.7676791 1.234395
b 0.9067236 1.133857
c 1.1229315 1.769534
```

### 3.10.7 Curve fit for OCW versus HBL

```
nls(log(hbl)~a*log(ocw)^b+c,data=b,start=list(a=1,b=1,c=1))
```

```
Nonlinear regression model
  model: log(hbl) ~ a * log(ocw)^b + c
   data: b
     a      b      c 
2.6309 0.6519 0.7132 
 residual sum-of-squares: 12.99

Number of iterations to convergence: 5 
Achieved convergence tolerance: 0.000001319
```

```
confint2(nls(log(hbl)~a*log(ocw)^b+c,data=b,start=list(a=1,b=1,c=1)))
```

```
       2.5 %    97.5 %
a  1.8435237 3.4182950
b  0.5356020 0.7682442
c -0.1986879 1.6249989
```

# 4 Analysis using species-average data for all species

```
par(mfrow=c(2,2))
plot(fit.all)
```

```
summary(fit.all)
```

```
Call:
lm(formula = log(bm) ~ I(log(ocw)^(2/3)), data = b)

Residuals:
     Min       1Q   Median       3Q      Max 
-1.30974 -0.23437 -0.02249  0.20268  1.12010 

Coefficients:
                  Estimate Std. Error t value            Pr(>|t|)    
(Intercept)       -8.19502    0.10904  -75.16 <0.0000000000000002 ***
I(log(ocw)^(2/3))  7.69289    0.05332  144.27 <0.0000000000000002 ***
---
Signif. codes:  0 '***' 0.001 '**' 0.01 '*' 0.05 '.' 0.1 ' ' 1

Residual standard error: 0.3899 on 402 degrees of freedom
Multiple R-squared:  0.9811,    Adjusted R-squared:  0.981 
F-statistic: 2.081e+04 on 1 and 402 DF,  p-value: < 0.00000000000000022
```

## 4.1 Average residuals by order (OCW)

```
datatable(b %>%
  mutate(residuals=fit.all$residuals) %>%
  mutate(PEcf=abs((exp(fit.all$fitted.values)*regression.stats(fit.all)$CF)-b$bm)/
           (exp(fit.all$fitted.values)*regression.stats(fit.all)$CF))%>%
  group_by(group) %>%
  summarise(residuals=round(mean(residuals),4),
            "%PEcf"=round(mean(PEcf)*100,2),
            N=n()))
```

## 4.2 Predicted body mass and error of CF-adjusted predicted values

Prediction error is in percent.

```
invisible(fitted.values<-data.frame("Actual BM"=round(b$bm,2),
                     "Predicted BM"=round((exp(fit.all$fitted.values)*regression.stats(fit.all)$CF),2),
                     "Percent PE"=round(((exp(fit.all$fitted.values)*regression.stats(fit.all)$CF)-b$bm)/(exp(fit.all$fitted.values)*regression.stats(fit.all)$CF),4)*100,
                     Order=b$group))
datatable(fitted.values)
```

## 4.3 Normality tests of residuals

```
bptest(fit.all) #Test for heteroskedasticity
```

```
    studentized Breusch-Pagan test

data:  fit.all
BP = 0.13618, df = 1, p-value = 0.7121
```

```
shapiro.test(fit.all$residuals) #Test for normality
```

```
    Shapiro-Wilk normality test

data:  fit.all$residuals
W = 0.98739, p-value = 0.00142
```

```
kurtosis(fit.all$residuals) #Kurtosis
```

```
[1] 0.5319824
```

```
skewness(fit.all$residuals) #Skewness
```

```
[1] 0.1925011
```

## 4.4 Histogram of residuals and Q-Q plot of species-average data

```
invisible(ocwhistogram<-ggplot(data=fit.all,aes(fit.all$residuals))+
        geom_histogram(binwidth=0.1,color="black",fill="white")+
        geom_vline(aes(xintercept=mean(fit.all$residuals)),
                   color="black", linetype="dashed", size=0.5)+
        stat_function(fun = function(x) dnorm(x, mean = mean(fit.all$residuals), sd = sd(fit.all$residuals)) * nrow(b) * 0.1,
                      color = "red", size = 0.5)+
        labs(x= expression(paste("Residuals")),y="Counts")+
        ggtitle("A")+
        theme_classic())
#Better Q-Q plot of data
invisible(QQocw<-autoplot(fit.all,which=2,ncol=1,label=FALSE,fill="light gray",shape=21,size=2)+
        ggtitle("B")+
        labs(y="Standardized Residuals")+
        theme_classic())
QQhist<-grid.arrange(ocwhistogram,QQocw[[1]],nrow=1)
```

Figure 5. Histogram (**A**) and Q-Q plot (**B**) of the residuals of the total species regression analysis, showing the approximately normal distribution of the residuals.

## 4.5 Accuracy statistics

```
regression.stats(fit.all)
```

```
median(abs(exp(fit.all$fitted.values)-b$bm)/exp(fit.all$fitted.values)) #Median %PE for data
```

```
[1] 0.2172943
```

**Percent of taxa within 20% of actual value**

```
sum(abs((exp(fit.all$fitted.values)*regression.stats(fit.all)$CF-b$bm)/b$bm)<0.2)/length(b$bm)
```

```
[1] 0.4158416
```

**Percent of taxa within 50% of actual value**

```
sum(abs((exp(fit.all$fitted.values)*regression.stats(fit.all)$CF-b$bm)/b$bm)<0.5)/length(b$bm)
```

```
[1] 0.8143564
```

## 4.6 Test of whether selectivity of specimens affects accuracy

```
t.test(fit.all$residuals~b$selective) #T-test of residuals between groups in which it was possible to be selective about which individuals were chosen.
```

```
    Welch Two Sample t-test

data:  fit.all$residuals by b$selective
t = -1.9007, df = 176.04, p-value = 0.05898
alternative hypothesis: true difference in means between group non-selective and group selective is not equal to 0
95 percent confidence interval:
 -0.153223171  0.002881386
sample estimates:
mean in group non-selective     mean in group selective 
                -0.01562959                  0.05954130
```

```
ggplot()+
        aes(b$selective,fit.all$residuals)+
        stat_boxplot(geom="errorbar",width=0.5)+
        geom_boxplot(fill="gray",outlier.shape=1,outlier.size=2)+
        labs(x="Selectivity",y="Residuals")+
        theme_classic()
```

Boxplot of residuals between taxa for which it was possible to be selective about specimens shown and those in which it was not. Not included in supplementary information but included here to show that the median value did not differ between the two categories before taking the absolute value.

```
t.test(abs(fit.all$residuals)~b$selective) #T-test of the absolute value of the residuals between groups in which it was possible to be selective about which individuals were chosen.
```

```
    Welch Two Sample t-test

data:  abs(fit.all$residuals) by b$selective
t = 3.3755, df = 168.51, p-value = 0.0009147
alternative hypothesis: true difference in means between group non-selective and group selective is not equal to 0
95 percent confidence interval:
 0.03637976 0.13888006
sample estimates:
mean in group non-selective     mean in group selective 
                  0.3149704                   0.2273405
```

```
(boxplot5<-ggplot()+
        aes(b$selective,abs(fit.all$residuals))+
        stat_boxplot(geom="errorbar",width=0.5)+
        geom_boxplot(fill="gray",outlier.shape=1,outlier.size=2)+
        labs(x="Selectivity",y="Absolute Value of the Residuals")+
        ggtitle("C")+
        theme_classic())
```

Figure S7c. Additional box plots of various parameters. **C**, box plot comparing species average residuals for species in which it was possible to be selective about what specimens were chosen versus species in which it was not possible to be selective. See Figure S7a-b for remaining plots shown in supplementary figure.

## 4.7 Plot of Residuals versus Sample Size

```
residdata<-data.frame(species=b$species,N=b$N,absresid=abs(fit.all$residuals)) %>%
    mutate(bin=cut_width(N, width=2, boundary=0))
residN<-lm(absresid~N,data=residdata)
summary(residN)
```

```
Call:
lm(formula = absresid ~ N, data = residdata)

Residuals:
     Min       1Q   Median       3Q      Max 
-0.31506 -0.19142 -0.07058  0.12326  0.98598 

Coefficients:
             Estimate Std. Error t value            Pr(>|t|)    
(Intercept)  0.330089   0.020748  15.910 <0.0000000000000002 ***
N           -0.006332   0.003149  -2.011               0.045 *  
---
Signif. codes:  0 '***' 0.001 '**' 0.01 '*' 0.05 '.' 0.1 ' ' 1

Residual standard error: 0.2508 on 402 degrees of freedom
Multiple R-squared:  0.009961,  Adjusted R-squared:  0.007498 
F-statistic: 4.044 on 1 and 402 DF,  p-value: 0.04498
```

```
par(mfrow=c(2,2))
plot(residN)
```

```
lm(absresid~N,data=residdata %>%filter(species!="Canis_latrans"))%>%summary() #Regression of absolute value of the residuals versus sample size minus Canis latrans
```

```
Call:
lm(formula = absresid ~ N, data = residdata %>% filter(species != 
    "Canis_latrans"))

Residuals:
     Min       1Q   Median       3Q      Max 
-0.31675 -0.19250 -0.07266  0.12329  0.98429 

Coefficients:
             Estimate Std. Error t value            Pr(>|t|)    
(Intercept)  0.332255   0.021161  15.702 <0.0000000000000002 ***
N           -0.006809   0.003276  -2.078              0.0383 *  
---
Signif. codes:  0 '***' 0.001 '**' 0.01 '*' 0.05 '.' 0.1 ' ' 1

Residual standard error: 0.251 on 401 degrees of freedom
Multiple R-squared:  0.01066,   Adjusted R-squared:  0.00819 
F-statistic:  4.32 on 1 and 401 DF,  p-value: 0.03831
```

```
invisible(samplesize<-ggplot(residdata,aes(N,absresid))+
        geom_jitter(size=3,shape=21,col="black",fill="light gray",width=0.3,height=0)+
        labs(x="Number of Specimens",y="Absolute Value of the Residuals")+
        scale_x_continuous(breaks=seq(0,16,5),limits=c(0,16))+
        scale_y_continuous(breaks=seq(0,1.25,.25))+
        ggtitle("A")+
        geom_smooth(formula=y~x,method="lm",level=0.9)+
        theme_classic()+
        geom_text(aes(x = 12, y = c(1.25,1.13,1.0),
                      label = c(paste("N == ",
                      nrow(b)),paste("r^{2} == "
                                     ,round(summary(residN)$adj.r.squared,4)),
paste("p == ",round(summary(residN)$coefficients[2,4],4)))),hjust=0,
                  parse = TRUE,data.frame()))
invisible(boxplot2<-ggplot(residdata,aes(bin,absresid))+
        geom_boxplot()+
        labs(x="Number of Specimens",y="Absolute Value of the Residuals")+
        scale_y_continuous(breaks=seq(0,1.25,.25))+
        scale_x_discrete(labels=c("1-2","3-4","5-6","7-8","9-10","11-12","13-14","15-16","27-28"))+
        ggtitle("B")+
        theme_classic())
grid.arrange(samplesize,boxplot2,ncol=2)
```

Figure S3. Scatter plot (**A**) and boxplot (**B**) of sample size versus absolute value of the residuals. *Canis latrans* (N=27) is omitted from **A** to better show the decrease in maximal residuals as sample size increases.

# 5 Examining interspecific differences in occiput shape

## 5.1 Scatterplot color-coded by occiput morphology type

```
plot_rabbit<-data.frame(b,predict(fit.all,interval="prediction"))%>%
    mutate(group2="Other")%>%
    mutate(group2=replace(group2,rabbit=="Yes","Rabbit-Like Occiput"))%>%
    mutate(group2=replace(group2,group=="Monotremata","Monotremata"))%>%
    mutate(group2=replace(group2,group=="Cingulata","Cingulata"))
lm_eqn <- function(plot_rabbit){
    m <- lm(I(log(bm)) ~ I(log(ocw)^(2/3)), data=subset(plot_rabbit,group2=='Rabbit-Like Occiput'));
    eq <- substitute(italic("ln(body mass)") == a + b %.% italic("ln(OCW)")^{2/3}*","~~italic(r)^2~"="~r2, 
                     list(a = format(unname(coef(m)[1]), digits = 5),
                          b = format(unname(coef(m)[2]), digits = 5),
                          r2 = format(summary(m)$r.squared, digits = 3)))
    as.character(as.expression(eq));
}    
(scatterplot.groups<-ggplot(plot_rabbit,aes(log(ocw)^(2/3),log(bm)))+
    geom_point(size=3,col="black",aes(fill=group2,shape=group2))+
    geom_point(data=subset(plot_rabbit,group2!='Other'),size=3,col="black"                ,aes(fill=group2,shape=group2))+
    geom_smooth(data=plot_rabbit %>%
                  filter(group2=="Other"|group2=='Rabbit-Like Occiput'), 
                aes(group=group2), color="transparent", formula=y~x, method="lm",
                level=0.9)+
    geom_smooth(aes(group=group2,color=group2),
               formula=y~x,method="lm",se=F,level=0.9)+
    labs(x= expression(paste("Natural Log OCW (mm)"^{2/3})),
         y="Natural Log Body Mass (g)")+
    theme_classic()+
    scale_x_continuous(breaks=seq(1,3.25,0.25))+
    scale_y_continuous(breaks=seq(0,17,1))+
    scale_shape_manual(values=c(22,24,21,23))+
    scale_fill_manual(values=c("red", "blue","light gray","yellow"))+
    scale_color_manual(values=c(NA, NA,"blue","red"),na.value=NA)+
    theme(legend.position = c(0.89,0.2))+
    labs(fill="Group",shape="Group",color="Group")+
    annotate("text",label = "Rabbit-Like Taxa Only",hjust=0, parse = FALSE,
             x = 1.2, y = 16.5)+
    geom_text(aes(x = 1.2, y = 15.5, label = lm_eqn(plot_rabbit)),hjust=0,
              parse = TRUE,data.frame()))
```

Figure 8. Scatter plot of natural log of occipital condyle width (OCW) raised to the 2/3 power against the natural log of body mass, showing groups that deviate from the main regression line (cingulates, monotremes, and taxa with rabbit-like occiputs) as well as the regression line formed by taxa with rabbit-like occiputs (in red).

## 5.2 Homogeneity of slopes test between specimens with rabbit-like occiputs and remaining sample

```
fit.rabbit1<-lm(log(bm)~rabbit*I(log(ocw)^(2/3)),data=b)
summary(fit.rabbit1)
```

```
Call:
lm(formula = log(bm) ~ rabbit * I(log(ocw)^(2/3)), data = b)

Residuals:
     Min       1Q   Median       3Q      Max 
-1.29030 -0.21409 -0.00611  0.20005  1.13689 

Coefficients:
                            Estimate Std. Error t value            Pr(>|t|)    
(Intercept)                 -8.33426    0.10293 -80.968 <0.0000000000000002 ***
rabbitYes                    0.68780    0.67964   1.012               0.312    
I(log(ocw)^(2/3))            7.74524    0.05008 154.655 <0.0000000000000002 ***
rabbitYes:I(log(ocw)^(2/3))  0.01847    0.37239   0.050               0.960    
---
Signif. codes:  0 '***' 0.001 '**' 0.01 '*' 0.05 '.' 0.1 ' ' 1

Residual standard error: 0.36 on 400 degrees of freedom
Multiple R-squared:  0.9839,    Adjusted R-squared:  0.9838 
F-statistic:  8162 on 3 and 400 DF,  p-value: < 0.00000000000000022
```

## 5.3 Regression with extra variables for monotremes and rabbit-like taxa

```
fit.rabbit<-lm(log(bm)~monotreme+rabbit+I(log(ocw)^(2/3)),data=b)
summary(fit.rabbit)
```

```
Call:
lm(formula = log(bm) ~ monotreme + rabbit + I(log(ocw)^(2/3)), 
    data = b)

Residuals:
     Min       1Q   Median       3Q      Max 
-0.89942 -0.21718 -0.01579  0.19318  1.13044 

Coefficients:
                  Estimate Std. Error t value             Pr(>|t|)    
(Intercept)       -8.35284    0.09817 -85.087 < 0.0000000000000002 ***
monotremeYes      -1.14562    0.20090  -5.702          0.000000023 ***
rabbitYes          0.71517    0.08202   8.720 < 0.0000000000000002 ***
I(log(ocw)^(2/3))  7.75884    0.04778 162.384 < 0.0000000000000002 ***
---
Signif. codes:  0 '***' 0.001 '**' 0.01 '*' 0.05 '.' 0.1 ' ' 1

Residual standard error: 0.3462 on 400 degrees of freedom
Multiple R-squared:  0.9851,    Adjusted R-squared:  0.985 
F-statistic:  8837 on 3 and 400 DF,  p-value: < 0.00000000000000022
```

```
par(mfrow=c(2,2))
plot(fit.rabbit)
```

```
datatable(data.frame(residuals=sort(fit.rabbit$residuals)))
```

```
(fit.rabbit.stats<-regression.stats(fit.rabbit))
```

### 5.3.1 Fitted values with correction factor

```
datatable(data.frame(fitted.bm=round(exp(fit.rabbit$fitted.values)*fit.rabbit.stats$CF,2)))
```

**Percent of taxa within 20% of actual value, controlling for condyle shape**

```
sum(abs((exp(fit.rabbit$fitted.values)*regression.stats(fit.rabbit)$CF-b$bm)/b$bm)<0.2)/length(b$bm)
```

```
[1] 0.4653465
```

# 6 Differences in regression slope and y-intercept between clades

## 6.1 Testing for differences in intercept

```
summary(lm(log(bm)~group+I(log(ocw)^(2/3)),data=b),digits=1)
```

```
Call:
lm(formula = log(bm) ~ group + I(log(ocw)^(2/3)), data = b)

Residuals:
    Min      1Q  Median      3Q     Max 
-0.7785 -0.1853 -0.0029  0.1707  1.0103 

Coefficients:
                      Estimate Std. Error t value             Pr(>|t|)    
(Intercept)           -8.88607    0.23155 -38.376 < 0.0000000000000002 ***
groupAnomaluromorpha   0.06886    0.23754   0.290              0.77206    
groupArtiodactyla     -0.24816    0.19369  -1.281              0.20090    
groupCarnivora        -0.27474    0.18591  -1.478              0.14031    
groupCastorimorpha     0.61197    0.20514   2.983              0.00304 ** 
groupCingulata        -0.62764    0.22188  -2.829              0.00493 ** 
groupDasyuromorphia   -0.15538    0.20742  -0.749              0.45425    
groupDermoptera        1.00905    0.35903   2.811              0.00521 ** 
groupDidelphimorphia  -0.01168    0.18938  -0.062              0.95085    
groupDiprotodontia     0.19972    0.19374   1.031              0.30327    
groupEulipotyphla      0.14409    0.19181   0.751              0.45300    
groupHyracoidea       -0.04815    0.23865  -0.202              0.84023    
groupHystricomorpha    0.18489    0.19506   0.948              0.34382    
groupLagomorpha        0.63037    0.20482   3.078              0.00224 ** 
groupMacroscelidea    -0.51760    0.23741  -2.180              0.02987 *  
groupMicrobiotheria   -0.21387    0.35950  -0.595              0.55227    
groupMonotremata      -1.32228    0.25655  -5.154          0.000000413 ***
groupMyomorpha         0.14604    0.18774   0.778              0.43712    
groupPaucituberculata -0.64657    0.25419  -2.544              0.01137 *  
groupPeramelemorphia  -0.03392    0.25448  -0.133              0.89404    
groupPerissodactyla   -0.31849    0.23346  -1.364              0.17331    
groupPholidota        -0.02195    0.36001  -0.061              0.95142    
groupPilosa           -0.01013    0.23988  -0.042              0.96634    
groupPrimates         -0.25140    0.18835  -1.335              0.18278    
groupProboscidea      -0.69069    0.37595  -1.837              0.06697 .  
groupScandentia       -0.53000    0.25384  -2.088              0.03748 *  
groupSciuromorpha      0.04638    0.18862   0.246              0.80589    
groupTubulidentata    -0.14710    0.36317  -0.405              0.68568    
I(log(ocw)^(2/3))      8.07781    0.08257  97.825 < 0.0000000000000002 ***
---
Signif. codes:  0 '***' 0.001 '**' 0.01 '*' 0.05 '.' 0.1 ' ' 1

Residual standard error: 0.3108 on 375 degrees of freedom
Multiple R-squared:  0.9888,    Adjusted R-squared:  0.9879 
F-statistic:  1179 on 28 and 375 DF,  p-value: < 0.00000000000000022
```

## 6.2 Testing for differences in slope

Treating Artiodactyla as the reference level due to the small sample of afrosoricidans in the sample (N=3)

```
summary(lm(log(bm)~group*I(log(ocw)^(2/3)),data=b %>% 
  mutate(group = relevel(factor(group), ref = "Artiodactyla"))))
```

```
Call:
lm(formula = log(bm) ~ group * I(log(ocw)^(2/3)), data = b %>% 
    mutate(group = relevel(factor(group), ref = "Artiodactyla")))

Residuals:
    Min      1Q  Median      3Q     Max 
-0.8830 -0.1670 -0.0051  0.1604  1.0828 

Coefficients: (5 not defined because of singularities)
                                         Estimate Std. Error t value             Pr(>|t|)    
(Intercept)                             -8.222926   0.541299 -15.191 < 0.0000000000000002 ***
groupAfrosoricida                        7.049410   2.111295   3.339             0.000931 ***
groupAnomaluromorpha                    -2.666696   1.546496  -1.724             0.085521 .  
groupCarnivora                          -2.046701   0.640657  -3.195             0.001526 ** 
groupCastorimorpha                       0.346108   0.813845   0.425             0.670895    
groupCingulata                          -2.546690   2.560381  -0.995             0.320587    
groupDasyuromorphia                     -1.800118   0.924746  -1.947             0.052375 .  
groupDermoptera                          1.034884   0.325092   3.183             0.001585 ** 
groupDidelphimorphia                    -2.042340   0.797221  -2.562             0.010827 *  
groupDiprotodontia                       0.087828   0.704400   0.125             0.900844    
groupEulipotyphla                        0.004243   0.671958   0.006             0.994966    
groupHyracoidea                         10.894740   9.624115   1.132             0.258392    
groupHystricomorpha                      2.305487   0.956300   2.411             0.016426 *  
groupLagomorpha                         -0.571445   1.372021  -0.416             0.677298    
groupMacroscelidea                      -0.714755   2.201135  -0.325             0.745584    
groupMicrobiotheria                     -0.316043   0.362489  -0.872             0.383873    
groupMonotremata                         5.623098   7.876207   0.714             0.475739    
groupMyomorpha                          -0.398834   0.723102  -0.552             0.581599    
groupPaucituberculata                    6.468879  11.950306   0.541             0.588632    
groupPeramelemorphia                    -4.243328   5.694382  -0.745             0.456660    
groupPerissodactyla                      6.227462   3.532035   1.763             0.078743 .  
groupPholidota                           0.090243   0.307947   0.293             0.769658    
groupPilosa                              3.008633   2.210399   1.361             0.174342    
groupPrimates                           -1.344552   0.828598  -1.623             0.105550    
groupProboscidea                        -0.204760   0.328903  -0.623             0.533978    
groupScandentia                          0.046293   5.651875   0.008             0.993469    
groupSciuromorpha                       -0.934085   0.866113  -1.078             0.281556    
groupTubulidentata                       0.087592   0.297338   0.295             0.768482    
I(log(ocw)^(2/3))                        7.710263   0.217682  35.420 < 0.0000000000000002 ***
groupAfrosoricida:I(log(ocw)^(2/3))     -3.984970   1.168128  -3.411             0.000721 ***
groupAnomaluromorpha:I(log(ocw)^(2/3))   1.475881   0.800897   1.843             0.066199 .  
groupCarnivora:I(log(ocw)^(2/3))         0.874961   0.267870   3.266             0.001196 ** 
groupCastorimorpha:I(log(ocw)^(2/3))     0.118353   0.435073   0.272             0.785759    
groupCingulata:I(log(ocw)^(2/3))         0.954486   1.188289   0.803             0.422374    
groupDasyuromorphia:I(log(ocw)^(2/3))    0.889501   0.451235   1.971             0.049475 *  
groupDermoptera:I(log(ocw)^(2/3))              NA         NA      NA                   NA    
groupDidelphimorphia:I(log(ocw)^(2/3))   1.188767   0.412022   2.885             0.004152 ** 
groupDiprotodontia:I(log(ocw)^(2/3))     0.086457   0.314686   0.275             0.783676    
groupEulipotyphla:I(log(ocw)^(2/3))      0.026648   0.336243   0.079             0.936877    
groupHyracoidea:I(log(ocw)^(2/3))       -5.249652   4.655145  -1.128             0.260208    
groupHystricomorpha:I(log(ocw)^(2/3))   -0.999833   0.442922  -2.257             0.024596 *  
groupLagomorpha:I(log(ocw)^(2/3))        0.653811   0.702723   0.930             0.352802    
groupMacroscelidea:I(log(ocw)^(2/3))     0.102187   1.231453   0.083             0.933914    
groupMicrobiotheria:I(log(ocw)^(2/3))          NA         NA      NA                   NA    
groupMonotremata:I(log(ocw)^(2/3))      -3.051171   3.536507  -0.863             0.388853    
groupMyomorpha:I(log(ocw)^(2/3))         0.292208   0.373705   0.782             0.434785    
groupPaucituberculata:I(log(ocw)^(2/3)) -4.487581   7.453733  -0.602             0.547522    
groupPeramelemorphia:I(log(ocw)^(2/3))   2.142493   2.844266   0.753             0.451791    
groupPerissodactyla:I(log(ocw)^(2/3))   -2.278143   1.298535  -1.754             0.080231 .  
groupPholidota:I(log(ocw)^(2/3))               NA         NA      NA                   NA    
groupPilosa:I(log(ocw)^(2/3))           -1.315323   1.001172  -1.314             0.189772    
groupPrimates:I(log(ocw)^(2/3))          0.565809   0.361432   1.565             0.118370    
groupProboscidea:I(log(ocw)^(2/3))             NA         NA      NA                   NA    
groupScandentia:I(log(ocw)^(2/3))       -0.354371   3.282568  -0.108             0.914093    
groupSciuromorpha:I(log(ocw)^(2/3))      0.539419   0.424996   1.269             0.205194    
groupTubulidentata:I(log(ocw)^(2/3))           NA         NA      NA                   NA    
---
Signif. codes:  0 '***' 0.001 '**' 0.01 '*' 0.05 '.' 0.1 ' ' 1

Residual standard error: 0.2944 on 353 degrees of freedom
Multiple R-squared:  0.9905,    Adjusted R-squared:  0.9892 
F-statistic: 737.4 on 50 and 353 DF,  p-value: < 0.00000000000000022
```

### 6.2.1 Differences in slope between orders

```
d_order<-tidy(lm(log(bm)~I(log(ocw)^(2/3))*group,data=b),conf.int=TRUE)%>%
  mutate(slope=estimate[2]+estimate,
                slope.low=estimate[2]+conf.low,
                slope.high=estimate[2]+conf.high)%>%
  mutate(slope=ifelse(term=="I(log(ocw)^(2/3))",estimate,slope))%>%
  mutate(slope.low=ifelse(term=="I(log(ocw)^(2/3))",conf.low,slope.low))%>%
  mutate(slope.high=ifelse(term=="I(log(ocw)^(2/3))",conf.high,slope.high))%>%
  mutate(group=c("Intercept","Afrosoricida",rep("NA",27),levels(as.factor(b$group))[-c(1)]))
  
ggplot(d_order %>% filter(str_detect(term, 'ocw')),
       aes(slope,factor(group,levels=rev(as.factor(group)))))+
  geom_vline(xintercept = fit.all$coefficients[2],linetype="dashed")+
  geom_errorbarh(aes(xmin=slope.low,xmax=slope.high,height=0))+
  geom_point(shape=24,size=2,fill="black")+
  labs(x="Slope",y="Taxonomic Group")+
  coord_cartesian(xlim=c(0,15))
```

Graph of slopes and 95% confidence intervals

## 6.3 Scatterplot of groups color-coded by taxonomic suborder/order and ordinal regression equations

```
plot_data<-data.frame(b,predict(fit.all,interval="prediction"))
(scatterplot.ordinal<-ggplot(plot_data %>% group_by(group) %>% filter(n() > 4),aes(log(ocw)^(2/3),log(bm),col=group))+
    geom_point(data=plot_data,size=2.5,shape=21,fill="gray",col="black")+
    geom_point(size=2.5,shape=21,aes(fill=group),col="black")+
    labs(x=expression(paste("Natural Log OCW (mm)"^{2/3})),
         y="Natural Log Body Mass (g)")+
    scale_x_continuous(breaks=seq(1,3.25,0.25))+
    scale_y_continuous(breaks=seq(0,17,1))+
    geom_smooth(data=plot_data %>% group_by(group) %>% filter(n() > 4),
              aes(group=group),formula=y~x,method="lm",size=1.35,col="black",se=F)+
    geom_smooth(data=plot_data %>% group_by(group) %>% filter(n() > 4),
                formula=y~x,method="lm",level=0.9,se=F)+
    geom_abline(slope=fit.all$coefficients[2],intercept=fit.all$coefficients[1],
                linetype="dashed",size=.75)+
    labs(color="Order",fill="Order")+
    guides(color=guide_legend(ncol=2),fill=guide_legend(ncol=2))+
    theme_classic()+
    theme(legend.position=c(0.8,0.2)))
```

Figure S4. Ordinal-level regression equations for various orders of mammals (and suborders of rodents) for which = 5 species are sampled. The dashed black line represents the best fit line of the total dataset. Data points which pertain to clades for which N < 5 are denoted in gray.

# 7 Subsetted species-average datasets

## 7.1 Reduced dataset excluding taxa with apomorphic occiput morphology

```
reduced<-b[b$outliertaxon!="Yes",]
fit.reduced<-lm(data=reduced,log(bm)~I(log(ocw)^(2/3)))
par(mfrow=c(2,2))
plot(fit.reduced)
```

```
summary(fit.reduced)
```

```
Call:
lm(formula = log(bm) ~ I(log(ocw)^(2/3)), data = reduced)

Residuals:
     Min       1Q   Median       3Q      Max 
-0.88404 -0.21799 -0.01586  0.18776  1.12914 

Coefficients:
                  Estimate Std. Error t value            Pr(>|t|)    
(Intercept)       -8.36414    0.09765  -85.66 <0.0000000000000002 ***
I(log(ocw)^(2/3))  7.76568    0.04757  163.25 <0.0000000000000002 ***
---
Signif. codes:  0 '***' 0.001 '**' 0.01 '*' 0.05 '.' 0.1 ' ' 1

Residual standard error: 0.3409 on 372 degrees of freedom
Multiple R-squared:  0.9862,    Adjusted R-squared:  0.9862 
F-statistic: 2.665e+04 on 1 and 372 DF,  p-value: < 0.00000000000000022
```

```
regression.stats(fit.reduced)
```

## 7.2 Therian-only dataset (excluding monotremes)

```
therian<-b[b$group!="Monotremata",]
fit.therian<-lm(data=therian,log(bm)~I(log(ocw)^(2/3)))
par(mfrow=c(2,2))
plot(fit.therian)
```

```
summary(fit.therian)
```

```
Call:
lm(formula = log(bm) ~ I(log(ocw)^(2/3)), data = therian)

Residuals:
     Min       1Q   Median       3Q      Max 
-0.93067 -0.24021 -0.03239  0.19824  1.11107 

Coefficients:
                  Estimate Std. Error t value            Pr(>|t|)    
(Intercept)       -8.21527    0.10575  -77.69 <0.0000000000000002 ***
I(log(ocw)^(2/3))  7.70727    0.05175  148.94 <0.0000000000000002 ***
---
Signif. codes:  0 '***' 0.001 '**' 0.01 '*' 0.05 '.' 0.1 ' ' 1

Residual standard error: 0.3779 on 399 degrees of freedom
Multiple R-squared:  0.9823,    Adjusted R-squared:  0.9823 
F-statistic: 2.218e+04 on 1 and 399 DF,  p-value: < 0.00000000000000022
```

```
regression.stats(fit.therian)
```

## 7.3 Dataset of all species for which N ≥ 6

```
morethansix<-b[b$N>5,]
fit.morethansix<-lm(log(bm)~I(log(ocw)^(2/3)),
                    data=morethansix)
par(mfrow=c(2,2))
plot(fit.morethansix)
```

```
summary(fit.morethansix)
```

```
Call:
lm(formula = log(bm) ~ I(log(ocw)^(2/3)), data = morethansix)

Residuals:
     Min       1Q   Median       3Q      Max 
-0.86742 -0.22257 -0.01309  0.19776  0.89594 

Coefficients:
                  Estimate Std. Error t value            Pr(>|t|)    
(Intercept)       -8.18776    0.16473   -49.7 <0.0000000000000002 ***
I(log(ocw)^(2/3))  7.68862    0.08767    87.7 <0.0000000000000002 ***
---
Signif. codes:  0 '***' 0.001 '**' 0.01 '*' 0.05 '.' 0.1 ' ' 1

Residual standard error: 0.3646 on 168 degrees of freedom
Multiple R-squared:  0.9786,    Adjusted R-squared:  0.9785 
F-statistic:  7691 on 1 and 168 DF,  p-value: < 0.00000000000000022
```

```
regression.stats(fit.morethansix)
```

## 7.4 Dataset of all species for which N ≥ 6, excluding taxa with specialized occiput morphology

```
morethansix.reduced<-morethansix[morethansix$outliertaxon!="Yes",]
fit.morethansix.reduced<-lm(log(bm)~I(log(ocw)^(2/3)),data=morethansix.reduced)
par(mfrow=c(2,2))
plot(fit.morethansix.reduced)
```

```
summary(fit.morethansix.reduced)
```

```
Call:
lm(formula = log(bm) ~ I(log(ocw)^(2/3)), data = morethansix.reduced)

Residuals:
     Min       1Q   Median       3Q      Max 
-0.83911 -0.20167  0.00522  0.18902  0.91398 

Coefficients:
                  Estimate Std. Error t value            Pr(>|t|)    
(Intercept)        -8.2391     0.1578  -52.23 <0.0000000000000002 ***
I(log(ocw)^(2/3))   7.6999     0.0839   91.78 <0.0000000000000002 ***
---
Signif. codes:  0 '***' 0.001 '**' 0.01 '*' 0.05 '.' 0.1 ' ' 1

Residual standard error: 0.3457 on 158 degrees of freedom
Multiple R-squared:  0.9816,    Adjusted R-squared:  0.9815 
F-statistic:  8423 on 1 and 158 DF,  p-value: < 0.00000000000000022
```

```
regression.stats(fit.morethansix.reduced)
```

## 7.5 Dataset of all species for which N ≥ 10

```
morethanten<-b[b$N > 9,]
fit.morethanten<-lm(log(bm)~I(log(ocw)^(2/3)),data=morethanten)
par(mfrow=c(2,2))
plot(fit.morethanten)
```

```
summary(fit.morethanten)
```

```
Call:
lm(formula = log(bm) ~ I(log(ocw)^(2/3)), data = morethanten)

Residuals:
     Min       1Q   Median       3Q      Max 
-0.61004 -0.17161 -0.03406  0.13444  0.83656 

Coefficients:
                  Estimate Std. Error t value            Pr(>|t|)    
(Intercept)       -8.19504    0.16508  -49.64 <0.0000000000000002 ***
I(log(ocw)^(2/3))  7.71479    0.08983   85.88 <0.0000000000000002 ***
---
Signif. codes:  0 '***' 0.001 '**' 0.01 '*' 0.05 '.' 0.1 ' ' 1

Residual standard error: 0.2833 on 73 degrees of freedom
Multiple R-squared:  0.9902,    Adjusted R-squared:  0.9901 
F-statistic:  7376 on 1 and 73 DF,  p-value: < 0.00000000000000022
```

```
regression.stats(fit.morethanten)
```

## 7.6 Dataset of all species for which N ≥ 10, excluding taxa with specialized occiput morphology

```
morethanten.reduced<-morethanten[morethanten$outliertaxon!="Yes",]
fit.morethanten.reduced<-lm(log(bm)~I(log(ocw)^(2/3)),data=morethanten.reduced)
par(mfrow=c(2,2))
plot(fit.morethanten.reduced)
```

```
summary(fit.morethanten.reduced)
```

```
Call:
lm(formula = log(bm) ~ I(log(ocw)^(2/3)), data = morethanten.reduced)

Residuals:
     Min       1Q   Median       3Q      Max 
-0.59239 -0.16268 -0.02759  0.13562  0.79834 

Coefficients:
                  Estimate Std. Error t value            Pr(>|t|)    
(Intercept)       -8.21147    0.15438  -53.19 <0.0000000000000002 ***
I(log(ocw)^(2/3))  7.71406    0.08391   91.93 <0.0000000000000002 ***
---
Signif. codes:  0 '***' 0.001 '**' 0.01 '*' 0.05 '.' 0.1 ' ' 1

Residual standard error: 0.2634 on 71 degrees of freedom
Multiple R-squared:  0.9917,    Adjusted R-squared:  0.9916 
F-statistic:  8451 on 1 and 71 DF,  p-value: < 0.00000000000000022
```

```
regression.stats(fit.morethanten.reduced)
```

# 8 OCW scaling patterns in different size classes

```
fit.onekg<-lm(log(bm)~I(log(ocw)^(2/3)),data=onekg)
par(mfrow=c(2,2))
plot(fit.onekg)
```

```
summary(fit.onekg)
```

```
Call:
lm(formula = log(bm) ~ I(log(ocw)^(2/3)), data = onekg)

Residuals:
     Min       1Q   Median       3Q      Max 
-1.35061 -0.23815 -0.04096  0.20871  1.16284 

Coefficients:
                  Estimate Std. Error t value            Pr(>|t|)    
(Intercept)        -7.5507     0.2481  -30.43 <0.0000000000000002 ***
I(log(ocw)^(2/3))   7.4292     0.1091   68.12 <0.0000000000000002 ***
---
Signif. codes:  0 '***' 0.001 '**' 0.01 '*' 0.05 '.' 0.1 ' ' 1

Residual standard error: 0.3796 on 230 degrees of freedom
Multiple R-squared:  0.9528,    Adjusted R-squared:  0.9526 
F-statistic:  4640 on 1 and 230 DF,  p-value: < 0.00000000000000022
```

```
regression.stats(fit.onekg)
```

```
b$bin<-cut(b$bm,labels=c("< 1000 g","> 1000 g"),breaks=c(-Inf,1000,Inf))
b$bin2<-cut(b$bm,labels=c("< 100 g","> 100 g"),breaks=c(-Inf,100,Inf))
b$bin3<-cut(b$bm,labels=c("< 10000 g","> 10000 g"),breaks=c(-Inf,10000,Inf))
```

Comparing untransformed regression lines for specimens above and below 1000 g

```
summary(lm(log(bm)~log(ocw)*bin,data=b))
```

```
Call:
lm(formula = log(bm) ~ log(ocw) * bin, data = b)

Residuals:
     Min       1Q   Median       3Q      Max 
-1.30636 -0.24801 -0.03455  0.22037  1.12112 

Coefficients:
                     Estimate Std. Error t value             Pr(>|t|)    
(Intercept)          -3.50415    0.16496 -21.242 < 0.0000000000000002 ***
log(ocw)              3.73012    0.07464  49.973 < 0.0000000000000002 ***
bin> 1000 g           1.70143    0.23250   7.318     0.00000000000139 ***
log(ocw):bin> 1000 g -0.49222    0.08840  -5.568     0.00000004736943 ***
---
Signif. codes:  0 '***' 0.001 '**' 0.01 '*' 0.05 '.' 0.1 ' ' 1

Residual standard error: 0.3787 on 400 degrees of freedom
Multiple R-squared:  0.9822,    Adjusted R-squared:  0.9821 
F-statistic:  7364 on 3 and 400 DF,  p-value: < 0.00000000000000022
```

Comparing regression lines for specimens above and below 1000 g

```
summary(lm(log(bm)~I(log(ocw)^(2/3))*bin,data=b))
```

```
Call:
lm(formula = log(bm) ~ I(log(ocw)^(2/3)) * bin, data = b)

Residuals:
     Min       1Q   Median       3Q      Max 
-1.35061 -0.24849 -0.03388  0.21500  1.16284 

Coefficients:
                              Estimate Std. Error t value            Pr(>|t|)    
(Intercept)                   -7.45698    0.24300 -30.687 <0.0000000000000002 ***
I(log(ocw)^(2/3))              7.21351    0.14421  50.022 <0.0000000000000002 ***
bin> 1000 g                   -0.09372    0.34702  -0.270               0.787    
I(log(ocw)^(2/3)):bin> 1000 g  0.21573    0.18070   1.194               0.233    
---
Signif. codes:  0 '***' 0.001 '**' 0.01 '*' 0.05 '.' 0.1 ' ' 1

Residual standard error: 0.379 on 400 degrees of freedom
Multiple R-squared:  0.9822,    Adjusted R-squared:  0.9821 
F-statistic:  7352 on 3 and 400 DF,  p-value: < 0.00000000000000022
```

Comparing regression lines for specimens above and below 100 g

```
summary(lm(log(bm)~I(log(ocw)^(2/3))*bin2,data=b))
```

```
Call:
lm(formula = log(bm) ~ I(log(ocw)^(2/3)) * bin2, data = b)

Residuals:
     Min       1Q   Median       3Q      Max 
-1.31388 -0.23443 -0.02236  0.21062  1.15064 

Coefficients:
                              Estimate Std. Error t value             Pr(>|t|)    
(Intercept)                    -6.4016     0.5325 -12.022 < 0.0000000000000002 ***
I(log(ocw)^(2/3))               6.4422     0.3528  18.258 < 0.0000000000000002 ***
bin2> 100 g                    -1.4744     0.5579  -2.643              0.00854 ** 
I(log(ocw)^(2/3)):bin2> 100 g   1.1132     0.3611   3.083              0.00219 ** 
---
Signif. codes:  0 '***' 0.001 '**' 0.01 '*' 0.05 '.' 0.1 ' ' 1

Residual standard error: 0.3807 on 400 degrees of freedom
Multiple R-squared:  0.982, Adjusted R-squared:  0.9819 
F-statistic:  7283 on 3 and 400 DF,  p-value: < 0.00000000000000022
```

Comparing regression lines for specimens above and below 10000 g

```
summary(lm(log(bm)~I(log(ocw)^(2/3))*bin3,data=b))
```

```
Call:
lm(formula = log(bm) ~ I(log(ocw)^(2/3)) * bin3, data = b)

Residuals:
     Min       1Q   Median       3Q      Max 
-1.21713 -0.24277 -0.02531  0.19400  1.17260 

Coefficients:
                                Estimate Std. Error t value            Pr(>|t|)    
(Intercept)                     -7.92325    0.15209 -52.095 <0.0000000000000002 ***
I(log(ocw)^(2/3))                7.53369    0.08068  93.378 <0.0000000000000002 ***
bin3> 10000 g                    0.85967    0.59345   1.449               0.148    
I(log(ocw)^(2/3)):bin3> 10000 g -0.26383    0.24417  -1.081               0.281    
---
Signif. codes:  0 '***' 0.001 '**' 0.01 '*' 0.05 '.' 0.1 ' ' 1

Residual standard error: 0.3847 on 400 degrees of freedom
Multiple R-squared:  0.9816,    Adjusted R-squared:  0.9815 
F-statistic:  7130 on 3 and 400 DF,  p-value: < 0.00000000000000022
```

## 8.1 Plotting differences in slope between transformed and non-transformed datasets for different size classes

```
onekg_linear<-ggplot(b,aes(log(ocw),log(bm),col=bin))+
  geom_point(data=subset(b,bm>1000),size=2,fill="grey",shape=21)+
  geom_point(data=subset(b,bm<1000),size=2,fill="white",shape=21)+
  geom_smooth(data=b %>% group_by(group),formula=y~x,
              method="lm",level=0.9,se=F)+
  geom_smooth(data=subset(b,bm>1000),formula=y~x,method="lm",color="blue",
              level=0.9,fullrange=TRUE)+
  geom_smooth(data=subset(b,bm<1000),formula=y~x,method="lm",color="red",
              level=0.9,fullrange=TRUE)+
  geom_hline(yintercept=log(1000),linetype="dashed")+
  labs(x="Unadjusted Natural Log OCW (mm)",y="Natural Log Body Mass (g)")+
  guides(color=guide_legend(ncol=2),fill=guide_legend(ncol=2))+
  theme_classic()+
  scale_color_manual(values=c("red","blue"))+
  scale_x_continuous(breaks=seq(1,6,0.5))+
  scale_y_continuous(breaks=seq(0,18,1))+
  guides(color = guide_legend(override.aes=list(fill = c("white","gray"))))+
  ggtitle("A")+
  theme(legend.position = c(0.89,0.2))+
  theme(legend.title=element_blank())
onekg_nonlinear<-ggplot(b,aes(log(ocw)^(2/3),log(bm),col=bin))+
    geom_point(data=subset(b,bm>1000),size=2,fill="grey",shape=21)+
    geom_point(data=subset(b,bm<1000),size=2,fill="white",shape=21)+
    geom_smooth(data=b %>% group_by(group),formula=y~x,
                method="lm",level=0.9,se=F)+
  geom_smooth(data=subset(b,bm>1000),formula=y~x,method="lm",color="blue",
              level=0.9,fullrange=TRUE)+
  geom_smooth(data=subset(b,bm<1000),formula=y~x,method="lm",color="red",
              level=0.9,fullrange=TRUE)+
  geom_hline(yintercept=log(1000),linetype="dashed")+
  labs(x=expression(paste("Natural Log OCW (mm)"^{2/3})),
       y="Natural Log Body Mass (g)")+
  guides(color=guide_legend(ncol=2),fill=guide_legend(ncol=2))+
  theme_classic()+
  scale_color_manual(values=c("red","blue"))+
  scale_x_continuous(breaks=seq(1,3.25,0.25))+
  scale_y_continuous(breaks=seq(0,18,1))+
  ggtitle("B")+
  guides(color = guide_legend(override.aes=list(fill = c("white","gray"))))+
  theme(legend.position = c(0.89,0.2))+
  theme(legend.title=element_blank())
hundredg_linear<-ggplot(b,aes(log(ocw),log(bm),col=bin2))+
  geom_point(data=subset(b,bm>100),size=2,fill="grey",shape=21)+
  geom_point(data=subset(b,bm<100),size=2,fill="white",shape=21)+
  geom_smooth(data=b %>% group_by(group),formula=y~x,
              method="lm",level=0.9,se=F)+
  geom_smooth(data=subset(b,bm>100),formula=y~x,method="lm",color="blue",
              level=0.9,fullrange=TRUE)+
  geom_smooth(data=subset(b,bm<100),formula=y~x,method="lm",color="red",
              level=0.9,fullrange=TRUE)+
  geom_hline(yintercept=log(100),linetype="dashed")+
  labs(x=expression(paste("Unadjusted Natural Log OCW (mm)")),
       y="Natural Log Body Mass (g)")+
  guides(color=guide_legend(ncol=2),fill=guide_legend(ncol=2))+
  theme_classic()+
  scale_color_manual(values=c("red","blue"))+
  scale_x_continuous(breaks=seq(1,6,0.5))+
  scale_y_continuous(breaks=seq(0,18,1))+
  ggtitle("C")+
  guides(color = guide_legend(override.aes=list(fill = c("white","gray"))))+
  theme(legend.position = c(0.89,0.2))+
  theme(legend.title=element_blank())
hundredg_nonlinear<-ggplot(b,aes(log(ocw)^(2/3),log(bm),col=bin2))+
  geom_point(data=subset(b,bm>100),size=2,fill="grey",shape=21)+
  geom_point(data=subset(b,bm<100),size=2,fill="white",shape=21)+
  geom_smooth(data=b %>% group_by(group),formula=y~x,
              method="lm",level=0.9,se=F)+
  geom_smooth(data=subset(b,bm>100),formula=y~x,method="lm",color="blue",
              level=0.9,fullrange=TRUE)+
  geom_smooth(data=subset(b,bm<100),formula=y~x,method="lm",color="red",
              level=0.9,fullrange=TRUE)+
  geom_hline(yintercept=log(100),linetype="dashed")+
  labs(x=expression(paste("Natural Log OCW (mm)"^{2/3})),
       y="Natural Log Body Mass (g)")+
  guides(color=guide_legend(ncol=2),fill=guide_legend(ncol=2))+
  theme_classic()+
  scale_color_manual(values=c("red","blue"))+
  scale_x_continuous(breaks=seq(1,3.25,0.25))+
  scale_y_continuous(breaks=seq(0,18,1))+
  ggtitle("D")+
  guides(color = guide_legend(override.aes=list(fill = c("white","gray"))))+
  theme(legend.position = c(0.89,0.2))+
  theme(legend.title=element_blank())
tenkg_linear<-ggplot(b,aes(log(ocw),log(bm),col=bin3))+
  geom_point(data=subset(b,bm>10000),size=2,fill="grey",shape=21)+
  geom_point(data=subset(b,bm<10000),size=2,fill="white",shape=21)+
  geom_smooth(data=b %>% group_by(group),formula=y~x,
              method="lm",level=0.9,se=F)+
  geom_smooth(data=subset(b,bm>10000),formula=y~x,method="lm",color="blue",
              level=0.9,fullrange=TRUE)+
  geom_smooth(data=subset(b,bm<10000),formula=y~x,method="lm",color="red",
              level=0.9,fullrange=TRUE)+
  geom_hline(yintercept=log(10000),linetype="dashed")+
  labs(x=expression(paste("Unadjusted Natural Log OCW (mm)")),y="Natural Log Body Mass (g)")+
  guides(color=guide_legend(ncol=2),fill=guide_legend(ncol=2))+
  theme_classic()+
  scale_color_manual(values=c("red","blue"))+
  scale_x_continuous(breaks=seq(1,6,0.5))+
  scale_y_continuous(breaks=seq(0,18,1))+
  ggtitle("E")+
  guides(color = guide_legend(override.aes=list(fill = c("white","gray"))))+
  theme(legend.position = c(0.89,0.2))+
  theme(legend.title=element_blank())
tenkg_nonlinear<-ggplot(b,aes(log(ocw)^(2/3),log(bm),col=bin3))+
  geom_point(data=subset(b,bm>10000),size=2,fill="grey",shape=21)+
  geom_point(data=subset(b,bm<10000),size=2,fill="white",shape=21)+
  geom_smooth(data=b %>% group_by(group),formula=y~x,
              method="lm",level=0.9,se=F)+
  geom_smooth(data=subset(b,bm>10000),formula=y~x,method="lm",color="blue",
              level=0.9,fullrange=TRUE)+
  geom_smooth(data=subset(b,bm<10000),formula=y~x,method="lm",color="red",
              level=0.9,fullrange=TRUE)+
  geom_hline(yintercept=log(10000),linetype="dashed")+
  labs(x=expression(paste("Natural Log OCW (mm)"^{2/3})),y="Natural Log Body Mass (g)")+
  guides(color=guide_legend(ncol=2),fill=guide_legend(ncol=2))+
  theme_classic()+
  scale_color_manual(values=c("red","blue"))+
  scale_x_continuous(breaks=seq(1,3.25,0.25))+
  scale_y_continuous(breaks=seq(0,18,1))+
  ggtitle("F")+
  guides(color = guide_legend(override.aes=list(fill = c("white","gray"))))+
  theme(legend.position = c(0.89,0.2))+
  theme(legend.title=element_blank())
sizeclassplot<-grid.arrange(onekg_linear,onekg_nonlinear,hundredg_linear,hundredg_nonlinear,tenkg_linear,tenkg_nonlinear,ncol=2)
```

Figure 4. Comparison of scaling patterns for different size classes. **A**, **C**, **E**: log-linear scaling relationships; **B**, **D**, **F**: scaling relationships of the data where log OCW is transformed by raising it to the 2/3 power. **A-B**, scaling patterns for taxa above and below 1 kg. **C-D**, scaling patterns for taxa above and below 100g. **E-F**, scaling patters for taxa above and below 10 kg.

# 9 Regressions for taxonomic subsets

## 9.1 Rodentia dataset

```
rodent<-subset(b, subset = group %in% c("Sciuromorpha","Castorimorpha","Anomaluromorpha","Hystricomorpha","Myomorpha"))
fit.rodent<-lm(log(bm)~I(log(ocw)^(2/3)),data=rodent)
par(mfrow=c(2,2))
plot(fit.rodent)
```

```
summary(fit.rodent)
```

```
Call:
lm(formula = log(bm) ~ I(log(ocw)^(2/3)), data = rodent)

Residuals:
     Min       1Q   Median       3Q      Max 
-0.74888 -0.25326 -0.06054  0.20704  0.78652 

Coefficients:
                  Estimate Std. Error t value            Pr(>|t|)    
(Intercept)        -8.2573     0.2457  -33.60 <0.0000000000000002 ***
I(log(ocw)^(2/3))   7.8157     0.1385   56.41 <0.0000000000000002 ***
---
Signif. codes:  0 '***' 0.001 '**' 0.01 '*' 0.05 '.' 0.1 ' ' 1

Residual standard error: 0.3422 on 94 degrees of freedom
Multiple R-squared:  0.9713,    Adjusted R-squared:  0.971 
F-statistic:  3183 on 1 and 94 DF,  p-value: < 0.00000000000000022
```

### 9.1.1 Residuals for rodent-only regression

```
invisible(ocwhistogram.rodent<-ggplot(data=fit.rodent,aes(fit.rodent$residuals))+
        geom_histogram(binwidth=0.1,color="black",fill="white")+
        geom_vline(aes(xintercept=mean(fit.all$residuals)),
                   color="black", linetype="dashed", size=0.5)+
        stat_function(fun = function(x) dnorm(x, mean = mean(fit.rodent$residuals), sd = sd(fit.rodent$residuals)) * nrow(rodent) * 0.1,
                      color = "red", size = 0.5)+
        labs(x= expression(paste("Residuals")),y="Counts")+
        ggtitle("A")+
        theme_classic())
invisible(QQocw.rodent<-autoplot(fit.rodent,which=2,ncol=1,label=FALSE,
                        fill="light gray",shape=21,size=2.5)+
        ggtitle("B")+
        theme_classic())
QQhist.rodent<-grid.arrange(ocwhistogram.rodent,QQocw.rodent[[1]],nrow=1)
```

Figure S5. Histogram (**A**) and Q-Q plot (**B**) of the residuals for the rodent regression equation.

## 9.2 Sciuromorpha dataset

```
squirrel<-b[b$group=="Sciuromorpha",]
fit.squirrel<-lm(log(bm)~I(log(ocw)^(2/3)),data=squirrel)
par(mfrow=c(2,2))
plot(fit.squirrel)
```

```
summary(fit.squirrel)
```

```
Call:
lm(formula = log(bm) ~ I(log(ocw)^(2/3)), data = squirrel)

Residuals:
     Min       1Q   Median       3Q      Max 
-0.35415 -0.10472 -0.03178  0.11464  0.44691 

Coefficients:
                  Estimate Std. Error t value            Pr(>|t|)    
(Intercept)        -9.1570     0.4841  -18.91 <0.0000000000000002 ***
I(log(ocw)^(2/3))   8.2497     0.2614   31.56 <0.0000000000000002 ***
---
Signif. codes:  0 '***' 0.001 '**' 0.01 '*' 0.05 '.' 0.1 ' ' 1

Residual standard error: 0.2108 on 27 degrees of freedom
Multiple R-squared:  0.9736,    Adjusted R-squared:  0.9726 
F-statistic: 996.3 on 1 and 27 DF,  p-value: < 0.00000000000000022
```

## 9.3 Carnivora dataset

```
carnivora<-b[b$group=="Carnivora",]
fit.carnivora<-lm(log(bm)~I(log(ocw)^(2/3)),data=carnivora)
par(mfrow=c(2,2))
plot(fit.carnivora)
```

```
summary(fit.carnivora)
```

```
Call:
lm(formula = log(bm) ~ I(log(ocw)^(2/3)), data = carnivora)

Residuals:
     Min       1Q   Median       3Q      Max 
-0.88299 -0.16866  0.00155  0.19912  0.72240 

Coefficients:
                  Estimate Std. Error t value            Pr(>|t|)    
(Intercept)       -10.2696     0.3371  -30.46 <0.0000000000000002 ***
I(log(ocw)^(2/3))   8.5852     0.1536   55.90 <0.0000000000000002 ***
---
Signif. codes:  0 '***' 0.001 '**' 0.01 '*' 0.05 '.' 0.1 ' ' 1

Residual standard error: 0.2896 on 79 degrees of freedom
Multiple R-squared:  0.9753,    Adjusted R-squared:  0.975 
F-statistic:  3125 on 1 and 79 DF,  p-value: < 0.00000000000000022
```

**Fitting the dataset with large dasyuromorphians (i.e., *Thylacinus*, *Dasyurus*, and *Sarcophilus*) to more directly compare to Van Valkenburgh (1990)**

```
carnivore2<-b%>%
  filter(group %in% c("Carnivora","Dasyuromorphia"))%>%
  filter(!(species %in% c("Dasyuroides_byrnei","Antechinus_stuartii","Antechinus_minimus","Myoictis_melas")))
fit.carnivore2<-lm(log(bm)~I((log(ocw))^(2/3)),data=carnivore2)
regression.stats(fit.carnivore2)
```

## 9.4 Ungulate dataset

The ungulate dataset includes Perissodactyla, Artiodactyla, and Hyracoidea, but not Proboscidea, in order to maintain parity with previous ungulate-based regression equations.

```
ungulate<-subset(b, subset = group %in% c("Artiodactyla","Perissodactyla"
                                          ,"Hyracoidea"))
fit.ungulate<-lm(log(bm)~I((log(ocw))^(2/3)),data=ungulate)
par(mfrow=c(2,2))
plot(fit.ungulate)
```

```
summary(fit.ungulate)
```

```
Call:
lm(formula = log(bm) ~ I((log(ocw))^(2/3)), data = ungulate)

Residuals:
     Min       1Q   Median       3Q      Max 
-0.61545 -0.22937 -0.03648  0.17226  1.09078 

Coefficients:
                    Estimate Std. Error t value            Pr(>|t|)    
(Intercept)          -8.0565     0.4882  -16.50 <0.0000000000000002 ***
I((log(ocw))^(2/3))   7.6451     0.1964   38.93 <0.0000000000000002 ***
---
Signif. codes:  0 '***' 0.001 '**' 0.01 '*' 0.05 '.' 0.1 ' ' 1

Residual standard error: 0.3361 on 59 degrees of freedom
Multiple R-squared:  0.9625,    Adjusted R-squared:  0.9619 
F-statistic:  1516 on 1 and 59 DF,  p-value: < 0.00000000000000022
```

## 9.5 Primates dataset

```
primates<-b%>%
  filter(group=="Primates")
fit.primates<-lm(log(bm)~I(log(ocw)^(2/3)),data=primates %>% filter(group=="Primates"))
par(mfrow=c(2,2))
plot(fit.primates)
```

```
summary(fit.primates)
```

```
Call:
lm(formula = log(bm) ~ I(log(ocw)^(2/3)), data = primates %>% 
    filter(group == "Primates"))

Residuals:
    Min      1Q  Median      3Q     Max 
-0.7323 -0.1350  0.0275  0.1415  0.4818 

Coefficients:
                  Estimate Std. Error t value            Pr(>|t|)    
(Intercept)        -9.5675     0.5329  -17.95 <0.0000000000000002 ***
I(log(ocw)^(2/3))   8.2761     0.2451   33.77 <0.0000000000000002 ***
---
Signif. codes:  0 '***' 0.001 '**' 0.01 '*' 0.05 '.' 0.1 ' ' 1

Residual standard error: 0.2501 on 42 degrees of freedom
Multiple R-squared:  0.9645,    Adjusted R-squared:  0.9636 
F-statistic:  1140 on 1 and 42 DF,  p-value: < 0.00000000000000022
```

**Primates excluding prosimians, to compare to other studies**

```
primates2<-b%>%
  filter(group=="Primates")%>%
  filter(species!="Perodicticus_potto")
fit.primates2<-lm(log(bm)~I(log(ocw)^(2/3)),data=primates2)
regression.stats(fit.primates2)
```

## 9.6 Australidelphia dataset

```
australidelphia<-subset(b, subset = group %in% c("Diprotodontia","Dasyuromorphia","Peramelemorphia","Microbiotheria"))
fit.australidelphia<-lm(log(bm)~I((log(ocw))^(2/3)),data=australidelphia)
par(mfrow=c(2,2))
plot(fit.australidelphia)
```

```
summary(fit.australidelphia)
```

```
Call:
lm(formula = log(bm) ~ I((log(ocw))^(2/3)), data = australidelphia)

Residuals:
     Min       1Q   Median       3Q      Max 
-0.58468 -0.32682  0.01498  0.23604  1.01547 

Coefficients:
                    Estimate Std. Error t value            Pr(>|t|)    
(Intercept)          -8.9050     0.4821  -18.47 <0.0000000000000002 ***
I((log(ocw))^(2/3))   8.1213     0.2475   32.81 <0.0000000000000002 ***
---
Signif. codes:  0 '***' 0.001 '**' 0.01 '*' 0.05 '.' 0.1 ' ' 1

Residual standard error: 0.3882 on 30 degrees of freedom
Multiple R-squared:  0.9729,    Adjusted R-squared:  0.972 
F-statistic:  1077 on 1 and 30 DF,  p-value: < 0.00000000000000022
```

## 9.7 Accuracy statistics for taxonomic subset regressions

```
rbind("All species"=regression.stats(fit.all),
      "All therians"=regression.stats(fit.therian),
      "Australidelphia"=regression.stats(fit.australidelphia),
      "Carnivora"=regression.stats(fit.carnivora),
      "Primates"=regression.stats(fit.primates),
      "Rodentia"=regression.stats(fit.rodent),
      "Sciuromorpha"=regression.stats(fit.squirrel),
      "Ungulates"=regression.stats(fit.ungulate)
      )
```

## 9.8 Regression lines for taxonomic subsets

```
ggplot(plot_data,aes(log(ocw)^(2/3),log(bm)))+
  geom_point(fill="gray",size=3,shape=21,col="black")+
  labs(x=expression(paste("Natural Log OCW (mm)"^{2/3})),y="Natural Log Body Mass (g)",show.legend=TRUE)+
  scale_x_continuous(breaks=seq(1,6,0.25))+
  scale_y_continuous(breaks=seq(0,20,1))+
  theme_classic()+
  geom_point(data=ungulate,fill="green",size=3,shape=21,col="black")+
  geom_point(data=carnivora,fill="blue",size=3,shape=21,col="black")+
  geom_point(data=rodent,fill="orange",size=3,shape=21,col="black")+
  geom_point(data=primates,fill="cyan",size=3,shape=21,col="black")+
  geom_point(data=australidelphia,fill="magenta",size=3,shape=21,col="black")+
  geom_smooth(data=carnivora,formula=y~x,col="transparent",
              fill="blue",se=T,fullrange=T,method="lm",alpha=0.1)+
  geom_smooth(data=ungulate,formula=y~x,col="transparent",
              fill="green",se=T,fullrange=T,method="lm",alpha=0.1,)+
  geom_smooth(data=rodent,formula=y~x,col="transparent",fill="orange"
              ,se=T,fullrange=T,method="lm",alpha=0.1)+
  geom_smooth(data=primates,formula=y~x,col="transparent",
              fill="cyan",se=T,fullrange=T,method="lm",alpha=0.1)+
  geom_smooth(data=australidelphia,formula=y~x,col="transparent",
              fill="magenta",se=T,fullrange=T,method="lm",alpha=0.1)+
  geom_smooth(formula=y~x,method="lm",se=F,
              fill="red",alpha=0.1,size=1)+  
  geom_smooth(formula=y~x,method="lm",se=F,
              aes(col="red"),size=1)+  
  geom_smooth(data=carnivora,formula=y~x,aes(col="blue"),se=F,fullrange=T
              ,method="lm")+
  geom_smooth(data=ungulate,formula=y~x,aes(col="green"),se=F,fullrange=T,
              method="lm")+
  geom_smooth(data=rodent,formula=y~x,aes(col="orange"),se=F,fullrange=T,
              method="lm")+
  geom_smooth(data=primates,formula=y~x,aes(col="cyan"),se=F,
              fullrange=T,method="lm")+
  geom_smooth(data=australidelphia,formula=y~x,aes(col="magenta"),se=F,
              fullrange=T,method="lm")+
  labs(fill="Regression",color="Regression")+
  scale_color_identity(name="Regression",labels=c(red="All Taxa",
                                                  green="Ungulates",
                                                  blue="Carnivora",
                                                  orange="Rodentia",
                                                  cyan="Primates",
                                                  magenta="Australidelphia"),
                       guide="legend")+
  guides(colour = guide_legend(reverse = T))+
  theme(legend.position=c(.8,.3),legend.spacing.y = unit(.1, "cm"))
```

Comparison of regression lines for taxonomic subsets

# 10 Examining a log-quadratic model

## 10.1 Statistical power of quadratic and cubic terms under log-quadratic and log-cubic models and summary plots

***Quadratic Model***

```
summary(fit.quadratic)
```

```
Call:
lm(formula = log(bm) ~ I(log(ocw)^2) + log(ocw), data = b)

Residuals:
     Min       1Q   Median       3Q      Max 
-1.32377 -0.24474 -0.02902  0.18794  1.13162 

Coefficients:
              Estimate Std. Error t value             Pr(>|t|)    
(Intercept)   -4.73010    0.22735 -20.806 < 0.0000000000000002 ***
I(log(ocw)^2) -0.19078    0.02584  -7.384    0.000000000000899 ***
log(ocw)       4.74897    0.15632  30.380 < 0.0000000000000002 ***
---
Signif. codes:  0 '***' 0.001 '**' 0.01 '*' 0.05 '.' 0.1 ' ' 1

Residual standard error: 0.3902 on 401 degrees of freedom
Multiple R-squared:  0.9811,    Adjusted R-squared:  0.981 
F-statistic: 1.039e+04 on 2 and 401 DF,  p-value: < 0.00000000000000022
```

```
par(mfrow=c(2,2))
plot(fit.quadratic)
```

***ANOVA of Log-Quadratic Model versus 2/3 Power Model***

```
anova(fit.all,fit.quadratic)
```

***Cubic Model***

```
summary(fit.cubic)
```

```
Call:
lm(formula = log(bm) ~ I(log(ocw)^3) + I(log(ocw)^2) + log(ocw), 
    data = b)

Residuals:
    Min      1Q  Median      3Q     Max 
-1.3183 -0.2417 -0.0277  0.1899  1.1285 

Coefficients:
              Estimate Std. Error t value          Pr(>|t|)    
(Intercept)   -4.99082    0.65539  -7.615 0.000000000000192 ***
I(log(ocw)^3)  0.01007    0.02373   0.424             0.672    
I(log(ocw)^2) -0.28559    0.22499  -1.269             0.205    
log(ocw)       5.03025    0.68129   7.383 0.000000000000904 ***
---
Signif. codes:  0 '***' 0.001 '**' 0.01 '*' 0.05 '.' 0.1 ' ' 1

Residual standard error: 0.3906 on 400 degrees of freedom
Multiple R-squared:  0.9811,    Adjusted R-squared:  0.9809 
F-statistic:  6913 on 3 and 400 DF,  p-value: < 0.00000000000000022
```

```
par(mfrow=c(2,2))
plot(fit.cubic)
```

## 10.2 Plot of leverage versus fitted values for log-quadratic model

```
grid.arrange(ncol=3,
data.frame(leverage=hatvalues(fit.line),fitted=fit.line$fitted.values)%>%
  ggplot(aes(y=leverage,x=fitted))+
  geom_point()+
  labs(x="Fitted Values (g)",y="Leverage")+
  theme_classic()+
  ggtitle("A"),
data.frame(leverage=hatvalues(fit.all),fitted=fit.all$fitted.values)%>%
  ggplot(aes(y=leverage,x=fitted))+
  geom_point()+
  labs(x="Fitted Values (g)",y="Leverage")+
  theme_classic()+
  ggtitle("B"),
data.frame(leverage=hatvalues(fit.quadratic),fitted=fit.quadratic$fitted.values)%>%
  ggplot(aes(y=leverage,x=fitted))+
  geom_point()+
  labs(x="Fitted Values (g)",y="Leverage")+
  theme_classic()+
  ggtitle("C")
)
```

Plot of leverages versus fitted values for (**A**) log-linear model, (**B**) a log-2/3 power model, and (**C**) a log-quadratic model, showing how only the most extreme values in the log-quadratic model have any influence over the curve.

## 10.3 Log-quadratic fit lines for different orders

```
ggplot(plot_data %>% group_by(group) %>% filter(n() > 4),
       aes(log(ocw),log(bm),fill=group,color=group))+
  geom_point(data=plot_data,fill="gray",size=3,shape=21,col="black")+
  geom_point(size=3,shape=21,col="black")+
  geom_smooth(formula=y~poly(x,2),method="lm",alpha=0.2)+
  stat_function(fun=function(x) fit.quadratic$coefficients[1]+
                                  fit.quadratic$coefficients[2]*I(x^2)+
                                  fit.quadratic$coefficients[3]*x,
                  col="black",linetype="dashed",size=0.75)+
  labs(x="Natural Log OCW (mm)",y="Natural Log Body Mass (g)",show.legend=TRUE)+
  scale_x_continuous(breaks=seq(1,6,0.5))+
  scale_y_continuous(breaks=seq(0,20,1))+
  theme_classic()+
  labs(fill="Regression",color="Regression")+
  guides(color=guide_legend(ncol=2),fill=guide_legend(ncol=2))+
  theme(legend.position=c(.8,.3),legend.spacing.y = unit(.1, "cm"))
```

Comparison of log-quadratic regression models by order, for all groups of N = 4

## 10.4 Plotting log-quadratic model comparing rabbit-like taxa to non-rabbit-like taxa

```
lm_eqn_quadratic1 <- function(plot_rabbit){
    m <- lm(I(log(bm)) ~ I(log(ocw)^2)+log(ocw), data=subset(plot_rabbit,group2=='Rabbit-Like Occiput'));
    eq <- substitute(italic("ln(body mass)") == a + b %.% italic("ln(OCW)"^{2})*+ c %.%italic("ln(OCW)"), 
                     list(a = format(unname(coef(m)[1]), digits = 5),
                          b = format(unname(coef(m)[2]), digits = 5),
                          c = format(unname(coef(m)[3]), digits = 5),
                          r2 = format(summary(m)$r.squared, digits = 3)))
    as.character(as.expression(eq));
}    
lm_eqn_quadratic2 <- function(plot_rabbit){
    m <- lm(I(log(bm)) ~ I(log(ocw)^2)+log(ocw), data=subset(plot_rabbit,group2=='Rabbit-Like Occiput'));
    eq <- substitute(italic(r)^2~"="~r2, 
                     list(r2 = format(summary(m)$r.squared, digits = 3)))
    as.character(as.expression(eq));
}    
(scatterplot.rabbit.quadratic<-ggplot(plot_rabbit,aes(log(ocw),log(bm)))+
    geom_point(size=3,col="black",aes(fill=group2,shape=group2))+
    geom_point(data=subset(plot_rabbit,group2!='Other'),size=3,col="black"                ,aes(fill=group2,shape=group2))+
    geom_smooth(data=plot_rabbit %>%
                  filter(group2=="Other"|group2=='Rabbit-Like Occiput'), 
                aes(group=group2), color="transparent", formula=y~I(x^2)+x, method="lm",
                level=0.9)+
    geom_smooth(aes(group=group2,color=group2),
               formula=y~I(x^2)+x,method="lm",se=F,level=0.9)+
    labs(x= expression(paste("Natural Log OCW (mm)")),
         y="Natural Log Body Mass (g)")+
    theme_classic()+
    scale_x_continuous(breaks=seq(1,6,0.5))+
    scale_y_continuous(breaks=seq(0,17,1))+
    scale_shape_manual(values=c(22,24,21,23))+
    scale_fill_manual(values=c("red", "blue","light gray","yellow"))+
    scale_color_manual(values=c(NA, NA,"blue","red"),na.value=NA)+
    theme(legend.position = c(0.89,0.2))+
    labs(fill="Group",shape="Group",color="Group")+
    annotate("text",label = "Rabbit-Like Taxa Only",hjust=0, parse = FALSE,
             x = 1.2, y = 16.5)+
    geom_text(aes(x = 1.2, y = 15.5, label = lm_eqn_quadratic1(plot_rabbit)),
              hjust=0,parse = TRUE,data.frame())+
    geom_text(aes(x = 1.2, y = 14.5, label = lm_eqn_quadratic2(plot_rabbit)),
              hjust=0,parse = TRUE,data.frame()))
```

Comparing differences in log-quadratic regression model between taxa with lagomorph-like occiputs and those without

## 10.5 Log-quadratic fit lines for taxonomic subgroups

```
ggplot(plot_data,aes(log(ocw),log(bm)))+
  geom_point(fill="gray",size=3,shape=21,col="black")+
  labs(x="Natural Log OCW (mm)",y="Natural Log Body Mass (g)",show.legend=TRUE)+
  scale_x_continuous(breaks=seq(1,6,0.5))+
  scale_y_continuous(breaks=seq(-3,20,1))+
  theme_classic()+
  geom_point(data=ungulate,fill="green",size=3,shape=21,col="black")+
  geom_point(data=carnivora,fill="blue",size=3,shape=21,col="black")+
  geom_point(data=rodent,fill="orange",size=3,shape=21,col="black")+
  geom_point(data=primates,fill="cyan",size=3,shape=21,col="black")+
  geom_point(data=australidelphia,fill="magenta",size=3,shape=21,col="black")+
  geom_smooth(data=carnivora,formula=y~poly(x,2),col="transparent",
              fill="blue",se=T,fullrange=T,method="lm",alpha=0.1)+
  geom_smooth(data=ungulate,formula=y~poly(x,2),col="transparent",
              fill="green",se=T,fullrange=T,method="lm",alpha=0.1,)+
  geom_smooth(data=rodent,formula=y~poly(x,2),col="transparent",fill="orange"
              ,se=T,fullrange=T,method="lm",alpha=0.1)+
  geom_smooth(data=primates,formula=y~poly(x,2),col="transparent",
              fill="cyan",se=T,fullrange=T,method="lm",alpha=0.1)+
  geom_smooth(data=australidelphia,formula=y~poly(x,2),col="transparent",
              fill="magenta",se=T,fullrange=T,method="lm",alpha=0.1)+
  geom_smooth(formula=y~poly(x,2),method="lm",se=F,
              fill="red",alpha=0.1,size=1)+  
  geom_smooth(formula=y~poly(x,2),method="lm",se=F,
              aes(col="red"),size=1)+  
  geom_smooth(data=carnivora,formula=y~poly(x,2),aes(col="blue"),se=F,fullrange=T
              ,method="lm")+
  geom_smooth(data=ungulate,formula=y~poly(x,2),aes(col="green"),se=F,fullrange=T,
              method="lm")+
  geom_smooth(data=rodent,formula=y~poly(x,2),aes(col="orange"),se=F,fullrange=T,
              method="lm")+
  geom_smooth(data=primates,formula=y~poly(x,2),aes(col="cyan"),se=F,
              fullrange=T,method="lm")+
  geom_smooth(data=australidelphia,formula=y~poly(x,2),aes(col="magenta"),se=F,
              fullrange=T,method="lm")+
  labs(fill="Regression",color="Regression")+
  scale_color_identity(name="Regression",labels=c(red="All Taxa",
                                                  green="Ungulates",
                                                  blue="Carnivora",
                                                  orange="Rodentia",
                                                  cyan="Primates",
                                                  magenta="Australidelphia"),
                       guide="legend")+
  guides(colour = guide_legend(reverse = T))+
  theme(legend.position=c(.8,.3),legend.spacing.y = unit(.1, "cm"))
```

Comparison of log-quadratic regression models for australidelphians, carnivorans, primates, rodents, and ungulates compared to that for all mammals

### 10.5.1 Plot of log-quadratic regression models for Sciuromorpha compared to Rodentia and Mammalia

```
ggplot(plot_data,aes(log(ocw),log(bm)))+
  geom_point(fill="gray",size=3,shape=21,col="black")+
  labs(x="Natural Log OCW (mm)",y="Natural Log Body Mass (g)",show.legend=TRUE)+
  scale_x_continuous(breaks=seq(1,6,0.5))+
  scale_y_continuous(breaks=seq(0,28,2))+
  theme_classic()+
  geom_smooth(formula=y~I(x^2)+x,method="lm",se=F,
              aes(col="red"),size=1)+  
  geom_point(aes(fill="transparent"),size=3,shape=21,col="transparent")+
  geom_point(data=rodent,aes(fill="green"),size=3,shape=21,col="black")+
  geom_point(data=squirrel,aes(fill="blue"),size=3,shape=21,col="black")+
  geom_smooth(data=squirrel,formula=y~poly(x,2),aes(col="blue",fill="blue"),
              alpha=0.2,size=1,method="lm",fullrange=TRUE)+
  geom_smooth(data=rodent,formula=y~poly(x,2),aes(col="green",fill="green"),
              alpha=0.2,size=1,method="lm",fullrange=TRUE)+
  labs(fill="Regression",color="Regression")+
  scale_color_identity(name="Regression",labels=c(red="All Taxa",
                                                  green="Rodentia",
                                                  blue="Sciuromorpha"),
                       guide="legend")+
    scale_fill_identity(name="Regression",labels=c(transparent="All Taxa",
                                                  green="Rodentia",
                                                  blue="Sciuromorpha"),
                       guide="legend")+
  guides(colour = guide_legend(reverse = T),fill = guide_legend(reverse = T))+
  theme(legend.position=c(.8,.2),legend.spacing.y = unit(.1, "cm"))
```

Comparison of log-quadratic regression models for all mammals, all rodents, and Sciuromorpha, showing how the narrow range of x values under a log-quadratic model for Sciuromorpha results in a poorer fit

## 10.6 Testing second-order term for various taxonomic groups

```
summary(lm(log(bm)~I(log(ocw)^2)+log(ocw),data=australidelphia))
```

```
Call:
lm(formula = log(bm) ~ I(log(ocw)^2) + log(ocw), data = australidelphia)

Residuals:
     Min       1Q   Median       3Q      Max 
-0.58442 -0.27971 -0.02904  0.18384  0.94903 

Coefficients:
              Estimate Std. Error t value    Pr(>|t|)    
(Intercept)    -7.0402     1.3262  -5.309 0.000010757 ***
I(log(ocw)^2)  -0.4803     0.1986  -2.418      0.0221 *  
log(ocw)        6.4654     1.0426   6.201 0.000000919 ***
---
Signif. codes:  0 '***' 0.001 '**' 0.01 '*' 0.05 '.' 0.1 ' ' 1

Residual standard error: 0.383 on 29 degrees of freedom
Multiple R-squared:  0.9745,    Adjusted R-squared:  0.9727 
F-statistic:   554 on 2 and 29 DF,  p-value: < 0.00000000000000022
```

```
summary(lm(log(bm)~I(log(ocw)^2)+log(ocw),data=carnivora))
```

```
Call:
lm(formula = log(bm) ~ I(log(ocw)^2) + log(ocw), data = carnivora)

Residuals:
     Min       1Q   Median       3Q      Max 
-0.89294 -0.16110  0.03026  0.18395  0.70199 

Coefficients:
              Estimate Std. Error t value       Pr(>|t|)    
(Intercept)    -7.1452     1.3179  -5.422 0.000000639370 ***
I(log(ocw)^2)  -0.2935     0.1215  -2.415         0.0181 *  
log(ocw)        5.7949     0.8055   7.194 0.000000000336 ***
---
Signif. codes:  0 '***' 0.001 '**' 0.01 '*' 0.05 '.' 0.1 ' ' 1

Residual standard error: 0.2908 on 78 degrees of freedom
Multiple R-squared:  0.9755,    Adjusted R-squared:  0.9748 
F-statistic:  1550 on 2 and 78 DF,  p-value: < 0.00000000000000022
```

```
summary(lm(log(bm)~I(log(ocw)^2)+log(ocw),data=primates))
```

```
Call:
lm(formula = log(bm) ~ I(log(ocw)^2) + log(ocw), data = primates)

Residuals:
     Min       1Q   Median       3Q      Max 
-0.71631 -0.15155  0.02928  0.14533  0.48109 

Coefficients:
              Estimate Std. Error t value Pr(>|t|)  
(Intercept)   -4.02595    2.35703  -1.708   0.0952 .
I(log(ocw)^2) -0.03301    0.23782  -0.139   0.8903  
log(ocw)       3.98416    1.50156   2.653   0.0113 *
---
Signif. codes:  0 '***' 0.001 '**' 0.01 '*' 0.05 '.' 0.1 ' ' 1

Residual standard error: 0.2512 on 41 degrees of freedom
Multiple R-squared:  0.965, Adjusted R-squared:  0.9633 
F-statistic:   565 on 2 and 41 DF,  p-value: < 0.00000000000000022
```

```
summary(lm(log(bm)~I(log(ocw)^2)+log(ocw),data=rodent))
```

```
Call:
lm(formula = log(bm) ~ I(log(ocw)^2) + log(ocw), data = rodent)

Residuals:
     Min       1Q   Median       3Q      Max 
-0.74602 -0.24551 -0.05912  0.20982  0.78589 

Coefficients:
              Estimate Std. Error t value        Pr(>|t|)    
(Intercept)    -4.8314     0.7689  -6.283 0.0000000105597 ***
I(log(ocw)^2)  -0.2035     0.1323  -1.538           0.127    
log(ocw)        4.8913     0.6486   7.542 0.0000000000303 ***
---
Signif. codes:  0 '***' 0.001 '**' 0.01 '*' 0.05 '.' 0.1 ' ' 1

Residual standard error: 0.3441 on 93 degrees of freedom
Multiple R-squared:  0.9713,    Adjusted R-squared:  0.9707 
F-statistic:  1573 on 2 and 93 DF,  p-value: < 0.00000000000000022
```

```
summary(lm(log(bm)~I(log(ocw)^2)+log(ocw),data=squirrel))
```

```
Call:
lm(formula = log(bm) ~ I(log(ocw)^2) + log(ocw), data = squirrel)

Residuals:
     Min       1Q   Median       3Q      Max 
-0.30320 -0.16365 -0.01766  0.15008  0.43141 

Coefficients:
              Estimate Std. Error t value Pr(>|t|)
(Intercept)    -1.5850     2.4380  -0.650    0.521
I(log(ocw)^2)   0.4097     0.3886   1.054    0.301
log(ocw)        2.0000     1.9570   1.022    0.316

Residual standard error: 0.2035 on 26 degrees of freedom
Multiple R-squared:  0.9763,    Adjusted R-squared:  0.9745 
F-statistic: 535.9 on 2 and 26 DF,  p-value: < 0.00000000000000022
```

```
summary(lm(log(bm)~I(log(ocw)^2)+log(ocw),data=ungulate))
```

```
Call:
lm(formula = log(bm) ~ I(log(ocw)^2) + log(ocw), data = ungulate)

Residuals:
     Min       1Q   Median       3Q      Max 
-0.64104 -0.24466 -0.06775  0.14091  1.09226 

Coefficients:
              Estimate Std. Error t value   Pr(>|t|)    
(Intercept)    -5.7800     2.0570  -2.810    0.00674 ** 
I(log(ocw)^2)  -0.2695     0.1402  -1.922    0.05950 .  
log(ocw)        5.3329     1.0801   4.937 0.00000705 ***
---
Signif. codes:  0 '***' 0.001 '**' 0.01 '*' 0.05 '.' 0.1 ' ' 1

Residual standard error: 0.3367 on 58 degrees of freedom
Multiple R-squared:  0.963, Adjusted R-squared:  0.9618 
F-statistic: 755.5 on 2 and 58 DF,  p-value: < 0.00000000000000022
```

## 10.7 Log-quadratic fit lines for superorders

### 10.7.1 Testing for differences in intercept

```
summary(lm(log(bm)~superorder+I(log(ocw)^2)+log(ocw),data=b),digits=1)
```

```
Call:
lm(formula = log(bm) ~ superorder + I(log(ocw)^2) + log(ocw), 
    data = b)

Residuals:
     Min       1Q   Median       3Q      Max 
-1.02029 -0.23084 -0.03218  0.21504  1.11255 

Coefficients:
                           Estimate Std. Error t value             Pr(>|t|)    
(Intercept)                -4.97414    0.23399 -21.258 < 0.0000000000000002 ***
superorderEuarchontoglires  0.25854    0.10643   2.429               0.0156 *  
superorderLaurasiatheria    0.04692    0.10619   0.442               0.6588    
superorderMarsupialia       0.16525    0.11261   1.467               0.1430    
superorderMonotremata      -1.05514    0.23548  -4.481     0.00000974465954 ***
superorderXenarthra        -0.12287    0.15518  -0.792               0.4289    
I(log(ocw)^2)              -0.18334    0.02482  -7.388     0.00000000000089 ***
log(ocw)                    4.76473    0.14885  32.011 < 0.0000000000000002 ***
---
Signif. codes:  0 '***' 0.001 '**' 0.01 '*' 0.05 '.' 0.1 ' ' 1

Residual standard error: 0.3663 on 396 degrees of freedom
Multiple R-squared:  0.9835,    Adjusted R-squared:  0.9832 
F-statistic:  3377 on 7 and 396 DF,  p-value: < 0.00000000000000022
```

```
summary(lm(log(bm)~superorder+rabbit+I(log(ocw)^2)+log(ocw),data=b),digits=1) #Adding lagomorph-like condylar morphology as additional term
```

```
Call:
lm(formula = log(bm) ~ superorder + rabbit + I(log(ocw)^2) + 
    log(ocw), data = b)

Residuals:
     Min       1Q   Median       3Q      Max 
-0.94098 -0.22038 -0.01564  0.19194  1.11178 

Coefficients:
                           Estimate Std. Error t value             Pr(>|t|)    
(Intercept)                -5.00703    0.21701 -23.073 < 0.0000000000000002 ***
superorderEuarchontoglires  0.17847    0.09918   1.799               0.0727 .  
superorderLaurasiatheria    0.04231    0.09846   0.430               0.6677    
superorderMarsupialia       0.17041    0.10442   1.632               0.1035    
superorderMonotremata      -1.05992    0.21835  -4.854  0.00000174289923131 ***
superorderXenarthra        -0.12605    0.14389  -0.876               0.3815    
rabbitYes                   0.67505    0.08336   8.098  0.00000000000000699 ***
I(log(ocw)^2)              -0.18342    0.02301  -7.972  0.00000000000001701 ***
log(ocw)                    4.77636    0.13802  34.605 < 0.0000000000000002 ***
---
Signif. codes:  0 '***' 0.001 '**' 0.01 '*' 0.05 '.' 0.1 ' ' 1

Residual standard error: 0.3397 on 395 degrees of freedom
Multiple R-squared:  0.9859,    Adjusted R-squared:  0.9856 
F-statistic:  3445 on 8 and 395 DF,  p-value: < 0.00000000000000022
```

### 10.7.2 Testing for differences in slope

```
print(summary(lm(log(bm)~superorder*(I(log(ocw)^2)+log(ocw)),data=b)),max.tbl.height="250px")
```

```
Call:
lm(formula = log(bm) ~ superorder * (I(log(ocw)^2) + log(ocw)), 
    data = b)

Residuals:
    Min      1Q  Median      3Q     Max 
-1.0068 -0.2342 -0.0280  0.1974  1.0331 

Coefficients:
                                          Estimate Std. Error t value       Pr(>|t|)    
(Intercept)                               -5.01912    1.23814  -4.054 0.000060980235 ***
superorderEuarchontoglires                -0.16170    1.35926  -0.119         0.9054    
superorderLaurasiatheria                   0.37649    1.28135   0.294         0.7691    
superorderMarsupialia                     -2.40712    1.58532  -1.518         0.1297    
superorderMonotremata                    114.84939  309.99048   0.370         0.7112    
superorderXenarthra                        9.61576   14.54768   0.661         0.5090    
I(log(ocw)^2)                             -0.18935    0.09668  -1.958         0.0509 .  
log(ocw)                                   4.79933    0.72675   6.604 0.000000000133 ***
superorderEuarchontoglires:I(log(ocw)^2)  -0.11091    0.12806  -0.866         0.3870    
superorderLaurasiatheria:I(log(ocw)^2)     0.04105    0.10257   0.400         0.6892    
superorderMarsupialia:I(log(ocw)^2)       -0.30063    0.18311  -1.642         0.1014    
superorderMonotremata:I(log(ocw)^2)        9.98600   27.86236   0.358         0.7202    
superorderXenarthra:I(log(ocw)^2)          0.89342    1.34854   0.663         0.5080    
superorderEuarchontoglires:log(ocw)        0.46391    0.85052   0.545         0.5858    
superorderLaurasiatheria:log(ocw)         -0.24342    0.75812  -0.321         0.7483    
superorderMarsupialia:log(ocw)             1.82389    1.07993   1.689         0.0920 .  
superorderMonotremata:log(ocw)           -68.10023  185.96141  -0.366         0.7144    
superorderXenarthra:log(ocw)              -5.92732    8.89030  -0.667         0.5054    
---
Signif. codes:  0 '***' 0.001 '**' 0.01 '*' 0.05 '.' 0.1 ' ' 1

Residual standard error: 0.3595 on 386 degrees of freedom
Multiple R-squared:  0.9845,    Adjusted R-squared:  0.9838 
F-statistic:  1445 on 17 and 386 DF,  p-value: < 0.00000000000000022
```

### 10.7.3 Scatterplot with regression lines

```
b <- b %>%
  mutate(superorder = case_when(
    group %in% c("Proboscidea","Afrosoricida","Hyracoidea","Tubulidentata","Macroscelidea") ~ "Afrotheria",
    group %in% c("Pilosa","Cingulata") ~ "Xenarthra",
    group %in% c("Dasyuromorphia","Didelphimorphia","Diprotodontia","Paucituberculata","Microbiotheria","Peramelemorphia") ~ "Marsupialia",
    group %in% c("Carnivora","Artiodactyla","Perissodactyla","Eulipotyphla","Pholidota") ~ "Laurasiatheria",
    group %in% c("Monotremata") ~ "Monotremata",
    group %in% c("Sciuromorpha","Primates","Castorimorpha","Anomaluromorpha","Scandentia","Hystricomorpha","Myomorpha","Lagomorpha","Dermoptera") ~ "Euarchontoglires"
    ))
(scatterplot.superordinal<-ggplot(b,aes(x=log(ocw),y=log(bm)))+
  geom_point(aes(fill=superorder),shape=21)+
  geom_smooth(formula=y~poly(x,2),color="transparent",aes(fill=superorder),
                alpha=0.1,method="lm")+
  geom_smooth(formula=y~poly(x,2),aes(color=superorder,group=superorder),
              method="lm",se=F)+
  labs(x="Natural Log OCW (mm)",y="Natural Log Body Mass (g)",color="Superorder",
       fill="Superorder")+
  theme_classic()+
  theme(legend.position = c(0.8,0.3)))
```

# 11 Individual specimen-level variation and effect of captivity status

## 11.1 Regression equation treating each specimen individually

```
nls(log(bm)~a*log(ocw)^b+c,data=e,start=list(a=1,b=1,c=1))
```

```
Nonlinear regression model
  model: log(bm) ~ a * log(ocw)^b + c
   data: e
     a      b      c 
 6.852  0.714 -7.235 
 residual sum-of-squares: 355.5

Number of iterations to convergence: 5 
Achieved convergence tolerance: 0.0000001837
```

```
confint2(nls(log(bm)~a*log(ocw)^b+c,data=e,start=list(a=1,b=1,c=1)))
```

```
       2.5 %     97.5 %
a  6.1602728  7.5440015
b  0.6714587  0.7566143
c -8.0386961 -6.4314256
```

```
fit.specimens<-lm(log(bm)~I((log(ocw))^(2/3)),data=e)
par(mfrow=c(2,2))
plot(fit.specimens)
```

```
summary(fit.specimens)
```

```
Call:
lm(formula = log(bm) ~ I((log(ocw))^(2/3)), data = e)

Residuals:
     Min       1Q   Median       3Q      Max 
-1.39583 -0.26026 -0.02176  0.25486  1.45412 

Coefficients:
                    Estimate Std. Error t value            Pr(>|t|)    
(Intercept)         -8.18617    0.04913  -166.6 <0.0000000000000002 ***
I((log(ocw))^(2/3))  7.68179    0.02530   303.6 <0.0000000000000002 ***
---
Signif. codes:  0 '***' 0.001 '**' 0.01 '*' 0.05 '.' 0.1 ' ' 1

Residual standard error: 0.4095 on 2125 degrees of freedom
Multiple R-squared:  0.9775,    Adjusted R-squared:  0.9775 
F-statistic: 9.219e+04 on 1 and 2125 DF,  p-value: < 0.00000000000000022
```

```
shapiro.test(fit.specimens$residuals)
```

```
    Shapiro-Wilk normality test

data:  fit.specimens$residuals
W = 0.99514, p-value = 0.000002007
```

```
regression.stats(fit.specimens)
```

## 11.2 Regression using species averages of wild-caught specimens only

```
wildonly<-subset(e, subset = zoo %in% c("Wild"))
wildaverage<-wildonly%>%
  group_by(species)%>%
  summarise(ocw=mean(ocw),bm=mean(bm),N=n())
(fit.wild<-nls(log(bm)~a*log(ocw)^b+c,data=wildaverage,start=list(a=1,b=1,c=1)))
```

```
Nonlinear regression model
  model: log(bm) ~ a * log(ocw)^b + c
   data: wildaverage
      a       b       c 
 7.6936  0.6631 -8.1544 
 residual sum-of-squares: 53.79

Number of iterations to convergence: 6 
Achieved convergence tolerance: 0.00000004801
```

```
confint2(nls(log(bm)~a*log(ocw)^b+c,data=wildaverage,start=list(a=1,b=1,c=1)))
```

```
        2.5 %     97.5 %
a   5.5440384  9.8431326
b   0.5508304  0.7752748
c -10.6187420 -5.6900237
```

```
AIC(fit.wild,k=2)
```

```
[1] 345.8861
```

```
fit.wild2<-lm(log(bm)~I(log(ocw)^(2/3)),data=wildaverage)
summary(fit.wild2)
```

```
Call:
lm(formula = log(bm) ~ I(log(ocw)^(2/3)), data = wildaverage)

Residuals:
     Min       1Q   Median       3Q      Max 
-1.27328 -0.21442 -0.01729  0.18917  1.13943 

Coefficients:
                  Estimate Std. Error t value            Pr(>|t|)    
(Intercept)        -8.0759     0.1261  -64.04 <0.0000000000000002 ***
I(log(ocw)^(2/3))   7.6249     0.0638  119.52 <0.0000000000000002 ***
---
Signif. codes:  0 '***' 0.001 '**' 0.01 '*' 0.05 '.' 0.1 ' ' 1

Residual standard error: 0.3954 on 344 degrees of freedom
Multiple R-squared:  0.9765,    Adjusted R-squared:  0.9764 
F-statistic: 1.429e+04 on 1 and 344 DF,  p-value: < 0.00000000000000022
```

```
regression.stats(fit.wild2)
```

## 11.3 Comparison of regression models between species-average of all specimens and species-average of all wild-caught specimens

```
anova(fit.wild,fit.all)
```

## 11.4 Test for if captivity has a significant effect

OLS by itself

```
summary(lm(log(bm) ~ I(log(ocw)^(2/3)) + zoo,data=e))
```

```
Call:
lm(formula = log(bm) ~ I(log(ocw)^(2/3)) + zoo, data = e)

Residuals:
     Min       1Q   Median       3Q      Max 
-1.37481 -0.25751 -0.01988  0.25568  1.47931 

Coefficients:
                  Estimate Std. Error  t value             Pr(>|t|)    
(Intercept)       -8.00531    0.06951 -115.166 < 0.0000000000000002 ***
I(log(ocw)^(2/3))  7.64890    0.02677  285.692 < 0.0000000000000002 ***
zooWild           -0.12809    0.03493   -3.667             0.000251 ***
---
Signif. codes:  0 '***' 0.001 '**' 0.01 '*' 0.05 '.' 0.1 ' ' 1

Residual standard error: 0.4083 on 2124 degrees of freedom
Multiple R-squared:  0.9776,    Adjusted R-squared:  0.9776 
F-statistic: 4.637e+04 on 2 and 2124 DF,  p-value: < 0.00000000000000022
```

Accounting for phylogenetic non-independence using a generalized linear mixed model.

```
f<-data.frame(subset(e,select=-c(family),animal=species))
force.ultrametric<-function(tree,method=c("nnls","extend")){
    method<-method[1]
    if(method=="nnls") tree<-nnls.tree(cophenetic(tree),tree,
                                       rooted=TRUE,trace=0)
    else if(method=="extend"){
        h<-diag(vcv(tree))
        d<-max(h)-h
        ii<-sapply(1:Ntip(tree),function(x,y) which(y==x),
                   y=tree$edge[,2])
        tree$edge.length[ii]<-tree$edge.length[ii]+d
    } else 
        cat("method not recognized: returning input tree\n\n")
    tree
}
set.seed(1)
captivity_mcmc <- MCMCglmm(fixed = log(bm) ~ I(log(ocw)^(2/3)) + zoo, 
                           random = ~ animal + specimen, 
                           family = "gaussian",
                           pedigree = force.ultrametric(trees[[1]]), 
                           data = f,
                           nitt = 100000,
                           burnin = 2000,
                           thin = 5,
                           prior = list(R = list(V=1, nu=0.002),
                                        G = list(G1 = list(V=1, nu=0.002),
                                                 G2 = list(V=1, nu=0.002))))
```

```
summary(captivity_mcmc)
```

```
 Iterations = 2001:99996
 Thinning interval  = 5
 Sample size  = 19600 

 DIC: -2307.349 

 G-structure:  ~animal

       post.mean l-95% CI u-95% CI eff.samp
animal    0.8635   0.6829    1.053     9634

               ~specimen

         post.mean l-95% CI u-95% CI eff.samp
specimen   0.02062 0.000444  0.04142     28.8

 R-structure:  ~units

      post.mean l-95% CI u-95% CI eff.samp
units   0.02227 0.000473  0.04171    28.76

 Location effects: log(bm) ~ I(log(ocw)^(2/3)) + zoo 

                  post.mean l-95% CI u-95% CI eff.samp    pMCMC    
(Intercept)        -7.90599 -8.93584 -6.92655    18759 <0.00005 ***
I(log(ocw)^(2/3))   7.34735  7.13911  7.55758    15268 <0.00005 ***
zooWild            -0.03162 -0.09307  0.03172    19600    0.325    
---
Signif. codes:  0 '***' 0.001 '**' 0.01 '*' 0.05 '.' 0.1 ' ' 1
```

## 11.5 Effects of captivity status

```
summary(glm(formula=factor(zoo)~log(bm),family = binomial(link = "logit"),data=e))
```

```
Call:
glm(formula = factor(zoo) ~ log(bm), family = binomial(link = "logit"), 
    data = e)

Deviance Residuals: 
    Min       1Q   Median       3Q      Max  
-3.0562   0.1024   0.2160   0.4094   1.3098  

Coefficients:
            Estimate Std. Error z value            Pr(>|z|)    
(Intercept)  7.32318    0.41923   17.47 <0.0000000000000002 ***
log(bm)     -0.60089    0.04438  -13.54 <0.0000000000000002 ***
---
Signif. codes:  0 '***' 0.001 '**' 0.01 '*' 0.05 '.' 0.1 ' ' 1

(Dispersion parameter for binomial family taken to be 1)

    Null deviance: 1170.39  on 2126  degrees of freedom
Residual deviance:  892.85  on 2125  degrees of freedom
AIC: 896.85

Number of Fisher Scoring iterations: 7
```

```
invisible(boxplot3<-ggplot()+
        aes(e$zoo,fit.specimens$residuals)+
        stat_boxplot(geom="errorbar",width=0.5)+
        geom_boxplot(fill="gray",outlier.shape=1,outlier.size=2)+
        labs(x="Captivity Status",y="Residuals")+
        ggtitle("A")+
        theme_classic())
invisible(boxplot4<-ggplot()+
        aes(e$zoo,log(e$bm))+
        stat_boxplot(geom="errorbar",width=0.5)+
        geom_boxplot(fill="gray",outlier.shape=1,outlier.size=2)+
        labs(x="Captivity Status",y="Natural Log Body Mass (g)")+
        ggtitle("B")+
        theme_classic())
grid.arrange(boxplot3,boxplot4,nrow=1)
```

Figure S7a-b. Additional box plots of various parameters. **A**, box plot of residuals versus captivity status for all specimens. **B**, box plot of residuals versus natural log of body mass (in g) for all specimens. See Figure S7c for remaining figure in supplementary information.

## 11.6 Examining the effect of gender

**Calculating averages by species and gender**

```
by_gender<-e %>%
  group_by(species,gender)%>%
  summarize(ocw=mean(ocw),bm=mean(bm))
```

```
`summarise()` has grouped output by 'species'. You can override using the `.groups` argument.
```

```
by_gender2 <- by_gender %>% filter(!is.na(gender)) #Dataset filtering out individuals of unknown sex
```

```
summary(lm(data=by_gender,log(bm)~I(log(ocw)^(2/3))+gender))
```

```
Call:
lm(formula = log(bm) ~ I(log(ocw)^(2/3)) + gender, data = by_gender)

Residuals:
     Min       1Q   Median       3Q      Max 
-1.39117 -0.25178 -0.03525  0.22463  1.11598 

Coefficients:
                  Estimate Std. Error t value            Pr(>|t|)    
(Intercept)       -8.24789    0.08927 -92.392 <0.0000000000000002 ***
I(log(ocw)^(2/3))  7.71241    0.04393 175.579 <0.0000000000000002 ***
genderMale         0.01723    0.03123   0.552               0.581    
---
Signif. codes:  0 '***' 0.001 '**' 0.01 '*' 0.05 '.' 0.1 ' ' 1

Residual standard error: 0.4033 on 665 degrees of freedom
  (17 observations deleted due to missingness)
Multiple R-squared:  0.9789,    Adjusted R-squared:  0.9788 
F-statistic: 1.544e+04 on 2 and 665 DF,  p-value: < 0.00000000000000022
```

```
regression.stats(lm(data=by_gender2,
                    log(bm)~I(log(ocw)^(2/3))+gender)) #Regression considering separate male and female species-averages wtih gender as an additional factor variable
```

```
regression.stats(lm(data=by_gender,log(bm)~I(log(ocw)^(2/3)))) #Regression considering separate male and female species-averages
```

# 12 Testing for relative brain size as potential confounding variable

## 12.1 Subsetting data

```
braindataframe<-b[!is.na(b$brainmass),]
```

## 12.2 Control OCW regression only for taxa that have brain mass (but without brainmass included)

```
fit.brain.control<-lm(log(bm)~I(log(ocw)^(2/3)),data=braindataframe)
summary(fit.brain.control)
```

```
Call:
lm(formula = log(bm) ~ I(log(ocw)^(2/3)), data = braindataframe)

Residuals:
     Min       1Q   Median       3Q      Max 
-1.24330 -0.24581 -0.02105  0.20321  1.10817 

Coefficients:
                  Estimate Std. Error t value            Pr(>|t|)    
(Intercept)       -8.08812    0.12618   -64.1 <0.0000000000000002 ***
I(log(ocw)^(2/3))  7.65044    0.06037   126.7 <0.0000000000000002 ***
---
Signif. codes:  0 '***' 0.001 '**' 0.01 '*' 0.05 '.' 0.1 ' ' 1

Residual standard error: 0.3844 on 321 degrees of freedom
Multiple R-squared:  0.9804,    Adjusted R-squared:  0.9803 
F-statistic: 1.606e+04 on 1 and 321 DF,  p-value: < 0.00000000000000022
```

## 12.3 Testing relative brain size against residuals of OCW regression

```
fit.eq<-lm(log(brainmass)~log(bm),data=braindataframe)
summary(lm(fit.eq$residuals~fit.brain.control$residuals))
```

```
Call:
lm(formula = fit.eq$residuals ~ fit.brain.control$residuals)

Residuals:
     Min       1Q   Median       3Q      Max 
-1.32454 -0.27262 -0.01632  0.25469  1.46088 

Coefficients:
                                           Estimate              Std. Error t value            Pr(>|t|)    
(Intercept)                 -0.00000000000000004567  0.02483641096023889508   0.000                   1    
fit.brain.control$residuals -0.53349871516256175408  0.06480725921695124070  -8.232 0.00000000000000468 ***
---
Signif. codes:  0 '***' 0.001 '**' 0.01 '*' 0.05 '.' 0.1 ' ' 1

Residual standard error: 0.4464 on 321 degrees of freedom
Multiple R-squared:  0.1743,    Adjusted R-squared:  0.1717 
F-statistic: 67.77 on 1 and 321 DF,  p-value: 0.000000000000004682
```

## 12.4 Plot of relative brain size versus residuals of control OCW regression

```
plot_brain_data<-data.frame(eq.resid=fit.eq$residuals,control.resid=fit.brain.control$residuals)
(scatterplot.brain<-ggplot(plot_brain_data,aes(x=control.resid,y=eq.resid))+
        geom_point(size=3,shape=21,col="black",fill="light gray")+
        labs(x= expression(paste("Residuals of OCW versus Body Mass")),y="Residuals of Brain Size versus Body Mass")+
        scale_x_continuous(breaks=seq(-1.5,1.5,0.5))+
        geom_smooth(method="lm")+
        theme_classic())
```

```
`geom_smooth()` using formula 'y ~ x'
```

Figure S8. Plot of the residuals of the regression of OCW and body mass against the residuals of the regression between brain mass (scaled to the 3/4 power) and body mass, showing that the residuals in relative brain size are not strongly correlated with residuals in OCW.

## 12.5 Testing relative brain size as an extra independent variable

```
fit.brain<-lm(log(bm)~I(log(ocw)^(2/3))+log(brainmass),data=braindataframe)
summary(fit.brain)
```

```
Call:
lm(formula = log(bm) ~ I(log(ocw)^(2/3)) + log(brainmass), data = braindataframe)

Residuals:
     Min       1Q   Median       3Q      Max 
-1.07610 -0.23308 -0.03416  0.21268  1.08554 

Coefficients:
                  Estimate Std. Error t value             Pr(>|t|)    
(Intercept)       -7.03109    0.41921 -16.772 < 0.0000000000000002 ***
I(log(ocw)^(2/3))  6.96789    0.26521  26.273 < 0.0000000000000002 ***
log(brainmass)     0.12473    0.04722   2.642              0.00865 ** 
---
Signif. codes:  0 '***' 0.001 '**' 0.01 '*' 0.05 '.' 0.1 ' ' 1

Residual standard error: 0.3809 on 320 degrees of freedom
Multiple R-squared:  0.9808,    Adjusted R-squared:  0.9807 
F-statistic:  8182 on 2 and 320 DF,  p-value: < 0.00000000000000022
```

```
rbind("Without brain mass"=regression.stats(fit.brain.control),"With brain mass"=regression.stats(fit.brain))
```

### 12.5.1 Mean percent difference in body mass estimates between regression equations with and without brain mass

```
mean(abs(exp(fit.brain$fitted.values)-exp(fit.brain.control$fitted.values))/exp(fit.brain$fitted.values))
```

```
[1] 0.04337037
```

### 12.5.2 Plotting residuals with brain mass against residuals without brain mass

```
(brainslope<-ggplot(data=data.frame(brain=fit.brain$residuals,ocw=fit.brain.control$residuals),aes(ocw,brain))+
  geom_point(size=2,shape=21,col="black",fill="light gray")+
  coord_fixed()+
  labs(y="Residuals with Brain Size",x="Residuals without Brain Size")+
  geom_abline(intercept=0,slope=1,color="red")+
  geom_smooth(formula=y~x,method="lm",size=0.75)+
  theme_classic())
```

Figure S9. Plot of the residuals of the regression equation including brain mass as an independent variable and the residuals of the regression of the same data where brain mass is not included. Red line represents a line with intercept of 0 and slope of 1, blue line represents ols fit. If including brain size significantly improved estimates, it would be expected that the slope would be much shallower than 1 due to residuals for extreme values being lower.

```
summary(lm(fit.brain.control$residuals~fit.brain$residuals))
```

```
Call:
lm(formula = fit.brain.control$residuals ~ fit.brain$residuals)

Residuals:
      Min        1Q    Median        3Q       Max 
-0.171343 -0.035299 -0.001826  0.033911  0.185806 

Coefficients:
                                  Estimate             Std. Error t value            Pr(>|t|)    
(Intercept)         -0.0000000000000000556  0.0031248830539756548     0.0                   1    
fit.brain$residuals  1.0000000000000006661  0.0082423905426672398   121.3 <0.0000000000000002 ***
---
Signif. codes:  0 '***' 0.001 '**' 0.01 '*' 0.05 '.' 0.1 ' ' 1

Residual standard error: 0.05616 on 321 degrees of freedom
Multiple R-squared:  0.9787,    Adjusted R-squared:  0.9786 
F-statistic: 1.472e+04 on 1 and 321 DF,  p-value: < 0.00000000000000022
```

# 13 Test for phylogenetic signal and PGLS

## 13.1 Testing for phylogenetic signal

```
signaldataset<-data.frame(b,residuals=fit.all$residuals,row.names = (b$species))
occipitalphysig <- tree_physig(trait.col = "residuals", data = signaldataset, phy = trees, method="lambda", n.tree=100, track=TRUE)
```

```
Used dataset has  404  species that match data and phylogeny
```

```
summary(occipitalphysig)
```

```
$Call
tree_physig(trait.col = "residuals", data = signaldataset, phy = trees, 
    n.tree = 100, method = "lambda", track = TRUE)

$Summary
            mean  CI_low CI_high     min     max
estimate 0.90136 0.89999 0.90273 0.88363 0.91791
pval     0.00000 0.00000 0.00000 0.00000 0.00000
```

## 13.2 Showing variation in AIC across models

```
fit.phylo.1<-gls(log(bm)~I(log(ocw)^(2/3)),data=b,
                 correlation=corBrownian(1,trees[[1]],form=~species))
fit.phylo.2<-gls(log(bm)~I(log(ocw)^(2/3)),data=b,
                 correlation=corBrownian(1,trees[[2]],form=~species))
fit.phylo.3<-gls(log(bm)~I(log(ocw)^(2/3)),data=b,
                 correlation=corBrownian(1,trees[[3]],form=~species))
fit.phylo.4<-gls(log(bm)~I(log(ocw)^(2/3)),data=b,
                 correlation=corBrownian(1,trees[[4]],form=~species))
fit.phylo.5<-gls(log(bm)~I(log(ocw)^(2/3)),data=b,
                 correlation=corBrownian(1,trees[[5]],form=~species))
fit.phylo.6<-gls(log(bm)~I(log(ocw)^(2/3)),data=b,
                 correlation=corBrownian(1,trees[[6]],form=~species))
fit.phylo.7<-gls(log(bm)~I(log(ocw)^(2/3)),data=b,
                 correlation=corBrownian(1,trees[[7]],form=~species))
fit.phylo.8<-gls(log(bm)~I(log(ocw)^(2/3)),data=b,
                 correlation=corBrownian(1,trees[[8]],form=~species))
fit.phylo.9<-gls(log(bm)~I(log(ocw)^(2/3)),data=b,
                 correlation=corBrownian(1,trees[[9]],form=~species))
fit.phylo.10<-gls(log(bm)~I(log(ocw)^(2/3)),data=b,
                  correlation=corBrownian(1,trees[[10]],form=~species))
fit.phylo.11<-gls(log(bm)~I(log(ocw)^(2/3)),data=b,
                  correlation=corBrownian(1,trees[[11]],form=~species))
fit.phylo.12<-gls(log(bm)~I(log(ocw)^(2/3)),data=b,
                  correlation=corBrownian(1,trees[[12]],form=~species))
fit.phylo.13<-gls(log(bm)~I(log(ocw)^(2/3)),data=b,
                  correlation=corBrownian(1,trees[[13]],form=~species))
fit.phylo.14<-gls(log(bm)~I(log(ocw)^(2/3)),data=b,
                  correlation=corBrownian(1,trees[[14]],form=~species))
fit.phylo.15<-gls(log(bm)~I(log(ocw)^(2/3)),data=b,
                  correlation=corBrownian(1,trees[[15]],form=~species))
fit.phylo.16<-gls(log(bm)~I(log(ocw)^(2/3)),data=b,
                  correlation=corBrownian(1,trees[[16]],form=~species))
fit.phylo.17<-gls(log(bm)~I(log(ocw)^(2/3)),data=b,
                  correlation=corBrownian(1,trees[[17]],form=~species))
fit.phylo.18<-gls(log(bm)~I(log(ocw)^(2/3)),data=b,
                  correlation=corBrownian(1,trees[[18]],form=~species))
fit.phylo.19<-gls(log(bm)~I(log(ocw)^(2/3)),data=b,
                  correlation=corBrownian(1,trees[[19]],form=~species))
fit.phylo.20<-gls(log(bm)~I(log(ocw)^(2/3)),data=b,
                  correlation=corBrownian(1,trees[[20]],form=~species))
fit.phylo.21<-gls(log(bm)~I(log(ocw)^(2/3)),data=b,
                  correlation=corBrownian(1,trees[[21]],form=~species))
fit.phylo.22<-gls(log(bm)~I(log(ocw)^(2/3)),data=b,
                  correlation=corBrownian(1,trees[[22]],form=~species))
fit.phylo.23<-gls(log(bm)~I(log(ocw)^(2/3)),data=b,
                  correlation=corBrownian(1,trees[[23]],form=~species))
fit.phylo.24<-gls(log(bm)~I(log(ocw)^(2/3)),data=b,
                  correlation=corBrownian(1,trees[[24]],form=~species))
fit.phylo.25<-gls(log(bm)~I(log(ocw)^(2/3)),data=b,
                  correlation=corBrownian(1,trees[[25]],form=~species))
fit.phylo.25<-gls(log(bm)~I(log(ocw)^(2/3)),data=b,
                  correlation=corBrownian(1,trees[[25]],form=~species))
fit.phylo.25<-gls(log(bm)~I(log(ocw)^(2/3)),data=b,
                  correlation=corBrownian(1,trees[[25]],form=~species))
fit.phylo.26<-gls(log(bm)~I(log(ocw)^(2/3)),data=b,
                  correlation=corBrownian(1,trees[[26]],form=~species))
fit.phylo.27<-gls(log(bm)~I(log(ocw)^(2/3)),data=b,
                  correlation=corBrownian(1,trees[[27]],form=~species))
fit.phylo.28<-gls(log(bm)~I(log(ocw)^(2/3)),data=b,
                  correlation=corBrownian(1,trees[[28]],form=~species))
fit.phylo.29<-gls(log(bm)~I(log(ocw)^(2/3)),data=b,
                  correlation=corBrownian(1,trees[[29]],form=~species))
fit.phylo.30<-gls(log(bm)~I(log(ocw)^(2/3)),data=b,
                  correlation=corBrownian(1,trees[[30]],form=~species))
fit.phylo.31<-gls(log(bm)~I(log(ocw)^(2/3)),data=b,
                  correlation=corBrownian(1,trees[[31]],form=~species))
fit.phylo.32<-gls(log(bm)~I(log(ocw)^(2/3)),data=b,
                  correlation=corBrownian(1,trees[[32]],form=~species))
fit.phylo.33<-gls(log(bm)~I(log(ocw)^(2/3)),data=b,
                  correlation=corBrownian(1,trees[[33]],form=~species))
fit.phylo.34<-gls(log(bm)~I(log(ocw)^(2/3)),data=b,
                  correlation=corBrownian(1,trees[[34]],form=~species))
fit.phylo.35<-gls(log(bm)~I(log(ocw)^(2/3)),data=b,
                  correlation=corBrownian(1,trees[[35]],form=~species))
fit.phylo.36<-gls(log(bm)~I(log(ocw)^(2/3)),data=b,
                  correlation=corBrownian(1,trees[[36]],form=~species))
fit.phylo.37<-gls(log(bm)~I(log(ocw)^(2/3)),data=b,
                  correlation=corBrownian(1,trees[[37]],form=~species))
fit.phylo.38<-gls(log(bm)~I(log(ocw)^(2/3)),data=b,
                  correlation=corBrownian(1,trees[[38]],form=~species))
fit.phylo.39<-gls(log(bm)~I(log(ocw)^(2/3)),data=b,
                  correlation=corBrownian(1,trees[[39]],form=~species))
fit.phylo.40<-gls(log(bm)~I(log(ocw)^(2/3)),data=b,
                  correlation=corBrownian(1,trees[[40]],form=~species))
fit.phylo.41<-gls(log(bm)~I(log(ocw)^(2/3)),data=b,
                  correlation=corBrownian(1,trees[[41]],form=~species))
fit.phylo.42<-gls(log(bm)~I(log(ocw)^(2/3)),data=b,
                  correlation=corBrownian(1,trees[[42]],form=~species))
fit.phylo.43<-gls(log(bm)~I(log(ocw)^(2/3)),data=b,
                  correlation=corBrownian(1,trees[[43]],form=~species))
fit.phylo.44<-gls(log(bm)~I(log(ocw)^(2/3)),data=b,
                  correlation=corBrownian(1,trees[[44]],form=~species))
fit.phylo.45<-gls(log(bm)~I(log(ocw)^(2/3)),data=b,
                  correlation=corBrownian(1,trees[[45]],form=~species))
fit.phylo.46<-gls(log(bm)~I(log(ocw)^(2/3)),data=b,
                  correlation=corBrownian(1,trees[[46]],form=~species))
fit.phylo.47<-gls(log(bm)~I(log(ocw)^(2/3)),data=b,
                  correlation=corBrownian(1,trees[[47]],form=~species))
fit.phylo.48<-gls(log(bm)~I(log(ocw)^(2/3)),data=b,
                  correlation=corBrownian(1,trees[[48]],form=~species))
fit.phylo.49<-gls(log(bm)~I(log(ocw)^(2/3)),data=b,
                  correlation=corBrownian(1,trees[[49]],form=~species))
fit.phylo.50<-gls(log(bm)~I(log(ocw)^(2/3)),data=b,
                  correlation=corBrownian(1,trees[[50]],form=~species))
fit.phylo.51<-gls(log(bm)~I(log(ocw)^(2/3)),data=b,
                  correlation=corBrownian(1,trees[[51]],form=~species))
fit.phylo.52<-gls(log(bm)~I(log(ocw)^(2/3)),data=b,
                  correlation=corBrownian(1,trees[[52]],form=~species))
fit.phylo.53<-gls(log(bm)~I(log(ocw)^(2/3)),data=b,
                  correlation=corBrownian(1,trees[[53]],form=~species))
fit.phylo.54<-gls(log(bm)~I(log(ocw)^(2/3)),data=b,
                  correlation=corBrownian(1,trees[[54]],form=~species))
fit.phylo.55<-gls(log(bm)~I(log(ocw)^(2/3)),data=b,
                  correlation=corBrownian(1,trees[[55]],form=~species))
fit.phylo.56<-gls(log(bm)~I(log(ocw)^(2/3)),data=b,
                  correlation=corBrownian(1,trees[[56]],form=~species))
fit.phylo.57<-gls(log(bm)~I(log(ocw)^(2/3)),data=b,
                  correlation=corBrownian(1,trees[[57]],form=~species))
fit.phylo.58<-gls(log(bm)~I(log(ocw)^(2/3)),data=b,
                  correlation=corBrownian(1,trees[[58]],form=~species))
fit.phylo.59<-gls(log(bm)~I(log(ocw)^(2/3)),data=b,
                  correlation=corBrownian(1,trees[[59]],form=~species))
fit.phylo.60<-gls(log(bm)~I(log(ocw)^(2/3)),data=b,
                  correlation=corBrownian(1,trees[[60]],form=~species))
fit.phylo.61<-gls(log(bm)~I(log(ocw)^(2/3)),data=b,
                  correlation=corBrownian(1,trees[[61]],form=~species))
fit.phylo.62<-gls(log(bm)~I(log(ocw)^(2/3)),data=b,
                  correlation=corBrownian(1,trees[[62]],form=~species))
fit.phylo.63<-gls(log(bm)~I(log(ocw)^(2/3)),data=b,
                  correlation=corBrownian(1,trees[[63]],form=~species))
fit.phylo.64<-gls(log(bm)~I(log(ocw)^(2/3)),data=b,
                  correlation=corBrownian(1,trees[[64]],form=~species))
fit.phylo.65<-gls(log(bm)~I(log(ocw)^(2/3)),data=b,
                  correlation=corBrownian(1,trees[[65]],form=~species))
fit.phylo.66<-gls(log(bm)~I(log(ocw)^(2/3)),data=b,
                  correlation=corBrownian(1,trees[[66]],form=~species))
fit.phylo.67<-gls(log(bm)~I(log(ocw)^(2/3)),data=b,
                  correlation=corBrownian(1,trees[[67]],form=~species))
fit.phylo.68<-gls(log(bm)~I(log(ocw)^(2/3)),data=b,
                  correlation=corBrownian(1,trees[[68]],form=~species))
fit.phylo.69<-gls(log(bm)~I(log(ocw)^(2/3)),data=b,
                  correlation=corBrownian(1,trees[[69]],form=~species))
fit.phylo.70<-gls(log(bm)~I(log(ocw)^(2/3)),data=b,
                  correlation=corBrownian(1,trees[[70]],form=~species))
fit.phylo.71<-gls(log(bm)~I(log(ocw)^(2/3)),data=b,
                  correlation=corBrownian(1,trees[[71]],form=~species))
fit.phylo.72<-gls(log(bm)~I(log(ocw)^(2/3)),data=b,
                  correlation=corBrownian(1,trees[[72]],form=~species))
fit.phylo.73<-gls(log(bm)~I(log(ocw)^(2/3)),data=b,
                  correlation=corBrownian(1,trees[[73]],form=~species))
fit.phylo.74<-gls(log(bm)~I(log(ocw)^(2/3)),data=b,
                  correlation=corBrownian(1,trees[[74]],form=~species))
fit.phylo.75<-gls(log(bm)~I(log(ocw)^(2/3)),data=b,
                  correlation=corBrownian(1,trees[[75]],form=~species))
fit.phylo.76<-gls(log(bm)~I(log(ocw)^(2/3)),data=b,
                  correlation=corBrownian(1,trees[[76]],form=~species))
fit.phylo.77<-gls(log(bm)~I(log(ocw)^(2/3)),data=b,
                  correlation=corBrownian(1,trees[[77]],form=~species))
fit.phylo.78<-gls(log(bm)~I(log(ocw)^(2/3)),data=b,
                  correlation=corBrownian(1,trees[[78]],form=~species))
fit.phylo.79<-gls(log(bm)~I(log(ocw)^(2/3)),data=b,
                  correlation=corBrownian(1,trees[[79]],form=~species))
fit.phylo.80<-gls(log(bm)~I(log(ocw)^(2/3)),data=b,
                  correlation=corBrownian(1,trees[[80]],form=~species))
fit.phylo.81<-gls(log(bm)~I(log(ocw)^(2/3)),data=b,
                  correlation=corBrownian(1,trees[[81]],form=~species))
fit.phylo.82<-gls(log(bm)~I(log(ocw)^(2/3)),data=b,
                  correlation=corBrownian(1,trees[[82]],form=~species))
fit.phylo.83<-gls(log(bm)~I(log(ocw)^(2/3)),data=b,
                  correlation=corBrownian(1,trees[[83]],form=~species))
fit.phylo.84<-gls(log(bm)~I(log(ocw)^(2/3)),data=b,
                  correlation=corBrownian(1,trees[[84]],form=~species))
fit.phylo.85<-gls(log(bm)~I(log(ocw)^(2/3)),data=b,
                  correlation=corBrownian(1,trees[[85]],form=~species))
fit.phylo.86<-gls(log(bm)~I(log(ocw)^(2/3)),data=b,
                  correlation=corBrownian(1,trees[[86]],form=~species))
fit.phylo.87<-gls(log(bm)~I(log(ocw)^(2/3)),data=b,
                  correlation=corBrownian(1,trees[[87]],form=~species))
fit.phylo.88<-gls(log(bm)~I(log(ocw)^(2/3)),data=b,
                  correlation=corBrownian(1,trees[[88]],form=~species))
fit.phylo.89<-gls(log(bm)~I(log(ocw)^(2/3)),data=b,
                  correlation=corBrownian(1,trees[[89]],form=~species))
fit.phylo.90<-gls(log(bm)~I(log(ocw)^(2/3)),data=b,
                  correlation=corBrownian(1,trees[[90]],form=~species))
fit.phylo.91<-gls(log(bm)~I(log(ocw)^(2/3)),data=b,
                  correlation=corBrownian(1,trees[[91]],form=~species))
fit.phylo.92<-gls(log(bm)~I(log(ocw)^(2/3)),data=b,
                  correlation=corBrownian(1,trees[[92]],form=~species))
fit.phylo.93<-gls(log(bm)~I(log(ocw)^(2/3)),data=b,
                  correlation=corBrownian(1,trees[[93]],form=~species))
fit.phylo.94<-gls(log(bm)~I(log(ocw)^(2/3)),data=b,
                  correlation=corBrownian(1,trees[[94]],form=~species))
fit.phylo.95<-gls(log(bm)~I(log(ocw)^(2/3)),data=b,
                  correlation=corBrownian(1,trees[[95]],form=~species))
fit.phylo.96<-gls(log(bm)~I(log(ocw)^(2/3)),data=b,
                  correlation=corBrownian(1,trees[[96]],form=~species))
fit.phylo.97<-gls(log(bm)~I(log(ocw)^(2/3)),data=b,
                  correlation=corBrownian(1,trees[[97]],form=~species))
fit.phylo.98<-gls(log(bm)~I(log(ocw)^(2/3)),data=b,
                  correlation=corBrownian(1,trees[[98]],form=~species))
fit.phylo.99<-gls(log(bm)~I(log(ocw)^(2/3)),data=b,
                  correlation=corBrownian(1,trees[[99]],form=~species))
fit.phylo.100<-gls(log(bm)~I(log(ocw)^(2/3)),data=b,
                  correlation=corBrownian(1,trees[[100]],form=~species))
```

```
(temptable<-rbind(regression.stats(fit.all),
                  regression.stats.gls(fit.phylo.1),
                  regression.stats.gls(fit.phylo.2),
                  regression.stats.gls(fit.phylo.3),
                  regression.stats.gls(fit.phylo.4),
                  regression.stats.gls(fit.phylo.5),
                  regression.stats.gls(fit.phylo.6),
                  regression.stats.gls(fit.phylo.7),
                  regression.stats.gls(fit.phylo.8),
                  regression.stats.gls(fit.phylo.9),
                  regression.stats.gls(fit.phylo.10),
                  regression.stats.gls(fit.phylo.11),
                  regression.stats.gls(fit.phylo.12),
                  regression.stats.gls(fit.phylo.13),
                  regression.stats.gls(fit.phylo.14),
                  regression.stats.gls(fit.phylo.15),
                  regression.stats.gls(fit.phylo.16),
                  regression.stats.gls(fit.phylo.17),
                  regression.stats.gls(fit.phylo.18),
                  regression.stats.gls(fit.phylo.19),
                  regression.stats.gls(fit.phylo.20),
                  regression.stats.gls(fit.phylo.21),
                  regression.stats.gls(fit.phylo.22),
                  regression.stats.gls(fit.phylo.23),
                  regression.stats.gls(fit.phylo.24),
                  regression.stats.gls(fit.phylo.25),
                  regression.stats.gls(fit.phylo.26),
                  regression.stats.gls(fit.phylo.27),
                  regression.stats.gls(fit.phylo.28),
                  regression.stats.gls(fit.phylo.29),
                  regression.stats.gls(fit.phylo.30),
                  regression.stats.gls(fit.phylo.31),
                  regression.stats.gls(fit.phylo.32),
                  regression.stats.gls(fit.phylo.33),
                  regression.stats.gls(fit.phylo.34),
                  regression.stats.gls(fit.phylo.35),
                  regression.stats.gls(fit.phylo.36),
                  regression.stats.gls(fit.phylo.37),
                  regression.stats.gls(fit.phylo.38),
                  regression.stats.gls(fit.phylo.39),
                  regression.stats.gls(fit.phylo.40),
                  regression.stats.gls(fit.phylo.41),
                  regression.stats.gls(fit.phylo.42),
                  regression.stats.gls(fit.phylo.43),
                  regression.stats.gls(fit.phylo.44),
                  regression.stats.gls(fit.phylo.45),
                  regression.stats.gls(fit.phylo.46),
                  regression.stats.gls(fit.phylo.47),
                  regression.stats.gls(fit.phylo.48),
                  regression.stats.gls(fit.phylo.49),
                  regression.stats.gls(fit.phylo.50),
                  regression.stats.gls(fit.phylo.51),
                  regression.stats.gls(fit.phylo.52),
                  regression.stats.gls(fit.phylo.53),
                  regression.stats.gls(fit.phylo.54),
                  regression.stats.gls(fit.phylo.55),
                  regression.stats.gls(fit.phylo.56),
                  regression.stats.gls(fit.phylo.57),
                  regression.stats.gls(fit.phylo.58),
                  regression.stats.gls(fit.phylo.59),
                  regression.stats.gls(fit.phylo.60),
                  regression.stats.gls(fit.phylo.61),
                  regression.stats.gls(fit.phylo.62),
                  regression.stats.gls(fit.phylo.63),
                  regression.stats.gls(fit.phylo.64),
                  regression.stats.gls(fit.phylo.65),
                  regression.stats.gls(fit.phylo.66),
                  regression.stats.gls(fit.phylo.67),
                  regression.stats.gls(fit.phylo.68),
                  regression.stats.gls(fit.phylo.69),
                  regression.stats.gls(fit.phylo.70),
                  regression.stats.gls(fit.phylo.71),
                  regression.stats.gls(fit.phylo.72),
                  regression.stats.gls(fit.phylo.73),
                  regression.stats.gls(fit.phylo.74),
                  regression.stats.gls(fit.phylo.75),
                  regression.stats.gls(fit.phylo.76),
                  regression.stats.gls(fit.phylo.77),
                  regression.stats.gls(fit.phylo.78),
                  regression.stats.gls(fit.phylo.79),
                  regression.stats.gls(fit.phylo.80),
                  regression.stats.gls(fit.phylo.81),
                  regression.stats.gls(fit.phylo.82),
                  regression.stats.gls(fit.phylo.83),
                  regression.stats.gls(fit.phylo.84),
                  regression.stats.gls(fit.phylo.85),
                  regression.stats.gls(fit.phylo.86),
                  regression.stats.gls(fit.phylo.87),
                  regression.stats.gls(fit.phylo.88),
                  regression.stats.gls(fit.phylo.89),
                  regression.stats.gls(fit.phylo.90),
                  regression.stats.gls(fit.phylo.91),
                  regression.stats.gls(fit.phylo.92),
                  regression.stats.gls(fit.phylo.93),
                  regression.stats.gls(fit.phylo.94),
                  regression.stats.gls(fit.phylo.95),
                  regression.stats.gls(fit.phylo.96),
                  regression.stats.gls(fit.phylo.97),
                  regression.stats.gls(fit.phylo.97),
                  regression.stats.gls(fit.phylo.98),
                  regression.stats.gls(fit.phylo.99),
                  regression.stats.gls(fit.phylo.100)))
```

### 13.2.1 Coefficients for PGLS models under 100 random trees

```
(coefficient_table<-data.frame(rbind(coefficients(fit.all),
                  coefficients(fit.phylo.1),
                  coefficients(fit.phylo.2),
                  coefficients(fit.phylo.3),
                  coefficients(fit.phylo.4),
                  coefficients(fit.phylo.5),
                  coefficients(fit.phylo.6),
                  coefficients(fit.phylo.7),
                  coefficients(fit.phylo.8),
                  coefficients(fit.phylo.9),
                  coefficients(fit.phylo.10),
                  coefficients(fit.phylo.11),
                  coefficients(fit.phylo.12),
                  coefficients(fit.phylo.13),
                  coefficients(fit.phylo.14),
                  coefficients(fit.phylo.15),
                  coefficients(fit.phylo.16),
                  coefficients(fit.phylo.17),
                  coefficients(fit.phylo.18),
                  coefficients(fit.phylo.19),
                  coefficients(fit.phylo.20),
                  coefficients(fit.phylo.21),
                  coefficients(fit.phylo.22),
                  coefficients(fit.phylo.23),
                  coefficients(fit.phylo.24),
                  coefficients(fit.phylo.25),
                  coefficients(fit.phylo.26),
                  coefficients(fit.phylo.27),
                  coefficients(fit.phylo.28),
                  coefficients(fit.phylo.29),
                  coefficients(fit.phylo.30),
                  coefficients(fit.phylo.31),
                  coefficients(fit.phylo.32),
                  coefficients(fit.phylo.33),
                  coefficients(fit.phylo.34),
                  coefficients(fit.phylo.35),
                  coefficients(fit.phylo.36),
                  coefficients(fit.phylo.37),
                  coefficients(fit.phylo.38),
                  coefficients(fit.phylo.39),
                  coefficients(fit.phylo.40),
                  coefficients(fit.phylo.41),
                  coefficients(fit.phylo.42),
                  coefficients(fit.phylo.43),
                  coefficients(fit.phylo.44),
                  coefficients(fit.phylo.45),
                  coefficients(fit.phylo.46),
                  coefficients(fit.phylo.47),
                  coefficients(fit.phylo.48),
                  coefficients(fit.phylo.49),
                  coefficients(fit.phylo.50),
                  coefficients(fit.phylo.51),
                  coefficients(fit.phylo.52),
                  coefficients(fit.phylo.53),
                  coefficients(fit.phylo.54),
                  coefficients(fit.phylo.55),
                  coefficients(fit.phylo.56),
                  coefficients(fit.phylo.57),
                  coefficients(fit.phylo.58),
                  coefficients(fit.phylo.59),
                  coefficients(fit.phylo.60),
                  coefficients(fit.phylo.61),
                  coefficients(fit.phylo.62),
                  coefficients(fit.phylo.63),
                  coefficients(fit.phylo.64),
                  coefficients(fit.phylo.65),
                  coefficients(fit.phylo.66),
                  coefficients(fit.phylo.67),
                  coefficients(fit.phylo.68),
                  coefficients(fit.phylo.69),
                  coefficients(fit.phylo.70),
                  coefficients(fit.phylo.71),
                  coefficients(fit.phylo.72),
                  coefficients(fit.phylo.73),
                  coefficients(fit.phylo.74),
                  coefficients(fit.phylo.75),
                  coefficients(fit.phylo.76),
                  coefficients(fit.phylo.77),
                  coefficients(fit.phylo.78),
                  coefficients(fit.phylo.79),
                  coefficients(fit.phylo.80),
                  coefficients(fit.phylo.81),
                  coefficients(fit.phylo.82),
                  coefficients(fit.phylo.83),
                  coefficients(fit.phylo.84),
                  coefficients(fit.phylo.85),
                  coefficients(fit.phylo.86),
                  coefficients(fit.phylo.87),
                  coefficients(fit.phylo.88),
                  coefficients(fit.phylo.89),
                  coefficients(fit.phylo.90),
                  coefficients(fit.phylo.91),
                  coefficients(fit.phylo.92),
                  coefficients(fit.phylo.93),
                  coefficients(fit.phylo.94),
                  coefficients(fit.phylo.95),
                  coefficients(fit.phylo.96),
                  coefficients(fit.phylo.97),
                  coefficients(fit.phylo.97),
                  coefficients(fit.phylo.98),
                  coefficients(fit.phylo.99),
                  coefficients(fit.phylo.100)))%>%
  rename(y_intercept=X.Intercept.,slope=I.log.ocw...2.3..))
```

**Mean and standard deviation of PGLS coefficients minus fit.phylo.77, which is an outlier**

```
data.frame(number=c("Mean","Standard Deviation"),
           slope=c(mean(coefficient_table$slope[-c(1,78)]),
                   sd(coefficient_table$slope[-c(1,78)])),
           y_intercept=c(mean(coefficient_table$y_intercept[-c(1,78)]),
                         sd(coefficient_table$y_intercept[-c(1,78)])))%>%
  mutate(across(where(is.numeric),round,3))
```

### 13.2.2 Plotting variation in PGLS regression lines

```
plot(log(bm)~I(log(ocw)^(2/3)),xlab="Natural Log OCW (mm)^{2/3}",ylab="Natural Log Body Mass",pch=21,bg="gray",data=b)
abline(fit.all,col="red")
abline(fit.phylo.1)
abline(fit.phylo.2)
abline(fit.phylo.3)
abline(fit.phylo.4)
abline(fit.phylo.5)
abline(fit.phylo.6)
abline(fit.phylo.7)
abline(fit.phylo.8)
abline(fit.phylo.9)
abline(fit.phylo.10)
abline(fit.phylo.11)
abline(fit.phylo.12)
abline(fit.phylo.13)
abline(fit.phylo.14)
abline(fit.phylo.15)
abline(fit.phylo.16)
abline(fit.phylo.17)
abline(fit.phylo.18)
abline(fit.phylo.19)
abline(fit.phylo.20)
abline(fit.phylo.21)
abline(fit.phylo.22)
abline(fit.phylo.23)
abline(fit.phylo.24)
abline(fit.phylo.25)
abline(fit.phylo.26)
abline(fit.phylo.27)
abline(fit.phylo.28)
abline(fit.phylo.29)
abline(fit.phylo.30)
abline(fit.phylo.31)
abline(fit.phylo.32)
abline(fit.phylo.33)
abline(fit.phylo.34)
abline(fit.phylo.35)
abline(fit.phylo.36)
abline(fit.phylo.37)
abline(fit.phylo.38)
abline(fit.phylo.39)
abline(fit.phylo.40)
abline(fit.phylo.41)
abline(fit.phylo.42)
abline(fit.phylo.43)
abline(fit.phylo.44)
abline(fit.phylo.45)
abline(fit.phylo.46)
abline(fit.phylo.47)
abline(fit.phylo.48)
abline(fit.phylo.49)
abline(fit.phylo.50)
abline(fit.phylo.51)
abline(fit.phylo.52)
abline(fit.phylo.53)
abline(fit.phylo.54)
abline(fit.phylo.55)
abline(fit.phylo.56)
abline(fit.phylo.57)
abline(fit.phylo.58)
abline(fit.phylo.59)
abline(fit.phylo.60)
abline(fit.phylo.61)
abline(fit.phylo.62)
abline(fit.phylo.63)
abline(fit.phylo.64)
abline(fit.phylo.65)
abline(fit.phylo.66)
abline(fit.phylo.67)
abline(fit.phylo.68)
abline(fit.phylo.69)
abline(fit.phylo.70)
abline(fit.phylo.71)
abline(fit.phylo.72)
abline(fit.phylo.73)
abline(fit.phylo.74)
abline(fit.phylo.75)
abline(fit.phylo.76)
abline(fit.phylo.77)
abline(fit.phylo.78)
abline(fit.phylo.79)
abline(fit.phylo.80)
abline(fit.phylo.81)
abline(fit.phylo.82)
abline(fit.phylo.83)
abline(fit.phylo.84)
abline(fit.phylo.85)
abline(fit.phylo.86)
abline(fit.phylo.87)
abline(fit.phylo.88)
abline(fit.phylo.89)
abline(fit.phylo.90)
abline(fit.phylo.91)
abline(fit.phylo.92)
abline(fit.phylo.93)
abline(fit.phylo.94)
abline(fit.phylo.95)
abline(fit.phylo.96)
abline(fit.phylo.97)
abline(fit.phylo.97)
abline(fit.phylo.98)
abline(fit.phylo.99)
abline(fit.phylo.100)
```

Plot of best-fit regression lines using PGLS under 100 random trees. Best fit OLS line provided in red.

### 13.2.3 Plotting distribution of AIC values

```
ggplot(temptable[-c(1)],aes(x=AIC))+
  geom_histogram(col="black",fill="gray",bins=30)+
  theme_classic()+
  labs(y="Count")+
  geom_vline(xintercept=mean(temptable$AIC[-c(1)]),lty=2,col="black")+
  geom_vline(xintercept=temptable$AIC[1],lty=2,col="red")+
  annotate("text",label="OLS",x=temptable$AIC[1]-3,y=3.8,angle=90,col="red")+
  annotate("text",label="PGLS",x=mean(temptable$AIC[-c(1)])-3,y=3.8,angle=90,col="black")
```

```
plot(AIC~PE,temptable)
```

Correlation between AIC and percent error (PE) across the different PGLS models

```
plot(AIC~PE,temptable,ylim=c(330,500),xlim=c(62,75))
```

Correlation between AIC and percent error (PE) across the different PGLS models

**Comparison of AIC across models**

```
data.frame(ols=temptable$AIC[1],
           mean.pgls=mean(temptable$AIC[-c(1)]),
           median.pgls=median(temptable$AIC[-c(1)]),
           sd.pgls=sd(temptable$AIC[-c(1)]),
           min=min(temptable$AIC[-c(1)]),
           max=max(temptable$AIC[-c(1)]))
```

**Comparison of BIC across models**

```
data.frame(ols=temptable$BIC[1],
           mean.pgls=mean(temptable$BIC[-c(1)]),
           median.pgls=median(temptable$BIC[-c(1)]),
           sd.pgls=sd(temptable$BIC[-c(1)]),
           min=min(temptable$BIC[-c(1)]),
           max=max(temptable$BIC[-c(1)]))
```

## 13.3 Visualizing phylogenetic signal of the residuals across tree

```
mapdata<-data.frame("residuals"=signaldataset$residuals,row.names=b$species)
mapdata<-setNames(mapdata$residuals,rownames(mapdata))
residualsignalfit<-fastAnc(trees[[1]],mapdata,vars=TRUE,CI=TRUE)
obj<-contMap(trees[[1]],mapdata,plot=FALSE)
condyletree<-plot(obj,type="fan",fsize=0.8,ftype="bi")
```

Figure S7. Residuals of the all-species regression equation plotted onto a phylogeny of the examined taxa. Higher than expected body masses are shown in reds and yellows and lower than expected body masses are shown in cyans and blues. There is very little variation in the residuals across most of the model, suggesting a lack of Brownian motion in the evolution of this trait, but there are extreme shifts in residual values at the base of several clades such as Lagomorpha and Monotremata.

## 13.4 Phylogenetic generalized least squares (PGLS)

```
fit.phylo<-gls(log(bm)~I(log(ocw)^(2/3)),data=b,
               correlation=corBrownian(1,trees[[1]],form=~species))
summary(fit.phylo)
```

```
Generalized least squares fit by REML
  Model: log(bm) ~ I(log(ocw)^(2/3)) 
  Data: b 
       AIC      BIC    logLik
  368.3649 380.3542 -181.1824

Correlation Structure: corBrownian
 Formula: ~species 
 Parameter estimate(s):
numeric(0)

Coefficients:
                      Value Std.Error   t-value p-value
(Intercept)       -9.095213 0.7046457 -12.90750       0
I(log(ocw)^(2/3))  7.933560 0.1233222  64.33198       0

 Correlation: 
                  (Intr)
I(log(ocw)^(2/3)) -0.351

Standardized residuals:
       Min         Q1        Med         Q3        Max 
-0.7251460  0.1209105  0.2926720  0.4908711  1.1713069 

Residual standard error: 1.324389 
Degrees of freedom: 404 total; 402 residual
```

```
regression.stats.gls(fit.phylo)
```

## 13.5 Under Ornstein-Uhlenbeck model

```
fit.phylo.ou<-gls(log(bm)~I(log(ocw)^(2/3)),data=signaldataset,
                correlation=corMartins(1,trees[[1]],form=~species,fixed=TRUE))
summary(fit.phylo.ou)
```

```
Generalized least squares fit by REML
  Model: log(bm) ~ I(log(ocw)^(2/3)) 
  Data: signaldataset 
       AIC      BIC    logLik
  393.7831 405.7725 -193.8916

Correlation Structure: corMartins
 Formula: ~species 
 Parameter estimate(s):
alpha 
    1 

Coefficients:
                      Value  Std.Error   t-value p-value
(Intercept)       -8.191059 0.10960486 -74.73263       0
I(log(ocw)^(2/3))  7.691496 0.05373533 143.13667       0

 Correlation: 
                  (Intr)
I(log(ocw)^(2/3)) -0.984

Standardized residuals:
        Min          Q1         Med          Q3         Max 
-3.37487122 -0.60620062 -0.06094444  0.51919309  2.88168075 

Residual standard error: 0.3883114 
Degrees of freedom: 404 total; 402 residual
```

```
regression.stats.gls(fit.phylo.ou)
```

## 13.6 PGLS excluding Monotremata

```
therian<-b[b$group!="Monotremata",]
fit.therian<-lm(data=therian,log(bm)~I(log(ocw)^(2/3)))
trees.theria<-lapply(trees,drop.tip,tip=c("Ornithorhynchus_anatinus","Tachyglossus_aculeatus"))
class(trees.theria)<-"multiPhylo"
fit.phylo.therian<-gls(log(bm)~I(log(ocw)^(2/3)),data=therian,correlation=corBrownian(1,trees.theria[[1]],form=~species))
mean(abs(exp(fit.phylo.therian$fitted)-therian$bm)/exp(fit.phylo.therian$fitted))
signaldataset.therian<-data.frame(therian,residuals=fit.therian$residuals,row.names = (therian$species))
occipitalphysig.therian<- tree_physig(trait.col = "residuals", data = signaldataset.therian, phy = trees.theria, method="lambda", n.tree=100, track=TRUE)
```

```
Warning in match_dataphy(get(trait.col) ~ 1, data, phy): Some phylo tips do not match species in data (this can be due to NA removal) species were dropped from phylogeny or data
```

```
Used dataset has  401  species that match data and phylogeny
```

```
summary(occipitalphysig.therian)
```

```
$Call
tree_physig(trait.col = "residuals", data = signaldataset.therian, 
    phy = trees.theria, n.tree = 100, method = "lambda", track = TRUE)

$Summary
            mean  CI_low CI_high    min     max
estimate 0.88422 0.88318 0.88526 0.8726 0.89565
pval     0.00000 0.00000 0.00000 0.0000 0.00000
```

## 13.7 Scatterplot of PGLS regression versus OLS regression

```
plot_data.phylo<-data.frame(signaldataset,predict(fit.phylo,interval="prediction"))
(scatterplot.phylo<-ggplot(plot_data.phylo,aes(log(ocw)^(2/3),log(bm)))+
    geom_point(aes(group=monotreme,fill=monotreme),size=3,shape=21,col="black")+
    labs(x= expression(paste("Natural Log OCW (mm)"^{2/3})),y="Natural Log Body Mass (g)",show.legend=TRUE)+
    scale_x_continuous(breaks=seq(1,3.25,0.25))+
    scale_y_continuous(breaks=seq(0,17,1))+
    geom_point(data=b[b$group=="Monotremata",],aes(log(ocw)^(2/3),log(bm)),shape=21,size=4,fill="black",col="white")+
    theme_classic()+
    geom_segment(aes(x=min(log(b$ocw)^(2/3)),xend=max(log(b$ocw)^(2/3)),
                     y=predict(fit.all,new=data.frame(ocw=min(b$ocw))),
                     yend=predict(fit.all,new=data.frame(ocw=max(b$ocw))),
                     col="red"),size=1)+
    geom_segment(x=min(log(b$ocw)^(2/3)),xend=max(log(b$ocw)^(2/3)),
                 y=min(fit.phylo$fitted),yend=max(fit.phylo$fitted),
                 aes(col="blue"),size=1)+
    geom_segment(aes(x=min(log(b$ocw)^(2/3)),xend=max(log(b$ocw)^(2/3)),
                     y=min(fit.phylo.therian$fitted),
                     yend=max(fit.phylo.therian$fitted),colour="green"),size=1)+
    geom_segment(aes(x=min(log(b$ocw)^(2/3)),xend=max(log(b$ocw)^(2/3)),
                     y=predict(fit.phylo.ou,new=data.frame(ocw=min(b$ocw))),
                     yend=predict(fit.phylo.ou,new=data.frame(ocw=max(b$ocw))),
                     col="#C77CFF"),linetype="dashed",size=1)+
    scale_fill_manual(values=c("light gray", "black"),
                      labels=c("Therians","Monotremes"))+
    labs(fill="Group",shape="Group",color="Regression")+
    scale_color_identity(name="Regression",labels=c(red="OLS",green="Brownian no monotremes",blue="Brownian with monotremes","#C77CFF"="OU model"), guide="legend")+
    guides(colour = guide_legend(reverse = T))+
    theme(legend.position=c(.8,.3),legend.spacing.y = unit(.1, "cm")))
```

Figure 9. Linear regression of the dataset under OLS (in red), PGLS under a Brownian model (in blue), and PGLS under a Brownian model excluding the monotreme taxa *Ornithorhynchus anatinus* and *Tachyglossus aculeatus* (taxa denoted in black), showing how the inclusion of monotreme taxa greatly biases the regression line due to the deep divergence between Theria and Monotremata.

## 13.8 PGLS under different models

### 13.8.1 Linear fit

```
fit.phylo.linear<-gls(log(bm)~I(log(ocw)^(1)),data=signaldataset,correlation=corBrownian(1,trees[[1]],form=~species))
summary(fit.phylo.linear)
```

```
Generalized least squares fit by REML
  Model: log(bm) ~ I(log(ocw)^(1)) 
  Data: signaldataset 
       AIC      BIC    logLik
  419.6031 431.5925 -206.8015

Correlation Structure: corBrownian
 Formula: ~species 
 Parameter estimate(s):
numeric(0)

Coefficients:
                    Value Std.Error  t-value p-value
(Intercept)     -3.503050 0.7225428 -4.84823       0
I(log(ocw)^(1))  3.591568 0.0597691 60.09071       0

 Correlation: 
                (Intr)
I(log(ocw)^(1)) -0.238

Standardized residuals:
       Min         Q1        Med         Q3        Max 
-0.5896115  0.1040837  0.2716052  0.4623266  1.1761027 

Residual standard error: 1.408781 
Degrees of freedom: 404 total; 402 residual
```

### 13.8.2 1/3 power fit

```
fit.phylo.power13<-gls(log(bm)~I(log(ocw)^(1/3)),data=signaldataset,correlation=corBrownian(1,trees[[1]],form=~species))
summary(fit.phylo.power13)
```

```
Generalized least squares fit by REML
  Model: log(bm) ~ I(log(ocw)^(1/3)) 
  Data: signaldataset 
       AIC      BIC    logLik
  366.9742 378.9635 -180.4871

Correlation Structure: corBrownian
 Formula: ~species 
 Parameter estimate(s):
numeric(0)

Coefficients:
                      Value Std.Error   t-value p-value
(Intercept)       -25.49309 0.8300269 -30.71357       0
I(log(ocw)^(1/3))  22.90312 0.3563693  64.26793       0

 Correlation: 
                  (Intr)
I(log(ocw)^(1/3)) -0.606

Standardized residuals:
        Min          Q1         Med          Q3         Max 
-0.79496234  0.09399651  0.28011251  0.52367444  1.27481401 

Residual standard error: 1.325592 
Degrees of freedom: 404 total; 402 residual
```

### 13.8.3 Accuracy statistics

```
fit.phylo.quadratic<-gls(log(bm)~I(log(ocw)^2)+log(ocw),data=b,
               correlation=corBrownian(1,trees[[1]],form=~species))
cbind(model=c("OLS","OLS","OLS","Brownian","Brownian","Brownian","Brownian","Brownian","OU Model"),
      data=c("all species","therians","all (w. shape)","all species","therians","all species","all species","all species","all species"),
      power=c("2/3","2/3","2/3","2/3","2/3","linear","quadratic","1/3","2/3"),
rbind(regression.stats(fit.all),
      regression.stats(fit.therian),
      regression.stats(fit.rabbit),
      regression.stats.gls(fit.phylo),
      regression.stats.gls(fit.phylo.therian),
      regression.stats.gls(fit.phylo.linear),
      regression.stats.gls(fit.phylo.quadratic),
      regression.stats.gls(fit.phylo.power13),
      regression.stats.gls(fit.phylo.ou)))
```

“OLS all species inc. shape, 2/3 power model” refers to fit.rabbit, the model including an additional categorical variable to parse out the unusual occiputs of monotremes and taxa with “rabbit-like” condyles. The model name is shortened here to make the accuracy statistics easier to see.

## 13.9 Examining log-quadratic model under PGLS

### 13.9.1 Phylogenetic generalized least squares (PGLS)

```
summary(fit.phylo.quadratic)
```

```
Generalized least squares fit by REML
  Model: log(bm) ~ I(log(ocw)^2) + log(ocw) 
  Data: b 
       AIC      BIC    logLik
  359.2197 375.1955 -175.6098

Correlation Structure: corBrownian
 Formula: ~species 
 Parameter estimate(s):
numeric(0)

Coefficients:
                  Value Std.Error  t-value p-value
(Intercept)   -6.914851 0.7765987 -8.90402       0
I(log(ocw)^2) -0.339541 0.0398173 -8.52749       0
log(ocw)       5.823037 0.2674092 21.77576       0

 Correlation: 
              (Intr) I(()^2
I(log(ocw)^2)  0.515       
log(ocw)      -0.546 -0.979

Standardized residuals:
        Min          Q1         Med          Q3         Max 
-0.82800119  0.08788309  0.28748906  0.52929519  1.24354609 

Residual standard error: 1.297767 
Degrees of freedom: 404 total; 401 residual
```

```
regression.stats.gls(fit.phylo.quadratic)
```

### 13.9.2 Under Ornstein-Uhlenbeck model

```
fit.phylo.ou.quadratic<-gls(log(bm)~I(log(ocw)^2)+log(ocw),data=signaldataset,
                correlation=corMartins(1,trees[[1]],form=~species,fixed=TRUE))
summary(fit.phylo.ou.quadratic)
```

```
Generalized least squares fit by REML
  Model: log(bm) ~ I(log(ocw)^2) + log(ocw) 
  Data: signaldataset 
       AIC      BIC    logLik
  402.4425 418.4184 -197.2213

Correlation Structure: corMartins
 Formula: ~species 
 Parameter estimate(s):
alpha 
    1 

Coefficients:
                  Value  Std.Error    t-value p-value
(Intercept)   -4.739440 0.22761351 -20.822315       0
I(log(ocw)^2) -0.192216 0.02591758  -7.416416       0
log(ocw)       4.757083 0.15665837  30.365971       0

 Correlation: 
              (Intr) I(()^2
I(log(ocw)^2)  0.944       
log(ocw)      -0.983 -0.987

Standardized residuals:
        Min          Q1         Med          Q3         Max 
-3.41026734 -0.63474608 -0.07936711  0.47856151  2.90617691 

Residual standard error: 0.3886408 
Degrees of freedom: 404 total; 401 residual
```

```
regression.stats.gls(fit.phylo.ou.quadratic)
```

### 13.9.3 Scatterplot of PGLS regression versus OLS regression

```
fit.phylo.therian.quadratic<-gls(log(bm)~I(log(ocw)^2)+log(ocw),data=therian,correlation=corBrownian(1,trees.theria[[1]],form=~species))
signaldataset.therian.quadratic<-data.frame(therian,residuals=fit.therian$residuals,row.names = (therian$species))
```

```
plot_data.phylo<-data.frame(signaldataset,predict(fit.phylo.quadratic,interval="prediction"))
(scatterplot.phylo.quadratic<-ggplot(plot_data.phylo,aes(log(ocw),log(bm)))+
    geom_point(aes(group=monotreme,fill=monotreme),size=3,shape=21,col="black")+
    labs(x="Natural Log OCW (mm)",y="Natural Log Body Mass (g)",show.legend=TRUE)+
    scale_x_continuous(breaks=seq(1,6,0.5))+
    scale_y_continuous(breaks=seq(0,17,1))+
    geom_point(data=b %>% filter(group=="Monotremata"),
               shape=21,size=4,fill="black",col="white")+
    theme_classic()+
    geom_smooth(formula=y~I(x^2)+x,method="lm",se=F,
                aes(col="red"),size=1)+  
    stat_function(fun = function(x) fit.phylo.quadratic$coefficients[1] +
                    fit.phylo.quadratic$coefficients[2] * x^2 +
                    fit.phylo.quadratic$coefficients[3] * x,
                 aes(col="blue"),size=1)+
    stat_function(fun = function(x) fit.phylo.therian.quadratic$coefficients[1] +
                    fit.phylo.therian.quadratic$coefficients[2] * x^2 +
                    fit.phylo.therian.quadratic$coefficients[3] * x,
                  aes(col="green"),size=1)+
    stat_function(fun = function(x) fit.phylo.ou.quadratic$coefficients[1] +
                    fit.phylo.ou.quadratic$coefficients[2] * x^2 +
                    fit.phylo.ou.quadratic$coefficients[3] * x,
                  aes(col="#C77CFF"),linetype="dashed",size=1)+
    scale_fill_manual(values=c("light gray", "black"),
                      labels=c("Therians","Monotremes"))+
    labs(fill="Group",shape="Group",color="Regression")+
    scale_color_identity(name="Regression",labels=c(red="OLS",green="Brownian no monotremes",blue="Brownian with monotremes","#C77CFF"="OU model"), guide="legend")+
    guides(colour = guide_legend(reverse = T))+
    theme(legend.position=c(.8,.3),legend.spacing.y = unit(.1, "cm")))
```

Comparison of log-quadratic regression line under OLS (in red), compared to log-quadratic PGLS models under Brownian motion (in blue), Brownian motion excluding Monotremata (line in green, monotreme taxa denoted in black) and an Ornstein-Uhlenbeck model (in purple).

# 14 Comparing OCW and body mass to head-body length (HBL) and condylobasal length

## 14.1 Skull (condylobasal) length versus body mass

```
nls(log(bm)~a*log(skull_length)^b+c,data=b,start=list(a=1,b=1,c=1))
```

```
Nonlinear regression model
  model: log(bm) ~ a * log(skull_length)^b + c
   data: b
       a        b        c 
 18.0171   0.4346 -26.8848 
 residual sum-of-squares: 89.36

Number of iterations to convergence: 13 
Achieved convergence tolerance: 0.000002423
```

```
confint2(nls(log(bm)~a*log(skull_length)^b+c,data=b,start=list(a=1,b=1,c=1)))
```

```
        2.5 %      97.5 %
a   8.0589419  27.9753081
b   0.2881211   0.5811162
c -38.3664132 -15.4032713
```

```
fit.skull_length<-lm(log(bm)~I(log(skull_length)^(1/2)),data=b)
summary(fit.skull_length)
```

```
Call:
lm(formula = log(bm) ~ I(log(skull_length)^(1/2)), data = b)

Residuals:
     Min       1Q   Median       3Q      Max 
-1.71786 -0.29780  0.00423  0.31898  1.47482 

Coefficients:
                           Estimate Std. Error t value            Pr(>|t|)    
(Intercept)                -22.4349     0.2516  -89.18 <0.0000000000000002 ***
I(log(skull_length)^(1/2))  14.2241     0.1199  118.65 <0.0000000000000002 ***
---
Signif. codes:  0 '***' 0.001 '**' 0.01 '*' 0.05 '.' 0.1 ' ' 1

Residual standard error: 0.4719 on 402 degrees of freedom
Multiple R-squared:  0.9722,    Adjusted R-squared:  0.9722 
F-statistic: 1.408e+04 on 1 and 402 DF,  p-value: < 0.00000000000000022
```

```
par(mfrow=c(2,2))
plot(fit.skull_length)
```

Significance of second-order term in log-quadratic model.

```
summary(lm(log(bm)~poly(log(skull_length),2),data=b))
```

```
Call:
lm(formula = log(bm) ~ poly(log(skull_length), 2), data = b)

Residuals:
     Min       1Q   Median       3Q      Max 
-1.74025 -0.30577  0.00935  0.30605  1.55623 

Coefficients:
                            Estimate Std. Error t value             Pr(>|t|)    
(Intercept)                  7.28523    0.02348 310.230 < 0.0000000000000002 ***
poly(log(skull_length), 2)1 55.88430    0.47201 118.397 < 0.0000000000000002 ***
poly(log(skull_length), 2)2 -3.58904    0.47201  -7.604    0.000000000000206 ***
---
Signif. codes:  0 '***' 0.001 '**' 0.01 '*' 0.05 '.' 0.1 ' ' 1

Residual standard error: 0.472 on 401 degrees of freedom
Multiple R-squared:  0.9723,    Adjusted R-squared:  0.9722 
F-statistic:  7038 on 2 and 401 DF,  p-value: < 0.00000000000000022
```

### 14.1.1 Accuracy statistics and Akaike Information Criterion for several regression models

```
fit.skull_length.line<-lm(log(bm)~I(log(skull_length)),data=b)
fit.skull_length.power12<-lm(log(bm)~I(log(skull_length)^(1/2)),data=b)
fit.skull_length.power23<-lm(log(bm)~I(log(skull_length)^(2/3)),data=b)
fit.skull_length.power34<-lm(log(bm)~I(log(skull_length)^(3/4)),data=b)
fit.skull_length.quadratic<-lm(log(bm)~poly(log(skull_length),2),data=b)
rbind("Skull length, log-linear model"=regression.stats(fit.skull_length.line),
      "Skull length, 1/2 power model"=regression.stats(fit.skull_length.power12),
      "Skull length, 2/3 power model"=regression.stats(fit.skull_length.power23),
      "Skull length, 3/4 power model"=regression.stats(fit.skull_length.power34),
      "Skull length, log-quadratic"=regression.stats(fit.skull_length.quadratic))
```

### 14.1.2 Effects of second-order term in log-quadratic model

```
summary(fit.skull_length.quadratic)
```

```
Call:
lm(formula = log(bm) ~ poly(log(skull_length), 2), data = b)

Residuals:
     Min       1Q   Median       3Q      Max 
-1.74025 -0.30577  0.00935  0.30605  1.55623 

Coefficients:
                            Estimate Std. Error t value             Pr(>|t|)    
(Intercept)                  7.28523    0.02348 310.230 < 0.0000000000000002 ***
poly(log(skull_length), 2)1 55.88430    0.47201 118.397 < 0.0000000000000002 ***
poly(log(skull_length), 2)2 -3.58904    0.47201  -7.604    0.000000000000206 ***
---
Signif. codes:  0 '***' 0.001 '**' 0.01 '*' 0.05 '.' 0.1 ' ' 1

Residual standard error: 0.472 on 401 degrees of freedom
Multiple R-squared:  0.9723,    Adjusted R-squared:  0.9722 
F-statistic:  7038 on 2 and 401 DF,  p-value: < 0.00000000000000022
```

Breusch-Pagel test of untransformed log skull length versus transformed skull length

```
bptest(fit.skull_length.line)
```

```
    studentized Breusch-Pagan test

data:  fit.skull_length.line
BP = 9.0425, df = 1, p-value = 0.002638
```

```
bptest(fit.skull_length)
```

```
    studentized Breusch-Pagan test

data:  fit.skull_length
BP = 8.0292, df = 1, p-value = 0.004603
```

### 14.1.3 Skull length excluding Primates

```
fit.skull_length.noprimates<-lm(log(bm)~I(log(skull_length)^(1/2)),data=b %>% filter(group!="Primates"))
summary(fit.skull_length.noprimates)
```

```
Call:
lm(formula = log(bm) ~ I(log(skull_length)^(1/2)), data = b %>% 
    filter(group != "Primates"))

Residuals:
     Min       1Q   Median       3Q      Max 
-1.66453 -0.28930  0.00274  0.28392  1.54769 

Coefficients:
                           Estimate Std. Error t value            Pr(>|t|)    
(Intercept)                -22.3646     0.2492  -89.73 <0.0000000000000002 ***
I(log(skull_length)^(1/2))  14.1665     0.1191  118.99 <0.0000000000000002 ***
---
Signif. codes:  0 '***' 0.001 '**' 0.01 '*' 0.05 '.' 0.1 ' ' 1

Residual standard error: 0.4615 on 358 degrees of freedom
Multiple R-squared:  0.9753,    Adjusted R-squared:  0.9753 
F-statistic: 1.416e+04 on 1 and 358 DF,  p-value: < 0.00000000000000022
```

```
regression.stats(fit.skull_length.noprimates)
```

### 14.1.4 Average residuals by order (skull length)

```
b %>%
  mutate(residuals.skull=fit.skull_length$residuals) %>%
  mutate(PEcf=abs((exp(fit.skull_length$fitted.values)*regression.stats(fit.skull_length)$CF)-b$skull_length)/
           (exp(fit.skull_length$fitted.values)*regression.stats(fit.skull_length)$CF))%>%
  group_by(group) %>%
  summarise(residuals=round(mean(residuals.skull),4),
            "%PEcf"=round(mean(PEcf)*100,2),
            N=n())
```

## 14.2 Head-body length versus body mass

```
nls(log(bm)~a*log(hbl)^b+c,data=b,start=list(a=1,b=1,c=1))
```

```
Nonlinear regression model
  model: log(bm) ~ a * log(hbl)^b + c
   data: b
       a        b        c 
  3.7468   0.9181 -11.8832 
 residual sum-of-squares: 72.31

Number of iterations to convergence: 5 
Achieved convergence tolerance: 0.0000000821
```

```
confint2(nls(log(bm)~a*log(hbl)^b+c,data=b,start=list(a=1,b=1,c=1)))
```

```
        2.5 %    97.5 %
a   2.0363617  5.457162
b   0.7585989  1.077585
c -15.1701928 -8.596144
```

```
fit.hbl<-lm(log(bm)~I(log(hbl)),data=b)
summary(fit.hbl)
```

```
Call:
lm(formula = log(bm) ~ I(log(hbl)), data = b)

Residuals:
    Min      1Q  Median      3Q     Max 
-1.3899 -0.2655 -0.0476  0.2296  1.5738 

Coefficients:
            Estimate Std. Error t value            Pr(>|t|)    
(Intercept) -10.3386     0.1349  -76.61 <0.0000000000000002 ***
I(log(hbl))   2.9749     0.0225  132.22 <0.0000000000000002 ***
---
Signif. codes:  0 '***' 0.001 '**' 0.01 '*' 0.05 '.' 0.1 ' ' 1

Residual standard error: 0.4247 on 402 degrees of freedom
Multiple R-squared:  0.9775,    Adjusted R-squared:  0.9775 
F-statistic: 1.748e+04 on 1 and 402 DF,  p-value: < 0.00000000000000022
```

```
par(mfrow=c(2,2))
plot(fit.hbl)
```

```
regression.stats(fit.hbl)
```

### 14.2.1 Accuracy statistics and Akaike Information Criterion for several alternate regression models

```
fit.hbl.power12<-lm(log(bm)~I(log(hbl)^(1/2)),data=b) #1/2 power model
fit.hbl.power23<-lm(log(bm)~I(log(hbl)^(2/3)),data=b) #2/3 power model
fit.hbl.power34<-lm(log(bm)~I(log(hbl)^(3/4)),data=b) #3/4 power model
fit.hbl.line<-lm(log(bm)~I(log(hbl)),data=b) #Linear model)
rbind("HBL, linear model"=regression.stats(fit.hbl.line),
      "HBL, 1/2 power model"=regression.stats(fit.hbl.power12),
      "HBL, 2/3 power model"=regression.stats(fit.hbl.power23),
      "HBL, 3/4 power model"=regression.stats(fit.hbl.power34))
```

### 14.2.2 Average residuals by order (HBL)

```
b %>%
  mutate(residuals.hbl=fit.hbl$residuals) %>%
  mutate(PEcf=abs((exp(fit.hbl$fitted.values)*regression.stats(fit.hbl)$CF)-b$hbl)/
           (exp(fit.hbl$fitted.values)*regression.stats(fit.hbl)$CF))%>%
  group_by(group) %>%
  summarise(residuals=round(mean(residuals.hbl),4),
            "%PEcf"=round(mean(PEcf)*100,2),
            N=n())
```

### 14.2.3 HBL excluding Primates

```
fit.hbl.noprimates<-lm(log(bm)~log(hbl),data=b %>% filter(group!="Primates"))
summary(fit.hbl.noprimates)
```

```
Call:
lm(formula = log(bm) ~ log(hbl), data = b %>% filter(group != 
    "Primates"))

Residuals:
     Min       1Q   Median       3Q      Max 
-1.34150 -0.22869 -0.00258  0.20293  1.69578 

Coefficients:
             Estimate Std. Error t value            Pr(>|t|)    
(Intercept) -10.26500    0.12139  -84.56 <0.0000000000000002 ***
log(hbl)      2.95110    0.02029  145.46 <0.0000000000000002 ***
---
Signif. codes:  0 '***' 0.001 '**' 0.01 '*' 0.05 '.' 0.1 ' ' 1

Residual standard error: 0.3791 on 358 degrees of freedom
Multiple R-squared:  0.9834,    Adjusted R-squared:  0.9833 
F-statistic: 2.116e+04 on 1 and 358 DF,  p-value: < 0.00000000000000022
```

```
regression.stats(fit.hbl.noprimates)
```

## 14.3 OCW compared to other linear variables

### 14.3.1 OCW versus condylobasal length

```
fit.skull_length.ocw<-lm(log(skull_length)~log(ocw),data=b)
summary(fit.skull_length.ocw)
```

```
Call:
lm(formula = log(skull_length) ~ log(ocw), data = b)

Residuals:
     Min       1Q   Median       3Q      Max 
-0.42773 -0.10460 -0.00374  0.09405  0.53483 

Coefficients:
            Estimate Std. Error t value            Pr(>|t|)    
(Intercept)  1.38825    0.02944   47.16 <0.0000000000000002 ***
log(ocw)     1.04366    0.00984  106.06 <0.0000000000000002 ***
---
Signif. codes:  0 '***' 0.001 '**' 0.01 '*' 0.05 '.' 0.1 ' ' 1

Residual standard error: 0.1531 on 402 degrees of freedom
Multiple R-squared:  0.9655,    Adjusted R-squared:  0.9654 
F-statistic: 1.125e+04 on 1 and 402 DF,  p-value: < 0.00000000000000022
```

```
regression.stats.skull_length(fit.skull_length.ocw)
```

### 14.3.2 OCW versus HBL

```
fit.hbl.ocw<-lm(log(hbl)~I(log(ocw)^(2/3)),data=b)
summary(fit.hbl.ocw)
```

```
Call:
lm(formula = log(hbl) ~ I(log(ocw)^(2/3)), data = b)

Residuals:
     Min       1Q   Median       3Q      Max 
-0.55039 -0.10331  0.00082  0.10483  0.51547 

Coefficients:
                  Estimate Std. Error t value            Pr(>|t|)    
(Intercept)        0.82550    0.05027   16.42 <0.0000000000000002 ***
I(log(ocw)^(2/3))  2.53378    0.02458  103.07 <0.0000000000000002 ***
---
Signif. codes:  0 '***' 0.001 '**' 0.01 '*' 0.05 '.' 0.1 ' ' 1

Residual standard error: 0.1798 on 402 degrees of freedom
Multiple R-squared:  0.9635,    Adjusted R-squared:  0.9634 
F-statistic: 1.062e+04 on 1 and 402 DF,  p-value: < 0.00000000000000022
```

```
regression.stats.hbl(fit.hbl.ocw)
```

```
b %>%
  mutate(residuals.hbl=fit.hbl.ocw$residuals) %>%
  group_by(group) %>%
  summarise(residuals=mean(residuals.hbl),N=n())%>%
  arrange(desc(abs(residuals)))
```

## 14.4 Multivariate equation with OCW, HBL, and condylobasal length

```
fit.three<-lm(log(bm)~log(hbl)+I(log(ocw)^(2/3))+I(log(skull_length)^(1/2)),data=b)
summary(fit.three)
```

```
Call:
lm(formula = log(bm) ~ log(hbl) + I(log(ocw)^(2/3)) + I(log(skull_length)^(1/2)), 
    data = b)

Residuals:
     Min       1Q   Median       3Q      Max 
-0.95326 -0.21989  0.01482  0.18041  0.76521 

Coefficients:
                           Estimate Std. Error t value             Pr(>|t|)    
(Intercept)                -11.1212     0.4907 -22.664 < 0.0000000000000002 ***
log(hbl)                     1.1223     0.1038  10.812 < 0.0000000000000002 ***
I(log(ocw)^(2/3))            3.8162     0.2441  15.632 < 0.0000000000000002 ***
I(log(skull_length)^(1/2))   1.9521     0.5172   3.774             0.000185 ***
---
Signif. codes:  0 '***' 0.001 '**' 0.01 '*' 0.05 '.' 0.1 ' ' 1

Residual standard error: 0.2997 on 400 degrees of freedom
Multiple R-squared:  0.9889,    Adjusted R-squared:  0.9888 
F-statistic: 1.184e+04 on 3 and 400 DF,  p-value: < 0.00000000000000022
```

```
rbind("OCW only"=regression.stats(fit.all),"OCW+HBL+skull length"=regression.stats(fit.three))
```

## 14.5 Covariance between residuals

```
cov(fit.all$residuals,fit.skull_length$residuals) #covariance between OCW and skull length
```

```
[1] 0.05379203
```

```
cov(fit.all$residuals,fit.hbl$residuals) #covariance between OCW and HBL
```

```
[1] 0.0215484
```

```
cov(fit.skull_length$residuals,fit.hbl$residuals) #covariance between HBL and skull length
```

```
[1] 0.0909295
```

```
b$residuals<-fit.all$residuals
b$residuals.hbl<-fit.hbl$residuals
b$residuals.skull_length<-fit.skull_length$residuals

summary(lm(residuals~residuals.hbl,data=b))
```

```
Call:
lm(formula = residuals ~ residuals.hbl, data = b)

Residuals:
     Min       1Q   Median       3Q      Max 
-1.28003 -0.22784 -0.03372  0.20788  1.14981 

Coefficients:
                             Estimate              Std. Error t value Pr(>|t|)   
(Intercept)   -0.00000000000000001436  0.01923199734792445034   0.000  1.00000   
residuals.hbl  0.11978561654708304307  0.04540016164074540256   2.638  0.00865 **
---
Signif. codes:  0 '***' 0.001 '**' 0.01 '*' 0.05 '.' 0.1 ' ' 1

Residual standard error: 0.3866 on 402 degrees of freedom
Multiple R-squared:  0.01702,   Adjusted R-squared:  0.01458 
F-statistic: 6.961 on 1 and 402 DF,  p-value: 0.008652
```

```
summary(lm(residuals~residuals.skull_length,data=b))
```

```
Call:
lm(formula = residuals ~ residuals.skull_length, data = b)

Residuals:
     Min       1Q   Median       3Q      Max 
-1.17715 -0.23027 -0.02614  0.21551  1.05393 

Coefficients:
                                      Estimate              Std. Error t value      Pr(>|t|)    
(Intercept)            -0.00000000000000001055  0.01854609303752978791   0.000             1    
residuals.skull_length  0.24211250302556466818  0.03939492233092518197   6.146 0.00000000192 ***
---
Signif. codes:  0 '***' 0.001 '**' 0.01 '*' 0.05 '.' 0.1 ' ' 1

Residual standard error: 0.3728 on 402 degrees of freedom
Multiple R-squared:  0.08589,   Adjusted R-squared:  0.08361 
F-statistic: 37.77 on 1 and 402 DF,  p-value: 0.000000001918
```

```
summary(lm(residuals.hbl~residuals.skull_length,data=b))
```

```
Call:
lm(formula = residuals.hbl ~ residuals.skull_length, data = b)

Residuals:
    Min      1Q  Median      3Q     Max 
-1.3756 -0.2243 -0.0219  0.2047  1.2980 

Coefficients:
                                     Estimate             Std. Error t value            Pr(>|t|)    
(Intercept)            0.00000000000000002847 0.01881594389522156865    0.00                   1    
residuals.skull_length 0.40926453767511250526 0.03996812950497467126   10.24 <0.0000000000000002 ***
---
Signif. codes:  0 '***' 0.001 '**' 0.01 '*' 0.05 '.' 0.1 ' ' 1

Residual standard error: 0.3782 on 402 degrees of freedom
Multiple R-squared:  0.2069,    Adjusted R-squared:  0.2049 
F-statistic: 104.9 on 1 and 402 DF,  p-value: < 0.00000000000000022
```

```
resid2<-grid.arrange(
  ggplot(b,aes(residuals,residuals.skull_length))+
    geom_point(size=3,shape=21,fill="gray")+
    theme_classic()+
    geom_hline(aes(yintercept=0),color="black", linetype="dashed", size=0.5)+
    geom_vline(aes(xintercept=0),color="black", linetype="dashed", size=0.5)+
    scale_y_continuous(limits=c(-1.75,1.75),breaks=seq(-2,2,0.5))+
    coord_fixed()+
    ggtitle("A")+
    labs(x="OCW Residuals",y="Skull Length Residuals"),
  ggplot(b,aes(residuals,residuals.hbl))+
    geom_point(size=3,shape=21,fill="gray")+
    theme_classic()+
    geom_hline(aes(yintercept=0),color="black", linetype="dashed", size=0.5)+
    geom_vline(aes(xintercept=0),color="black", linetype="dashed", size=0.5)+
    scale_y_continuous(limits=c(-1.75,1.75),breaks=seq(-2,2,0.5))+
    coord_fixed()+
    ggtitle("B")+
    labs(x="OCW Residuals",y="Head-Body Length Residuals"),
  nrow=1)
```

Figure S10. Plot of the residuals for OCW against the residuals for the regressions of head-body length (**A**) and skull length (**B**).

# 15 Using OCW to predict body mass of *Hyaenodon*

```
hyaenodon<-data.frame(row.names=c("Hyaenodon_crucians","Hyaenodon_horridus"),
                                   ocw=c(32,42),
                                   monotreme=c("No","No"),
                                   rabbit=c("No","No"))
```

These data were obtained from figured occiputs of *Hyaenodon* in Lange-Badré (1979)

**Predicting body mass in *Hyaenodon* using the all-species equation**

```
exp(predict(fit.all,hyaenodon,interval="prediction"))*regression.stats(fit.all)$CF
```

```
                        fit       lwr     upr
Hyaenodon_crucians 12949.05  6007.701 27910.5
Hyaenodon_horridus 32167.15 14915.412 69372.9
```

**Predicting body mass in *Hyaenodon* using the all-species equation with additional characters for condyle shape**

```
exp(predict(fit.rabbit,hyaenodon,interval="prediction"))*regression.stats(fit.rabbit)$CF
```

```
                        fit       lwr      upr
Hyaenodon_crucians 12591.01  6366.314 24901.93
Hyaenodon_horridus 31522.64 15930.590 62375.40
```

**Predicting body mass in *Hyaenodon* using the Carnivora-only equation**

```
exp(predict(fit.carnivora,hyaenodon,interval="prediction"))*regression.stats(fit.carnivora)$CF
```

```
                        fit       lwr      upr
Hyaenodon_crucians 12361.47  6915.208 22097.10
Hyaenodon_horridus 34125.80 19031.175 61192.76
```

**Predicting body mass in *Hyaenodon* using the PGLS equation**

```
data.frame(OLS=exp(predict(fit.all,hyaenodon))*regression.stats(fit.all)$CF,PGLS=exp(predict(fit.phylo,hyaenodon,interval="prediction"))*regression.stats.gls(fit.phylo)$CF)%>%
  mutate(Percent_Dif=round((OLS-PGLS)/OLS,4)*100)
```

# 16 Saving figures produced by script

# 17 Session Information

```
xfun::session_info()
```

```
R version 4.1.1 (2021-08-10)
Platform: x86_64-w64-mingw32/x64 (64-bit)
Running under: Windows 10 x64 (build 19042)

Locale: LC_COLLATE=English_United States.1252  LC_CTYPE=English_United States.1252    LC_MONETARY=English_United States.1252 LC_NUMERIC=C                           LC_TIME=English_United States.1252    

Package version:
  ape_5.5                 aplot_0.1.1             askpass_1.1             assertthat_0.2.1        backports_1.2.1         base64enc_0.1.3         bit_4.0.4               bit64_4.0.5             blob_1.2.2              bookdown_0.24           broom_0.7.10            broom.mixed_0.2.7       bslib_0.3.0             callr_3.7.0             caper_1.0.1             caret_6.0-89            cellranger_1.1.0        class_7.3-19            cli_3.0.1               clipr_0.7.1             clusterGeneration_1.3.7 coda_0.19-4             codetools_0.2-18        colorspace_2.0-2        combinat_0.0-8          compiler_4.1.1          corpcor_1.6.10          cpp11_0.4.1             crayon_1.4.2            crosstalk_1.2.0         cubature_2.0.4.2        curl_4.3.2              data.table_1.14.2       DBI_1.1.1               dbplyr_2.1.1            deSolve_1.29            digest_0.6.28           dplyr_1.0.7             DT_0.19                 dtplyr_1.1.0            e1071_1.7-9            
  ellipsis_0.3.2          evaluate_0.14           expm_0.999-6            fansi_0.5.0             farver_2.1.0            fastmap_1.1.0           fastmatch_1.1-3         FNN_1.1.3               forcats_0.5.1           foreach_1.5.1           fs_1.5.0                future_1.22.1           future.apply_1.8.1      gargle_1.2.0            geiger_2.0.7            generics_0.1.1          ggfortify_0.4.12        ggfun_0.0.4             ggplot2_3.3.5           ggplotify_0.1.0         ggtree_3.0.4            globals_0.14.0          glue_1.4.2              googledrive_2.0.0       googlesheets4_1.0.0     gower_0.2.2             graphics_4.1.1          grDevices_4.1.1         grid_4.1.1              gridExtra_2.3           gridGraphics_0.5-1      gtable_0.3.0            haven_2.4.3             highr_0.9               hms_1.1.1               htmltools_0.5.2         htmlwidgets_1.5.4       httr_1.4.2              ids_1.0.1               igraph_1.2.6            ipred_0.9-12           
  isoband_0.2.5           iterators_1.0.13        jquerylib_0.1.4         jsonlite_1.7.2          kableExtra_1.3.4        kernlab_0.9.29          KernSmooth_2.23-20      knitr_1.36              ks_1.13.2               labeling_0.4.2          later_1.3.0             lattice_0.20-45         lava_1.6.10             lazyeval_0.2.2          lifecycle_1.0.1         listenv_0.8.0           lmtest_0.9-38           lubridate_1.7.10        magrittr_2.0.1          maps_3.4.0              MASS_7.3-54             Matrix_1.3-4            mclust_5.4.7            MCMCglmm_2.32           methods_4.1.1           mgcv_1.8-37             mime_0.12               misc3d_0.9.0            mnormt_2.0.2            ModelMetrics_1.2.2.2    modelr_0.1.8            motmot_2.1.3            multicool_0.1.12        munsell_0.5.0           mvtnorm_1.1-2           ncbit_2013.3.29         nlme_3.1-153            nlstools_2.0-0          nnet_7.3-16             numDeriv_2016.8-1.1     openssl_1.4.5          
  parallel_4.1.1          parallelly_1.28.1       patchwork_1.1.1         phangorn_2.7.1          phylolm_2.6.2           phytools_0.7-90         pillar_1.6.4            pkgconfig_2.0.3         plot3D_1.4              plotrix_3.8-2           plyr_1.8.6              pracma_2.3.3            prettyunits_1.1.1       pROC_1.18.0             processx_3.5.2          prodlim_2019.11.13      progress_1.2.2          progressr_0.9.0         promises_1.2.0.1        proxy_0.4-26            ps_1.6.0                purrr_0.3.4             quadprog_1.5-8          R6_2.5.1                rappdirs_0.3.3          RColorBrewer_1.1.2      Rcpp_1.0.7              readr_2.0.2             readxl_1.3.1            recipes_0.1.17          rematch_1.0.1           rematch2_2.1.2          reprex_2.0.1            reshape2_1.4.4          rlang_0.4.11            rmarkdown_2.11          rmdformats_1.0.3.9000   rpart_4.1-15            rstudioapi_0.13         rvest_1.0.2             sass_0.4.0             
  scales_1.1.1            scatterplot3d_0.3-41    selectr_0.4.2           sensiPhy_0.8.5          splines_4.1.1           SQUAREM_2021.1          stats_4.1.1             stats4_4.1.1            stringi_1.7.4           stringr_1.4.0           subplex_1.6             survival_3.2-13         svglite_2.0.0           sys_3.4                 systemfonts_1.0.2       tcltk_4.1.1             tensorA_0.36.2          tibble_3.1.5            tidyr_1.1.4             tidyselect_1.1.1        tidytree_0.3.5          tidyverse_1.3.1         timeDate_3043.102       tinytex_0.35            tmvnsim_1.0-2           tools_4.1.1             treeio_1.16.2           tzdb_0.1.2              utf8_1.2.2              utils_4.1.1             uuid_1.0.3              vctrs_0.3.8             viridisLite_0.4.0       vroom_1.5.5             webshot_0.5.2           withr_2.4.2             xfun_0.26               xml2_1.3.2              yaml_2.2.1              yulab.utils_0.0.2       zoo_1.8-9
```

×

- 1 Installing R Packages
- 2 Importing and subsetting dataset
  - 2.1 Importing dataset
  - 2.2 Subsetting data for necessary analyses
  - 2.3 Importing phylogenetic data
  - 2.4 Code Book
- 3 Examining non-linearity of the data
  - 3.1 Fitting Models
  - 3.2 Distribution of data
  - 3.3 Box-cox plots
  - 3.4 Scatterplot of log-transformed data
  - 3.5 Akaike Information Criterion and accuracy statistics for various alternative regression models
  - 3.6 Plot of Ln OCW versus Ln body mass showing non-linearity
  - 3.7 Residuals vs. Fitted Graphs
  - 3.8 Regression plot of other variables and untransformed variables
  - 3.9 Examining effects of non-linearity on predicting OCW and body mass
  - 3.10 Non-linear curve fitting for OCW, HBL, and skull length
    - 3.10.1 Curve fit for OCW, all species
    - 3.10.2 Curve fit for OCW, therians only
    - 3.10.3 Curve fit for OCW, reduced dataset
    - 3.10.4 Curve fit for condylobasal length of skull
    - 3.10.5 Curve fit for HBL
    - 3.10.6 Curve fit for OCW versus skull length
    - 3.10.7 Curve fit for OCW versus HBL
- 4 Analysis using species-average data for all species
  - 4.1 Average residuals by order (OCW)
  - 4.2 Predicted body mass and error of CF-adjusted predicted values
  - 4.3 Normality tests of residuals
  - 4.4 Histogram of residuals and Q-Q plot of species-average data
  - 4.5 Accuracy statistics
  - 4.6 Test of whether selectivity of specimens affects accuracy
  - 4.7 Plot of Residuals versus Sample Size
- 5 Examining interspecific differences in occiput shape
  - 5.1 Scatterplot color-coded by occiput morphology type
  - 5.2 Homogeneity of slopes test between specimens with rabbit-like occiputs and remaining sample
  - 5.3 Regression with extra variables for monotremes and rabbit-like taxa
    - 5.3.1 Fitted values with correction factor
- 6 Differences in regression slope and y-intercept between clades
  - 6.1 Testing for differences in intercept
  - 6.2 Testing for differences in slope
    - 6.2.1 Differences in slope between orders
  - 6.3 Scatterplot of groups color-coded by taxonomic suborder/order and ordinal regression equations
- 7 Subsetted species-average datasets
  - 7.1 Reduced dataset excluding taxa with apomorphic occiput morphology
  - 7.2 Therian-only dataset (excluding monotremes)
  - 7.3 Dataset of all species for which N ≥ 6
  - 7.4 Dataset of all species for which N ≥ 6, excluding taxa with specialized occiput morphology
  - 7.5 Dataset of all species for which N ≥ 10
  - 7.6 Dataset of all species for which N ≥ 10, excluding taxa with specialized occiput morphology
- 8 OCW scaling patterns in different size classes
  - 8.1 Plotting differences in slope between transformed and non-transformed datasets for different size classes
- 9 Regressions for taxonomic subsets
  - 9.1 Rodentia dataset
    - 9.1.1 Residuals for rodent-only regression
  - 9.2 Sciuromorpha dataset
  - 9.3 Carnivora dataset
  - 9.4 Ungulate dataset
  - 9.5 Primates dataset
  - 9.6 Australidelphia dataset
  - 9.7 Accuracy statistics for taxonomic subset regressions
  - 9.8 Regression lines for taxonomic subsets
- 10 Examining a log-quadratic model
  - 10.1 Statistical power of quadratic and cubic terms under log-quadratic and log-cubic models and summary plots
  - 10.2 Plot of leverage versus fitted values for log-quadratic model
  - 10.3 Log-quadratic fit lines for different orders
  - 10.4 Plotting log-quadratic model comparing rabbit-like taxa to non-rabbit-like taxa
  - 10.5 Log-quadratic fit lines for taxonomic subgroups
    - 10.5.1 Plot of log-quadratic regression models for Sciuromorpha compared to Rodentia and Mammalia
  - 10.6 Testing second-order term for various taxonomic groups
  - 10.7 Log-quadratic fit lines for superorders
    - 10.7.1 Testing for differences in intercept
    - 10.7.2 Testing for differences in slope
    - 10.7.3 Scatterplot with regression lines
- 11 Individual specimen-level variation and effect of captivity status
  - 11.1 Regression equation treating each specimen individually
  - 11.2 Regression using species averages of wild-caught specimens only
  - 11.3 Comparison of regression models between species-average of all specimens and species-average of all wild-caught specimens
  - 11.4 Test for if captivity has a significant effect
  - 11.5 Effects of captivity status
  - 11.6 Examining the effect of gender
- 12 Testing for relative brain size as potential confounding variable
  - 12.1 Subsetting data
  - 12.2 Control OCW regression only for taxa that have brain mass (but without brainmass included)
  - 12.3 Testing relative brain size against residuals of OCW regression
  - 12.4 Plot of relative brain size versus residuals of control OCW regression
  - 12.5 Testing relative brain size as an extra independent variable
    - 12.5.1 Mean percent difference in body mass estimates between regression equations with and without brain mass
    - 12.5.2 Plotting residuals with brain mass against residuals without brain mass
- 13 Test for phylogenetic signal and PGLS
  - 13.1 Testing for phylogenetic signal
  - 13.2 Showing variation in AIC across models
    - 13.2.1 Coefficients for PGLS models under 100 random trees
    - 13.2.2 Plotting variation in PGLS regression lines
    - 13.2.3 Plotting distribution of AIC values
  - 13.3 Visualizing phylogenetic signal of the residuals across tree
  - 13.4 Phylogenetic generalized least squares (PGLS)
  - 13.5 Under Ornstein-Uhlenbeck model
  - 13.6 PGLS excluding Monotremata
  - 13.7 Scatterplot of PGLS regression versus OLS regression
  - 13.8 PGLS under different models
    - 13.8.1 Linear fit
    - 13.8.2 1/3 power fit
    - 13.8.3 Accuracy statistics
  - 13.9 Examining log-quadratic model under PGLS
    - 13.9.1 Phylogenetic generalized least squares (PGLS)
    - 13.9.2 Under Ornstein-Uhlenbeck model
    - 13.9.3 Scatterplot of PGLS regression versus OLS regression
- 14 Comparing OCW and body mass to head-body length (HBL) and condylobasal length
  - 14.1 Skull (condylobasal) length versus body mass
    - 14.1.1 Accuracy statistics and Akaike Information Criterion for several regression models
    - 14.1.2 Effects of second-order term in log-quadratic model
    - 14.1.3 Skull length excluding Primates
    - 14.1.4 Average residuals by order (skull length)
  - 14.2 Head-body length versus body mass
    - 14.2.1 Accuracy statistics and Akaike Information Criterion for several alternate regression models
    - 14.2.2 Average residuals by order (HBL)
    - 14.2.3 HBL excluding Primates
  - 14.3 OCW compared to other linear variables
    - 14.3.1 OCW versus condylobasal length
    - 14.3.2 OCW versus HBL
  - 14.4 Multivariate equation with OCW, HBL, and condylobasal length
  - 14.5 Covariance between residuals
- 15 Using OCW to predict body mass of *Hyaenodon*
- 16 Saving figures produced by script
- 17 Session Information
